# The effect of acupuncture on quality of life in patients with irritable bowel syndrome: A systematic review and meta-analysis

**DOI:** 10.1371/journal.pone.0314678

**Published:** 2025-02-13

**Authors:** Jun Zhou, Narendra Lamichhane, Zhifang Xu, Jiaqi Wang, Vo Dai Quynh, Jing Huang, Feifei Gao, Meidan Zhao, Zelin Chen, Tianyi Zhao

**Affiliations:** 1 Research Center of Experimental Acupuncture Science, Tianjin University of Traditional Chinese Medicine, Tianjin, China; 2 School of Acupuncture and Moxibustion and Tuina, Tianjin University of Traditional Chinese Medicine, Tianjin, China; 3 School of Medical Technology, Tianjin University of Traditional Chinese Medicine, Tianjin, China; 4 School of Traditional Chinese Medicine, Tianjin University of Traditional Chinese Medicine, Tianjin, China; University of Maryland at College Park, UNITED STATES OF AMERICA

## Abstract

**Background:**

Acupuncture has been used to improve the quality of life (QoL) of patients in clinical settings. However, the effect of acupuncture on QoL in patients with irritable bowel syndrome (IBS) remains unclear. We conducted a systematic review and meta-analysis of randomized controlled trials (RCTs) to evaluate the effect of acupuncture on the QoL of patients with IBS.

**Method:**

PubMed, Cochrane Central Register of Controlled Trials, Embase, and Web of Science were screened from inception to September 2023. RCTs published in English comparing acupuncture with sham acupuncture, usual care, pharmacological interventions, or other therapies were analyzed for QoL in patients with IBS. The primary outcome was QoL and secondary outcomes were the IBS-symptom severity scale (IBS-SSS) and abdominal pain. The Cochrane Collaboration recommendations were used to assess the risk of bias.

**Findings:**

Fourteen articles with 2,038 participants were included. The pooled result showed acupuncture can significantly improve the QoL of patients with IBS compared to the conventional treatment (MD = 6.62, 95% CI, 2.30 to 10.94, *P<0*.*001*, *I*^*2*^ = 72.45%). Additionally, acupuncture was superior to other interventions in relieving the symptoms’ severity of IBS (MD = -46.58, 95% CI, -91.49 to -1.68, *P*<0.001, *I*^*2*^ = 90.76%). Nevertheless, acupuncture was not associated with abdominal pain reduction (MD = -0.35, 95% CI, -0.91 to 0.20, *P* = 0.21, *I*^*2*^ = 0.00%). Lower adverse events were observed in the acupuncture group. Thus, the quality of this study was relatively high.

**Conclusion:**

The meta-analysis showed that acupuncture improves QoL and symptom severity in patients with IBS and that the optimal parameters for acupuncture to improve QoL in patients with IBS are 30 minutes of acupuncture per session, less than or equal to five sessions per week, and a 4-week course of treatment. However, more high-quality clinical trials are needed to provide stronger evidence.

## 1. Introduction

Irritable Bowel Syndrome (IBS) is a functional bowel disorder characterized by frequent abdominal pain and diarrhea [1]. It is categorized into four subtypes: IBS with constipation (IBS-C), diarrheal IBS (IBS-D), mixed IBS (IBS-M), and unspecified IBS (IBS-U) [2]. The global prevalence of IBS ranges from 3% to 15% in different regions of the world, with a gradually increasing prevalence [3, 4]. IBS represents 30% of all gastrointestinal disorders in primary care [5]. The chronic and recurrent nature of IBS could ultimately lead to the need for frequent medical consultations, aggravate depression and anxiety disorders, increase work absenteeism, reduce productivity, and increase hospitalizations, all of which have a considerable impact on quality of life (QoL) and elevate the economic burden on patient’s family [6, 7]. A cohort study in the Netherlands found a quarterly total direct health cost of $909 and an indirect cost of $1,535 [8].

Over the past 20 years, a number of medications have been developed to treat IBS, such as rifaximin, eluxadoline, and alosetron, but these drugs often produce adverse reactions such as nausea and constipation, resulting in the need for additional interventions and increasing the financial burden on patients [9, 10]. Since there are no specific biological disease markers, quality of life assessment is a crucial factor in determining the degree of recovery [11]. Various therapeutic modalities, including dietary modifications and psychological interventions, aim to ease IBS symptoms and improve QoL. However, uncertainties exist in understanding specific dietary triggers, such as Fermentable Oligosaccharides, Disaccharides, Monosaccharides, and Polyols (FODMAPs), lactose, or gluten, and the effectiveness of treatments that target gut bacteria, such as probiotics, has not been consistently confirmed. Consequently, there is a need for effective options for patients and healthcare providers to improve their QoL.

Acupuncture, an ancient traditional Chinese medicine (TCM) therapy, is reported as a significant intervention for addressing functional gastrointestinal disorders by stimulating specific acupoints [12, 13]. Previous systematic reviews and meta-analyses have also reported that acupuncture is an effective and safe intervention for IBS, alleviating symptoms such as abdominal pain, symptom severity, anxiety, and depression [14–16]. We previously summarized the central and peripheral analgesic mechanisms of acupuncture in the treatment of IBS. At the peripheral level, acupuncture can alleviate inflammation and pain by reducing 5-hydroxytryptamine and its receptors in intestinal sensory endings, and by blocking the activity of intestinal glial cells to reduce pain-related neurotransmitters, thereby weakening peripheral sensitization. Acupuncture can inhibit the activity of N-methyl-D-aspartate receptor ion channels in the spinal cord and opioids in the cingulate cortex of the central nervous system. Furthermore, acupuncture can regulate IBS depression by targeting hypothalamic-pituitary-adrenal axis-related hormones and neurotransmitters via the relevant brain nuclei, thereby improving the IBS-induced VH response [17].

The clinical studies have also shown acupuncture potentially increases the QoL [18, 19]. Particularly, during the COVID-19 pandemic, improving the QoL of patients with IBS has attracted increasing attention. Notably, there was an observed increase in IBS incidence and worsening of QoL, and acupuncture was effective for the management of IBS [20, 21]. Additionally, its integration with other therapies offers holistic benefits, especially for patients with IBS dealing with anxiety and depression, which can affect their daily lives [22, 23]. The therapeutic effect of acupuncture is closely related to parameters such as treatment frequency, duration, and course of treatment [24]. For instance, 30 minutes, 3 times/week for 4 weeks was beneficial to composite response rates [25], 30 minutes, 5 times/week for 4 weeks is favorable for symptom relief [26], and 30 minutes, 3 times/week for 6 weeks helps to improve symptoms and QoL [27] in people with IBS. Although studies have found that acupuncture improves the QoL in patients with IBS [28, 29], no study has summarized the optimal parameters and methods by which acupuncture improves QoL. Thus, analyzing the acupuncture parameters and methods can offer valuable insights to clinicians [30]. Therefore, this meta-analysis aimed to investigate the efficacy and safety of acupuncture on the QoL of patients with IBS.

## 2. Materials and methods

### 2.1 Protocol and registration

Our study was conducted based on the checklist of the preferred reporting items for systematic reviews and meta-analyses (PRISMA) guidelines 2020 [31] (S1 File). This study was registered with PROSPERO (registration number: CRD42023479766).

### 2.2 Literature search strategy

Two authors (ZJ and LN) independently searched for studies published in PubMed, the Cochrane Central Register of Controlled Trials, Embase, and Web of Science from inception to September 2023. The search was limited to articles published in English using the search terms “acupuncture” and “irritable bowel syndrome” For PubMed, the following search strategy was used: (“Acupuncture therapy” MeSH OR “Acupuncture” Title/Abstract OR “transcutaneous electric stimulation” MeSH OR “Auricular acupuncture” MeSH) AND (“Irritable bowel syndrome” MeSH OR “Irritable bowel syndrome” Title/Abstract OR “Irritable Colon” Title/Abstract) AND (“clinical trials” OR “RCT” OR “Randomized”). The search strategy for each database is provided in the S2 File. Additionally, relevant reviews were manually examined to identify gaps in the included articles and to prevent omissions.

### 2.3 Eligibility criteria

#### 2.3.1 Study types

Randomized controlled trials (RCTs) of acupuncture for the treatment of IBS.

#### 2.3.2 Participants

Patients were diagnosed with IBS according to Rome I–IV [32], expert consensus [33] or by a clinical physician. There were no restrictions on patient age or sex.

#### 2.3.3 Interventions

Interventions performed with acupuncture, including manual acupuncture (ACU), electroacupuncture (EA), and transcutaneous electrical stimulation were included.

#### 2.3.4 Comparisons

Participants who were treated with placebo acupuncture, medicine, or usual care were included. There were no restrictions on the dosage, intake route, or duration of treatment.

#### 2.3.5 Outcomes measures

The primary outcome was the QoL of patients with IBS. Among them, IBS Quality of Life (IBS-QOL) was mostly used to assess the QoL of patients with IBS. It is a quality-of-life measurement designed for IBS, utilizing a model based on qualitative interviews with Rome criteria-diagnosed patients, consisting of 34 entries across eight dimensions, reflecting improved health-related QoL [34]. Other QoL scales were also used. The IBS-36 is a comprehensive and detailed assessment tool containing 36 entries, which includes not only an assessment of the patient’s QoL but also covers several dimensions such as symptom severity, mental health, physical functioning, and illness perception and coping [35]. The SF-36 is a validated global measure of health-related QoL that is not specific to a particular disease and has been widely used for a variety of conditions. It uses 36 items to assess eight scales (physical functioning, physical role, physical pain, general health, vitality, social functioning, emotional role, and physical well-being) [36]. The FDDQL is specifically designed to assess the impact of functional digestive disorders on QoL [37]. The secondary outcomes were IBS symptom severity scale (IBS-SSS) [38] and abdominal pain. Adverse events and the safety of acupuncture were also analyzed.

### 2.4 Exclusion criteria

(1) Observational studies, review studies, dissertations, animal studies, data mining studies, and meta-analyses were excluded; (2) studies involving interventions such as moxibustion, acupressure, auricular acupuncture, laser acupuncture, cupping, and acupoint injections were excluded based on research aims, insufficient research evidence for IBS and the potential to reduce study heterogeneity after the discussion; (3) duplicate publications were excluded; and (4) studies with incomplete outcomes or those that could not be retrieved from the literature were also excluded. See S1 and S2 Tables for numbered tables of all studies.

### 2.5 Study selection and data extraction

Two researchers (ZJ and LN) conducted independent literature screening and data extraction and cross-verified their findings. In case of disagreement, a third researcher was consulted for adjudication. The following information was extracted for data analysis: (1) general information, including the name of the first author, publication year, sample size, and diagnostic criteria; (2) baseline data encompassing age, sex, course of disease, and outcome indicators for each group before treatment; (3) intervention details, such as the type, frequency, and duration of acupuncture administered; and (4) post-treatment data, with inter-group differences converted using baseline disparities and final values. In the case of missing data, we tried to contact the original authors through phone or email and when data was not available, we excluded the article mentioning the clear reason for exclusion. See S3 Table for all data extracted.

### 2.6 Quality assessment of risk of bias

The risk of bias in the included studies was assessed using the Cochrane Collaboration’s Risk of Bias tool recommended by the Cochrane Handbook 5.1, which evaluates studies based on criteria, such as the method of random sequence generation, allocation concealment, blinding of participants and personnel, blinding of outcome assessment, handling of incomplete outcome data, selective reporting of outcomes, and consideration of other potential biases [39]. See S4 Table for Cochrance Quality Assessment Scale.

### 2.7 Statistical analysis

Statistical analyses were performed using Stata 17.0 software. The mean difference (MD) was used for the outcomes assessed on the same scale. A fixed-effects model was employed for the meta-analysis when the *I*^*2*^ value was less than 50% and the *P*-value was greater than 0.05. Conversely, a random-effects model was used when the *I*^*2*^ value exceeded 50% and the source of heterogeneity was subsequently analyzed. Forest plots were used for the visual representation of the results, and a statistical test with a *P* value less than 0.05 was considered statistically significant. Subgroup analyses were performed based on different dimensions of QoL, intervention used, frequency of acupuncture, needle retention time, and duration of treatment. Publication bias was assessed subjectively by observing the symmetry of the funnel plots, and objectively using Egger’s test. No publication bias was inferred if the funnel plots were symmetrical. The Egger’s test was used to objectively assess publication bias. A p-value greater than 0.05 indicated that the funnel plots were likely symmetrical. In addition, a descriptive analysis was performed in the absence of suitable data.

## 3. Results

### 3.1 Identification of studies

**Fig 1:**
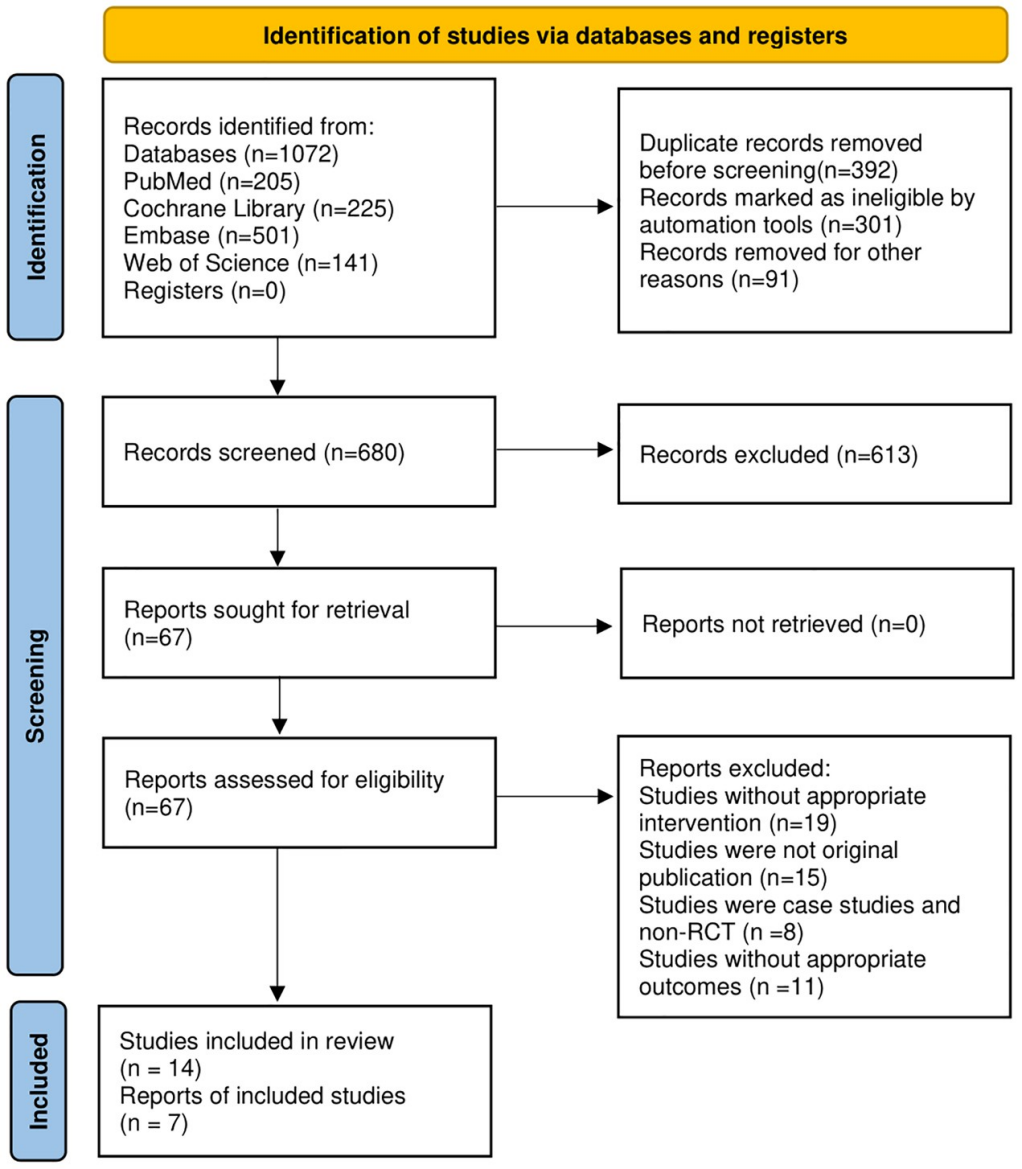
Study selection process according to PRISMA. RCT: Randomized clinical trial.

Initially, 1,072 studies were identified. After eliminating 392 duplicate articles, the titles and abstracts of 680 articles were evaluated. Among these, 293 articles were reviews, meta-analyses, and study protocols, whereas 268 studies unrelated to IBS and acupuncture. and 52 articles published in languages other than English were excluded. Subsequently, the remaining 67 full-text articles were screened. After excluding 53 articles that did not align with appropriate intervention methods and outcomes, 14 RCTs [19, 25, 27, 40–50] were considered suitable for this meta-analysis. Fig 1 shows the results of the literature screening process.

### 3.2 Characteristics of included studies

Among the 14 studies, 2,038 participants were included: seven studies from China [19, 25, 27, 42, 44, 47, 50], two from the UK [41, 46], two from Germany [48, 49], one from the USA [43], one from Turkey [40] and one from Canada [45], with sample sizes ranging from 27 to 519. All trials were published in English between 2005 and 2022. Six RCTs [19, 25, 42, 44, 47, 50] described the type of IBS as IBS-D, two studies [27, 40] mentioned IBS-C/D, and the remaining articles did not describe it. IBS was diagnosed using the Rome criteria: two articles mentioned Rome IV [25, 42], six articles mentioned Rome III [19, 27, 40, 44, 47, 50], four articles mentioned Rome II [41, 43, 48, 49], one article mentioned Rome I [45], and one article mentioned that the diagnosis was performed by a clinical physician [46].

### 3.3 Characteristics of intervention

The basic interventions included ACU, EA, and TES, while the comparators included sham acupuncture, sham TES, and medicine. Among the 14 articles, four compared acupuncture with Western medicine [19, 27, 44, 50], seven compared acupuncture with sham acupuncture [25, 41, 43, 45, 47–49], and two compared TES with sham TES [40, 42]. Similarly, one study compared acupuncture adjuncts to routine treatment with routine treatment alone [46]. The baseline features and intervention methods used in this study are listed in Table 1.

**Table 1:** Basic characteristics of included studies.

Study	Country	Sample size (T/C)	Female population (T/C)	Mean Age (T/C)	Duration of treatment	Type of IBS and Diagnosis Criteria	Intervention	Comparator	Outcomes
Hu et al., 2022 [42]	China	21/16	13/6	44.40±13.40/45.40±11.70	4 weeks	IBS-D, Rome IV	TEA	Sham-TEA	IBS-QOL, IBS-SSS, VAS
Qi et al., 2022 [25]	China	30/30/30	11/10/20	36.70±12.20/31.00 ±9.90/35.70±11.10	4 weeks	IBS-D, Rome IV	Acupuncture	Sham Acupuncture	IBS-QOL, IBS-SSS
Pei et al., 2020 [27]	China	344/175	165/87	45.89±13.01/47.00±12.73s	6 weeks	IBS-C/D, Rome III	Acupuncture	Western medicine	IBS-QOL, IBS-SSS
Arthur Dun-Ping Mak et al., 2019 [47]	Hongkong, China	40/40	20/22	50.85±11.57/50.83±14.15	10 weeks	IBS-D, Rome III	Electroacupuncture	Sham electroacupuncture	EQ-5D, 5-point Likert scale
C. Lowe et al., 2017 [45]	Canada	43/36	36/26	42.00±15.00/43.00±15.00	4 weeks	not stated, Rome I	Acupuncture	Sham Acupuncture	SF-36, IBS-36, IBS symptom assessment
Zheng et al., 2016 [19]	China	113/111/112/112	69/60/59/65	38.60±16.70/41.20±16.50/41.70±17.50/40.60±16.70	4 weeks	IBS-D, Rome III	Electroacupuncture	Western medicine	SF-36
Li et al., 2013 [44]	China	35/35	20/17	39.10±11.80/37.90±11.50	4 weeks	IBS-D, Rome III	Acupuncture	Western medicine	IBS-QOL, IBS symptom assessment
Şahin Çoban et al., 2012 [40]	Turkey	29/29	24/19	43.10±11.10/40.50±9.10	4 weeks	IBS-C/D, Rome III	IFC	Sham IFC	IBS-QOL, VAS
Hugh MacPherson et al., 2012 [46]	UK	116/117	95/93	44.28 ±14.31/42.68±14.79	10 weeks	Both not stated	Acupuncture+ Routine treatment	Routine treatment	EQ-5D, SF-12 Quality of Life IBS-SSS
Sun et al., 2011 [50]	China	30/30	NR	42.75±10.22/39.53±8.91	4 weeks	IBS-D, Rome III	Acupuncture	Western medicine	IBS-QOL, IBS-SSS
Anthony J. Lembo et al., 2009 [43]	USA	78/75/77	78/77/74	37.50±14.60/38.90±14.10/39.00±14.00	6 weeks	not stated, Rome II	Acupuncture	Sham Acupuncture	IBS-QOL, IBS-SSS
Schneider et al., 2007 [49]	Germany	18/14	15/13	46.23±15.00/41.80±14.51	5 weeks	not stated, Rome II	Acupuncture	Sham Acupuncture	SF-36, FD-QOL
Schneider et al., 2006 [48]	Germany	22/21	17/17	47.63±14.71/47.14±16.01	5 weeks	not stated, Rome II	Acupuncture	Sham Acupuncture	SF-36, FD-QOL
Forbes et al., 2005 [41]	UK	27/32	16/23	43.00/44.40	13 weeks	not stated, Rome II	Acupuncture	Sham Acupuncture	EuroQol, IBS symptom assessment

IFC, interferential current; IBS-SSS, IBS Symptom Severity Scale; IBS-QOL: IBS Quality of Life Scale; TEA: Transcutaneous electrical stimulation; VAS, visual analog scale

### 3.4 Risk of bias assessment

**Fig 2:**
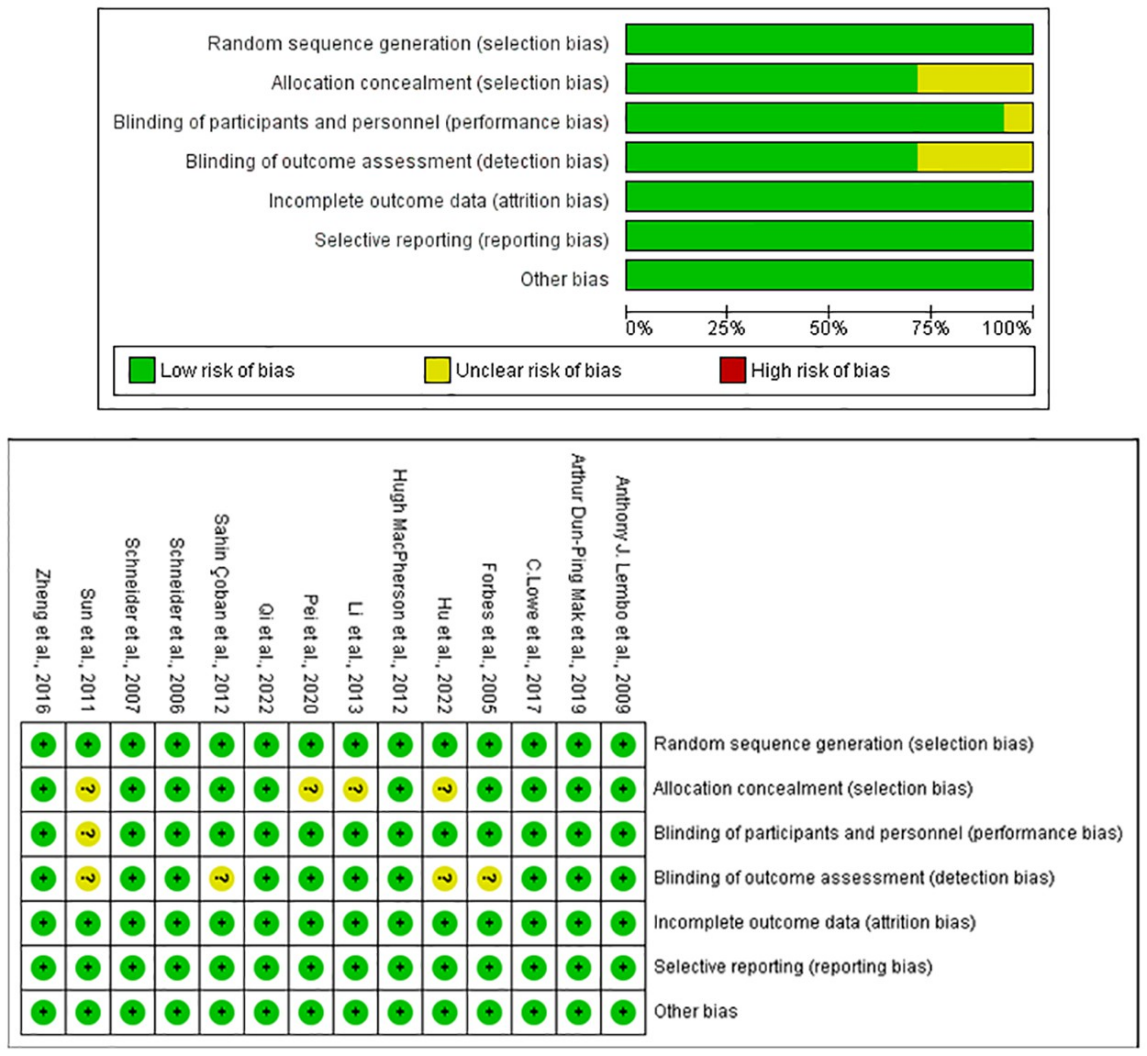
Risk of bias assessment graph of randomized clinical trials and risk of bias summary of randomized clinical trials.

Two researchers (ZJ and LN) independently evaluated the risk of bias in the included studies. All the included articles reported data without selective reporting or other biases. One article did not mention the blinding of participants, which resulted in an unclear risk [50]. Additionally, four studies [40–42, 50] did not mention blinding of the assessors in the study and were assessed as uncertain. Four studies [27, 42, 44, 50] were rated as "risk unclear" because the studies did not mention allocation concealment. A summary of the Risk of Bias is shown in Fig 2.

### 3.5 Primary outcome

#### 3.5.1 QoL

**Fig 3:**
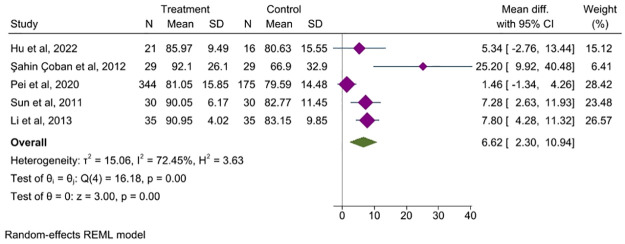
Forest plot comparing the QoL between acupuncture group and control group. CI: Confidence Interval, N: Number, SD: Standard deviation.

In this study, 14 RCTs assessed the QoL before and after treatment. Seven studies [25, 27, 40, 42–44, 50] with 987 patients used the IBS-QOL scale. An analysis of five studies showed that acupuncture significantly improved the QoL of patients with IBS compared with conventional therapy (MD = 6.62, 95% CI, 2.30 to 10.94, *P<*0.001, *I*^2^ = 72.45%), Fig 3. However, two studies [25, 43] assessing efficacy by response rate showed no statistical difference between acupuncture and sham stimulation in improving QoL, whereas one study [45] used the IBS-36 to evaluate the QoL of IBS participants between the acupuncture and sham groups. There was a numerical but not significant difference for QoL (MD = -0.8, 95% CI, -0.3 to 0.2, *P* = 0.28) between those receiving acupuncture versus sham acupuncture. In addition, four studies reported the SF36. Among them, one study [49] reported the total SF-36 score and showed that acupuncture did not improve QoL in patients with IBS compared to sham acupuncture. A study [19] evaluated physical functioning, mental health, social functioning, and other dimensions of the SF-36, and the results showed that there was no statistical difference in any of the dimensions between EA and Western medicine. One study [48] showed that acupuncture can significantly improve the pain scale compared to sham stimulation, whereas the other studies showed no statistical differences. Another study [45] showed that patients in acupuncture and sham stimulation groups had increased mental and physical health scores on the SF-36 scale, but this was not statistically significant. Additionally, two studies compared the FDDQL between acupuncture and sham acupuncture to assess QoL in patients with IBS. One study [48] reported both total score and subscale data and showed a significant improvement in the FDDQL total score in both groups without significant differences between the two groups. The subscales of the FDDQL also showed significant improvements in both groups after treatment, such as the daily activity index, which improved by approximately 7% and 16% (acupuncture and sham, respectively), diet (23% and 16%), discomfort (14% and 6%), and disease coping (21% and 3%); however, there was no significant difference between the two groups. Another study [49] reported only the total score showed improvements in QoL in both groups; however, there were no significant differences between the groups.

### 3.6 Secondary outcome

#### 3.6.1 IBS-SSS

**Fig 4:**
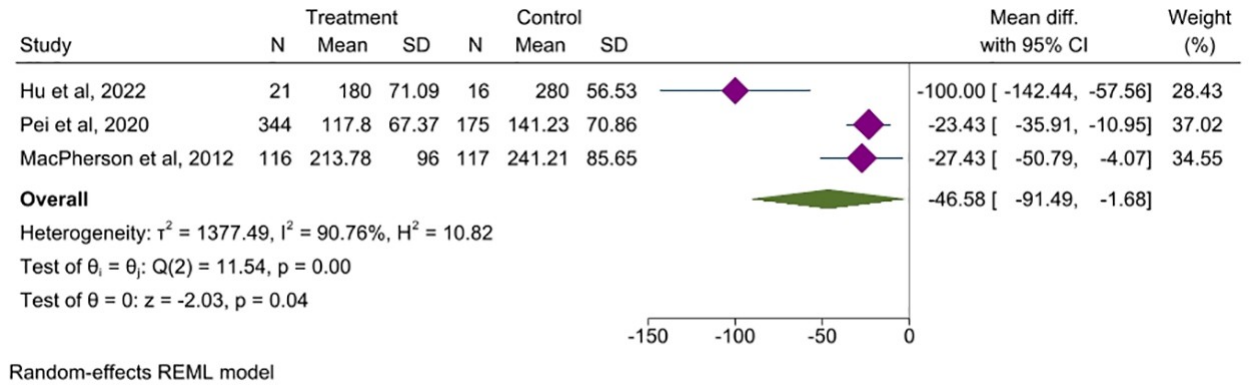
Forest plot comparing the symptoms severity between acupuncture group and control group. CI: Confidence Interval, N: Number, SD: Standard deviation.

Nine articles assessed the total symptom scores after treatment. Six studies [25, 27, 42, 43, 46, 50] compared the IBS-SSS between the acupuncture and control groups to assess symptom severity. Among them, three studies [27, 42, 46] with 789 patients incorporated the total symptom scores that could be analyzed. The analyzed results suggest that acupuncture can significantly reduce the severity of symptoms in patients with IBS compared to the control group (MD = -46.58, 95% CI, -91.49, -1.68, *P* = 0.00, *I*^*2*^ = 90.76%) Fig 4. Additionally, three studies [41, 44, 45] evaluated symptom severity scores using their own designed questionnaires. As there was variation in the questionnaire and assessment scales, we could not statistically analyze it.

#### 3.6.2 Abdominal pain

**Fig 5:**
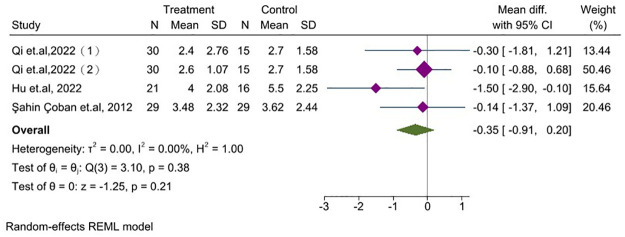
Forest plot comparing the abdominal pain between acupuncture group and control group. CI: Confidence Interval, N: Number, SD: Standard deviation.

Three studies [25, 40, 42] involving 155 patients were conducted to assess abdominal pain intensity using the VAS in individuals with IBS. It indicated that acupuncture did not show a substantial reduction in abdominal pain symptoms compared to sham acupuncture and Western medicine in patients with IBS (MD = -0.35, 95% CI, -0.91 to 0.20, *P* = 0.21, *I*^*2*^ = 0.00%), Fig 5.

### 3.7 Subgroup analysis

#### 3.7.1 Different subclass of IBS-QOL

**Fig 6:**
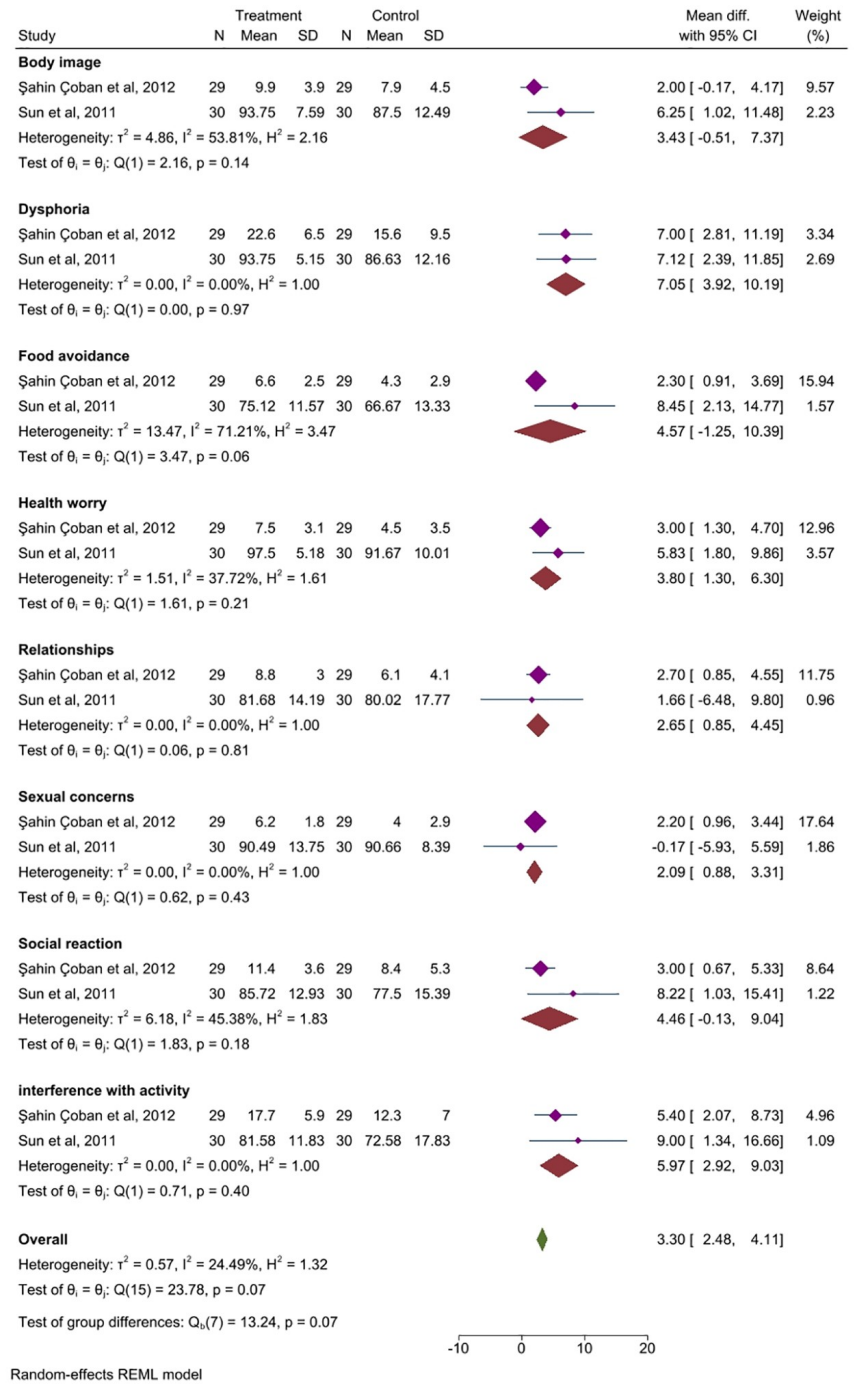
Forest plot of subgroup analysis comparing the different subclass of IBS-QOL between acupuncture group and control group. CI: Confidence Interval, N: Number, SD: Standard deviation.

Acupuncture compared with the control group showed advantages in the following areas: somatic complaints (MD = 7.05, 95% CI, 3.92 to 10.19, *P =* 0.97, *I*^*2*^ = 0.00%), health concerns (MD = 3.80, 95% CI, 1. 30 to 6.30, *P =* 0.21, *I*^*2*^ = 37.72), interpersonal relationships (MD = 2.65, 95% CI: 0. 85 to 4.45, *P =* 0.81, *I*^*2*^ = 0.00%), sexual problems (MD = 2.09, 95% CI, 0.88 to 3.31, *P =* 0.43, *I*^*2*^ = 0.00%), and daily activities interference (MD = 5.97, 95% CI, 2.92 to 9.03, *P =* 0.40, *I*^2^ = 0.00%). The remaining aspects did not show statistical differences, Fig 6.

#### 3.7.2 Different control

**Fig 7:**
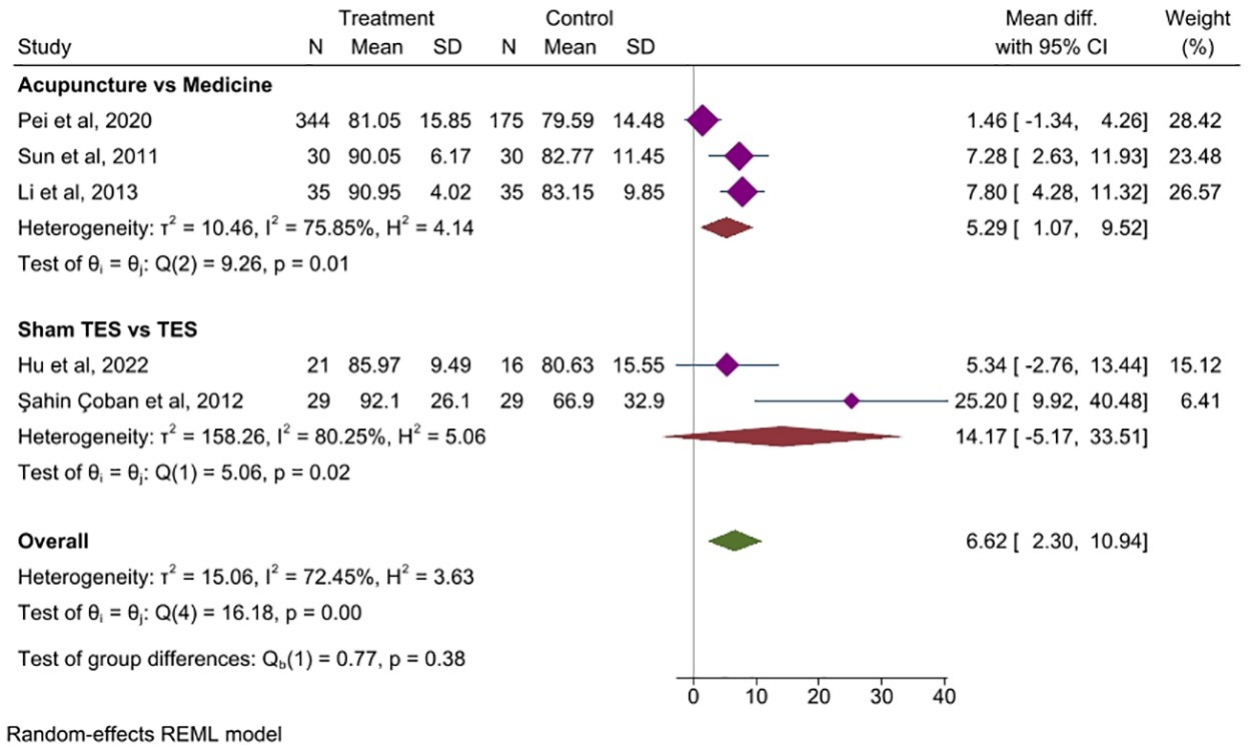
Forest plot of subgroup analysis comparing the impact of different intervention in IBS-QOL between treatment group and control group. CI, Confidence Interval, N: Number, SD: Standard deviation, TES: Transcutaneous electric stimulation.

*Acupuncture vs medicine*. Three studies [27, 44, 50] compared IBS-QOL scores between the acupuncture and medicine groups. It showed that acupuncture is superior to medication in improving the QoL for patients with IBS (MD = 5.29, 95% CI, 1.07 to 9.52, *P* = 0.01), Fig 7.

*TES vs sham TES*. Two studies [40, 42] compared IBS-QOL scores between the TES and sham TES groups. No significant differences were found between the two groups. These results indicate that TES is not inferior to sham treatment in improving the QoL of patients with IBS. (MD = 14.17, 95% CI, -5.17 to 33.51, *P =* 0.02), Fig 7.

#### 3.7.3 Acupuncture parameters

**Fig 8:**
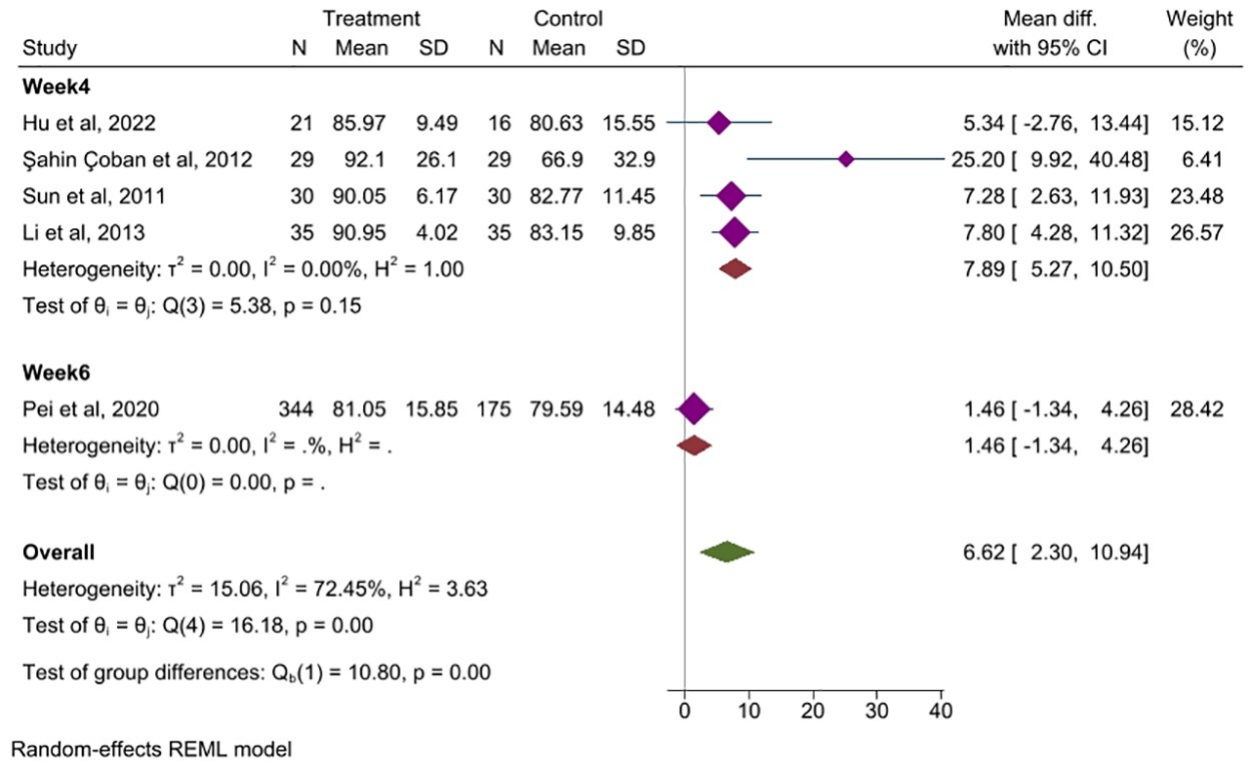
Forest plot of subgroup analysis comparing the duration of treatment between acupuncture group and control group. CI: Confidence Interval, N: Number, SD: Standard deviation.

*Duration of treatment*. Five studies [27, 40, 42, 44, 50] comparing the effect of different intervention durations on the QoL of patients with IBS. It showed an improvement in QoL at 4 weeks of treatment (MD = 7.89, 95% CI, 5.27 to 10.50, *P<*0.001) while no significant improvement in QoL was found at 6 weeks of treatment (MD = 1.46, 95% CI, -1.34 to 4.26, *P<*0.001), Fig 8.

**Fig 9:**
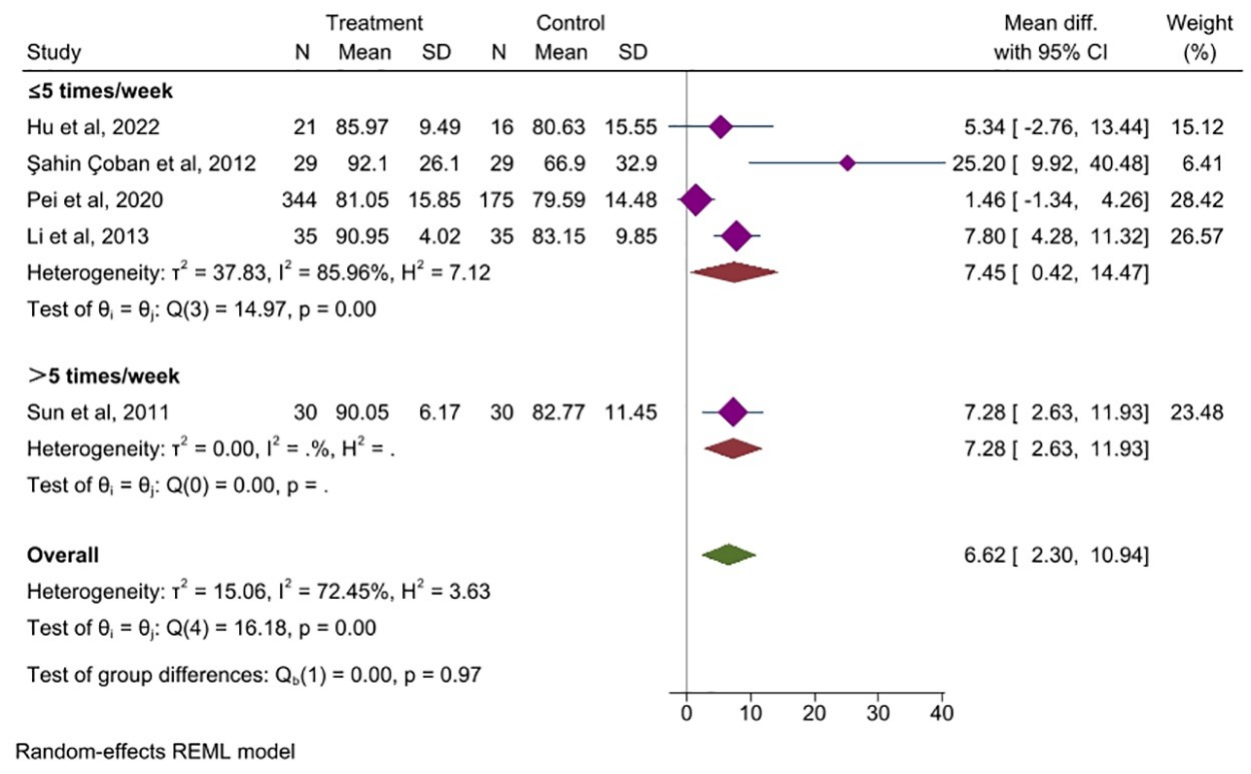
Forest plot of subgroup analysis comparing the frequency of treatment between acupuncture group and control group. CI: Confidence Interval, N: Number, SD: Standard deviation.

*Frequency of treatment*. Five studies [27, 40, 42, 44, 50] compared the effect of frequency of acupuncture interventions on QoL in patients with IBS. It showed that both less than five acupuncture sessions a week and more than five acupuncture sessions a week were beneficial in improving the QoL for people with IBS (MD = 7.45, 95% CI, 0.42 to 14.47, *P =* 0.038 and MD = 7.28, 95% CI, 2.63 to 11.93, *P =* 0.002, respectively), Fig 9.

**Fig 10:**
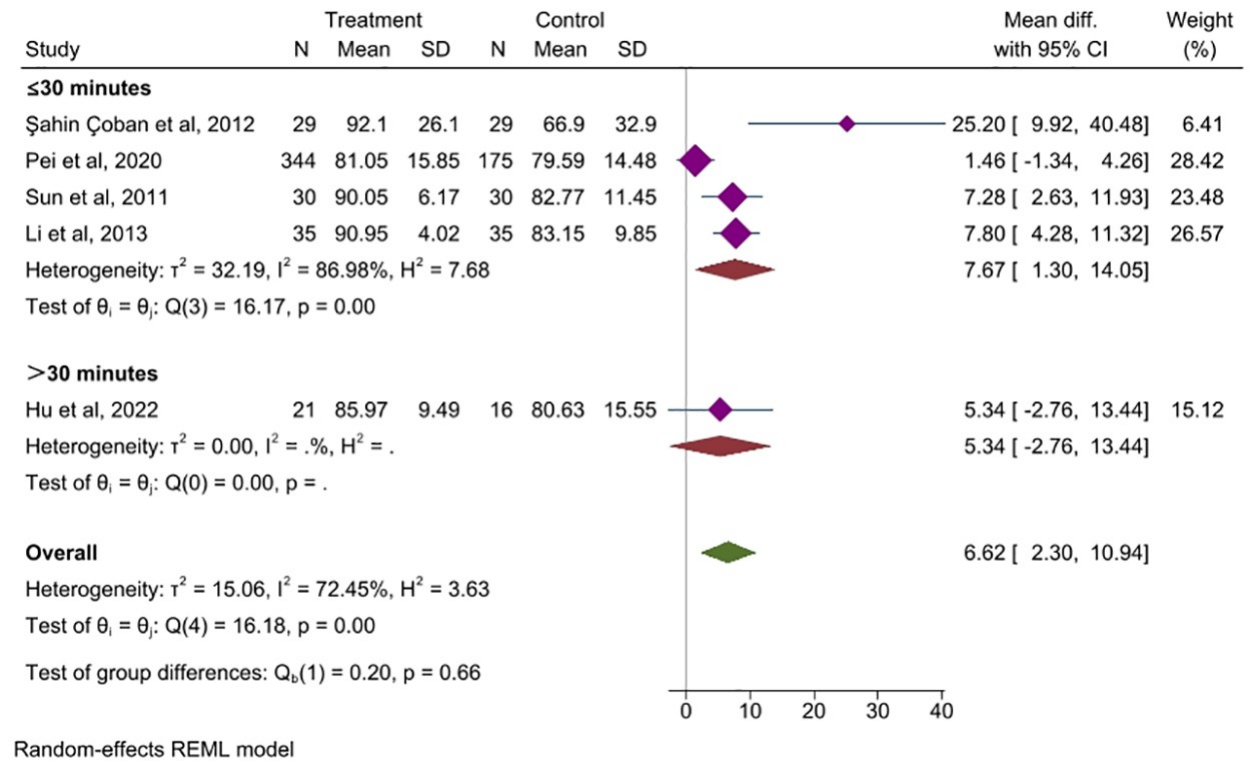
Forest plot of subgroup analysis comparing the needle retention time between the acupuncture group and control group. CI: Confidence Interval, N: Number, SD: Standard deviation.

*Needle retention duration*. Five studies [27, 40, 42, 44, 50] compared the effect of the needle retention time on QoL in IBS, and acupuncture sessions lasting more than 30 minutes per day did not show a significant effect (MD = 5.34, 95% CI, -2.76 to 13.44, *P =* 0.196), while sessions having less than 30 minutes demonstrated a significant effect (MD = 7.67, 95% CI, 1.30 to 14.05, *P<0*.*001*, *I*^*2*^ = 86.98%), Fig 10.

### 3.8 Publication bias

**Fig 11:**
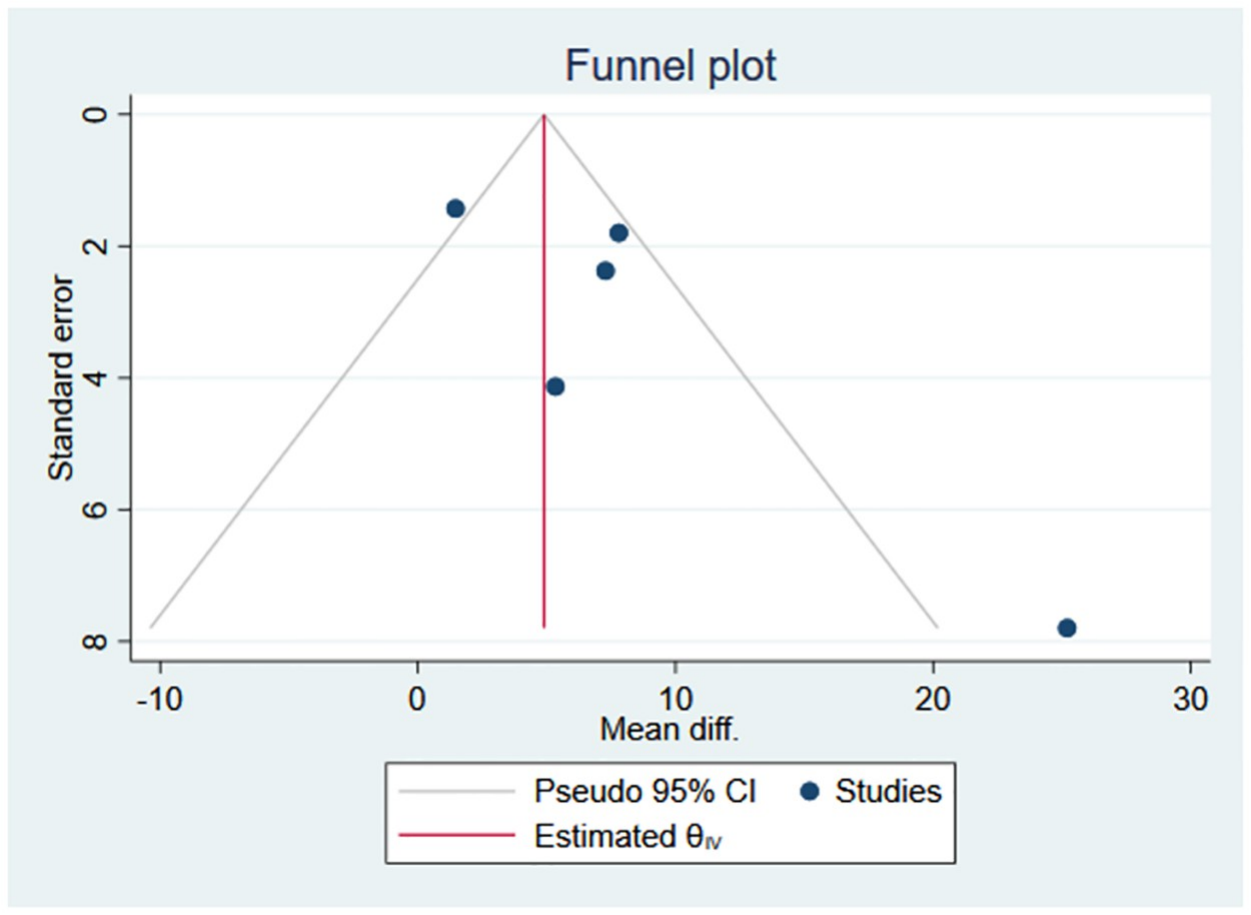
Funnel plot for publication bias. CI: Confidence Interval, SND: Standard normal distribution.

**Fig 12:**
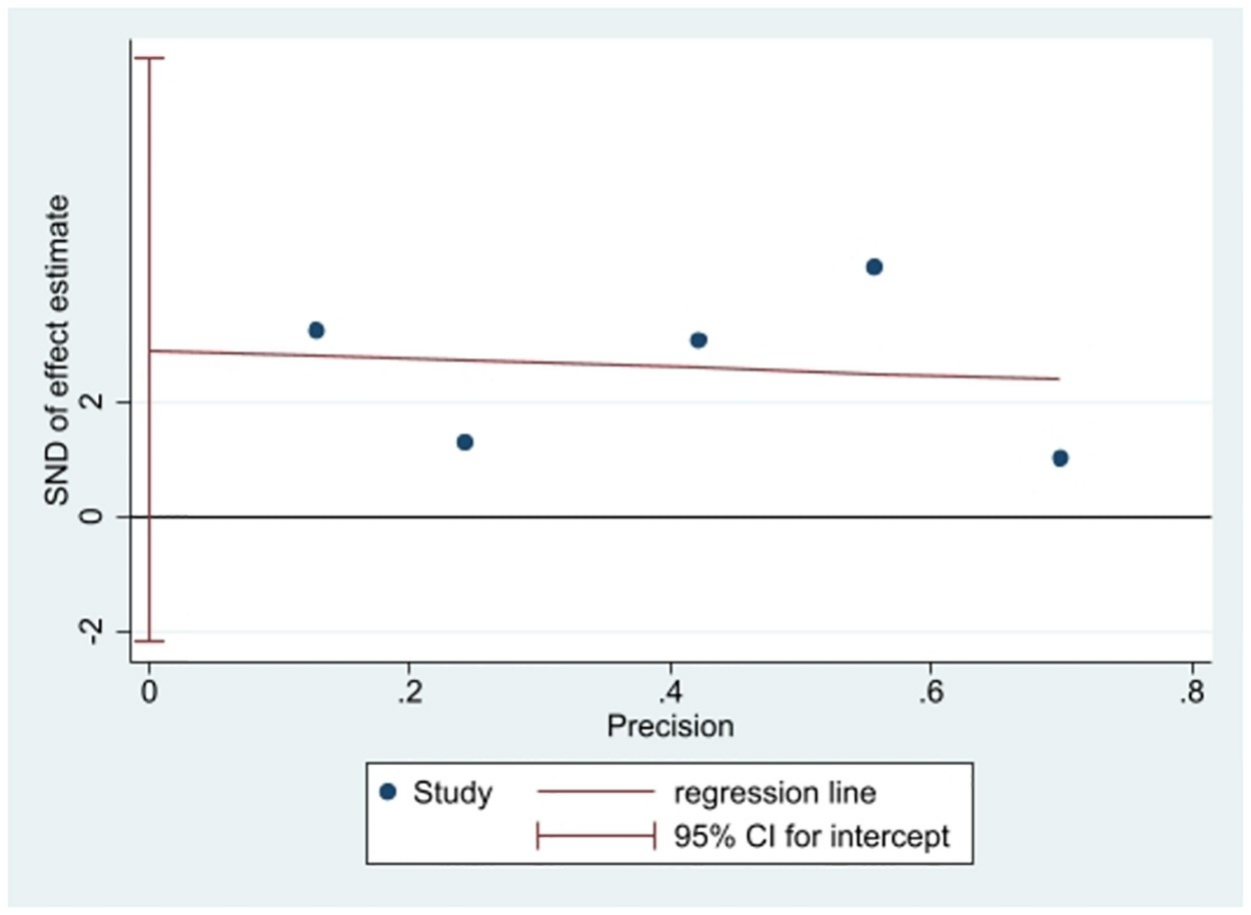
Egger test graph for publication bias. CI: Confidence Interval, SND: Standard normal distribution.

The potential for publication bias was assessed using the funnel plot shown in Fig 11. Visual inspection of the funnel plot did not reveal any evidence of asymmetry. The Egger test did not detect any potential publication bias (*P =* 0.11 > 0.05) Fig 12.

### 3.9 Adverse events

Adverse events were reported in three studies [19, 27, 46] such as subcutaneous hematoma, abdominal pain, cold limbs, and nerve pain. However, these events did not affect the efficacy of acupuncture, and all adverse effects were transient, with no serious adverse effects. The remaining 11 studies did not report adverse events.

## 4. Discussion

This review systematically examined the efficacy of acupuncture in improving the QoL of patients with IBS. Finally, 14 studies involving 2,038 patients with IBS were evaluated. The results showed that acupuncture is beneficial in relieving symptom severity and improving QoL in patients with IBS compared to conventional therapy. Furthermore, from the analysis of the eight sub-classes of the IBS-QOL, acupuncture was found to be crucial for improving mood, reducing health concerns, and improving relationships and daily life in patients with IBS. In addition, subgroup analyses showed that less than 30 min of needle retention, less than five times per week for 4 weeks, may be preferred to improve QoL in patients with IBS, and it is hoped that this review will inform the clinical decisions of acupuncturists. Only three trials reported adverse events, including subcutaneous hematoma in two studies and other adverse events such as abdominal pain, cold limbs, and nerve pain in one trial, and the adverse events were mild, which did not affect the treatment effect.

### 4.1 Principal findings and comparison with other studies

Several studies have analyzed the effects of acupuncture on QoL and IBS symptoms. Guo et al. conducted a meta-analysis to investigate the effects of acupuncture on IBS in 2020 [29]. They included 31 RCTs with 3,234 patients from five electronic databases, and the IBS-QOL and IBS-SSS were used to evaluate QoL and IBS symptoms, respectively. They demonstrated that acupuncture had a significant effect on QoL improvement and symptom severity reduction compared to the control group after the elimination of the heterogeneous sources. Our findings are consistent with those of previous studies. Furthermore, we found significant QoL improvement with acupuncture compared with pharmaceutical drugs, according to the included studies [15, 28]. It has been reported that medicines mainly treat IBS by relieving smooth muscle spasms in the digestive tract and anticholinergic agents such as pinaverium bromide and otilonium bromide. Similarly, acupuncture has been shown a potential role in stimulating gastrointestinal movement, regulating intestinal flora, and offering symptomatic relief [51]. It may be related to anti-inflammation for acupuncture in patients with IBS, since acupuncture has been reported to reduce inflammation by inhibiting the transient receptor potential vanilloid-1 channel in peripheral intestinal sensory neurons [52], downregulating 5-hydroxytryptamine 3 receptor (5-HT3R) expression within the serotonin pathway, increasing 5-HT4R expression, influencing the activity of receptors and neurotransmitters in peripheral sensory endings [17, 53–55]. However, acupuncture is associated with fewer adverse events than other drugs [16]. Additionally, as a chronic disease, the QoL of patients with IBS is related to several factors in addition to symptoms. For instance, depression and anxiety have been associated with the QoL in patients with IBS. Acupuncture plays a significant role in improving emotions, health, daily life, and relationships. Acupuncture is beneficial for alleviating depression and anxiety by modulating nerve-related functions [56]. Therefore, the reasons mentioned above may also explain why acupuncture promotes QoL in patients with IBS.

We found that acupuncture did not significantly improve pain in patients with IBS. This finding is inconsistent with those of previous studies [28] and we speculate that there are several possible reasons for this result. First, different acupuncture treatment protocols and parameters were used in previous studies and in our research, which led to inconsistent results. Second, the primary outcomes of the clinical trials included in the studies varied owing to different research aims. For instance, some trials considered symptom or mood improvement of patients with IBS as the primary outcome indicators, which is an essential basis for sample size calculation. Therefore, it is possible that previous studies have failed to achieve the sample sizes to improve pain for individuals with IBS, especially for some patients with IBS for whom pain is not a major complaint. However, a previous study [28], reported a significant benefit of true acupuncture in pain relief in patients with IBS when compared to sham acupuncture with lower heterogeneity. This illustrates the potential efficacy of acupuncture in treating abdominal pain in patients with IBS. One research conducted by Zheng et al. (2019) found no significant differences in QoL between acupuncture and sham acupuncture [15], which is consistent with our study. However, different intervention methods and strategies, limited literature included in the meta-analysis, smaller sample sizes of each study, and the fact that QoL was not the primary outcome in the original research may have contributed to the ineffectiveness of true acupuncture. Therefore, additional high-quality studies are necessary to evaluate the effects of acupuncture on multiple aspects of patients with IBS, including symptoms, pain, and QoL.

### 4.2 Clinical implications

This study conducted a meta-analysis that investigated the effect of acupuncture on the IBS treatment. Additionally, we evaluated the effects of different acupuncture parameters and methods on the QoL of patients with IBS using a subgroup analysis. This study demonstrated the benefits of acupuncture in alleviating symptom severity and improving QoL.

As a complementary therapy for IBS management, acupuncture has been found to be well tolerated with few adverse reactions; therefore, it is universally used in clinics [16]. Multiple studies have also shown the benefits of acupuncture in improving IBS symptoms and QoL [27, 43, 57]. Furthermore, we found that acupuncture with 30 minutes per session, less than or equal to 5 sessions per week, lasting for a 4-week course of treatment may be the optimal parameter in improving QoL for patients with IBS. Owing to the lack of conclusive findings in previous studies, we aimed to determine the ideal acupuncture parameters. This finding could provide significant evidence for patients with IBS when considering acupuncture treatment, acupuncturists when performing acupuncture treatment, and decision-makers when considering the development of policies and financial support for gastrointestinal diseases related to IBS. Additionally, patients with IBS may experience a variety of complaints such as abdominal discomfort, changes in bowel habits and patterns, health concerns, and a decrease in QoL. Therefore, it is reasonable to assess the overall effects on symptoms and QoL in patients with IBS.

### 4.3 Limitations

Our study had certain limitations. First, the studies included in this review mainly evaluated short-term efficacy, and follow-up data after acupuncture were lacking. Additionally, the sample sizes of most studies were small. Second, most included trials were conducted in China. Epidemiological data indicate a broad range of IBS prevalence worldwide, emphasizing the necessity of conducting additional cross-regional studies [7]. Furthermore, most outcomes were subjective assessments. In the future, more high-quality clinical studies are needed to clarify the efficacy of acupuncture on the QoL of patients with IBS.

## 5. Conclusion

The findings of our study suggest that acupuncture may be a feasible and safe intervention for improving the QoL in patients with IBS. However, further research with a larger sample size, long-term efficacy, and higher-quality study design is warranted.

## Supporting information

S1 Table List of articles identified from different databases. (DOCX)

S2 Table List of included and excluded studies. (DOCX)

S3 Table All data extracted. (XLSX)

S4 Table Cochrance quality assessment scale. (XLSX)

S1 File PRISMA 2020 checklist. (DOCX)

S2 File Search strategy used in different databases. (DOCX)
