# Supplementary material for: The effect of acupuncture on quality of life in patients with irritable bowel syndrome: A systematic review and meta-analysis
Source: PLoS One. 2025 Feb 13;20(2):e0314678. doi: 10.1371/journal.pone.0314678 (PMC11824959; doi:10.1371/journal.pone.0314678)
Supplement: S1 Table — (DOCX) [file pone.0314678.s001.docx]

**S1 Table.** List of articles identified from different databases:

| 1 | Zou, L., J.R. Ruan, J.Y. Chen, J.J. Wang, S.S. Zhu, L.M. Liao, et al., [Moxibustion relieves colonic inflammation by up-regulating expression of miR-345-3p/miR-216a-5p and down-regulating NF-κB p65 in colonic tissue of rats with diarrhea-predominant irritable bowel syndrome]. Zhen Ci Yan Jiu, 2023. 48(3): p. 226-232. |
| --- | --- |
| 2 | Ziessman, H.A., Functional hepatobiliary disease: Chronic acalculous gallbladder and chronic acalculous biliary disease. Seminars in Nuclear Medicine, 2006. 36(2): p. 119-132. |
| 3 | Zhu, Y., Z. Wu, X. Ma, H. Liu, C. Bao, L. Yang, et al., Brain regions involved in moxibustion-induced analgesia in irritable bowel syndrome with diarrhea: A functional magnetic resonance imaging study. BMC Complementary and Alternative Medicine, 2014. 14(1). |
| 4 | Zhu, Y., Z. Wu, X. Ma, H. Liu, C. Bao, L. Yang, et al., Brain regions involved in moxibustion-induced analgesia in irritable bowel syndrome with diarrhea: a functional magnetic resonance imaging study. BMC Complement Altern Med, 2014. 14: p. 500. |
| 5 | Zhu, Y., Z. Wu, X. Ma, H. Liu, C. Bao, L. Yang, et al., Brain regions involved in moxibustion-induced analgesia in irritable bowel syndrome with diarrhea: a functional magnetic resonance imaging study. BMC complementary and alternative medicine, 2014. 14: p. 500. |
| 6 | Zhu, S.S., J.J. Wang, L. Zou, J.Y. Chen, K.W. Li, L.M. Liao, et al., [Anti-inflammation effect of moxibustion for rats with diarrhea-predominant irritable bowel syndrome based on multiple miRNAs regulating NF-κB signal pathway]. Zhongguo Zhen Jiu, 2022. 42(6): p. 654-662. |
| 7 | Zhu, M., J. Gong, and Y. Liu, On the natural medical features of traditional Chinese medicine. Journal of Traditional Chinese Medicine, 2007. 27(2): p. 158-160. |
| 8 | Zhu, L.P., Y.H. Ma, S.S. Ye, and Z.Q. Shu, Acupuncture for Diarrhoea-Predominant Irritable Bowel Syndrome: A Network Meta-Analysis. EVIDENCE-BASED COMPLEMENTARY AND ALTERNATIVE MEDICINE, 2018. 2018. |
| 9 | Zhu, L.P., Y.H. Ma, and X.Y. Deng, Comparison of acupuncture and other drugs for chronic constipation: A network meta-analysis. PLOS ONE, 2018. 13(4). |
| 10 | Zhu, L., Y. Ma, S. Ye, and Z. Shu, Acupuncture for Diarrhoea-Predominant Irritable Bowel Syndrome: A Network Meta-Analysis. Evidence-based Complementary and Alternative Medicine, 2018. 2018. |
| 11 | Zhu, L., Y. Ma, and X. Deng, Comparison of acupuncture and other drugs for chronic constipation: A network meta-analysis. PLoS ONE, 2018. 13(4). |
| 12 | Zhu, L., Z. Li, B. Xu, C. Xia, J. Cheng, X. Xiang, et al., Effects of electroacupuncture at Zusanli (ST 36) on neurons in the colonic myenteric plexus in rats with irritable bowel syndrome with constipation. Neural Regeneration Research, 2011. 6(33): p. 2605-2609. |
| 13 | Zhou, Z.X., H.F. Ma, Y.C. Yang, J.T. Chen, and Y.H. Feng, Effect of electroacupuncture on colonic motility and RhoA and ROCK protein expression in IBS-D rats. Zhen ci yan jiu = Acupuncture research, 2022. 47(7): p. 611-616. |
| 14 | Zhou, Z.X., H.F. Ma, Y.C. Yang, J.T. Chen, and Y.H. Feng, [Effect of electroacupuncture on colonic motility and RhoA and ROCK protein expression in IBS-D rats]. Zhen Ci Yan Jiu, 2022. 47(7): p. 611-616. |
| 15 | Zhou, Y.Y., N.J. Wanner, Y. Xiao, X.Z. Shi, X.H. Jiang, J.G. Gu, et al., Electroacupuncture alleviates stress-induced visceral hypersensitivity through an opioid system in rats. World J Gastroenterol, 2012. 18(48): p. 7201-7211. |
| 16 | Zhou, X.L., H. Wang, J. Li, S. Wu, F. Wu, W. Lu, et al., Effect of acupuncture-moxibustion stimulation of combined "Biao-Ben" acupoints on autonomic nervous activity and related factors in rats with irritable bowel syndrome diarrhea. Zhen ci yan jiu = Acupuncture research, 2023. 48(7): p. 635-642. |
| 17 | Zhou, X.L., H. Wang, J. Li, S. Wu, F. Wu, W. Lu, et al., [Effect of acupuncture-moxibustion stimulation of combined "Biao-Ben" acupoints on autonomic nervous activity and related factors in rats with irritable bowel syndrome diarrhea]. Zhen Ci Yan Jiu, 2023. 48(7): p. 635-642. |
| 18 | Zhou, J., B.M. Zhou, X.Y. Kou, T. Jian, L.M. Chen, X.H. Lei, et al., Effect of summer acupoint application treatment (SAAT) on gut microbiota in healthy Asian adults: A randomized controlled trial. MEDICINE, 2023. 102(9). |
| 19 | Zhou, J., B. Zhou, X. Kou, T. Jian, L. Chen, X. Lei, et al., Effect of summer acupoint application treatment (SAAT) on gut microbiota in healthy Asian adults: A randomized controlled trial. Medicine (United States), 2023. 102(9): p. E32951. |
| 20 | Zhou, J., B. Zhou, X. Kou, T. Jian, L. Chen, X. Lei, et al., Effect of summer acupoint application treatment (SAAT) on gut microbiota in healthy Asian adults: A randomized controlled trial. Medicine, 2023. 102(9): p. e32951. |
| 21 | Zhou, H.F., S.Q. Ding, Y.J. Ding, L.L. Wang, H. Liu, J. Fang, et al., [Observation on effect characteristics of electroacupuncture for different types of functional constipation]. Zhongguo zhen jiu = Chinese acupuncture & moxibustion, 2014. 34(5): p. 435-438. |
| 22 | Zhou, H.F., S.Q. Ding, Y.J. Ding, L.L. Wang, H. Liu, J. Fang, et al., [Observation on effect characteristics of electroacupuncture for different types of functional constipation]. Zhongguo Zhen Jiu, 2014. 34(5): p. 435-438. |
| 23 | Zhong, L.L.D., T.F. Lam, W. Yang, Y. Zheng, Z.P. Lyu, and Z.X. Bian, Electro-acupuncture for irritable bowel syndrome patients: study protocol for a single-blinded randomized sham-controlled clinical trial. TRIALS, 2021. 22(1). |
| 24 | Zhong, L.L.D., T.F. Lam, W. Yang, Y. Zheng, Z. Lyu, and Z. Bian, Electro-acupuncture for irritable bowel syndrome patients: study protocol for a single-blinded randomized sham-controlled clinical trial. Trials, 2021. 22(1). |
| 25 | Zhong, L.L.D., T.F. Lam, W. Yang, Y. Zheng, Z. Lyu, and Z. Bian, Electro-acupuncture for irritable bowel syndrome patients: study protocol for a single-blinded randomized sham-controlled clinical trial. Trials, 2021. 22(1): p. 619. |
| 26 | Zhong, L.L.D., T.F. Lam, W. Yang, Y. Zheng, Z. Lyu, and Z. Bian, Electro-acupuncture for irritable bowel syndrome patients: study protocol for a single-blinded randomized sham-controlled clinical trial. Trials, 2021. 22(1): p. 619. |
| 27 | Zhenzhong, L., Y. Xiaojun, T. Weijun, C. Yuehua, S. Jie, Z. Jimeng, et al., Comparative effect of electroacupuncture and moxibustion on the expression of substance P and vasoactive intestinal peptide in patients with irritable bowel syndrome. Journal of traditional Chinese medicine = Chung i tsa chih ying wen pan / sponsored by All-China Association of Traditional Chinese Medicine, Academy of Traditional Chinese Medicine, 2015. 35(4): p. 402-410. |
| 28 | Zhenzhong, L., Y. Xiaojun, T. Weijun, C. Yuehua, S. Jie, Z. Jimeng, et al., Comparative effect of electroacupuncture and moxibustion on the expression of substance P and vasoactive intestinal peptide in patients with irritable bowel syndrome. Journal of traditional chinese medicine = chung i tsa chih ying wen pan, 2015. 35(4): p. 402‐410. |
| 29 | Zhenzhong, L., Y. Xiaojun, T. Weijun, C. Yuehua, S. Jie, Z. Jimeng, et al., Comparative effect of electroacupuncture and moxibustion on the expression of substance P and vasoactive intestinal peptide in patients with irritable bowel syndrome. J Tradit Chin Med, 2015. 35(4): p. 402-410. |
| 30 | Zheng, X., M.J. Zhi, L.L. Zhao, L. Chen, Z.Q. He, S.S. Jing, et al., Effect mechanism of blistering moxibustion on visceral hypersensitivity of irritable bowel syndrome in mice based on 5-HT signal pathway. Zhongguo zhen jiu = Chinese acupuncture & moxibustion, 2022. 42(7): p. 773-778. |
| 31 | Zheng, X., M.J. Zhi, L.L. Zhao, L. Chen, Z.Q. He, S.S. Jing, et al., [Effect mechanism of blistering moxibustion on visceral hypersensitivity of irritable bowel syndrome in mice based on 5-HT signal pathway]. Zhongguo Zhen Jiu, 2022. 42(7): p. 773-778. |
| 32 | Zheng, Q.H., J. Wang, H. Zheng, L.Y. Lu, S.Y. Zhou, X.Y. Hao, et al., What types of patients with chronic diarrhea benefit more from acupuncture treatment? A secondary analysis of a randomized controlled trial. EUROPEAN JOURNAL OF INTEGRATIVE MEDICINE, 2020. 35. |
| 33 | Zheng, L., New insights into the interplay between intestinal flora and bile acids in inflammatory bowel disease. World Journal of Clinical Cases, 2022. 10(30): p. 10823-10839. |
| 34 | Zheng, H.Z., R.X. Chen, X.F. Zhao, G.H. Li, Y. Liang, H. Zhang, et al., Comparison between the Effects of Acupuncture Relative to Other Controls on Irritable Bowel Syndrome: A Meta-Analysis. PAIN RESEARCH & MANAGEMENT, 2019. 2019. |
| 35 | Zheng, H., J. Xu, X. Sun, F. Zeng, Y. Li, X. Wu, et al., Electroacupuncture for patients with refractory functional dyspepsia: A randomized controlled trial. NEUROGASTROENTEROLOGY AND MOTILITY, 2018. 30(7). |
| 36 | Zheng, H., Z.S. Liu, W. Zhang, M. Chen, F. Zhong, X.H. Jing, et al., Acupuncture for patients with chronic functional constipation: A randomized controlled trial. NEUROGASTROENTEROLOGY AND MOTILITY, 2018. 30(7). |
| 37 | Zheng, H., Y. Li, W. Zhang, F. Zeng, S.Y. Zhou, H.B. Zheng, et al., Electroacupuncture for patients with diarrhea-predominant irritable bowel syndrome or functional diarrhea A randomized controlled trial. MEDICINE, 2016. 95(24). |
| 38 | Zheng, H., Y. Li, W. Zhang, F. Zeng, S.Y. Zhou, H.B. Zheng, et al., Electroacupuncture for patients with diarrheapredominant irritable bowel syndrome or functional diarrhea A randomized controlled trial. Medicine (United States), 2016. 95(24). |
| 39 | Zheng, H., Y. Li, W. Zhang, F. Zeng, S.Y. Zhou, H.B. Zheng, et al., Electroacupuncture for patients with diarrhea-predominant irritable bowel syndrome or functional diarrhea: A randomized controlled trial. Medicine (Baltimore), 2016. 95(24): p. e3884. |
| 40 | Zheng, H., Y. Li, W. Zhang, F. Zeng, S.Y. Zhou, H.B. Zheng, et al., Electroacupuncture for patients with diarrheapredominant irritable bowel syndrome or functional diarrhea A randomized controlled trial. Medicine (united states), 2016. 95(24) (no pagination). |
| 41 | Zheng, H., Y. Li, W. Zhang, F. Zeng, S.Y. Zhou, H.B. Zheng, et al., Electroacupuncture for patients with diarrhea-predominant irritable bowel syndrome or functional diarrhea: a randomized controlled trial. Medicine, 2016. 95(24): p. e3884. |
| 42 | Zheng, H., R. Chen, X. Zhao, G. Li, Y. Liang, H. Zhang, et al., Comparison between the Effects of Acupuncture Relative to Other Controls on Irritable Bowel Syndrome: A Meta-Analysis. Pain Research and Management, 2019. 2019. |
| 43 | Zheng, H., R. Chen, X. Zhao, G. Li, Y. Liang, H. Zhang, et al., Comparison between the Effects of Acupuncture Relative to Other Controls on Irritable Bowel Syndrome: A Meta-Analysis. Pain Res Manag, 2019. 2019: p. 2871505. |
| 44 | Zheng, H., Q. Chen, M. Chen, X. Wu, T.W. She, J. Li, et al., Nonpharmacological conservative treatments for chronic functional constipation: A systematic review and network meta-analysis. NEUROGASTROENTEROLOGY AND MOTILITY, 2019. 31(1). |
| 45 | Zheng, C.H., G.Y. Huang, X.H. Xu, Y. Wang, M.M. Zhang, W. Wang, et al., Electro-acupuncture with different current intensities to treat functional constipation: a study protocol for a randomized controlled trial. TRIALS, 2013. 14. |
| 46 | Zhao, Z.R., Y.X. Wang, F.Y. Xu, W.C. Zhang, Q.Y. Wang, and W. Huang, Herbal-moxa plaster for diarrhea type irritable bowel syndrome of spleen and kidney yang deficiency: a randomized controlled trial. Zhongguo zhen jiu [Chinese acupuncture & moxibustion], 2023. 43(6): p. 617‐621. |
| 47 | Zhao, Z.R., Y.X. Wang, F.Y. Xu, W.C. Zhang, Q.Y. Wang, and W. Huang, [Herbal-moxa plaster for diarrhea type irritable bowel syndrome of spleen and kidney yang deficiency: a randomized controlled trial]. Zhongguo Zhen Jiu, 2023. 43(6): p. 617-621. |
| 48 | Zhao, Y., H.L. Jiang, Y. Shi, W. Zhang, L.X. Zhang, Y.J. Hou, et al., Electroacupuncture Alleviates Visceral Hypersensitivity in IBS-D Rats by Inhibiting EGCs Activity through Regulating BDNF/TrkB Signaling Pathway. Evidence-based Complementary and Alternative Medicine, 2022. 2022. |
| 49 | Zhao, Y., H.L. Jiang, Y. Shi, W. Zhang, L.X. Zhang, Y.J. Hou, et al., Electroacupuncture Alleviates Visceral Hypersensitivity in IBS-D Rats by Inhibiting EGCs Activity through Regulating BDNF/TrkB Signaling Pathway. Evid Based Complement Alternat Med, 2022. 2022: p. 2497430. |
| 50 | Zhao, M., M. Zhao, Z. Wang, Z. Wang, Z. Weng, Z. Weng, et al., Electroacupuncture Improves IBS Visceral Hypersensitivity by Inhibiting the Activation of Astrocytes in the Medial Thalamus and Anterior Cingulate Cortex. Evidence-based Complementary and Alternative Medicine, 2020. 2020. |
| 51 | Zhao, M., Z. Wang, Z. Weng, F. Zhang, G. Li, Z. Ma, et al., Electroacupuncture Improves IBS Visceral Hypersensitivity by Inhibiting the Activation of Astrocytes in the Medial Thalamus and Anterior Cingulate Cortex. Evid Based Complement Alternat Med, 2020. 2020: p. 2562979. |
| 52 | Zhao, J.M., L.Y. Wu, H.R. Liu, H.Y. Hu, J.Y. Wang, R.J. Huang, et al., Factorial study of moxibustion in treatment of diarrhea-predominant irritable bowel syndrome. World Journal of Gastroenterology, 2014. 20(37): p. 13563-13572. |
| 53 | Zhao, J.M., L.Y. Wu, H.R. Liu, H.Y. Hu, J.Y. Wang, R.J. Huang, et al., Factorial study of moxibustion in treatment of diarrhea-predominant irritable bowel syndrome. World J Gastroenterol, 2014. 20(37): p. 13563-13572. |
| 54 | Zhao, J.M., L.Y. Wu, H.R. Liu, H.Y. Hu, J.Y. Wang, R.J. Huang, et al., Factorial study of moxibustion in treatment of diarrhea-predominant irritable bowel syndrome. World journal of gastroenterology, 2014. 20(37): p. 13563‐13572. |
| 55 | Zhao, J.M., J.H. Lu, X.J. Yin, L.Y. Wu, C.H. Bao, X.K. Chen, et al., Comparison of Electroacupuncture and Mild-Warm Moxibustion on Brain-Gut Function in Patients with Constipation-Predominant Irritable Bowel Syndrome: A Randomized Controlled Trial. CHINESE JOURNAL OF INTEGRATIVE MEDICINE, 2018. 24(5): p. 328-335. |
| 56 | Zhao, J.M., J.H. Lu, X.J. Yin, L.Y. Wu, C.H. Bao, X.K. Chen, et al., Comparison of Electroacupuncture and Mild-Warm Moxibustion on Brain-Gut Function in Patients with Constipation-Predominant Irritable Bowel Syndrome: A Randomized Controlled Trial. Chinese Journal of Integrative Medicine, 2018. 24(5): p. 328-335. |
| 57 | Zhao, J.M., J.H. Lu, X.J. Yin, L.Y. Wu, C.H. Bao, X.K. Chen, et al., Comparison of Electroacupuncture and Mild-Warm Moxibustion on Brain-Gut Function in Patients with Constipation-Predominant Irritable Bowel Syndrome: A Randomized Controlled Trial. Chin J Integr Med, 2018. 24(5): p. 328-335. |
| 58 | Zhao, J.M., J.H. Lu, X.J. Yin, L.Y. Wu, C.H. Bao, X.K. Chen, et al., Comparison of Electroacupuncture and Mild-Warm Moxibustion on Brain-Gut Function in Patients with Constipation-Predominant Irritable Bowel Syndrome: a Randomized Controlled Trial. Chinese journal of integrative medicine, 2018. 24(5): p. 328‐335. |
| 59 | Zhao, J.M., J.H. Lu, X.J. Yin, X.K. Chen, Y.H. Chen, W.J. Tang, et al., Comparison of electroacupuncture and moxibustion on brain-gut function in patients with diarrhea-predominant irritable bowel syndrome: A randomized controlled trial. CHINESE JOURNAL OF INTEGRATIVE MEDICINE, 2015. 21(11): p. 855-865. |
| 60 | Zhao, J.M., J.H. Lu, X.J. Yin, X.K. Chen, Y.H. Chen, W.J. Tang, et al., Comparison of electroacupuncture and moxibustion on brain-gut function in patients with diarrhea-predominant irritable bowel syndrome: A randomized controlled trial. Chinese Journal of Integrative Medicine, 2015. 21(11): p. 855-865. |
| 61 | Zhao, J.M., J.H. Lu, X.J. Yin, X.K. Chen, Y.H. Chen, W.J. Tang, et al., Comparison of electroacupuncture and moxibustion on brain-gut function in patients with diarrhea-predominant irritable bowel syndrome: A randomized controlled trial. Chin J Integr Med, 2015. 21(11): p. 855-865. |
| 62 | Zhao, J.M., J.H. Lu, X.J. Yin, X.K. Chen, Y.H. Chen, W.J. Tang, et al., Comparison of electroacupuncture and moxibustion on brain-gut function in patients with diarrhea-predominant irritable bowel syndrome: a randomized controlled trial. Chinese journal of integrative medicine, 2015. 21(11): p. 855‐865. |
| 63 | Zhao, J., X. Li, X. Chen, H. Zheng, K. Ye, Y. Shi, et al., Common traditional Chinese medicine therapies for diarrhea-predominant irritable bowel syndrome: An overview of systematic reviews. Journal of Traditional Chinese Medical Sciences, 2022. 9(3): p. 330-339. |
| 64 | Zhao, J., M. Chen, X. Wang, K. Ye, S.H. Shi, H.X. Li, et al., Efficacy of acupuncture in refractory irritable bowel syndrome: study protocol for a randomised controlled trial. BMJ OPEN, 2021. 11(9). |
| 65 | Zhao, J., M. Chen, X. Wang, K. Ye, S. Shi, H. Li, et al., Efficacy of acupuncture in refractory irritable bowel syndrome: Study protocol for a randomised controlled trial. BMJ Open, 2021. 11(9). |
| 66 | Zhao, J., M. Chen, X. Wang, K. Ye, S. Shi, H. Li, et al., Efficacy of acupuncture in refractory irritable bowel syndrome: study protocol for a randomised controlled trial. BMJ Open, 2021. 11(9): p. e045655. |
| 67 | Zhao, J., M. Chen, X. Wang, K. Ye, S. Shi, H. Li, et al., Efficacy of acupuncture in refractory irritable bowel syndrome: study protocol for a randomised controlled trial. BMJ open, 2021. 11(9): p. e045655. |
| 68 | Zhang, Y.N., H.J. Zhao, Y. Wang, Y. Lu, and S.J. Wang, [Effect of Electroacupuncture Intervention on Constipation-predominant Irritable Bow l Syndrome and Colonic CGRP and SP Expression in Rats]. Zhen Ci Yan Jiu, 2016. 41(1): p. 31-34. |
| 69 | Zhang, Y., H. Zhao, Y. Wang, Y. Lu, and S. Wang, Effect of Electroacupuncture Intervention on Constipation-predominant Irritable Bow l Syndrome and Colonic CGRP and SP Expression in Rats. Zhen ci yan jiu = Acupuncture research / [Zhongguo yi xue ke xue yuan Yi xue qing bao yan jiu suo bian ji], 2016. 41(1): p. 31-34. |
| 70 | Zhang, Y., Z.Q. Li, and F. Han, Electroacupuncture for patients with irritable bowel syndrome A systematic review and meta-analysis protocol. MEDICINE, 2018. 97(31). |
| 71 | Zhang, Y., Z. Li, and F. Han, Electroacupuncture for patients with irritable bowel syndrome: A systematic review and meta-analysis protocol. Medicine (United States), 2018. 97(31). |
| 72 | Zhang, Y., Z. Li, and F. Han, Electroacupuncture for patients with irritable bowel syndrome: A systematic review and meta-analysis protocol. Medicine (Baltimore), 2018. 97(31): p. e11627. |
| 73 | Zhang, W. and F. Zhong, Two-way adjusting effect of acupuncture on Tianshu (ST25) and Dachangshu (BL25) for treating functional bowel disease. Planta Medica, 2016. 82(5). |
| 74 | Zhang, R.X., L.X. Lao, K. Ren, and B.M. Berman, Mechanisms of Acupuncture-Electroacupuncture on Persistent Pain. ANESTHESIOLOGY, 2014. 120(2): p. 482-503. |
| 75 | Zhang, N., Y. Cheng, and L. Wang, Mechanism of Traditional Chinese Medicine in Treating Depressive-related Syndromes Based on Intestinal Flora:A Review. Chinese Journal of Experimental Traditional Medical Formulae, 2022. 28(19): p. 267-273. |
| 76 | Zhang, J., H. Xiong, D. Chu, P. Cheng, W. Qian, and S. Liu, Effect and mechanism of electroacupuncture at ST-36 on visceral hypersensitivity in rats. Chinese Journal of Gastroenterology, 2010. 15(11): p. 665-668. |
| 77 | Zhang, J., X. Ge, K. Zhang, Y. Qi, S. Ren, and X. Zhai, Acupuncture for Parkinson's disease-related constipation: current evidence and perspectives. Frontiers in Neurology, 2023. 14. |
| 78 | Zhang, H.C., S.K. Han, and J.L. Tang, [Fifty cases of irritable bowel syndrome of diarrhea type treated with scalp acupuncture]. Zhongguo zhen jiu = Chinese acupuncture & moxibustion, 2011. 31(7): p. 605-606. |
| 79 | Zhang, H.C., S.K. Han, and J.L. Tang, Fifty cases of irritable bowel syndrome of diarrhea type treated with scalp acupuncture. Zhongguo zhen jiu [Chinese acupuncture & moxibustion], 2011. 31(7): p. 605‐606. |
| 80 | Zhang, H.C., S.K. Han, and J.L. Tang, [Fifty cases of irritable bowel syndrome of diarrhea type treated with scalp acupuncture]. Zhongguo Zhen Jiu, 2011. 31(7): p. 605-606. |
| 81 | Zhang, H.C., S.K. Han, and J.L. Tang, Tonifying qi Invigorating blood Dispersing wind combined with scalp acupuncture for treating diarrhea-predominant irritable bowel syndrome in 50 Cases. Journal of traditional chinese medicine [zhong yi za zhi], 2010. 51(Sl): p. 220‐221. |
| 82 | Zhang, H., F. Xie, H. Gong, H. Huang, S. Chen, M. Kang, et al., [Effects of heat-sensitive moxibustion on HPA axis in rats with irritable bowel syndrome]. Zhongguo Zhen Jiu, 2017. 37(12): p. 1315-1321. |
| 83 | Zhang, H., Z. Bian, and Z. Lin, Are acupoints specific for diseases? A systematic review of the randomized controlled trials with sham acupuncture controls. Chinese Medicine, 2010. 5. |
| 84 | Zhang, G.Z., T. Zhang, Z. Cao, Z.J. Tao, T.H. Wan, M.X. Yao, et al., Effects and Mechanisms of Acupuncture on Diarrhea-Predominant Irritable Bowel Syndrome: A Systematic Review. FRONTIERS IN NEUROSCIENCE, 2022. 16. |
| 85 | Zhang, G., T. Zhang, Z. Cao, Z. Tao, T. Wan, M. Yao, et al., Effects and Mechanisms of Acupuncture on Diarrhea-Predominant Irritable Bowel Syndrome: A Systematic Review. Frontiers in Neuroscience, 2022. 16. |
| 86 | Zhang, G., T. Zhang, Z. Cao, Z. Tao, T. Wan, M. Yao, et al., Effects and Mechanisms of Acupuncture on Diarrhea-Predominant Irritable Bowel Syndrome: A Systematic Review. Front Neurosci, 2022. 16: p. 918701. |
| 87 | Zhang, F., Z. Ma, Z. Weng, M. Zhao, H. Zheng, L. Wu, et al., P2X3Receptor in Primary Afferent Neurons Mediates the Relief of Visceral Hypersensitivity by Electroacupuncture in an Irritable Bowel Syndrome Rat Model. Gastroenterology Research and Practice, 2020. 2020. |
| 88 | Zhang, C., L. Guo, Y. Wang, L. Zhang, and T. Chang, Electroacupuncture combined with qibei mixture for diarrhea-predominant irritable bowel syndrome: A randomized controlled trial. World Journal of Acupuncture - Moxibustion, 2018. 28(1): p. 19-24. |
| 89 | Zhang, C., L. Guo, Y. Wang, L. Zhang, and T. Chang, Electroacupuncture combined with qibei mixture for diarrhea-predominant irritable bowel syndrome: a randomized controlled trial. World journal of acupuncture - moxibustion, 2018. 28(1): p. 19‐24. |
| 90 | Zhan, D.W., J.H. Sun, K.T. Luo, L.Z. Xu, J.L. Zhou, L.X. Pei, et al., [Effects and efficacy observation of acupuncture on serum 5-HT in patients with diarrhea-predominant irritable bowel syndrome]. Zhongguo zhen jiu = Chinese acupuncture & moxibustion, 2014. 34(2): p. 135-138. |
| 91 | Zhan, D.W., J.H. Sun, K.T. Luo, L.Z. Xu, J.L. Zhou, L.X. Pei, et al., [Effects and efficacy observation of acupuncture on serum 5-HT in patients with diarrhea-predominant irritable bowel syndrome]. Zhongguo Zhen Jiu, 2014. 34(2): p. 135-138. |
| 92 | Zhan, D.W., J.H. Sun, K.T. Luo, L.Z. Xu, J.L. Zhou, L.X. Pei, et al., Effects and efficacy observation of acupuncture on serum 5-HT in patients with diarrhea-predominant irritable bowel syndrome. Zhongguo zhen jiu [Chinese acupuncture & moxibustion], 2014. 34(2): p. 135‐138. |
| 93 | Zeiter, D.K., Abdominal Pain in Children: From the Eternal City to the Examination Room. Pediatric Clinics of North America, 2017. 64(3): p. 525-541. |
| 94 | Zaslawski, C., Clinical reasoning in traditional Chinese medicine: Implications for clinical research. Clinical Acupuncture and Oriental Medicine, 2003. 4(2-3): p. 94-101. |
| 95 | Yu, L.M., Y.L. Zhang, Y.W. Wang, W. Ye, and B. Lu, Umbilicus acupuncture for treatment of diarrhea-type irritable bowel syndrome: Efficacy and impact on brain-gut peptides. World Chinese Journal of Digestology, 2020. 28(13): p. 538-543. |
| 96 | Yu, L.M., Y.L. Zhang, Y.W. Wang, W. Ye, and B. Lu, Umbilicus acupuncture for treatment of diarrhea-type irritable bowel syndrome: efficacy and impact on brain-gut peptides. World chinese journal of digestology, 2020. 28(13): p. 538‐543. |
| 97 | Youssef, N.N., Childhood and adolescent constipation: Review and advances in management. Current Treatment Options in Gastroenterology, 2007. 10(5): p. 401-411. |
| 98 | Yoon, S.L., O. Grundmann, L. Koepp, and L. Farrell, Management of irritable bowel syndrome (IBS) in adults: Conventional and complementary/alternative approaches. Alternative Medicine Review, 2011. 16(2): p. 134-151. |
| 99 | Yoon, S.L., O. Grundmann, L. Koepp, and L. Farrell, Management of irritable bowel syndrome (IBS) in adults: conventional and complementary/alternative approaches. Altern Med Rev, 2011. 16(2): p. 134-151. |
| 100 | Yin, X.W., S.W. Li, M.H. Ouyang, R. Ou, Z.H. Chen, S. Wei, et al., Acupoint application therapy for diarrhea-predominant irritable bowel syndrome: a protocol for systematic review and network meta-analysis. ANNALS OF PALLIATIVE MEDICINE, 2022. |
| 101 | Yin, X., S. Li, M. Ouyang, R. Ou, Z. Chen, S. Wei, et al., Acupoint application therapy for diarrhea-predominant irritable bowel syndrome: a protocol for systematic review and network meta-analysis. Annals of palliative medicine, 2022. 11(12): p. 3785-3793. |
| 102 | Yin, X., S. Li, M. Ouyang, R. Ou, Z. Chen, S. Wei, et al., Acupoint application therapy for diarrhea-predominant irritable bowel syndrome: a protocol for systematic review and network meta-analysis. Ann Palliat Med, 2022. 11(12): p. 3785-3793. |
| 103 | Yin, T., Z.X. He, P.H. Ma, L.K. Hou, L. Chen, K.N. Xie, et al., Effect and cerebral mechanism of acupuncture treatment for functional constipation: study protocol for a randomized controlled clinical trial. TRIALS, 2019. 20. |
| 104 | Yik, Y.I., L. Stathopoulos, J.M. Hutson, and B.R. Southwell, Home Transcutaneous Electrical Stimulation Therapy to Treat Children With Anorectal Retention: A Pilot Study. NEUROMODULATION, 2016. 19(5): p. 515-521. |
| 105 | Ye, Z., X.Q. Wei, S.Q. Feng, Q.H. Gu, J. Li, L. Kuai, et al., Effectiveness and safety of acupuncture for postoperative ileus following gastrointestinal surgery: A systematic review and meta-analysis. PLOS ONE, 2022. 17(7). |
| 106 | Ye, Y., C.C. Zhou, H.Q. Hu, I. Fukuzawa, and H.L. Zhang, Underlying mechanisms of acupuncture therapy on polycystic ovary syndrome: Evidences from animal and clinical studies. Frontiers in Endocrinology, 2022. 13. |
| 107 | Yazdani, S. and L. Zeltzer, Treatment of chronic pain in children and adolescents. Pain Management, 2013. 3(4): p. 303-314. |
| 108 | Yao, J.P., Y. Zhao, Y. Chen, L.P. Chen, X.M. Feng, Y. Li, et al., Effect of electroacupuncture on intestinal epithelial mucosal barrier function in rats with diarrhea-predominant irritable bowel syndrome. Zhen ci yan jiu = Acupuncture research, 2020. 45(5): p. 357-362. |
| 109 | Yao, J.P., Y. Zhao, Y. Chen, L.P. Chen, X.M. Feng, Y. Li, et al., [Effect of electroacupuncture on intestinal epithelial mucosal barrier function in rats with diarrhea-predominant irritable bowel syndrome]. Zhen Ci Yan Jiu, 2020. 45(5): p. 357-362. |
| 110 | Yao, C.J., Y.L. Li, M.J. Pu, L.H. Luo, and P.M. Feng, Traditional Chinese medicine for irritable bowel syndrome A protocol for meta-analysis. MEDICINE, 2020. 99(48). |
| 111 | Yang, Y.M., K.H. Rao, K. Zhan, M. Shen, H. Zheng, S.M. Qin, et al., Clinical evidence of acupuncture and moxibustion for irritable bowel syndrome: A systematic review and meta-analysis of randomized controlled trials. FRONTIERS IN PUBLIC HEALTH, 2022. 10. |
| 112 | Yang, Y.C., Z.X. Zhou, T. Xue, Y.H. Feng, J.T. Chen, T.N. Wang, et al., Effect of electroacupuncture on visceral sensitivity and colonic NGF, TrkA, TRPV1 expression in IBS-D rats. Zhongguo zhen jiu = Chinese acupuncture & moxibustion, 2022. 42(12): p. 1395-1402. |
| 113 | Yang, Y.C., Z.X. Zhou, T. Xue, Y.H. Feng, J.T. Chen, T.N. Wang, et al., [Effect of electroacupuncture on visceral sensitivity and colonic NGF, TrkA, TRPV1 expression in IBS-D rats]. Zhongguo Zhen Jiu, 2022. 42(12): p. 1395-1402. |
| 114 | Yang, Y., K. Rao, K. Zhan, M. Shen, H. Zheng, S. Qin, et al., Clinical evidence of acupuncture and moxibustion for irritable bowel syndrome: A systematic review and meta-analysis of randomized controlled trials. Frontiers in public health, 2022. 10: p. 1022145. |
| 115 | Yang, Y., K. Rao, K. Zhan, M. Shen, H. Zheng, S. Qin, et al., Clinical evidence of acupuncture and moxibustion for irritable bowel syndrome: A systematic review and meta-analysis of randomized controlled trials. Front Public Health, 2022. 10: p. 1022145. |
| 116 | Yang, N.N., J.W. Yang, C.X. Tan, Y.J. Li, Y. Wang, L.Y. Qi, et al., The Influence of Psychological Status on Acupuncture for Postprandial Distress Syndrome: A Subgroup Analysis of a Multicenter, Randomized Controlled Trial. EVIDENCE-BASED COMPLEMENTARY AND ALTERNATIVE MEDICINE, 2022. 2022. |
| 117 | Yang, N.N., C.X. Tan, L.L. Lin, X.T. Su, Y.J. Li, L.Y. Qi, et al., Potential Mechanisms of Acupuncture for Functional Dyspepsia Based on Pathophysiology. Frontiers in Neuroscience, 2022. 15. |
| 118 | Yang, M.X., L. Zhao, J. Yang, X.X. Cao, Z.N. Yu, and F.R. Liang, [Bibliometrics analysis on researches of illness spectrum for acu-moxibustion therapy and prospect]. Zhen ci yan jiu = Acupuncture research / [Zhongguo yi xue ke xue yuan Yi xue qing bao yan jiu suo bian ji], 2014. 39(3): p. 247-251. |
| 119 | Yang, L., Y. Lu, H.F. Zhang, X.P. Ma, C.H. Bao, H.G. Wu, et al., Effect of warming moxibustion Tianshu (ST 25, bilateral) and Qihai (CV 6) for the treatment of diarrhea-dominant irritable bowel syndrome: a patient-blinded pilot trial with orthogonal design. JOURNAL OF TRADITIONAL CHINESE MEDICINE, 2017. 37(4): p. 538-545. |
| 120 | Yang, J.Y., D.Q. Tao, Y. Zeng, J. Yang, D. Xu, T. Shi, et al., Modified Shenling Baizhu powder combined with electroacupuncture for treatment of diarrhea-predominant irritable bowel syndrome: Effect on clinical symptoms and psychological state. World Chinese Journal of Digestology, 2017. 25(12): p. 1115-1122. |
| 121 | Yang, J., B. Shang, H. Shi, S. Zhu, G. Lu, and F. Dai, The role of toll-like receptor 4 and mast cell in the ameliorating effect of electroacupuncture on visceral hypersensitivity in rats. Neurogastroenterology and Motility, 2019. 31(6). |
| 122 | Yang, J., B. Shang, H. Shi, S. Zhu, G. Lu, and F. Dai, The role of toll-like receptor 4 and mast cell in the ameliorating effect of electroacupuncture on visceral hypersensitivity in rats. Neurogastroenterol Motil, 2019. 31(6): p. e13583. |
| 123 | Yang, D.Y., H. Wang, J. Li, S. Wu, J.M. Liu, D. Wang, et al., Effect of Electroacupuncture at "Zusanli" (ST 36) on Vimentin (A Kind of Cytoskeleton Protein Related to Smooth Muscle Contraction) in Rats with Diarrhea-predominant Irritable Bowel Syndrome. Zhen ci yan jiu = Acupuncture research, 2017. 42(5): p. 402-406. |
| 124 | Yang, D.Y., H. Wang, J. Li, S. Wu, J.M. Liu, D. Wang, et al., [Effect of Electroacupuncture at "Zusanli" (ST 36) on Vimentin (A Kind of Cytoskeleton Protein Related to Smooth Muscle Contraction) in Rats with Diarrhea-predominant Irritable Bowel Syndrome]. Zhen Ci Yan Jiu, 2017. 42(5): p. 402-406. |
| 125 | Yan, J., Z.W. Miao, J. Lu, F. Ge, L.H. Yu, W.B. Shang, et al., Acupuncture plus Chinese Herbal Medicine for Irritable Bowel Syndrome with Diarrhea: A Systematic Review and Meta-Analysis. EVIDENCE-BASED COMPLEMENTARY AND ALTERNATIVE MEDICINE, 2019. 2019. |
| 126 | Yan, J., Z.W. Miao, J. Lu, F. Ge, L.H. Yu, W.B. Shang, et al., Acupuncture plus Chinese Herbal Medicine for Irritable Bowel Syndrome with Diarrhea: A Systematic Review and Meta-Analysis. Evidence-based Complementary and Alternative Medicine, 2019. 2019. |
| 127 | Yan, J., Z.W. Miao, J. Lu, F. Ge, L.H. Yu, W.B. Shang, et al., Acupuncture plus Chinese Herbal Medicine for Irritable Bowel Syndrome with Diarrhea: A Systematic Review and Meta-Analysis. Evid Based Complement Alternat Med, 2019. 2019: p. 7680963. |
| 128 | Yan, H. and Q. Du, Advances in the treatment of irritable bowel syndrome. Chinese Journal of Gastroenterology, 2007. 12(7): p. 442-445. |
| 129 | Yaklai, K., S. Pattanakuhar, N. Chattipakorn, and S.C. Chattipakorn, The Role of Acupuncture on the Gut-Brain-Microbiota Axis in Irritable Bowel Syndrome. American Journal of Chinese Medicine, 2021. 49(2): p. 285-314. |
| 130 | Xue, H. and S. Shao, Effect of acupuncture combined with Astragalus injection on peripheral blood inflammatory factors in children with diarrhea-predominant irritable bowel syndrome. European Journal of Inflammation, 2019. 17. |
| 131 | Xue, H. and S. Shao, Effect of acupuncture combined with Astragalus injection on peripheral blood inflammatory factors in children with diarrhea-predominant irritable bowel syndrome. European journal of inflammation, 2019. 17. |
| 132 | Xuan, Y.C., J. Liu, Y.Y. Huang, X.Y. Lu, L.L. Geng, X.M. Jiang, et al., Therapeutic effect of long-snake moxibustion combined with western medication on diarrhea type irritable bowel syndrome of spleen and kidney yang deficiency. Zhongguo zhen jiu [Chinese acupuncture & moxibustion], 2021. 41(2): p. 133‐136. |
| 133 | Xuan, Y.C., J. Liu, Y.Y. Huang, X.Y. Lu, L.L. Geng, X.M. Jiang, et al., [Therapeutic effect of long-snake moxibustion combined with western medication on diarrhea type irritable bowel syndrome of spleen and kidney yang deficiency]. Zhongguo Zhen Jiu, 2021. 41(2): p. 133-136. |
| 134 | Xu, S. and L. Lao, From basic science studies to clinical trials: What recent acupuncture research tells us. Medical Acupuncture, 2012. 24(1): p. 10-14. |
| 135 | Xu, G.Y., J.H. Winston, and J.D.Z. Chen, Electroacupuncture attenuates visceral hyperalgesia and inhibits the enhanced excitability of colon specific sensory neurons in a rat model of irritable bowel syndrome. Neurogastroenterology and Motility, 2009. 21(12): p. 1302-1308+e1125. |
| 136 | Xiong, X.R. and Y. Lin, Acupuncture and moxibustion and TCM simultaneously for treating Diarrhea-predominant irritable bowel syndrome in 42 Cases. Journal of fujian university of TCM [fu jian zhong yi xue yuan xue bao], 2008. 18(2): p. 38‐40. |
| 137 | Xiong, P. and W. Yang, The high prevalence and burden of irritable bowel syndrome among university students. General Hospital Psychiatry, 2023. 84: p. 253-255. |
| 138 | Xing, L.Y., L.X. Qu, H. Chen, and S. Gao, [Clinical observation on acupressure at Jiaji points for irritable bowel syndrome]. Zhongguo zhen jiu = Chinese acupuncture & moxibustion, 2013. 33(8): p. 739-742. |
| 139 | Xing, L.Y., L.X. Qu, H. Chen, and S. Gao, [Clinical observation on acupressure at Jiaji points for irritable bowel syndrome]. Zhongguo zhen jiu = Chinese acupuncture & moxibustion, 2013. 33(8): p. 739-742. |
| 140 | Xing, L.Y., L.X. Qu, H. Chen, and S. Gao, Clinical observation on acupressure at Jiaji points for irritable bowel syndrome. Zhongguo zhen jiu [Chinese acupuncture & moxibustion], 2013. 33(8): p. 739‐742. |
| 141 | Xing, L.Y., L.X. Qu, H. Chen, and S. Gao, [Clinical observation on acupressure at Jiaji points for irritable bowel syndrome]. Zhongguo Zhen Jiu, 2013. 33(8): p. 739-742. |
| 142 | Xiao-Peng, M., L.Y. Tan, Y. Yang, H.G. Wu, B. Jiang, H.R. Liu, et al., Effect of electro-acupuncture on substance P, its receptor and corticotropin-releasing hormone in rats with irritable bowel syndrome. World Journal of Gastroenterology, 2009. 15(41): p. 5211-5217. |
| 143 | Xiao, W.B. and Y.L. Liu, Rectal hypersensitivity reduced by acupoint TENS in patients with diarrhea-predominant irritable bowel syndrome: a pilot study. Digestive diseases and sciences, 2004. 49(2): p. 312‐319. |
| 144 | Xiao, W.B. and Y.L. Liu, Rectal hypersensitivity reduced by acupoint TENS in patients with diarrhea-predominant irritable bowel syndrome: a pilot study. Dig Dis Sci, 2004. 49(2): p. 312-319. |
| 145 | Xiang, H., T. Zhang, A. Al-Danakh, D. Yang, and L. Wang, Neuromodulation in Chronic Pelvic Pain: A Narrative Review. Pain and Therapy, 2022. 11(3): p. 789-816. |
| 146 | Wu, X.L., Y.L. Wang, J.H. Sun, Y.Y. Shu, L.X. Pei, J.L. Zhou, et al., [Clinical observation on acupuncture for diarrhea-predominant irritable bowel syndrome patients in syndrome of liver-stagnation and spleen-deficiency and its impact on Th1/Th2]. 2013. p. 1057-1060. |
| 147 | Wu, X.L., Y.L. Wang, J.H. Sun, Y.Y. Shu, L.X. Pei, J.L. Zhou, et al., Clinical observation on acupuncture for diarrhea-predominant irritable bowel syndrome patients in syndrome of liver-stagnation and spleen-deficiency and its impact on Th1/Th2. Zhongguo zhen jiu [Chinese acupuncture & moxibustion], 2013. 33(12): p. 1057‐1060. |
| 148 | Wu, X.L., Y.L. Wang, J.H. Sun, Y.Y. Shu, L.X. Pei, J.L. Zhou, et al., [Clinical observation on acupuncture for diarrhea-predominant irritable bowel syndrome patients in syndrome of liver-stagnation and spleen-deficiency and its impact on Th1/Th2]. Zhongguo Zhen Jiu, 2013. 33(12): p. 1057-1060. |
| 149 | Wu, X., C.H. Zheng, X.H. Xu, P. Ding, F. Xiong, M. Tian, et al., Electroacupuncture for Functional Constipation: A Multicenter, Randomized, Control Trial. EVIDENCE-BASED COMPLEMENTARY AND ALTERNATIVE MEDICINE, 2017. 2017. |
| 150 | Wu, J.C.Y., Complementary and alternative medicine modalities for the treatment of irritable bowel syndrome: Facts or myths? Gastroenterology and Hepatology, 2010. 6(11): p. 705-711. |
| 151 | Wu, J.C., E.T. Ziea, L. Lao, E.F. Lam, C.S. Chan, A.Y. Liang, et al., Effect of electroacupuncture on visceral hyperalgesia, serotonin and fos expression in an animal model of irritable bowel syndrome. J Neurogastroenterol Motil, 2010. 16(3): p. 306-314. |
| 152 | Wu, J., Q.W. Fu, S.S. Yang, H. Wang, and Y.F. Li, Efficacy and Safety of Acupoint Catgut Embedding for Diarrhea-Predominant Irritable Bowel Syndrome and Constipation-Predominant Irritable Bowel Syndrome: A Systematic Review and Meta-Analysis. EVIDENCE-BASED COMPLEMENTARY AND ALTERNATIVE MEDICINE, 2020. 2020. |
| 153 | Wu, J., Q. Fu, S. Yang, H. Wang, and Y. Li, Efficacy and safety of acupoint catgut embedding for diarrhea-predominant irritable bowel syndrome and constipation-predominant irritable bowel syndrome: A systematic review and meta-analysis. Evidence-based Complementary and Alternative Medicine, 2020. 2020. |
| 154 | Wu, I.X.Y., C.H.L. Wong, R.S.T. Ho, W.K.W. Cheung, A.C. Ford, J.C.Y. Wu, et al., Acupuncture and related therapies for treating irritable bowel syndrome: overview of systematic reviews and network meta-analysis. Integrative Medicine Research, 2020. 9. |
| 155 | Wu, I.X.Y., C.H.L. Wong, R.S.T. Ho, W.K.W. Cheung, A.C. Ford, J.C.Y. Wu, et al., Acupuncture and related therapies for treating irritable bowel syndrome: overview of systematic reviews and network meta-analysis. THERAPEUTIC ADVANCES IN GASTROENTEROLOGY, 2019. 12. |
| 156 | Wu, I.X.Y., C.H.L. Wong, R.S.T. Ho, W.K.W. Cheung, A.C. Ford, J.C.Y. Wu, et al., Acupuncture and related therapies for treating irritable bowel syndrome: overview of systematic reviews and network meta-analysis. Therapeutic Advances in Gastroenterology, 2019. 12. |
| 157 | Wu, I.X.Y., C.H.L. Wong, R.S.T. Ho, W.K.W. Cheung, A.C. Ford, J.C.Y. Wu, et al., Acupuncture and related therapies for treating irritable bowel syndrome: overview of systematic reviews and network meta-analysis. Therap Adv Gastroenterol, 2019. 12: p. 1756284818820438. |
| 158 | Wu, H.G., B. Jiang, E.H. Zhou, Z. Shi, D.R. Shi, Y.H. Cui, et al., Regulatory mechanism of electroacupuncture in irritable bowel syndrome: Preventing MC activation and decreasing SP VIP secretion. Digestive Diseases and Sciences, 2008. 53(6): p. 1644-1651. |
| 159 | Wu, H.G., B. Jiang, E.H. Zhou, Z. Shi, D.R. Shi, Y.H. Cui, et al., Regulatory mechanism of electroacupuncture in irritable bowel syndrome: preventing MC activation and decreasing SP VIP secretion. Dig Dis Sci, 2008. 53(6): p. 1644-1651. |
| 160 | Wong, C.H.L., I.X.Y. Wu, R.S.T. Ho, W.K.W. Cheung, A.C. Ford, J.C.Y. Wu, et al., Acupuncture and related therapies for treating irritable bowel syndrome: Overview of systematic reviews and network meta-analysis. Gut, 2018. 67: p. A38. |
| 161 | Włodarczyk, J., A. Waśniewska, J. Fichna, A. Dziki, Ł. Dziki, and M. Włodarczyk, Current overview on clinical management of chronic constipation. Journal of Clinical Medicine, 2021. 10(8). |
| 162 | Winter, J.W. and R.C. Heading, The nonerosive reflux disease-gastroesophageal reflux disease controversy. Current Opinion in Gastroenterology, 2008. 24(4): p. 509-515. |
| 163 | Wiebelitz, K.R. and A.M. Beer, Phytotherapy of chronic abdominal pain following pancreatic carcinoma surgery: A single case observation. International Journal of General Medicine, 2012. 5: p. 845-848. |
| 164 | Whitfield, K.L. and R.J. Schulman, Treatment options for functional gastrointestinal disorders: From empiric to complementary approaches. Pediatric Annals, 2009. 38(5): p. 288-294. |
| 165 | Weng, Z.J., L.Y. Wu, C.L. Zhou, C.Z. Dou, Y. Shi, H.R. Liu, et al., Effect of electroacupuncture on P2X<inf>3</inf> receptor regulation in the peripheral and central nervous systems of rats with visceral pain caused by irritable bowel syndrome. Purinergic Signalling, 2015. 11(3): p. 321-329. |
| 166 | Weng, Z.J., L.Y. Wu, C.L. Zhou, C.Z. Dou, Y. Shi, H.R. Liu, et al., Effect of electroacupuncture on P2X3 receptor regulation in the peripheral and central nervous systems of rats with visceral pain caused by irritable bowel syndrome. Purinergic Signal, 2015. 11(3): p. 321-329. |
| 167 | Wei, X.X., Y.T. Wen, Y.C. Wei, X. Liang, X.X. Ma, B.H. Zhang, et al., External therapy of traditional Chinese medicine for treating irritable bowel syndrome with diarrhea: A systematic review and meta-analysis. FRONTIERS IN MEDICINE, 2022. 9. |
| 168 | Wei, X., Y. Wen, Y. Wei, X. Liang, X. Ma, B. Zhang, et al., External therapy of traditional Chinese medicine for treating irritable bowel syndrome with diarrhea: A systematic review and meta-analysis. Frontiers in Medicine, 2022. 9. |
| 169 | Wei, X., Y. Wen, Y. Wei, X. Liang, X. Ma, B. Zhang, et al., External therapy of traditional Chinese medicine for treating irritable bowel syndrome with diarrhea: A systematic review and meta-analysis. Front Med (Lausanne), 2022. 9: p. 940328. |
| 170 | Wauters, L., R. Dickman, V. Drug, A. Mulak, J. Serra, P. Enck, et al., United European Gastroenterology (UEG) and European Society for Neurogastroenterology and Motility (ESNM) consensus on functional dyspepsia. Neurogastroenterology and Motility, 2021. 33(9). |
| 171 | Wang, Z.W., B.W. Chen, S.Z. Chen, L.L. Zhang, H.W. Sun, and X.L. Chang, Effect of electroacupuncture at "Zusanli" (ST36) and "Tianshu" (ST25) on intestinal function and autonomic nerve balance in rats with irritable bowel syndrome. Zhen ci yan jiu = Acupuncture research, 2023. 48(2): p. 165-171. |
| 172 | Wang, Z.W., B.W. Chen, S.Z. Chen, L.L. Zhang, H.W. Sun, and X.L. Chang, [Effect of electroacupuncture at "Zusanli" (ST36) and "Tianshu" (ST25) on intestinal function and autonomic nerve balance in rats with irritable bowel syndrome]. Zhen Ci Yan Jiu, 2023. 48(2): p. 165-171. |
| 173 | Wang, Z.Q., M.W. Xu, Z. Shi, C.H. Bao, H.R. Liu, C.L. Zhou, et al., Mild moxibustion for Irritable Bowel Syndrome with Diarrhea (IBS-D): A randomized controlled trial. JOURNAL OF ETHNOPHARMACOLOGY, 2022. 289. |
| 174 | Wang, Z., M. Xu, Z. Shi, C. Bao, H. Liu, C. Zhou, et al., Mild moxibustion for Irritable Bowel Syndrome with Diarrhea (IBS-D): A randomized controlled trial. Journal of Ethnopharmacology, 2022. 289. |
| 175 | Wang, Z., M. Xu, Z. Shi, C. Bao, H. Liu, C. Zhou, et al., Mild moxibustion for Irritable Bowel Syndrome with Diarrhea (IBS-D): A randomized controlled trial. J Ethnopharmacol, 2022. 289: p. 115064. |
| 176 | Wang, Z., M. Xu, Z. Shi, C. Bao, H. Liu, C. Zhou, et al., Mild moxibustion for Irritable Bowel Syndrome with Diarrhea (IBS-D): a randomized controlled trial. Journal of ethnopharmacology, 2022. 289: p. 115064. |
| 177 | Wang, Y.N., M.S. Sun, X.X. Ni, T. Tian, L. Liu, X. Li, et al., Comparison of Effects and Brain-Gut Regulatory Mechanisms of Acupuncture and Flunarizine for Migraine: Study Protocol for a Randomized Controlled Trial. EVIDENCE-BASED COMPLEMENTARY AND ALTERNATIVE MEDICINE, 2021. 2021. |
| 178 | Wang, Y.J., D.S. Wang, H.Q. Guan, J. Wang, J.Y. Chai, J.R. Zhao, et al., Effects of eye-acupuncture therapy on serum and colonic SP and VIP contents in rats with irritable bowel syndrome. Zhen ci yan jiu = Acupuncture research / [Zhongguo yi xue ke xue yuan Yi xue qing bao yan jiu suo bian ji], 2010. 35(1): p. 8-11, 26. |
| 179 | Wang, Y.J., D.S. Wang, H.Q. Guan, J. Wang, J.Y. Chai, J.R. Zhao, et al., [Effects of eye-acupuncture therapy on serum and colonic SP and VIP contents in rats with irritable bowel syndrome]. Zhen Ci Yan Jiu, 2010. 35(1): p. 8-11, 26. |
| 180 | Wang, Y.J., H.H. Liu, X.D. Liu, J.Y. Chai, J.R. Zhao, and D.S. Wang, Eye acupuncture therapy up-regulates aquaporin 3 expression in the colon of rats with diarrhea-predominant irritable bowel syndrome. World Chinese Journal of Digestology, 2011. 19(9): p. 899-904. |
| 181 | Wang, X.Y., H. Wang, Y.Y. Guan, R.L. Cai, and G.M. Shen, Acupuncture for functional gastrointestinal disorders: A systematic review and meta-analysis. JOURNAL OF GASTROENTEROLOGY AND HEPATOLOGY, 2021. 36(11): p. 3015-3026. |
| 182 | Wang, X.Y., H. Wang, Y.Y. Guan, R.L. Cai, and G.M. Shen, Acupuncture for functional gastrointestinal disorders: A systematic review and meta-analysis. Journal of Gastroenterology and Hepatology (Australia), 2021. 36(11): p. 3015-3026. |
| 183 | Wang, X.Y., H. Wang, Y.Y. Guan, R.L. Cai, and G.M. Shen, Acupuncture for functional gastrointestinal disorders: A systematic review and meta-analysis. J Gastroenterol Hepatol, 2021. 36(11): p. 3015-3026. |
| 184 | Wang, X.S., X.L. Shi, J. Lv, J.C. Zhang, Y.L. Huo, G. Zuo, et al., Acupuncture and related therapies for the anxiety and depression in irritable bowel syndrome with diarrhea (IBS-D): A network meta-analysis of randomized controlled trials. FRONTIERS IN PSYCHIATRY, 2022. 13. |
| 185 | Wang, X., X. Shi, J. Lv, J. Zhang, Y. Huo, G. Zuo, et al., Acupuncture and related therapies for the anxiety and depression in irritable bowel syndrome with diarrhea (IBS-D): A network meta-analysis of randomized controlled trials. Frontiers in Psychiatry, 2022. 13. |
| 186 | Wang, X., X. Shi, J. Lv, J. Zhang, Y. Huo, G. Zuo, et al., Acupuncture and related therapies for the anxiety and depression in irritable bowel syndrome with diarrhea (IBS-D): A network meta-analysis of randomized controlled trials. Front Psychiatry, 2022. 13: p. 1067329. |
| 187 | Wang, X., Q. Qi, H. Wu, Y. Liu, Y. Wang, C. Wang, et al., Moxibustion modulates the gut microbiota in rats with irritable bowel syndrome. Gastroenterology, 2017. 152(5): p. S632-S633. |
| 188 | Wang, X., Q. Qi, Y. Wang, H. Wu, X. Jin, H. Yao, et al., Gut microbiota was modulated by moxibustion stimulation in rats with irritable bowel syndrome. Chinese Medicine (United Kingdom), 2018. 13(1). |
| 189 | Wang, X., Q. Qi, Y. Wang, H. Wu, X. Jin, H. Yao, et al., Gut microbiota was modulated by moxibustion stimulation in rats with irritable bowel syndrome. Chin Med, 2018. 13: p. 63. |
| 190 | Wang, X., H. Liu, G. Ding, Y. Chen, H. Wu, N. Li, et al., Effects of electroacupuncture on c-Fos expression in the spinal cord and brain of rats with chronic visceral hypersensitivity. Neural Regeneration Research, 2009. 4(5): p. 339-343. |
| 191 | Wang, S.W., Forty-two cases of ulcerative colitis treated with comprehensive TCM therapy. Henan traditional chinese medicine [henan zhong yi], 2016. 36(4): p. 643‐645. |
| 192 | Wang, S.S., X.R. Wang, R.Y. Yang, Y. Xu, and M.Y. Li, Efficacy and mechanism of acupuncture combined with Tongxieyaofang for diarrhea-type irritable bowel syndrome of liver depression and spleen deficiency. Zhongguo zhen jiu = Chinese acupuncture & moxibustion, 2020. 40(6): p. 605-609. |
| 193 | Wang, S.S., X.R. Wang, R.Y. Yang, Y. Xu, and M.Y. Li, Efficacy and mechanism of acupuncture combined with Tongxieyaofang for diarrhea-type irritable bowel syndrome of liver depression and spleen deficiency. Zhongguo zhen jiu [Chinese acupuncture & moxibustion], 2020. 40(6): p. 605‐609. |
| 194 | Wang, S.S., X.R. Wang, R.Y. Yang, Y. Xu, and M.Y. Li, [Efficacy and mechanism of acupuncture combined with Tongxieyaofang for diarrhea-type irritable bowel syndrome of liver depression and spleen deficiency]. Zhongguo Zhen Jiu, 2020. 40(6): p. 605-609. |
| 195 | Wang, S., M.W. Guo, Y.S. Gao, X.X. Ren, Y. Lan, M.X. Ji, et al., Effect of Electroacupuncture at "Neiguan" (PC 6) and "Tianshu" (ST 25) for Colonic Motility and D 2 Receptor in Irritable Bowel Syndrome Rats. Zhen ci yan jiu = Acupuncture research, 2018. 43(1): p. 25-29. |
| 196 | Wang, S., M.W. Guo, Y.S. Gao, X.X. Ren, Y. Lan, M.X. Ji, et al., [Effect of Electroacupuncture at "Neiguan" (PC 6) and "Tianshu" (ST 25) for Colonic Motility and D 2 Receptor in Irritable Bowel Syndrome Rats]. Zhen Ci Yan Jiu, 2018. 43(1): p. 25-29. |
| 197 | Wang, R.S., A.J. Lembo, T.J. Kaptchuk, V. Cheng, J. Nee, J. Iturrino, et al., Genomic Effects Associated With Response to Placebo Treatment in a Randomized Trial of Irritable Bowel Syndrome. Frontiers in pain research (Lausanne, Switzerland), 2021. 2: p. 775386. |
| 198 | Wang, R.S., A.J. Lembo, T.J. Kaptchuk, V. Cheng, J. Nee, J. Iturrino, et al., Genomic Effects Associated With Response to Placebo Treatment in a Randomized Trial of Irritable Bowel Syndrome. Front Pain Res (Lausanne), 2021. 2: p. 775386. |
| 199 | Wang, L. and S. Zhang, Therapeutic study on scalp acupuncture plus body acupuncture for diarrhea-predominant irritable bowel syndrome. Shanghai journal of acupuncture and moxibustion [shang hai zheng jiu za zhi], 2016. 35(4): p. 405‐407. |
| 200 | Wang, L., C.C. Yu, J. Li, Q. Tian, and Y.J. Du, Mechanism of Action of Acupuncture in Obesity: A Perspective From the Hypothalamus. Frontiers in Endocrinology, 2021. 12. |
| 201 | Wang, L., M.M. Xu, Q.H. Zheng, W. Zhang, and Y. Li, The Effectiveness of Acupuncture in Management of Functional Constipation: A Systematic Review and Meta-Analysis. EVIDENCE-BASED COMPLEMENTARY AND ALTERNATIVE MEDICINE, 2020. 2020. |
| 202 | Wang, L., D. Wang, M.M. Xu, W. Cao, Y. Liu, T.H. Hou, et al., Effectiveness of different acupuncture courses for functional constipation A protocol for systematic review and network meta-analysis. MEDICINE, 2020. 99(21). |
| 203 | Wang, F., S.X. He, J. Yan, L.R. Mai, and L.J. Yang, Effects of herb-partitioned moxibustion for diarrhoea-predominant irritable bowel syndrome A protocol for systematic review and meta-analysis. MEDICINE, 2020. 99(34). |
| 204 | Wang, F., S. He, J. Yan, L. Mai, and L. Yang, Effects of herb-partitioned moxibustion for diarrhoea-predominant irritable bowel syndrome: A protocol for systematic review and meta-analysis. Medicine (Baltimore), 2020. 99(34): p. e21817. |
| 205 | Wal, A., P. Wal, N. Verma, A.K. Rai, and P. Vatsha, Mechanism, Pathophysiology and Herbal Management-A Review on Irritable Bowel Syndrome. Natural Products Journal, 2023. 13(3): p. 16-30. |
| 206 | Wadhera, V., D.A. Lemberg, S.T. Leach, and A.S. Day, Complementary and alternative medicine in children attending gastroenterology clinics: Usage patterns and reasons for use. Journal of Paediatrics and Child Health, 2011. 47(12): p. 904-910. |
| 207 | Vlieger, A.M. and M.A. Benninga, Complementary therapies for pediatric functional gastrointestinal disorders. Journal of Pediatric Gastroenterology and Nutrition, 2008. 47(5): p. 707-709. |
| 208 | Villalon-Gomez, J.M., How to meet the challenges of managing patients with IBS. Journal of Family Practice, 2021. 70(9): p. 431-441. |
| 209 | Videlock, E.J., F. Cremonini, E.A. Friedlander, T. Kaptchuk, and A. Lembo, Is the McGill pain questionnaire less sensitive to change in response to a placebo treatment in irritable bowel syndrome in comparison to a single item pain severity scale? Gastroenterology, 2011. 140(5): p. S610-S611. |
| 210 | Videlock, E.J., F. Cremonini, E.A. Friedlander, T. Kaptchuk, and A. Lembo, Is the McGill pain questionnaire less sensitive to change in response to a placebo treatment in irritable bowel syndrome in comparison to a single item pain severity scale? Gastroenterology, 2011. 140(5): p. S610‐S611. |
| 211 | Vercellini, P., P. Vigano, E. Somigliana, A. Abbiati, G. Barbara, and L. Fedele, Medical, surgical and alternative treatments for chronic pelvic pain in women: a descriptive review. GYNECOLOGICAL ENDOCRINOLOGY, 2009. 25(4): p. 208-221. |
| 212 | Vercellini, P., P. Viganò, E. Somigliana, A. Abbiati, G. Barbara, and L. Fedele, Medical, surgical and alternative treatments for chronic pelvic pain in women: A descriptive review. Gynecological Endocrinology, 2009. 25(4): p. 208-221. |
| 213 | Verástegui Escolano, C., Electro-acupuncture decreases 5-HT, CGRP and increases NPY in the brain-gut axis in two rat models of Diarrhea-predominant irritable bowel syndrome(D-IBS). Revista Internacional de Acupuntura, 2016. 10(4): p. 155-157. |
| 214 | Vase, L., S. Baram, N. Takakura, H. Yajima, M. Takayama, T.J. Kaptchuk, et al., Specifying the nonspecific components of acupuncture analgesia. PAIN, 2013. 154(9): p. 1659-1667. |
| 215 | Varghese, C., W. Xu, C. Daker, I.P. Bissett, and C. Cederwall, Clinical utility of Gastric Alimetry® in the management of intestinal failure patients with possible underlying gut motility disorders. Clinical Nutrition Open Science, 2023. 51: p. 15-25. |
| 216 | Van Wunnik, B.P.W., C.G.M.I. Baeten, and B.R. Southwell, Neuromodulation for constipation: Sacral and transcutaneous stimulation. Best Practice and Research: Clinical Gastroenterology, 2011. 25(1): p. 181-191. |
| 217 | Van Tilburg, M.A.L., O.S. Palsson, R.L. Levy, A.D. Feld, M.J. Turner, D.A. Drossman, et al., Complementary and alternative medicine use and cost in functional bowel disorders: A six month prospective study in a large HMO. BMC Complementary and Alternative Medicine, 2008. 8. |
| 218 | van Haselen, R. and R. Jutte, The placebo effect and its ramifications for clinical practice and research. Villa La Collina at Lake Como, Italy, 4-6 May 2012. COMPLEMENTARY THERAPIES IN MEDICINE, 2013. 21(2): p. 85-93. |
| 219 | Van Haselen, R. and R. Jütte, The placebo effect and its ramifications for clinical practice and research. Villa La Collina at Lake Como, Italy, 4-6 May 2012. Complementary Therapies in Medicine, 2013. 21(2): p. 85-93. |
| 220 | Valenzuela, J., J. Alvarado, H. Cohen, A. Damiao, C. Francisconi, L. Frugone, et al., Latin-American consensus document on irritable bowel syndrome. Gastroenterologia y Hepatologia, 2004. 27(5): p. 325-343. |
| 221 | Usai-Satta, P., M. Bellini, M. Lai, F. Oppia, and F. Cabras, Therapeutic approach for irritable bowel syndrome: Old and new Strategies. Current Clinical Pharmacology, 2018. 13(3): p. 164-172. |
| 222 | Tort, S., A. Balboa, M. Marzo, R. Carrillo, M. Mínguez, J. Valdepérez, et al., Clinical practice guideline for irritable bowel syndrome. Gastroenterologia y Hepatologia, 2006. 29(8): p. 467-521. |
| 223 | Tong, L., L.B. Wu, N. Li, H.L. Cheng, R.L. Cai, and H.R. Chu, [Moxibustion relieves abdominal hypersensitivity and diarrhea by regulating colonic 5-hydroxytryptamine signaling pathway in rats with diarrhea type irritable bowel syndrome]. Zhen Ci Yan Jiu, 2020. 45(7): p. 535-540. |
| 224 | Tingting, M., Ideas on trial design in acupuncture for IBS. American Journal of Gastroenterology, 2010. 105(3): p. 699. |
| 225 | Tingting, M., Ideas on trial design in acupuncture for IBS. American journal of gastroenterology, 2010. 105(3): p. 699. |
| 226 | Tingting, M., Ideas on trial design in acupuncture for IBS. Am J Gastroenterol, 2010. 105(3): p. 699. |
| 227 | Tillisch, K. and L. Chang, Diagnosis and treatment of irritable bowel syndrome: State of the art. Current Gastroenterology Reports, 2005. 7(4): p. 249-256. |
| 228 | Tillisch, K., Complementary and alternative medicine for gastrointestinal disorders. Clinical Medicine, Journal of the Royal College of Physicians of London, 2007. 7(3): p. 224-227. |
| 229 | Till, S.R., R. Nakamura, A. Schrepf, and S. As-Sanie, Approach to Diagnosis and Management of Chronic Pelvic Pain in Women: Incorporating Chronic Overlapping Pain Conditions in Assessment and Management. Obstetrics and Gynecology Clinics of North America, 2022. 49(2): p. 219-239. |
| 230 | Tick, E., Asklepian Dream Healing of Irritable Bowel Syndrome. Explore: The Journal of Science and Healing, 2005. 1(4): p. 290-291. |
| 231 | Thukral, C. and J.L. Wolf, Therapy insight: Drugs for gastrointestinal disorders in pregnant women. Nature Clinical Practice Gastroenterology and Hepatology, 2006. 3(5): p. 256-266. |
| 232 | Thomas, L.V., K. Suzuki, and J. Zhao, Probiotics: A proactive approach to health. A symposium report. British Journal of Nutrition, 2015. 114(S1): p. S1-S15. |
| 233 | Teixeira, M.Z., C. Guedes, P.V. Barreto, and M.A. Martins, The placebo effect and homeopathy. HOMEOPATHY, 2010. 99(2): p. 119-129. |
| 234 | Tctr, Efficacy and safety of mild moxibustion in the treatment of diarrhea-predominant irritable bowel syndrome (spleen deficiency and dampness excess syndrome): a study protocol for a randomized controlled trial. https://trialsearch.who.int/Trial2.aspx?TrialID=TCTR20230523002, 2023. |
| 235 | Tao, B., S. Chao, C.H. Zheng, and G.Y. Huang, Acupuncture for the treatment of functional constipation. JOURNAL OF TRADITIONAL CHINESE MEDICINE, 2016. 36(5): p. 578-587. |
| 236 | Tang, Z.P., Traditional Chinese medicine clinical experience of the treatment for irritable bowel syndrome. Chinese Journal of Integrative Medicine, 2009. 15(2): p. 93-94. |
| 237 | Tan, L.H., K.G. Li, Y.Y. Wu, Y. Lan, M.W. Guo, W.L. Zhu, et al., Effect of Electroacupuncture at "Yintang" (GV 29) and "Tianshu" (ST 25) on the Ethology and the Expression of TRPV 1 Receptor in Colon of Irritable Bowel Syndrome Rats. Zhen ci yan jiu = Acupuncture research, 2017. 42(2): p. 136-140. |
| 238 | Tan, L.H., K.G. Li, Y.Y. Wu, Y. Lan, M.W. Guo, W.L. Zhu, et al., [Effect of Electroacupuncture at "Yintang" (GV 29) and "Tianshu" (ST 25) on the Ethology and the Expression of TRPV 1 Receptor in Colon of Irritable Bowel Syndrome Rats]. Zhen Ci Yan Jiu, 2017. 42(2): p. 136-140. |
| 239 | Tan, L.H., K.G. Li, Y.Y. Wu, M.W. Guo, Y. Lan, S. Wang, et al., Effect of Electroacupuncture at Different Acupoints on the Expression of NMDA Receptors in ACC and Colon in IBS Rats. Evidence-based Complementary and Alternative Medicine, 2019. 2019. |
| 240 | Tan, L.H., K.G. Li, Y.Y. Wu, M.W. Guo, Y. Lan, S. Wang, et al., Effect of Electroacupuncture at Different Acupoints on the Expression of NMDA Receptors in ACC and Colon in IBS Rats. Evid Based Complement Alternat Med, 2019. 2019: p. 4213928. |
| 241 | Talotta, R., F. Atzeni, L. Bazzichi, C. Giacomelli, M. Di Franco, F. Salaffi, et al., Algo-dysfunctional syndromes: A critical digest of the recent literature. Clinical and Experimental Rheumatology, 2015. 33: p. S102-S108. |
| 242 | Takahashi, T., Effect and mechanism of acupuncture on gastrointestinal diseases. 2013. p. 273-294. |
| 243 | Takahashi, T., Acupuncture for functional gastrointestinal disorders. Journal of Gastroenterology, 2006. 41(5): p. 408-417. |
| 244 | Sun, Y.Z., S.L. Wang, and T.Y. Yu, Tiaoshen acupuncture method combined with electroacupuncture for diarrhea-type irritable bowel syndrome : a randomized controlled trial. Zhongguo zhen jiu = Chinese acupuncture & moxibustion, 2021. 41(1): p. 13-16. |
| 245 | Sun, Y.Z., S.L. Wang, and T.Y. Yu, Tiaoshen acupuncture method combined with electroacupuncture for diarrhea-type irritable bowel syndrome : a randomized controlled trial. Zhongguo zhen jiu [Chinese acupuncture & moxibustion], 2021. 41(1): p. 13‐16. |
| 246 | Sun, Y.Z., S.L. Wang, and T.Y. Yu, [Tiaoshen acupuncture method combined with electroacupuncture for diarrhea-type irritable bowel syndrome : a randomized controlled trial]. Zhongguo Zhen Jiu, 2021. 41(1): p. 13-16. |
| 247 | Sun, Y.Z. and J. Song, Therapeutic observation of acupuncture at Jiaji (EX-B2) for irritable bowel syndrome. Shanghai journal of acupuncture and moxibustion [shang hai zhen jiu za zhi], 2015. 34(9): p. 856‐857. |
| 248 | Sun, M., Y. Zhang, Y. Song, J. Guo, T. Zhao, Y. Wang, et al., Electroacupuncture at Tianshu (ST25) and Zusanli (ST36) alleviates stress-induced irritable bowel syndrome in mice by modulating gut microbiota and corticotropin-releasing factor. Journal of Traditional Chinese Medicine, 2022. 42(5): p. 732-740. |
| 249 | Sun, M., Y. Zhang, Y. Song, J. Guo, Y. Wang, C. Xin, et al., Electroacupuncture alleviates water avoidance stress-induced irritable bowel syndrome in mice by improving intestinal barrier functions and suppressing the expression of inflammatory cytokines. Journal of Traditional Chinese Medicine, 2023. 43(3): p. 494-500. |
| 250 | Sun, J.W., M.L. Sun, D. Li, J. Zhao, S.H. Shi, H.X. Li, et al., Efficacy of acupuncture based on acupoint combination theory for irritable bowel syndrome: a study protocol for a multicenter randomized controlled trial. TRIALS, 2021. 22(1). |
| 251 | Sun, J.W., M.L. Sun, D. Li, J. Zhao, S.H. Shi, H.X. Li, et al., Efficacy of acupuncture based on acupoint combination theory for irritable bowel syndrome: a study protocol for a multicenter randomized controlled trial. Trials, 2021. 22(1). |
| 252 | Sun, J.W., M.L. Sun, D. Li, J. Zhao, S.H. Shi, H.X. Li, et al., Efficacy of acupuncture based on acupoint combination theory for irritable bowel syndrome: a study protocol for a multicenter randomized controlled trial. Trials, 2021. 22(1): p. 719. |
| 253 | Sun, J.W., M.L. Sun, D. Li, J. Zhao, S.H. Shi, H.X. Li, et al., Efficacy of acupuncture based on acupoint combination theory for irritable bowel syndrome: a study protocol for a multicenter randomized controlled trial. Trials, 2021. 22(1). |
| 254 | Sun, J.H., X.L. Wu, C. Xia, L.Z. Xu, L.X. Pei, H. Li, et al., Clinical evaluation of Soothing Gan and invigorating Pi acupuncture treatment on diarrhea-predominant irritable bowel syndrome. Chinese journal of integrative medicine, 2011. 17(10): p. 780‐785. |
| 255 | Sun, J.H., X.L. Wu, C. Xia, L.Z. Xu, L.X. Pei, H. Li, et al., Clinical evaluation of Soothing Gan and invigorating Pi acupuncture treatment on diarrhea-predominant irritable bowel syndrome. Chin J Integr Med, 2011. 17(10): p. 780-785. |
| 256 | Sun, J.H., X.L. Wu, C. Xia, L.Z. Xu, L.X. Pei, L. Hao, et al., Clinical evaluation of Soothing Gan and invigorating Pi acupuncture treatment on diarrhea-predominant irritable bowel syndrome. Chinese Journal of Integrative Medicine, 2011. 17(10): p. 780-785. |
| 257 | Sun, J., X. Wu, Y. Meng, J. Cheng, H. Ning, Y. Peng, et al., Electro-acupuncture decreases 5-HT, CGRP and increases NPY in the brain-gut axis in two rat models of Diarrhea-predominant irritable bowel syndrome(D-IBS). BMC Complementary and Alternative Medicine, 2015. 15(1). |
| 258 | Sun, J., X. Wu, Y. Meng, J. Cheng, H. Ning, Y. Peng, et al., Electro-acupuncture decreases 5-HT, CGRP and increases NPY in the brain-gut axis in two rat models of Diarrhea-predominant irritable bowel syndrome(D-IBS). BMC Complement Altern Med, 2015. 15: p. 340. |
| 259 | Stuardi, T. and H. MacPherson, Acupuncture for irritable bowel syndrome: Diagnosis and treatment of patients in a pragmatic trial. Journal of Alternative and Complementary Medicine, 2012. 18(11): p. 1021-1027. |
| 260 | Stuardi, T. and H. MacPherson, Acupuncture for irritable bowel syndrome: diagnosis and treatment of patients in a pragmatic trial. Journal of alternative and complementary medicine (New York, N.Y.), 2012. 18(11): p. 1021‐1027. |
| 261 | Stuardi, T. and H. MacPherson, Acupuncture for irritable bowel syndrome: diagnosis and treatment of patients in a pragmatic trial. J Altern Complement Med, 2012. 18(11): p. 1021-1027. |
| 262 | Strobele, S. and E. Nel, Functional abdominal pain - Not exclusively an adult condition. South African Gastroenterology Review, 2013. 11(1): p. 33-36. |
| 263 | Stones, R.W. and C. Price, Health services for women with chronic pelvic pain. Journal of the Royal Society of Medicine, 2002. 95(11): p. 531-535. |
| 264 | Stone, J.A.M., The status of acupuncture and oriental medicine in the United States. Chinese Journal of Integrative Medicine, 2014. 20(4): p. 243-249. |
| 265 | Staud, R. and D.D. Price, Mechanisms of acupuncture analgesia for clinical and experimental pain. EXPERT REVIEW OF NEUROTHERAPEUTICS, 2006. 6(5): p. 661-667. |
| 266 | Staud, R., Effectiveness of CAM Therapy: Understanding the Evidence. RHEUMATIC DISEASE CLINICS OF NORTH AMERICA, 2011. 37(1): p. 9-+. |
| 267 | Stamuli, E., K. Bloor, H. MacPherson, H. Tilbrook, T. Stuardi, S. Brabyn, et al., Cost-effectiveness of acupuncture for irritable bowel syndrome: findings from an economic evaluation conducted alongside a pragmatic randomised controlled trial in primary care. BMC GASTROENTEROLOGY, 2012. 12. |
| 268 | Stamuli, E., K. Bloor, H. MacPherson, H. Tilbrook, T. Stuardi, S. Brabyn, et al., Cost-effectiveness of acupuncture for irritable bowel syndrome: Findings from an economic evaluation conducted alongside a pragmatic randomised controlled trial in primary care. BMC Gastroenterology, 2012. 12. |
| 269 | Stamuli, E., K. Bloor, H. MacPherson, H. Tilbrook, T. Stuardi, S. Brabyn, et al., Cost-effectiveness of acupuncture for irritable bowel syndrome: findings from an economic evaluation conducted alongside a pragmatic randomised controlled trial in primary care. BMC gastroenterology, 2012. 12: p. 149. |
| 270 | Stamuli, E., K. Bloor, H. MacPherson, H. Tilbrook, T. Stuardi, S. Brabyn, et al., Cost-effectiveness of acupuncture for irritable bowel syndrome: findings from an economic evaluation conducted alongside a pragmatic randomised controlled trial in primary care. BMC Gastroenterol, 2012. 12: p. 149. |
| 271 | Srinivasan, A.K., J.D. Kaye, and R. Moldwin, Myofascial dysfunction associated with chronic pelvic floor pain: Management strategies. Current Pain and Headache Reports, 2007. 11(5): p. 359-364. |
| 272 | Srinath, A.I., C. Walter, M.C. Newara, and E.M. Szigethy, Pain management in patients with inflammatory bowel disease: Insights for the clinician. Therapeutic Advances in Gastroenterology, 2012. 5(5): p. 339-357. |
| 273 | Southwell, B.R., Electro-Neuromodulation for Colonic Disorders-Review of Meta-Analyses, Systematic Reviews, and RCTs. NEUROMODULATION, 2020. 23(8): p. 1061-1081. |
| 274 | Southwell, B.R., Electro-Neuromodulation for Colonic Disorders—Review of Meta-Analyses, Systematic Reviews, and RCTs. Neuromodulation, 2020. 23(8): p. 1061-1081. |
| 275 | Southwell, B.R., Electro-Neuromodulation for Colonic Disorders-Review of Meta-Analyses, Systematic Reviews, and RCTs. Neuromodulation, 2020. 23(8): p. 1061-1081. |
| 276 | Song, S.Y., Y.J. Wang, D.S. Wang, and J.Y. Chai, [Effects of oculo-acupuncture therapy on colonic serotonin reuptake transporter expression in rats with irritable bowel syndrome]. Zhen ci yan jiu = Acupuncture research / [Zhongguo yi xue ke xue yuan Yi xue qing bao yan jiu suo bian ji], 2011. 36(2): p. 101-104, 115. |
| 277 | Song, S.Y., Y.J. Wang, D.S. Wang, and J.Y. Chai, [Effects of oculo-acupuncture therapy on colonic serotonin reuptake transporter expression in rats with irritable bowel syndrome]. Zhen Ci Yan Jiu, 2011. 36(2): p. 101-104, 115. |
| 278 | Sohn, L., D. Belvis, and S. Suresh, Neuropathic pain or somatoform disorder: Is the verdict in the differential block? Paediatric Anaesthesia, 2009. 19(6): p. 630-631. |
| 279 | Şoban, S., E. Akbal, S. Kkl, G. Kkl, M.A. Ulaşl, S. Erke, et al., Clinical trial: Transcutaneous interferential electrical stimulation in individuals with irritable bowel syndrome-a prospective double-blind randomized study. Digestion, 2012. 86(2): p. 86-93. |
| 280 | Soban, S., E. Akbal, S. Kkl, G. Kkl, M.A. Ulasl, S. Erke, et al., Clinical trial: transcutaneous interferential electrical stimulation in individuals with irritable bowel syndrome-a prospective double-blind randomized study. Digestion, 2012. 86(2): p. 86‐93. |
| 281 | Smith, G.D., D.T. Steinke, M. Kinnear, K.I. Penny, N. Pathmanathan, and I.D. Penman, A comparison of irritable bowel syndrome patients managed in primary and secondary care: The episode IBS study. British Journal of General Practice, 2004. 54(504): p. 503-507. |
| 282 | Sipaviciute, A., T. Aukstikalnis, N.E. Samalavicius, and A. Dulskas, The Role of Traditional Acupuncture in Patients with Fecal Incontinence-Mini-Review. INTERNATIONAL JOURNAL OF ENVIRONMENTAL RESEARCH AND PUBLIC HEALTH, 2021. 18(4). |
| 283 | Sipaviciute, A., T. Aukstikalnis, N.E. Samalavicius, and A. Dulskas, The role of traditional acupuncture in patients with fecal incontinence—mini-review. International Journal of Environmental Research and Public Health, 2021. 18(4): p. 1-15. |
| 284 | Sipaviciute, A., T. Aukstikalnis, N.E. Samalavicius, and A. Dulskas, The Role of Traditional Acupuncture in Patients with Fecal Incontinence-Mini-Review. Int J Environ Res Public Health, 2021. 18(4). |
| 285 | Singh, B.B., R. Khorsan, and S.P. Vinjamury, Influence of comorbidities on improvement of fibromyalgia symptoms when treated with acupuncture: a short report. Alternative therapies in health and medicine, 2008. 14(5): p. 24-25. |
| 286 | Singh, B.B., R. Khorsan, and S.P. Vinjamury, Influence of comorbidities on improvement of fibromyalgia symptoms when treated with acupuncture: a short report. Altern Ther Health Med, 2008. 14(5): p. 24-25. |
| 287 | Siedentopf, F., P. Weijenborg, M. Engman, B. Maier, A. Cagnacci, S. Mimoun, et al., ISPOG European Consensus Statement-chronic pelvic pain in women (short version). Journal of Psychosomatic Obstetrics and Gynecology, 2015. 36(4): p. 161-170. |
| 288 | Shuai, P., X.H. Zhou, L.X. Lao, and X.S. Li, Issues of design and statistical analysis in controlled clinical acupuncture trials: an analysis of English-language reports from Western journals. STATISTICS IN MEDICINE, 2012. 31(7): p. 606-618. |
| 289 | Showalter, K., A. Hoffmann, N. Decredico, A. Thakrar, E. Arroyo, I. Goldberg, et al., Complementary therapies for patients with systemic sclerosis. Journal of Scleroderma and Related Disorders, 2019. 4(3): p. 187-199. |
| 290 | Shi, Z.M., Y.S. Zhu, Q.X. Wang, and M.N. Lei, [Comparative study on irritable bowel syndrome treated with acupuncture and western medicine]. Zhongguo zhen jiu = Chinese acupuncture & moxibustion, 2011. 31(7): p. 607-609. |
| 291 | Shi, Z.M., Y.S. Zhu, Q.X. Wang, and M.N. Lei, Comparative study on irritable bowel syndrome treated with acupuncture and western medicine. Zhongguo zhen jiu [Chinese acupuncture & moxibustion], 2011. 31(7): p. 607‐609. |
| 292 | Shi, Z.M., Y.S. Zhu, Q.X. Wang, and M.N. Lei, [Comparative study on irritable bowel syndrome treated with acupuncture and western medicine]. Zhongguo Zhen Jiu, 2011. 31(7): p. 607-609. |
| 293 | Shi, Y.Z., K. Ye, M. Chen, X. Xie, X.Y. Fan, C.R. Xie, et al., Acupuncture for irritable bowel syndrome: Study protocol of a prospective, multicentre, registry study in real-world settings. European Journal of Integrative Medicine, 2022. 55. |
| 294 | Shi, Y.Z., Q.F. Tao, D. Qin, M. Chen, S.G. Yu, and H. Zheng, Acupuncture vs. antispasmodics in the treatment of irritable bowel syndrome: An adjusted indirect treatment comparison meta-analysis. FRONTIERS IN PHYSIOLOGY, 2022. 13. |
| 295 | Shi, Y.Z., Q.F. Tao, D. Qin, M. Chen, S.G. Yu, and H. Zheng, Acupuncture vs. antispasmodics in the treatment of irritable bowel syndrome: An adjusted indirect treatment comparison meta-analysis. Frontiers in Physiology, 2022. 13. |
| 296 | Shi, Y.Z., Q.F. Tao, D. Qin, M. Chen, S.G. Yu, and H. Zheng, Acupuncture vs. antispasmodics in the treatment of irritable bowel syndrome: An adjusted indirect treatment comparison meta-analysis. Front Physiol, 2022. 13: p. 1001978. |
| 297 | Shi, Y., Y.H. Chen, X.J. Yin, A.Q. Wang, X.K. Chen, J.H. Lu, et al., Electroacupuncture versus Moxibustion for Irritable Bowel Syndrome: A Randomized, Parallel-Controlled Trial. EVIDENCE-BASED COMPLEMENTARY AND ALTERNATIVE MEDICINE, 2015. 2015. |
| 298 | Shi, Y., Y.H. Chen, X.J. Yin, A.Q. Wang, X.K. Chen, J.H. Lu, et al., Electroacupuncture versus moxibustion for irritable bowel syndrome: A randomized, parallel-controlled trial. Evidence-based Complementary and Alternative Medicine, 2015. 2015. |
| 299 | Shi, Y., Y.H. Chen, X.J. Yin, A.Q. Wang, X.K. Chen, J.H. Lu, et al., Electroacupuncture versus Moxibustion for Irritable Bowel Syndrome: A Randomized, Parallel-Controlled Trial. Evid Based Complement Alternat Med, 2015. 2015: p. 361786. |
| 300 | Shi, Y., Y.H. Chen, X.J. Yin, A.Q. Wang, X.K. Chen, J.H. Lu, et al., Electroacupuncture versus moxibustion for irritable bowel syndrome: a randomized, parallel-controlled trial. Evidence-based complementary and alternative medicine, 2015. 2015. |
| 301 | Shi, X., Y. Hu, B. Zhang, W. Li, J.D. Chen, and F. Liu, Ameliorating effects and mechanisms of transcutaneous auricular vagal nerve stimulation on abdominal pain and constipation. JCI insight, 2021. 6(14). |
| 302 | Shi, J.Y., J.A. Paredes Mogica, and E.J.B. De, Non-Surgical Management of Chronic Pelvic Pain in Females. Current Urology Reports, 2022. 23(10): p. 245-254. |
| 303 | Shi, H., Y. Niu, Q. Huang, Z.M. Yang, and L. Yang, Diarrhea-predominant irritable bowel syndrome of spleen deficiency and damp excess treated with fire needling therapy with filiform needle and acupoint application therapy: a randomized controlled trial. Zhongguo zhen jiu = Chinese acupuncture & moxibustion, 2021. 41(9): p. 984-990. |
| 304 | Shi, H., Y. Niu, Q. Huang, Z.M. Yang, and L. Yang, Diarrhea-predominant irritable bowel syndrome of spleen deficiency and damp excess treated with fire needling therapy with filiform needle and acupoint application therapy: a randomized controlled trial. Zhongguo zhen jiu [Chinese acupuncture & moxibustion], 2021. 41(9): p. 984‐990. |
| 305 | Shi, H., Y. Niu, Q. Huang, Z.M. Yang, and L. Yang, [Diarrhea-predominant irritable bowel syndrome of spleen deficiency and damp excess treated with fire needling therapy with filiform needle and acupoint application therapy: a randomized controlled trial]. Zhongguo Zhen Jiu, 2021. 41(9): p. 984-990. |
| 306 | Shen, Y.H.A. and R. Nahas, Complementary and alternative medicine for treatment of irritable bowel syndrome. CANADIAN FAMILY PHYSICIAN, 2009. 55(2): p. 143-148. |
| 307 | Shen, Y.H.A. and R. Nahas, Complementary and alternative medicine for treatment of irritable bowel syndrome. Canadian Family Physician, 2009. 55(2): p. 143-148. |
| 308 | Shen, Y.H. and R. Nahas, Complementary and alternative medicine for treatment of irritable bowel syndrome. Can Fam Physician, 2009. 55(2): p. 143-148. |
| 309 | Shen, J.H., Y.M. Ye, K.X. Zhu, and S.S. Li, Acupuncture for diarrhea-predominant irritable bowel syndrome: A randomized control study. World Journal of Acupuncture - Moxibustion, 2022. 32(2): p. 123-130. |
| 310 | Shen, J.H., Y.M. Ye, K.X. Zhu, and S.S. Li, Acupuncture for diarrhea-predominant irritable bowel syndrome: a randomized control study. World journal of acupuncture - moxibustion, 2022. 32(2): p. 123‐130. |
| 311 | Shaver, J.L., Fibromyalgia syndrome in women. Nursing Clinics of North America, 2004. 39(1): p. 195-204. |
| 312 | Shakeel, M., A. Trinidade, and K.W. Ah-See, Complementary and alternative medicine use by otolaryngology patients: A paradigm for practitioners in all surgical specialties. European Archives of Oto-Rhino-Laryngology, 2010. 267(6): p. 961-971. |
| 313 | Schneider, A., C. Weiland, P. Enck, S. Joos, K. Streitberger, C. Maser-Gluth, et al., Neuroendocrinological effects of acupuncture treatment in patients with irritable bowel syndrome. Complementary Therapies in Medicine, 2007. 15(4): p. 255-263. |
| 314 | Schneider, A., C. Weiland, P. Enck, S. Joos, K. Streitberger, C. Maser-Gluth, et al., Neuroendocrinological effects of acupuncture treatment in patients with irritable bowel syndrome. Complementary therapies in medicine, 2007. 15(4): p. 255‐263. |
| 315 | Schneider, A., C. Weiland, P. Enck, S. Joos, K. Streitberger, C. Maser-Gluth, et al., Neuroendocrinological effects of acupuncture treatment in patients with irritable bowel syndrome. Complement Ther Med, 2007. 15(4): p. 255-263. |
| 316 | Schneider, A., K. Streitberger, and S. Joos, Acupuncture treatment in gastrointestinal disease: A systematic review. WORLD JOURNAL OF GASTROENTEROLOGY, 2007. 13(25): p. 3417-3424. |
| 317 | Schneider, A., K. Streitberger, and S. Joos, Acupuncture treatment in gastrointestinal diseases: A systematic review. World Journal of Gastroenterology, 2007. 13(25): p. 3417-3424. |
| 318 | Schneider, A., K. Streitberger, and S. Joos, Acupuncture treatment in gastrointestinal diseases: a systematic review. World J Gastroenterol, 2007. 13(25): p. 3417-3424. |
| 319 | Schneider, A., P. Enck, K. Streitberger, C. Weiland, S. Bagheri, S. Witte, et al., Acupuncture treatment in irritable bowel syndrome. Gut, 2006. 55(5): p. 649-654. |
| 320 | Schneider, A., P. Enck, K. Streitberger, C. Weiland, S. Bagheri, S. Witte, et al., Acupuncture treatment in irritable bowel syndrome. Gut, 2006. 55(5): p. 649‐654. |
| 321 | Schneider, A., P. Enck, K. Streitberger, C. Weiland, S. Bagheri, S. Witte, et al., Acupuncture treatment in irritable bowel syndrome. Gut, 2006. 55(5): p. 649-654. |
| 322 | Schneider, A., P. Enck, K. Streitberger, S. Joos, C. Weiland, S. Bagheri, et al., Specific physiological and unspecific psychological effects during acupuncture treatment in patients with irritable bowel syndrome: Results of a randomised controlled trial. Deutsche Zeitschrift fur Akupunktur, 2008. 51(1): p. 8-16. |
| 323 | Schneider, A., P. Enck, K. Streitberger, S. Joos, C. Weiland, S. Bagheri, et al., Specific physiological and unspecific psychological effects during acupuncture treatment in patients with irritable bowel syndrome: Results of a randomised controlled trial. Revista Internacional de Acupuntura, 2008. 2(3): p. 146-154. |
| 324 | Schneider, A., P. Enck, K. Streitberger, S. Joos, C. Weiland, S. Bagheri, et al., Specific physiological and unspecific psychological effects during acupuncture treatment in patients with irritable bowel syndrome: results of a randomised controlled trial. Deutsche zeitschrift fur akupunktur, 2008. 51(1): p. 8‐16. |
| 325 | Schneider, A., P. Enck, K. Streitberger, S. Joos, C. Weiland, S. Bagheri, et al., Specific physiological and unspecific psychological effects during acupuncture treatment in patients with irritable bowel syndrome: results of a randomised controlled trial. Revista internacional de acupuntura, 2008. 2(3): p. 146‐154. |
| 326 | Schneider, A., P. Enck, and K. Streitberger, Stratification by sex and subgroup is necessary for RCT on IBS - Reply. GUT, 2006. 55(5): p. 744-744. |
| 327 | Schneider, A., P. Enck, and K. Streitberger, Authors' reply [2]. Gut, 2006. 55(5): p. 744. |
| 328 | Scaciota, A.C.L., D. Matos, M.M.B. Rosa, M.E.S. Colovati, E.F.B.C. Bellotto, and A.L.C. Martimbianco, Interventions for the treatment of irritable bowel syndrome: A review of cochrane systematic reviews. Arquivos de Gastroenterologia, 2021. 58(1): p. 120-126. |
| 329 | Sarzi-Puttini, P., F. Atzeni, M. Di Franco, D. Buskila, A. Alciati, C. Giacomelli, et al., Dysfunctional syndromes and fibromyalgia: A 2012 critical digest. Clinical and Experimental Rheumatology, 2012. 30(SUPPL.74): p. S143-S151. |
| 330 | Saps, M. and C. Di Lorenzo, Probiotics for abdominal pain disorders in children - Safe to use but are they helpful?: Commentary. Nature Clinical Practice Gastroenterology and Hepatology, 2007. 4(8): p. 430-431. |
| 331 | Santucci, N.R., M. Saps, and M.A. van Tilburg, New advances in the treatment of paediatric functional abdominal pain disorders. The Lancet Gastroenterology and Hepatology, 2020. 5(3): p. 316-328. |
| 332 | Santucci, N.R., M. Saps, and M.A. van Tilburg, New advances in the treatment of paediatric functional abdominal pain disorders. Lancet Gastroenterol Hepatol, 2020. 5(3): p. 316-328. |
| 333 | Sandhu, B.K. and S.P. Paul, Irritable bowel syndrome in children: Pathogenesis, diagnosis and evidence-based treatment. World Journal of Gastroenterology, 2014. 20(20): p. 6013-6023. |
| 334 | Sack, K., The pain that never heals: Diagnosing and managing patients with fibromyalgia. Advanced Studies in Medicine, 2004. 4(8): p. 401-408. |
| 335 | Ruiz, C., A. Yousefi, R. Hu, C. McLaughlin, E. Kokkoutou, and L. Conboy, Using machine learning techniques to understand the complexity of pain experience with acupuncture treatment. Journal of Alternative and Complementary Medicine, 2021. 27(11): p. A6-A7. |
| 336 | Rudolph, C.D. and A. Miranda, Treatment Options for Functional Abdominal Pain. Pediatric Annals, 2004. 33(2): p. 105-112. |
| 337 | Reynolds, J.A., J.M. Bland, and H. MacPherson, Acupuncture for irritable bowel syndrome - an exploratory randomised controlled trial. ACUPUNCTURE IN MEDICINE, 2008. 26(1): p. 8-16. |
| 338 | Reynolds, J.A., J.M. Bland, and H. MacPherson, Acupuncture for irritable bowel syndrome - An exploratory randomised controlled trial. Acupuncture in Medicine, 2008. 26(1): p. 8-16. |
| 339 | Reynolds, J.A., J.M. Bland, and H. MacPherson, Acupuncture for irritable bowel syndrome an exploratory randomised controlled trial. Acupuncture in medicine, 2008. 26(1): p. 8‐16. |
| 340 | Reynolds, J.A., J.M. Bland, and H. MacPherson, Acupuncture for irritable bowel syndrome an exploratory randomised controlled trial. Acupunct Med, 2008. 26(1): p. 8-16. |
| 341 | Rao, V.L., A.S. Cifu, and L.W. Yang, Pharmacologic management of irritable bowel syndrome. JAMA - Journal of the American Medical Association, 2015. 314(24): p. 2684-2685. |
| 342 | Rafiei, R., M. Ataie, M.A. Ramezani, A. Etemadi, B. Ataei, H. Nikyar, et al., A new acupuncture method for management of irritable bowel syndrome: A randomized double blind clinical trial. JOURNAL OF RESEARCH IN MEDICAL SCIENCES, 2014. 19(10): p. 913-917. |
| 343 | Rafiei, R., M. Ataie, M.A. Ramezani, A. Etemadi, B. Ataei, H. Nikyar, et al., A new acupuncture method for management of irritable bowel syndrome: A randomized double blind clinical trial. Journal of Research in Medical Sciences, 2014. 19(10): p. 913-917. |
| 344 | Rafiei, R., M. Ataie, M.A. Ramezani, A. Etemadi, B. Ataei, H. Nikyar, et al., A new acupuncture method for management of irritable bowel syndrome: a randomized double blind clinical trial. Journal of research in medical sciences, 2014. 19(10): p. 913‐917. |
| 345 | Rafiei, R., M. Ataie, M.A. Ramezani, A. Etemadi, B. Ataei, H. Nikyar, et al., A new acupuncture method for management of irritable bowel syndrome: A randomized double blind clinical trial. J Res Med Sci, 2014. 19(10): p. 913-917. |
| 346 | Rabitti, S., C.M. Giovanardi, and D. Colussi, Acupuncture and Related Therapies for the Treatment of Gastrointestinal Diseases. Journal of Clinical Gastroenterology, 2021. 55(3): p. 207-217. |
| 347 | Quilty, M. and L. Conboy, Relationship of treatment beliefs to subject blinding: The case of a placebo-controlled RCT in Irritable Bowel Syndrome. BMC Complementary and Alternative Medicine, 2012. 12. |
| 348 | Quilty, M. and L. Conboy, Relationship of treatment beliefs to subject blinding: the case of a placebo-controlled RCT in Irritable Bowel Syndrome. BMC complementary and alternative medicine, 2012. 12. |
| 349 | Quilty, M. and L. Conboy, Relationship of treatment beliefs to subject blinding: the case of a placebo-controlled RCT in Irritable Bowel Syndrome. BMC complementary and alternative medicine, 2012. 12. |
| 350 | Qu, L., L. Xing, W. Norman, H. Chen, and S. Gao, Irritable bowel syndrome treated by traditional Chinese spinal orthopedic manipulation. Journal of traditional chinese medicine = chung i tsa chih ying wen pan, 2012. 32(4): p. 565‐570. |
| 351 | Qin, Z., B. Li, J. Wu, J. Tian, S. Xie, Z. Mao, et al., Acupuncture for chronic diarrhea in adults protocol for a systematic review. Medicine (United States), 2017. 96(4). |
| 352 | Qin, Z., B. Li, J. Wu, J. Tian, S. Xie, Z. Mao, et al., Acupuncture for chronic diarrhea in adults: Protocol for a systematic review. Medicine (Baltimore), 2017. 96(4): p. e5952. |
| 353 | Qin, Y., W. Yi, S. Lin, C. Yang, and Z. Zhuang, Clinical effect of abdominal acupuncture for diarrhea irritable bowel syndrome. Zhongguo zhen jiu = Chinese acupuncture & moxibustion, 2017. 37(12): p. 1265-1268. |
| 354 | Qin, Y., W. Yi, S. Lin, C. Yang, and Z. Zhuang, Clinical effect of abdominal acupuncture for diarrhea irritable bowel syndrome. Zhongguo zhen jiu [Chinese acupuncture & moxibustion], 2017. 37(12): p. 1265‐1268. |
| 355 | Qin, Y., W. Yi, S. Lin, C. Yang, and Z. Zhuang, [Clinical effect of abdominal acupuncture for diarrhea irritable bowel syndrome]. Zhongguo Zhen Jiu, 2017. 37(12): p. 1265-1268. |
| 356 | Qin, Y., M.W. Guo, Y. Lan, Y.F. Wang, S. Wang, M.X. Ji, et al., Effect of electroacupuncture of "Hegu" (LI4) and "Zusanli" (ST36) on intestinal sensitivity and motility in irritable bowel syndrome rats. Zhen ci yan jiu = Acupuncture research, 2020. 45(4): p. 293-298. |
| 357 | Qin, Y., M.W. Guo, Y. Lan, Y.F. Wang, S. Wang, M.X. Ji, et al., [Effect of electroacupuncture of "Hegu" (LI4) and "Zusanli" (ST36) on intestinal sensitivity and motility in irritable bowel syndrome rats]. Zhen Ci Yan Jiu, 2020. 45(4): p. 293-298. |
| 358 | Qi, Q., H. Wu, X. Jin, D. Jin, Y. Wang, C. Wang, et al., Effect of moxibustion on the expression of GDNF and its receptor GFRα3 in the colon and spinal cord of rats with irritable bowel syndrome. Acupuncture in medicine : journal of the British Medical Acupuncture Society, 2019. 37(4): p. 244-251. |
| 359 | Qi, Q., H. Wu, X. Jin, D. Jin, Y. Wang, C. Wang, et al., Effect of moxibustion on the expression of GDNF and its receptor GFRα3 in the colon and spinal cord of rats with irritable bowel syndrome. Acupunct Med, 2019. 37(4): p. 244-251. |
| 360 | Qi, L.Y., J.W. Yang, S.Y. Yan, J.F. Tu, Y.F. She, Y. Li, et al., Acupuncture for the Treatment of Diarrhea-Predominant Irritable Bowel Syndrome: A Pilot Randomized Clinical Trial. JAMA Network Open, 2022. 5(12): p. E2248817. |
| 361 | Qi, L.Y., J.W. Yang, S.Y. Yan, J.F. Tu, Y.F. She, Y. Li, et al., Acupuncture for the Treatment of Diarrhea-Predominant Irritable Bowel Syndrome: a Pilot Randomized Clinical Trial. JAMA network open, 2022. 5(12): p. e2248817. |
| 362 | Qi, L.Y., J.W. Yang, S.Y. Yan, J.F. Tu, Y.F. She, Y. Li, et al., Acupuncture for the Treatment of Diarrhea-Predominant Irritable Bowel Syndrome: A Pilot Randomized Clinical Trial. JAMA Netw Open, 2022. 5(12): p. e2248817. |
| 363 | Qi, L.Y., J.W. Yang, S.Y. Yan, Y.F. She, H. Hu, Y. Li, et al., Effect of acupuncture for diarrhea-predominant irritable bowel syndrome: study protocol for a randomized clinical trial. TRIALS, 2022. 23(1). |
| 364 | Qi, L.Y., J.W. Yang, S.Y. Yan, Y.F. She, H. Hu, Y. Li, et al., Effect of acupuncture for diarrhea-predominant irritable bowel syndrome: study protocol for a randomized clinical trial. Trials, 2022. 23(1). |
| 365 | Qi, L.Y., J.W. Yang, S.Y. Yan, Y.F. She, H. Hu, Y. Li, et al., Effect of acupuncture for diarrhea-predominant irritable bowel syndrome: study protocol for a randomized clinical trial. Trials, 2022. 23(1): p. 711. |
| 366 | Qi, L.Y., J.W. Yang, S.Y. Yan, Y.F. She, H. Hu, Y. Li, et al., Effect of acupuncture for diarrhea-predominant irritable bowel syndrome: study protocol for a randomized clinical trial. Trials, 2022. 23(1): p. 711. |
| 367 | Qi, L.Y., Y. Wang, L.Q. Wang, Y.F. She, G.X. Shi, Y. Li, et al., Acupuncture for the treatment of diarrheal-predominant irritable bowel syndrome: study protocol for a pilot randomized controlled trial (vol 22 ,page 253,year 2021). TRIALS, 2023. 24(1). |
| 368 | Qi, L.Y., Y. Wang, L.Q. Wang, Y.F. She, G.X. Shi, Y. Li, et al., Correction: Acupuncture for the treatment of diarrheal-predominant irritable bowel syndrome: study protocol for a pilot randomized controlled trial (Trials, (2021), 22, 1, (253), 10.1186/s13063-021-05211-x). Trials, 2023. 24(1). |
| 369 | Qi, L.Y., Y. Wang, L.Q. Wang, Y.F. She, G.X. Shi, Y. Li, et al., Acupuncture for the treatment of diarrheal-predominant irritable bowel syndrome: study protocol for a pilot randomized controlled trial. TRIALS, 2021. 22(1). |
| 370 | Qi, L.Y., Y. Wang, L.Q. Wang, Y.F. She, G.X. Shi, Y. Li, et al., Acupuncture for the treatment of diarrheal-predominant irritable bowel syndrome: study protocol for a pilot randomized controlled trial. Trials, 2021. 22(1). |
| 371 | Qi, L.Y., Y. Wang, L.Q. Wang, Y.F. She, G.X. Shi, Y. Li, et al., Acupuncture for the treatment of diarrheal-predominant irritable bowel syndrome: study protocol for a pilot randomized controlled trial. Trials, 2021. 22(1): p. 253. |
| 372 | Qi, L.Y., Y. Wang, L.Q. Wang, Y.F. She, G.X. Shi, Y. Li, et al., Correction: acupuncture for the treatment of diarrheal-predominant irritable bowel syndrome: study protocol for a pilot randomized controlled trial (Trials, (2021), 22, 1, (253), 10.1186/s13063-021-05211-x). Trials, 2023. 24(1). |
| 373 | Qi, L.Y., Y. Wang, L.Q. Wang, Y.F. She, G.X. Shi, Y. Li, et al., Acupuncture for the treatment of diarrheal-predominant irritable bowel syndrome: study protocol for a pilot randomized controlled trial. Trials, 2021. 22(1): p. 253. |
| 374 | Puttini, P.S., W. Häuser, K. Lawson, and H. Sprott, Topical seminar summary: Fibromyalgia syndrome. European Journal of Pain, 2009. 13: p. S5-S6. |
| 375 | Pontari, M. and L. Giusto, New developments in the diagnosis and treatment of chronic prostatitis/chronic pelvic pain syndrome. Current Opinion in Urology, 2013. 23(6): p. 565-569. |
| 376 | Podovei, M. and B. Kuo, Irritable bowel syndrome: A practical review. Southern Medical Journal, 2006. 99(11): p. 1235-1243. |
| 377 | Pisipati, S., B.A. Connor, and M.S. Riddle, Updates on the epidemiology, pathogenesis, diagnosis, and management of postinfectious irritable bowel syndrome. Curr Opin Infect Dis, 2020. 33(5): p. 411-418. |
| 378 | Piche, T., M. Dapoigny, C. Bouteloup, P. Chassagne, B. Coffin, V. Desfourneaux, et al., Recommendations for clinical practice in the management and treatment of chronic constipation of adults. Gastroenterologie Clinique et Biologique, 2007. 31(2): p. 125-135. |
| 379 | Petzke, F., CNS processing of pain in functional somatic syndromes. Schmerz, 2010. 24(2): p. 146-155. |
| 380 | Pettit, J. and R. Glickman-Simon, Osteopathic Manipulative Therapy for Preterm Infants, Acupuncture for Menopausal Symptoms, Mindfulness-Based Stress Reduction for Chronic Low Back Pain, Chocolate for Ischemic Heart Disease, Berberine for Irritable Bowel Syndrome. Explore, 2016. 12(5): p. 388-392. |
| 381 | Perez, M.E. and N.N. Youssef, Dyspepsia in childhood and adolescence: Insights and treatment considerations. Current Gastroenterology Reports, 2007. 9(6): p. 447-455. |
| 382 | Peng, Y.H., H. You, X.M. Chen, Y.B. Chen, Y.L. Yang, J.P. Huang, et al., Effect of electroacupuncture at homotopic and heterotopic acupoints on abdominal pain in patients with irritable bowel syndrome: study protocol for a randomized controlled trial. TRIALS, 2018. 19. |
| 383 | Peng, Y., H. You, X. Chen, Y. Chen, Y. Yang, J. Huang, et al., Effect of electroacupuncture at homotopic and heterotopic acupoints on abdominal pain in patients with irritable bowel syndrome: Study protocol for a randomized controlled trial. Trials, 2018. 19(1). |
| 384 | Peng, Y., H. You, X. Chen, Y. Chen, Y. Yang, J. Huang, et al., Effect of electroacupuncture at homotopic and heterotopic acupoints on abdominal pain in patients with irritable bowel syndrome: study protocol for a randomized controlled trial 11 Medical and Health Sciences 1103 Clinical Sciences 11 Medical and Health Sciences 1109 Neurosciences. Trials, 2018. 19(1). |
| 385 | Peng, Y., H. You, X. Chen, Y. Chen, Y. Yang, J. Huang, et al., Effect of electroacupuncture at homotopic and heterotopic acupoints on abdominal pain in patients with irritable bowel syndrome: study protocol for a randomized controlled trial. Trials, 2018. 19(1): p. 559. |
| 386 | Pei, L.X., X.C. Zhang, J.H. Sun, H. Geng, and X.L. Wu, [Meta analysis of acupuncture-moxibustion in treatment of irritable bowel syndrome]. Zhongguo zhen jiu = Chinese acupuncture & moxibustion, 2012. 32(10): p. 957-960. |
| 387 | Pei, L.X., X.C. Zhang, J.H. Sun, H. Geng, and X.L. Wu, [Meta analysis of acupuncture-moxibustion in treatment of irritable bowel syndrome]. Zhongguo Zhen Jiu, 2012. 32(10): p. 957-960. |
| 388 | Pei, L.X., W. Zhang, Y.F. Song, H. Geng, L. Chen, X.L. Wu, et al., Electroacupuncture of "Tianshu" (ST 25) Suppresses Visceral Pain Possibly by Down-regulating Mast Cell Activation, and Tryptase and SP Expression in Rats with Post-infectious Irritable Bowel Syndrome. Zhen ci yan jiu = Acupuncture research, 2018. 43(7): p. 419-423. |
| 389 | Pei, L.X., W. Zhang, Y.F. Song, H. Geng, L. Chen, X.L. Wu, et al., [Electroacupuncture of "Tianshu" (ST 25) Suppresses Visceral Pain Possibly by Down-regulating Mast Cell Activation, and Tryptase and SP Expression in Rats with Post-infectious Irritable Bowel Syndrome]. Zhen Ci Yan Jiu, 2018. 43(7): p. 419-423. |
| 390 | Pei, L.X., H. Geng, J. Guo, G.H. Yang, L. Wang, R.R. Shen, et al., Effect of Acupuncture in Patients With Irritable Bowel Syndrome: A Randomized Controlled Trial. MAYO CLINIC PROCEEDINGS, 2020. 95(8): p. 1671-1683. |
| 391 | Pei, L.X., H. Geng, H. Chen, X.L. Wu, L. Chen, J.L. Zhou, et al., Acupuncture for irritable bowel syndrome: study protocol for a multicenter randomized controlled trial. TRIALS, 2018. 19. |
| 392 | Pei, L.X., H. Geng, H. Chen, X.L. Wu, L. Chen, J.L. Zhou, et al., Acupuncture for irritable bowel syndrome: Study protocol for a multicenter randomized controlled trial. Trials, 2018. 19(1). |
| 393 | Pei, L.X., H. Geng, H. Chen, X.L. Wu, L. Chen, J.L. Zhou, et al., Acupuncture for irritable bowel syndrome: study protocol for a multicenter randomized controlled trial. Trials, 2018. 19(1): p. 529. |
| 394 | Pei, L.X., H. Geng, H. Chen, X.L. Wu, L. Chen, J.L. Zhou, et al., Acupuncture for irritable bowel syndrome: study protocol for a multicenter randomized controlled trial. Trials, 2018. 19(1): p. 529. |
| 395 | Pei, L.X., H. Geng, H. Chen, X.L. Wu, L. Chen, J.L. Zhou, et al., Acupuncture for irritable bowel syndrome: study protocol for a multicenter randomized controlled trial 11 Medical and Health Sciences 1103 Clinical Sciences 11 Medical and Health Sciences 1104 Complementary and Alternative Medicine. Trials, 2018. 19(1). |
| 396 | Pei, L.X., H. Geng, H. Chen, X.L. Wu, L. Chen, J.L. Zhou, et al., Acupuncture for irritable bowel syndrome: study protocol for a multicenter randomized controlled trial. Trials, 2018. 19(1): p. 529. |
| 397 | Pei, L.X., H. Chen, J. Guo, L. Chen, X.L. Wu, W.L. Xu, et al., Effect of acupuncture and its influence on visceral hypersensitivity in IBS-D patients: Study protocol for a randomized controlled trial. MEDICINE, 2018. 97(21). |
| 398 | Pei, L., L. Zhu, J. Sun, X. Wu, and L. Chen, Constipation predominant irritable bowel syndrome treated with acupuncture for regulating the mind and strengthening the spleen: a randomized controlled trial. Zhongguo zhen jiu = Chinese acupuncture & moxibustion, 2015. 35(11): p. 1095-1098. |
| 399 | Pei, L., L. Zhu, J. Sun, X. Wu, and L. Chen, Constipation predominant irritable bowel syndrome treated with acupuncture for regulating the mind and strengthening the spleen: a randomized controlled trial. Zhongguo zhen jiu [Chinese acupuncture & moxibustion], 2015. 35(11): p. 1095‐1098. |
| 400 | Pei, L., L. Zhu, J. Sun, X. Wu, and L. Chen, [Constipation predominant irritable bowel syndrome treated with acupuncture for regulating the mind and strengthening the spleen: a randomized controlled trial]. Zhongguo Zhen Jiu, 2015. 35(11): p. 1095-1098. |
| 401 | Pei, L., H. Geng, J. Guo, G. Yang, L. Wang, R. Shen, et al., Effect of Acupuncture in Patients With Irritable Bowel Syndrome: A Randomized Controlled Trial. Mayo Clinic Proceedings, 2020. 95(8): p. 1671-1683. |
| 402 | Pei, L., H. Geng, J. Guo, G. Yang, L. Wang, R. Shen, et al., Effect of Acupuncture in Patients With Irritable Bowel Syndrome: A Randomized Controlled Trial. Mayo Clin Proc, 2020. 95(8): p. 1671-1683. |
| 403 | Pei, L., H. Geng, J. Guo, G. Yang, L. Wang, R. Shen, et al., Effect of Acupuncture in Patients With Irritable Bowel Syndrome: a Randomized Controlled Trial. Mayo Clinic proceedings, 2020. 95(8): p. 1671‐1683. |
| 404 | Pei, L., H. Chen, J. Guo, L. Chen, X. Wu, W. Xu, et al., Effect of acupuncture and its influence on visceral hypersensitivity in IBS-D patients Study protocol for a randomized controlled trial. Medicine (United States), 2018. 97(21). |
| 405 | Pei, L., H. Chen, J. Guo, L. Chen, X. Wu, W. Xu, et al., Effect of acupuncture and its influence on visceral hypersensitivity in IBS-D patients: study protocol for a randomized controlled trial. Medicine, 2018. 97(21): p. e10877. |
| 406 | Pei, L., H. Chen, J. Guo, L. Chen, X. Wu, W. Xu, et al., Effect of acupuncture and its influence on visceral hypersensitivity in IBS-D patients: Study protocol for a randomized controlled trial. Medicine (Baltimore), 2018. 97(21): p. e10877. |
| 407 | Paul, S.P. and D. Basude, Non-pharmacological management of abdominal pain-related functional gastrointestinal disorders in children. World Journal of Pediatrics, 2016. 12(4): p. 389-398. |
| 408 | Patel, N.K., E. Kusema, M.D. Crowell, and B.E. Lacy, The placebo response in complementary and alternative medicine trials for functional dyspepsia. American Journal of Gastroenterology, 2017. 112: p. S249-S250. |
| 409 | Patel, M., I. Urits, A.D. Kaye, and O. Viswanath, The role of acupuncture in the treatment of chronic pain. BEST PRACTICE & RESEARCH-CLINICAL ANAESTHESIOLOGY, 2020. 34(3): p. 603-616. |
| 410 | Passos, M.C.F., A.J. Lembo, L.A. Conboy, T.J. Kaptchuk, J.M. Kelly, M.T. Quilty, et al., Adequate Relief in a Treatment Trial With IBS Patients: A Prospective Assessment. AMERICAN JOURNAL OF GASTROENTEROLOGY, 2009. 104(4): p. 912-919. |
| 411 | Passos, M.C.F., A.J. Lembo, L.A. Conboy, T.J. Kaptchuk, J.M. Kelly, M.T. Quilty, et al., Adequate relief in a treatment trial with IBS patients: A prospective assessment. American Journal of Gastroenterology, 2009. 104(4): p. 912-919. |
| 412 | Passos, M.C.F., A.J. Lembo, L.A. Conboy, T.J. Kaptchuk, J.M. Kelly, M.T. Quilty, et al., Adequate relief in a treatment trial with IBS patients: A prospective assessment. American Journal of Gastroenterology, 2009. 104(4): p. X912-919. |
| 413 | Passos, M.C., A.J. Lembo, L.A. Conboy, T.J. Kaptchuk, J.M. Kelly, M.T. Quilty, et al., Adequate relief in a treatment trial with IBS patients: a prospective assessment. American journal of gastroenterology, 2009. 104(4): p. 912‐919. |
| 414 | Passos, M.C., A.J. Lembo, L.A. Conboy, T.J. Kaptchuk, J.M. Kelly, M.T. Quilty, et al., Adequate relief in a treatment trial with IBS patients: a prospective assessment. Am J Gastroenterol, 2009. 104(4): p. 912-919. |
| 415 | Park, J.Y., Y.K. Kim, S.Y. Kim, H. Lee, C.J. Choi, Y. Chae, et al., Acupuncture modulates brain neural activity in patients: a systematic review and meta-analysis. Oriental Pharmacy and Experimental Medicine, 2017. 17(2): p. 111-126. |
| 416 | Park, J.W., B.H. Lee, and H. Lee, Moxibustion in the management of irritable bowel syndrome: systematic review and meta-analysis. BMC COMPLEMENTARY AND ALTERNATIVE MEDICINE, 2013. 13. |
| 417 | Park, J.W., B.H. Lee, and H. Lee, Moxibustion in the management of irritable bowel syndrome: Systematic review and meta-analysis. BMC Complementary and Alternative Medicine, 2013. 13. |
| 418 | Park, J.W., B.H. Lee, and H. Lee, Moxibustion in the management of irritable bowel syndrome: systematic review and meta-analysis. BMC Complement Altern Med, 2013. 13: p. 247. |
| 419 | Park, J., K. Linde, E. Manheimer, A. Molsberger, K. Sherman, C. Smith, et al., The Status and Future of Acupuncture Clinical Research. JOURNAL OF ALTERNATIVE AND COMPLEMENTARY MEDICINE, 2008. 14(7): p. 871-881. |
| 420 | Park, H.J. and C. Cha, The Effect of Korean Hand Acupuncture on Young, Single Korean Students With Irritable Bowel Syndrome. GASTROENTEROLOGY NURSING, 2012. 35(6): p. 403-414. |
| 421 | Park, H.J. and C. Cha, The effect of Korean hand acupuncture on young, single Korean students with irritable bowel syndrome. Gastroenterology nursing : the official journal of the Society of Gastroenterology Nurses and Associates, 2012. 35(6): p. 403-414. |
| 422 | Park, H.J. and C. Cha, The effect of Korean hand acupuncture on young, single Korean students with irritable bowel syndrome. Gastroenterology nursing, 2012. 35(6): p. 403‐414. |
| 423 | Park, H.J. and C. Cha, The effect of Korean hand acupuncture on young, single Korean students with irritable bowel syndrome. Gastroenterol Nurs, 2012. 35(6): p. 403-414. |
| 424 | Pacheco, R.L., A. Roizenblatt, A.F.T. Góis, C.O.C. Latorraca, C.F.M.G.P. Mota, and R. Riera, What do cochrane systematic reviews say about the management of irritable bowel syndrome? Sao Paulo Medical Journal, 2019. 137(1): p. 82-91. |
| 425 | Pacheco, R.L., A. Roizenblatt, A.F.T. Góis, C.O.C. Latorraca, C. Mota, and R. Riera, What do Cochrane systematic reviews say about the management of irritable bowel syndrome? Sao Paulo Med J, 2019. 137(1): p. 82-91. |
| 426 | Overland, M.K., Dyspepsia. Medical Clinics of North America, 2014. 98(3): p. 549-564. |
| 427 | Osemene, N.I., Irritable bowel syndrome in children and adolescents. 2015. p. 46-50. |
| 428 | Olden, K.W., Review of acupuncture treatment in irritable bowel syndrome: Commentary. Evidence-Based Gastroenterology, 2006. 7(4): p. 102-103. |
| 429 | Okumo, T., Y. Takayama, K. Maruyama, M. Kato, and M. Sunagawa, Senso-Immunologic Prospects for Complex Regional Pain Syndrome Treatment. Frontiers in Immunology, 2021. 12. |
| 430 | Ntr, Functional abdominal pain (FAP) within the context of internalizing disorders in childhood; A randomized controlled cognitive-behavioural family intervention. https://trialsearch.who.int/Trial2.aspx?TrialID=NTR1613, 2009. |
| 431 | Noddin, L., M. Callahan, and B.E. Lacy, Irritable bowel syndrome and functional dyspepsia: Different diseases or a single disorder with different manifestations? MedGenMed Medscape General Medicine, 2005. 7(3). |
| 432 | Nerurkar, A., "The gut-brain axis: How to manage pain caused by this cross-talk": An overview of the symposium. Global Advances In Health and Medicine, 2015. 4(6): p. 61-64. |
| 433 | Nelson, P., G. Apte, R. Justiz, J.M. Brismeé, G. Dedrick, and P.S. Sizer, Chronic Female Pelvic Pain-Part 2: Differential Diagnosis and Management. Pain Practice, 2012. 12(2): p. 111-141. |
| 434 | Nee, J. and A. Lembo, Review Article: Current and future treatment approaches for IBS with diarrhoea (IBS-D) and IBS mixed pattern (IBS-M). ALIMENTARY PHARMACOLOGY & THERAPEUTICS, 2021. 54: p. S63-S74. |
| 435 | Nee, J. and A. Lembo, Review Article: Current and future treatment approaches for IBS with diarrhoea (IBS-D) and IBS mixed pattern (IBS-M). Alimentary Pharmacology and Therapeutics, 2021. 54(S1): p. S63-S74. |
| 436 | Nee, J. and A. Lembo, Review Article: Current and future treatment approaches for IBS with diarrhoea (IBS-D) and IBS mixed pattern (IBS-M). Aliment Pharmacol Ther, 2021. 54 Suppl 1: p. S63-s74. |
| 437 | Nct, Self- Administered Acupressure for Diarrhea Predominant Irritable Bowel Syndrome. https://clinicaltrials.gov/show/NCT05702255, 2023. |
| 438 | Nct, Effect of Transcutaneous Auricular Vagal Nerve Stimulation on Chronic Constipation. https://clinicaltrials.gov/show/NCT05723731, 2023. |
| 439 | Nct, Aerobic Exercise Verus Acupuncture on the Quality of Life in Women Suffering From Irritable Bowel Syndrome. https://clinicaltrials.gov/ct2/show/NCT05947929, 2023. |
| 440 | Nct, Transcutaneous Electric Nerve Stimulation (TENS) for Vagal Modulation. https://clinicaltrials.gov/ct2/show/NCT05987813, 2023. |
| 441 | Nct, Laser Acupuncture on Irritable Bowel Syndrome in Females. https://clinicaltrials.gov/show/NCT05757037, 2023. |
| 442 | Nct, Effect of Acupressure on Bowel Functions After Cesarean Section. https://clinicaltrials.gov/show/NCT05251818, 2022. |
| 443 | Nct, Optimization of Transcutaneous Electrical Acustimulation Modalities for Treatment of IBS-C. https://clinicaltrials.gov/show/NCT04953728, 2021. |
| 444 | Nct, Acupuncture-like Transcutaneous Electrical Nerve Stimulation on Bowel Symptom and Quality of Life in Patients With Irritable Bowel Syndrome. https://clinicaltrials.gov/show/NCT05042661, 2021. |
| 445 | Nct, Acupuncture for Female With Non-cyclic Chronic Pelvic Pain. https://clinicaltrials.gov/show/NCT04553562, 2020. |
| 446 | Nct, Auricular Vagus Nerve Stimulation For Fibromyalgia Syndrome. https://clinicaltrials.gov/show/NCT04260906, 2020. |
| 447 | Nct, Auricular Vagus Nerve Stimulation in Painful and Inflammatory Erosive Hand Osteoarthritis. https://clinicaltrials.gov/show/NCT04520516, 2020. |
| 448 | Nct, A Study of Randomized Sham-control Auricular TENS Unit Stimulation in Pediatric Functional Gastrointestinal Disorders. https://clinicaltrials.gov/show/NCT04247100, 2020. |
| 449 | Nct, Acupuncture for Irritable Bowel Syndrome Patients. https://clinicaltrials.gov/show/NCT04387383, 2020. |
| 450 | Nct, Percutaneous Electrical Nerve Field Stimulation for Adults With Irritable Bowel Syndrome. https://clinicaltrials.gov/show/NCT04428619, 2020. |
| 451 | Nct, Efficacy of Acupuncture in Refractory Irritable Bowel Syndrome. https://clinicaltrials.gov/show/NCT04276961, 2020. |
| 452 | Nct, Symptom Management for Irritable Bowel Syndrome. https://clinicaltrials.gov/show/NCT02079857, 2014. |
| 453 | Nct, Acupuncture in the Treatment of Gulf War Illness. https://clinicaltrials.gov/show/NCT01305811, 2011. |
| 454 | Nct, Trial for Quantity-Effect Relationship of Acupuncture With Two-ways Regulation to Treat Functional Enteropathy. https://clinicaltrials.gov/show/NCT01274793, 2011. |
| 455 | Nct, Effects of an Integrative Treatment Model to Reduce Anxiety and Depression in Minor Mental Health Problems and Medically Unexplained Symptoms. https://clinicaltrials.gov/show/NCT01631500, 2011. |
| 456 | Nct, Acupuncture for Patients With Diarrhea-predominant IBS or Functional Diarrhea: a Randomized Controlled Trial. https://clinicaltrials.gov/show/NCT01350570, 2011. |
| 457 | Nct, Effect of Electroacupuncture in Patients With Irritable Bowel Syndrome. https://clinicaltrials.gov/show/NCT00900965, 2009. |
| 458 | Nct, Acupuncture/Moxibustion for Irritable Bowel Syndrome (IBS). https://clinicaltrials.gov/show/NCT00945074, 2009. |
| 459 | Nct, Effect of Acupuncture on Symptoms of Diarrhea and Pain in IBS. https://clinicaltrials.gov/show/NCT00219505, 2005. |
| 460 | Nct, Acupuncture for Irritable Bowel Syndrome (IBS). https://clinicaltrials.gov/show/NCT00093327, 2004. |
| 461 | Nct, Acupuncture for Irritable Bowel Syndrome. https://clinicaltrials.gov/show/NCT00065403, 2003. |
| 462 | Nakano, M., A. Maeda, M. Sakamoto, S. Yamazaki, T. Shibaji, and N. Suzuki, Management of intractable toothache based on personality disorder: A case report. Journal of Japanese Dental Society of Anesthesiology, 2005. 33(3): p. 398-402. |
| 463 | Mullin, G.E., O. Pickett-Blakely, and J.O. Clarke, Integrative medicine in gastrointestinal disease: Evaluating the evidence. Expert Review of Gastroenterology and Hepatology, 2008. 2(2): p. 261-280. |
| 464 | Mullin, G.E. and J.O. Clarke, Role of Complementary and Alternative Medicine in Managing Gastrointestinal Motility Disorders. Nutrition in Clinical Practice, 2010. 25(1): p. 85-87. |
| 465 | Mulak, A. and L. Paradowski, Therapy for irritable bowel syndrome - Present possibilities and new perspectives. Gastroenterologia Polska, 2004. 11(6): p. 521-527. |
| 466 | Moynihan, N.T., M.J. Callahan, B. Kalsmith, and P.L. Moses, How do you spell relief for irritable bowel syndrome? Journal of Family Practice, 2008. 57(2): p. 100-108. |
| 467 | Mountifield, R. and J.M. Andrews, Managing irritable bowel syndrome. Medicine Today, 2010. 11(2): p. 32-40. |
| 468 | Mou, J.J., Q. Wang, H.Y. Luo, and Q.K. Feng, The observation of clinical efficacy of treating IBS-D with needle warming therapy. World chinese medicine [shi jie zhong yi yao], 2016. 11(11): p. 2404‐2407. |
| 469 | Mora, R.M., Doctor-patient relationship is the most established component of placebo effect. FMC Formacion Medica Continuada en Atencion Primaria, 2009. 16(3): p. 178. |
| 470 | Mora, R.M., Doctor-patient relationship is the most established component of placebo effect. FMC formacion medica continuada en atencion primaria, 2009. 16(3): p. 178. |
| 471 | Molfetta, L., G. Saviola, D. Fornasari, P.A. Grossi, A. Corcione, and B. Frediani, Osteoarticular pain: therapeutic approach by paradigms. European Review for Medical and Pharmacological Sciences, 2022. 26(11): p. 4054-4068. |
| 472 | Moayyedi, P., M. Marsiglio, C.N. Andrews, L.A. Graff, C. Korownyk, B. Kvern, et al., Patient engagement and multidisciplinary involvement has an impact on clinical guideline development and decisions: A comparison of two irritable bowel syndrome guidelines using the same data. Journal of the Canadian Association of Gastroenterology, 2019. 2(1): p. 30-36. |
| 473 | Moayyedi, P., C.N. Andrews, G. MacQueen, C. Korownyk, M. Marsiglio, L. Graff, et al., Canadian Association of gastroenterology clinical practice guideline for the management of irritable bowel syndrome (IBS). Journal of the Canadian Association of Gastroenterology, 2019. 2(1): p. 6-29. |
| 474 | Moayyedi, P., C.N. Andrews, G. MacQueen, C. Korownyk, M. Marsiglio, L. Graff, et al., Canadian Association of Gastroenterology Clinical Practice Guideline for the Management of Irritable Bowel Syndrome (IBS). J Can Assoc Gastroenterol, 2019. 2(1): p. 6-29. |
| 475 | Mizuta, Y., S. Shikuwa, H. Isomoto, R. Mishima, Y. Akazawa, J.I. Masuda, et al., Recent insights into digestive motility in functional dyspepsia. Journal of Gastroenterology, 2006. 41(11): p. 1025-1040. |
| 476 | Mion, F., S. Pellissier, A. Garros, H. Damon, S. Roman, and B. Bonaz, Transcutaneous auricular vagus nerve stimulation for the treatment of irritable bowel syndrome: a pilot, open-label study. Bioelectronics in medicine, 2020. 3(1): p. 167‐174. |
| 477 | Minocha, A. and T. Abell, Irritable bowel syndrome in the older adult. Geriatrics and Aging, 2004. 7(10): p. 15-19. |
| 478 | Minocha, A., Irritable bowel syndrome in the older patient. Clinical Geriatrics, 2005. 13(4): p. 19-24. |
| 479 | Milgrom, L.R., On the observed specific and non-specific effects of complex therapeutic interventions: Truly separate or complementary? 2015. p. 79-92. |
| 480 | Michelfelder, A.J., K.C. Lee, and E.M. Bading, Integrative medicine and gastrointestinal disease. Primary Care - Clinics in Office Practice, 2010. 37(2): p. 255-267. |
| 481 | Michael Wolfe, M. and R.C. Lowe, Investing in the future of GERD. Journal of Clinical Gastroenterology, 2007. 41(SUPPL.2): p. S209-S216. |
| 482 | Metyas, S., C. Chen, M. Joseph, N. Hanna, J. Basta, and A. Khalil, Subcategories of Fibromyalgia-A New Concept. Current Rheumatology Reviews, 2023. 19(1): p. 18-25. |
| 483 | Mengzhu, S., Z. Yujie, S. Yafang, G. Jing, W. Yuhang, X. Chen, et al., Electroacupuncture alleviates water avoidance stress-induced irritable bowel syndrome in mice by improving intestinal barrier functions and suppressing the expression of inflammatory cytokines. J Tradit Chin Med, 2023. 43(3): p. 494-500. |
| 484 | Mengzhu, S., Z. Yujie, S. Yafang, G. Jing, Z. Tingting, W. Yuhang, et al., Electroacupuncture at Tianshu (ST25) and Zusanli (ST36) alleviates stress-induced irritable bowel syndrome in mice by modulating gut microbiota and corticotropin-releasing factor. J Tradit Chin Med, 2022. 42(5): p. 732-740. |
| 485 | Meissner, K., Journal Club. Complementary Medicine Research, 2022. 29(2): p. 93-94. |
| 486 | Meisler, J.G., Toward optimal health: The experts discuss fibromyalgia. Journal of Women's Health and Gender-Based Medicine, 2000. 9(10): p. 1055-1060. |
| 487 | Mease, P., Fibromyalgia syndrome: Review of clinical presentation, pathogenesis, outcome measures, and treatment. JOURNAL OF RHEUMATOLOGY, 2005. 32: p. 6-21. |
| 488 | Mease, P., Fibromyalgia syndrome: Review of clinical presentation, pathogenesis, outcome measures, and treatment. Journal of Rheumatology, 2005. 32(SUPPL. 75): p. 6-21. |
| 489 | Mearin, F., E. Rey, and A. Balboa, Functional and motor gastrointestinal disorders. Gastroenterologia y Hepatologia, 2015. 38: p. 3-12. |
| 490 | Mearin, F., E. Rey, and A. Balboa, Functional and motor gastrointestinal disorders. Gastroenterologia y hepatologia, 2015. 38: p. 3-12. |
| 491 | Mearin, F., E. Rey, and A. Balboa, Functional and motor gastrointestinal disorders. Gastroenterologia y Hepatologia, 2015. 38: p. 3-12. |
| 492 | Mearin, F., E. Rey, and A. Balboa, [Functional and motor gastrointestinal disorders]. Gastroenterol Hepatol, 2015. 38 Suppl 1: p. 3-12. |
| 493 | Mearin, F., C. Ciriza, M. Minguez, E. Rey, J.J. Mascort, E. Pena, et al., Irritable bowel syndrome with constipation and functional constipation in adults: Treatment (Part 2 of 2). ATENCION PRIMARIA, 2017. 49(3): p. 177-194. |
| 494 | Mearin, F., C. Ciriza, M. Mínguez, E. Rey, J.J. Mascort, E. Peña, et al., Clinical Practice Guideline: Irritable bowel syndrome with constipation and functional constipation in the adult. Rev Esp Enferm Dig, 2016. 108(6): p. 332-363. |
| 495 | McPartland, J.M., G.W. Guy, and V. Di Marzo, Care and feeding of the endocannabinoid system: A systematic review of potential clinical interventions that upregulate the endocannabinoid system. PLoS ONE, 2014. 9(3). |
| 496 | Masuy, I., J. Pannemans, and J. Tack, Irritable bowel syndrome: diagnosis and management. MINERVA GASTROENTEROLOGICA E DIETOLOGICA, 2020. 66(2): p. 136-150. |
| 497 | Manheimer, E., S. Wieland, E. Kimbrough, K. Cheng, and B.M. Berman, Evidence from the cochrane collaboration for traditional chinese medicine therapies. Journal of Alternative and Complementary Medicine, 2009. 15(9): p. 1001-1014. |
| 498 | Manheimer, E., L.S. Wieland, K. Cheng, S.M. Li, X.Y. Shen, B.M. Berman, et al., Acupuncture for Irritable Bowel Syndrome: Systematic Review and Meta-Analysis. AMERICAN JOURNAL OF GASTROENTEROLOGY, 2012. 107(6): p. 835-847. |
| 499 | Manheimer, E., L.S. Wieland, K. Cheng, S.M. Li, X. Shen, B.M. Berman, et al., Acupuncture for irritable bowel syndrome: Systematic review and meta-analysis. American Journal of Gastroenterology, 2012. 107(6): p. 835-847. |
| 500 | Manheimer, E., L.S. Wieland, K. Cheng, S.M. Li, X. Shen, B.M. Berman, et al., Acupuncture for irritable bowel syndrome: systematic review and meta-analysis. Am J Gastroenterol, 2012. 107(6): p. 835-847; quiz 848. |
| 501 | Manheimer, E., K. Cheng, L.S. Wieland, L.S. Min, X.Y. Shen, B.M. Berman, et al., Acupuncture for treatment of irritable bowel syndrome. COCHRANE DATABASE OF SYSTEMATIC REVIEWS, 2012(5). |
| 502 | Manheimer, E., K. Cheng, L.S. Wieland, L.S. Min, X. Shen, B.M. Berman, et al., Acupuncture for treatment of irritable bowel syndrome. Cochrane database of systematic reviews (Online), 2012. 5: p. CD005111. |
| 503 | Manheimer, E., K. Cheng, L.S. Wieland, L.S. Min, X. Shen, B.M. Berman, et al., Acupuncture for treatment of irritable bowel syndrome. Cochrane Database of Systematic Reviews, 2012(5). |
| 504 | Manheimer, E., K. Cheng, L.S. Wieland, L.S. Min, X. Shen, B.M. Berman, et al., Acupuncture for treatment of irritable bowel syndrome. Cochrane Database Syst Rev, 2012. 2012(5): p. Cd005111. |
| 505 | Malone, M.A., Irritable Bowel Syndrome. Primary Care - Clinics in Office Practice, 2011. 38(3): p. 433-447. |
| 506 | Mak, A.D.P., V.C.H. Chung, S.Y. Yuen, Y.K. Tse, S.Y.S. Wong, Y. Ju, et al., Noneffectiveness of electroacupuncture for comorbid generalized anxiety disorder and irritable bowel syndrome. JOURNAL OF GASTROENTEROLOGY AND HEPATOLOGY, 2019. 34(10): p. 1736-1742. |
| 507 | Mak, A.D.P., V.C.H. Chung, S.Y. Yuen, Y.K. Tse, S.Y.S. Wong, Y. Ju, et al., Noneffectiveness of electroacupuncture for comorbid generalized anxiety disorder and irritable bowel syndrome. Journal of Gastroenterology and Hepatology (Australia), 2019. 34(10): p. 1736-1742. |
| 508 | Mak, A.D., V.C.H. Chung, S.Y. Yuen, Y.K. Tse, S.Y.S. Wong, Y. Ju, et al., Noneffectiveness of electroacupuncture for comorbid generalized anxiety disorder and irritable bowel syndrome. J Gastroenterol Hepatol, 2019. 34(10): p. 1736-1742. |
| 509 | Mak, A.D., V.C.H. Chung, S.Y. Yuen, Y.K. Tse, S.Y.S. Wong, Y. Ju, et al., Noneffectiveness of electroacupuncture for comorbid generalized anxiety disorder and irritable bowel syndrome. Journal of gastroenterology and hepatology, 2019. 34(10): p. 1736‐1742. |
| 510 | Mahabir, V.K., C.S. Smith, C. Vannabouathong, J.J. Merchant, and A.L. Garibaldi, Comparing medical cannabis use in 5 US states: a retrospective database study. Journal of Cannabis Research, 2021. 3(1). |
| 511 | Magge, S.S. and J.L. Wolf, Complementary and alternative medicine and mind-body therapies for treatment of irritable bowel syndrome in women. Women's Health, 2013. 9(6): p. 557-567. |
| 512 | Magge, S.S. and J.L. Wolf, Complementary and alternative medicine and mind-body therapies for treatment of irritable bowel syndrome in women. Womens Health (Lond), 2013. 9(6): p. 557-567. |
| 513 | Magge, S. and A. Lembo, Complementary and Alternative Medicine for the Irritable Bowel Syndrome. Gastroenterology Clinics of North America, 2011. 40(1): p. 245-253. |
| 514 | Magge, S. and A. Lembo, Complementary and alternative medicine for the irritable bowel syndrome. Gastroenterol Clin North Am, 2011. 40(1): p. 245-253. |
| 515 | MacPherson, H., H. Tilbrook, J.M. Bland, K. Bloor, S. Brabyn, H. Cox, et al., Acupuncture for irritable bowel syndrome: primary care based pragmatic randomised controlled trial. BMC GASTROENTEROLOGY, 2012. 12. |
| 516 | MacPherson, H., H. Tilbrook, J.M. Bland, K. Bloor, S. Brabyn, H. Cox, et al., Acupuncture for irritable bowel syndrome: Primary care based pragmatic randomised controlled trial. BMC Gastroenterology, 2012. 12. |
| 517 | MacPherson, H., H. Tilbrook, J.M. Bland, K. Bloor, S. Brabyn, H. Cox, et al., Acupuncture for irritable bowel syndrome: primary care based pragmatic randomised controlled trial. BMC Gastroenterol, 2012. 12: p. 150. |
| 518 | MacPherson, H., H. Tilbrook, J.M. Bland, K. Bloor, S. Brabyn, H. Cox, et al., Acupuncture for irritable bowel syndrome: primary care based pragmatic randomised controlled trial. BMC gastroenterology, 2012. 12: p. 150. |
| 519 | MacPherson, H., H. Tilbrook, D. Agbedjro, H. Buckley, C. Hewitt, and C. Frost, Acupuncture for irritable bowel syndrome: 2-year follow-up of a randomised controlled trial. ACUPUNCTURE IN MEDICINE, 2017. 35(1). |
| 520 | MacPherson, H., H. Tilbrook, D. Agbedjro, H. Buckley, C. Hewitt, and C. Frost, Acupuncture for irritable bowel syndrome: 2-year follow-up of a randomised controlled trial. Acupuncture in medicine : journal of the British Medical Acupuncture Society, 2017. 35(1): p. 17-23. |
| 521 | MacPherson, H., H. Tilbrook, D. Agbedjro, H. Buckley, C. Hewitt, and C. Frost, Acupuncture for irritable bowel syndrome: 2-year follow-up of a randomised controlled trial. Acupuncture in medicine, 2017. 35(1): p. 17‐23. |
| 522 | MacPherson, H., H. Tilbrook, D. Agbedjro, H. Buckley, C. Hewitt, and C. Frost, Acupuncture for irritable bowel syndrome: 2-year follow-up of a randomised controlled trial. Acupuncture in medicine, 2017. 35(1): p. 17‐23. |
| 523 | MacPherson, H., H. Tilbrook, D. Agbedjro, H. Buckley, C. Hewitt, and C. Frost, Acupuncture for irritable bowel syndrome: 2-year follow-up of a randomised controlled trial. Acupunct Med, 2017. 35(1): p. 17-23. |
| 524 | MacPherson, H., M. Bland, K. Bloor, H. Cox, D. Geddes, A. Kang'ombe, et al., Acupuncture for irritable bowel syndrome: A protocol for a pragmatic randomised controlled trial. BMC GASTROENTEROLOGY, 2010. 10. |
| 525 | MacPherson, H., M. Bland, K. Bloor, H. Cox, D. Geddes, A. Kang'ombe, et al., Acupuncture for irritable bowel syndrome: A protocol for a pragmatic randomised controlled trial. BMC Gastroenterology, 2010. 10. |
| 526 | MacPherson, H., M. Bland, K. Bloor, H. Cox, D. Geddes, A. Kang'ombe, et al., Acupuncture for irritable bowel syndrome: a protocol for a pragmatic randomised controlled trial. BMC Gastroenterol, 2010. 10: p. 63. |
| 527 | MacPherson, H., M. Bland, K. Bloor, H. Cox, D. Geddes, A. Kang'ombe, et al., Acupuncture for irritable bowel syndrome: a protocol for a pragmatic randomised controlled trial. BMC gastroenterology, 2010. 10: p. 63. |
| 528 | MacPherson, H., D.G. Altman, R. Hammerschlag, Y.P. Li, T.X. Wu, A. White, et al., Revised STandards for Reporting Interventions in Clinical Trials of Acupuncture (STRICTA): Extending the CONSORT Statement. JOURNAL OF ALTERNATIVE AND COMPLEMENTARY MEDICINE, 2010. 16(10): p. ST1-ST14. |
| 529 | MacPherson, H., D.G. Altman, R. Hammerschlag, Y.P. Li, T.X. Wu, A. White, et al., Revised STandards for Reporting Interventions in Clinical Trials of Acupuncture (STRICTA): extending the CONSORT statement. ACUPUNCTURE IN MEDICINE, 2010. 28(2): p. 83-93. |
| 530 | MacPherson, H., D.G. Altman, R. Hammerschlag, Y.P. Li, T.X. Wu, A. White, et al., Revised STandards for Reporting Interventions in Clinical Trials of Acupuncture (STRICTA): Extending the CONSORT Statement. PLOS MEDICINE, 2010. 7(6). |
| 531 | MacPherson, H., Pragmatic clinical trials. Complementary Therapies in Medicine, 2004. 12(2-3): p. 136-140. |
| 532 | Ma, Z.B., Y.Y. Zheng, L.X. Ma, N.N. Guo, C. Li, Y.P. Wang, et al., Clinical studies on the indications of 33 acupoints. Medical Acupuncture, 2008. 20(4): p. 269-275. |
| 533 | Ma, Z.B., Y.Y. Zheng, L.X. Ma, N.N. Guo, C. Li, Y.P. Wang, et al., Clinical studies on the indications of 33 acupoints. Medical acupuncture, 2008. 20(4): p. 269‐275. |
| 534 | Ma, Y., M. Dong, K. Zhou, C. Mita, J. Liu, and P.M. Wayne, Publication trends in acupuncture research: A 20-year bibliometric analysis based on pubMed. PLoS ONE, 2016. 11(12). |
| 535 | Ma, Y., M. Dong, K. Zhou, C. Mita, J. Liu, and P.M. Wayne, Publication trends in acupuncture research: a 20-year bibliometric analysis based on pubMed. PloS one, 2016. 11(12) (no pagination). |
| 536 | Ma, X.P., L.Y. Tan, Y. Yang, H.G. Wu, B. Jiang, H.R. Liu, et al., Effect of electro-acupuncture on substance P, its receptor and corticotropin-releasing hormone in rats with irritable bowel syndrome. World J Gastroenterol, 2009. 15(41): p. 5211-5217. |
| 537 | Ma, X.P., J. Hong, C.P. An, D. Zhang, Y. Huang, H.G. Wu, et al., Acupuncture-moxibustion in treating irritable bowel syndrome: How does it work? WORLD JOURNAL OF GASTROENTEROLOGY, 2014. 20(20): p. 6044-6054. |
| 538 | Ma, T.T., F. Zeng, Y. Li, C.M. Wang, X.P. Tian, S.Y. Yu, et al., Which Subtype of Functional Dyspepsia Patients Responses Better to Acupuncture? A Retrospective Analysis of a Randomized Controlled Trial. FORSCHENDE KOMPLEMENTARMEDIZIN, 2015. 22(2): p. 94-100. |
| 539 | Ma, T.H. and S.J. Zhang, Shenling Baizhu San combined with acupoint RN 8 application for treating diarrhea-predominant irritable bowel syndrome in 72 Cases. Hebei journal of traditional chinese medicine [he bei zhong yi], 2011. 31(1): p. 48‐49. |
| 540 | Ma, S.S., H. Yu, Y.Q. Li, X.W. Zhang, N. Liang, Z. Li, et al., Qualitative research on TCM complex interventions - the experience and cognition of Chinese patients. European Journal of Integrative Medicine, 2023. 59. |
| 541 | Lv, P.R., Y.S. Su, W. He, X.Y. Wang, H. Shi, X.N. Zhang, et al., Electroacupuncture Alleviated Referral Hindpaw Hyperalgesia via Suppressing Spinal Long-Term Potentiation (LTP) in TNBS-Induced Colitis Rats. Neural Plast, 2019. 2019: p. 2098083. |
| 542 | Luty, J., Medically unexplained syndromes: Irritable bowel syndrome, fibromyalgia and chronic fatigue. BJPsych Advances, 2018. 24(4): p. 252-263. |
| 543 | Luo, Q.P., S.S. Kuang, X.F. Yang, L.M. Jin, P.B. Chen, Y. Chen, et al., Bowls-unblocking and mind-regulating acupuncture therapy for constipation-predominant irritable bowel syndrome: An exploratory randomized clinical trial 通腑调神针法治疗便秘型肠易激综合征:随机对照试验. World Journal of Acupuncture - Moxibustion, 2023. 33(2): p. 90-96. |
| 544 | Luo, Q.P., S.S. Kuang, X.F. Yang, L.M. Jin, P.B. Chen, Y. Chen, et al., Bowls-unblocking and mind-regulating acupuncture therapy for constipation-predominant irritable bowel syndrome: an exploratory randomized clinical trial 通腑调神针法治疗便秘型肠易激综合征: 随机对照试验. World journal of acupuncture - moxibustion, 2023. |
| 545 | Luigi, G.V.M.A.V. and F. Fabio, Randomised controlled trial comparing acupuncture with placebo acupuncture for the treatment of irritable bowel syndrome. 2012. 4: p. 121. |
| 546 | Luigi, G., M. Vittorio, V. Alfredo, and F. Fabio, Randomised controlled trial comparing acupuncture with placebo acupuncture for the treatment of irritable bowel syndrome. European Journal of Integrative Medicine, 2012. 4: p. 121. |
| 547 | Luigi, G., M. Vittorio, V. Alfredo, and F. Fabio, Randomised controlled trial comparing acupuncture with placebo acupuncture for the treatment of irritable bowel syndrome. European journal of integrative medicine, 2012. 4: p. 121. |
| 548 | Lu, C.L. and F.Y. Chang, Placebo effect in patients with irritable bowel syndrome. Journal of Gastroenterology and Hepatology (Australia), 2011. 26(SUPPL. 3): p. 116-118. |
| 549 | Lowe, C., W. Depew, and S. Vanner, A placebo-controlled, double-blind trial of acupuncture in the treatment of irritable bowel syndrome (IBS). Gastroenterology, 2000. 118(4 Suppl 2): p. A3168. |
| 550 | Lowe, C., A. Aiken, A.G. Day, W. Depew, and S.J. Vanner, Sham acupuncture is as efficacious as true acupuncture for the treatment of IBS: A randomized placebo controlled trial. NEUROGASTROENTEROLOGY AND MOTILITY, 2017. 29(7). |
| 551 | Lowe, C., A. Aiken, A.G. Day, W. Depew, and S.J. Vanner, Sham acupuncture is as efficacious as true acupuncture for the treatment of IBS: A randomized placebo controlled trial. Neurogastroenterology and Motility, 2017. 29(7). |
| 552 | Lowe, C., A. Aiken, A.G. Day, W. Depew, and S.J. Vanner, Sham acupuncture is as efficacious as true acupuncture for the treatment of IBS: a randomized placebo controlled trial. Neurogastroenterology and motility, 2017. (no pagination). |
| 553 | Lowe, C., A. Aiken, A.G. Day, W. Depew, and S.J. Vanner, Sham acupuncture is as efficacious as true acupuncture for the treatment of IBS: a randomized placebo controlled trial. Neurogastroenterology and motility, 2017. 29(7). |
| 554 | Lowe, C., A. Aiken, A.G. Day, W. Depew, and S.J. Vanner, Sham acupuncture is as efficacious as true acupuncture for the treatment of IBS: A randomized placebo controlled trial. Neurogastroenterol Motil, 2017. 29(7). |
| 555 | Long, Z.R., C.H. Yu, Y. Yang, H.N. Wang, and X.X. Chi, Clinical observation on acupuncture combined with microorganism pharmaceutical preparations for treatment of irritable bowel syndrome of constipation type. Zhongguo zhen jiu = Chinese acupuncture & moxibustion, 2006. 26(6): p. 403-405. |
| 556 | Long, Z.R., C.H. Yu, Y. Yang, H.N. Wang, and X.X. Chi, Clinical observation on acupuncture combined with microorganism pharmaceutical preparations for treatment of irritable bowel syndrome of constipation type. Zhongguo zhen jiu [Chinese acupuncture & moxibustion], 2006. 26(6): p. 403‐405. |
| 557 | Long, Z.R., C.H. Yu, Y. Yang, H.N. Wang, and X.X. Chi, [Clinical observation on acupuncture combined with microorganism pharmaceutical preparations for treatment of irritable bowel syndrome of constipation type]. Zhongguo Zhen Jiu, 2006. 26(6): p. 403-405. |
| 558 | Liu, Y.C., Clinical study of ascending-descending acupuncture method regulation of the spleen and stomach for treatment of Spleen Deficiency-type diarrhea in irritable bowel syndrome. Shanghai journal of acupuncture and moxibustion [shang hai zhen jiu za zhi], 2015. 34(1): p. 25‐27. |
| 559 | Liu, X., J.N. Cao, T. Liu, H. Zhong, M. Liu, X.R. Chang, et al., Effect of herb-partitioned moxibustion on structure and functional prediction of gut microbiota in rats with irritable bowel syndrome with diarrhea. World Journal of Traditional Chinese Medicine, 2023. 9(2): p. 141-149. |
| 560 | Liu, W.H., C. Chen, F. Wang, S.N. Guo, Y. Hao, and S.D. Li, Development trend and current situation of acupuncture-moxibustion indications. World Journal of Acupuncture - Moxibustion, 2020. 30(4): p. 245-250. |
| 561 | Liu, N. and J. Wang, Clinical study of acupuncture at Shangjuxu (ST37) in treating irritable bowel syndrome. International journal of clinical acupuncture, 2013. 22(3): p. 135‐137. |
| 562 | Liu, M.R., R.F. Xiao, Z.P. Peng, H.N. Zuo, K. Zhu, and S.M. Wang, [Effect of acupuncture at "Zusanli" (ST 36 and "Taichong" (LR 3) on gastrointestinal hormone levels in rats with diarrhea type irritable bowel syndrome]. Zhen ci yan jiu = Acupuncture research / [Zhongguo yi xue ke xue yuan Yi xue qing bao yan jiu suo bian ji], 2012. 37(5): p. 363-368. |
| 563 | Liu, M.R., R.F. Xiao, Z.P. Peng, H.N. Zuo, K. Zhu, and S.M. Wang, [Effect of acupuncture at "Zusanli" (ST 36 and "Taichong" (LR 3) on gastrointestinal hormone levels in rats with diarrhea type irritable bowel syndrome]. Zhen Ci Yan Jiu, 2012. 37(5): p. 363-368. |
| 564 | Liu, J.J., C.A.Y. Chen, E. Giovannucci, and C.Y. Wu, Subsequent Primary Cancers of the Digestive System Among Childhood and Adolescent Cancer Survivors From 1975 to 2015 in the United States. AMERICAN JOURNAL OF GASTROENTEROLOGY, 2021. 116(5): p. 1063-1071. |
| 565 | Liu, J., R. Peng, Q. Tan, B. Li, J. Chen, G. Liu, et al., Proteomic analysis of rat colonic mucosa following acupuncture treatment for irritable bowel syndrome with diarrhea. PLoS ONE, 2022. 17(9 September). |
| 566 | Liu, J., R. Peng, Q. Tan, B. Li, J. Chen, G. Liu, et al., Proteomic analysis of rat colonic mucosa following acupuncture treatment for irritable bowel syndrome with diarrhea. PLoS One, 2022. 17(9): p. e0273853. |
| 567 | Liu, H.R., X.Y. Fang, H.G. Wu, L.Y. Wu, J. Li, Z.J. Weng, et al., Effects of electroacupuncture on corticotropin-releasing hormone in rats with chronic visceral hypersensitivity. World Journal of Gastroenterology, 2015. 21(23): p. 7181-7190. |
| 568 | Liu, H.R., X.Y. Fang, H.G. Wu, L.Y. Wu, J. Li, Z.J. Weng, et al., Effects of electroacupuncture on corticotropin-releasing hormone in rats with chronic visceral hypersensitivity. World J Gastroenterol, 2015. 21(23): p. 7181-7190. |
| 569 | Liu, H.H., X.D. Liu, Y.J. Wang, H.Q. Guan, J.Y. Chai, J.R. Zhao, et al., [Effects of acupoint area and non-acupoint area of eye-acupuncture on expressions of VIP and AQP 8 in colonic tissues in rats with D-IBS]. Zhongguo zhen jiu = Chinese acupuncture & moxibustion, 2012. 32(10): p. 919-924. |
| 570 | Liu, H.H., X.D. Liu, Y.J. Wang, H.Q. Guan, J.Y. Chai, J.R. Zhao, et al., [Effects of acupoint area and non-acupoint area of eye-acupuncture on expressions of VIP and AQP 8 in colonic tissues in rats with D-IBS]. Zhongguo Zhen Jiu, 2012. 32(10): p. 919-924. |
| 571 | Liu, H., Y. Zhang, D. Qi, and W. Li, Downregulation of the spinal NMDA receptor NR2B subunit during electro-acupuncture relief of chronic visceral hyperalgesia. The journal of physiological sciences : JPS, 2017. 67(1): p. 197-206. |
| 572 | Liu, H., Y. Zhang, D. Qi, and W. Li, Downregulation of the spinal NMDA receptor NR2B subunit during electro-acupuncture relief of chronic visceral hyperalgesia. J Physiol Sci, 2017. 67(1): p. 197-206. |
| 573 | Liu, H., C. Bao, C. Wang, Y. Yan, J. Li, Z. Hu, et al., LONG-TERM EFFECT OF MOXIBUSTION ON IRRITABLE BOWEL SYNDROME: A RANDOMIZED CLINCAL TRIAL. Gastroenterology, 2018. 154(6): p. S-196. |
| 574 | Liu, H., C. Bao, C. Wang, Y. Yan, J. Li, Z. Hu, et al., LONG-TERM EFFECT OF MOXIBUSTION ON IRRITABLE BOWEL SYNDROME: a RANDOMIZED CLINCAL TRIAL. Gastroenterology, 2018. 154(6): p. S‐196. |
| 575 | Liu, H., C. Bao, J. Li, Z. Hu, Y. Shi, J. Shen, et al., EFFECT OF MOXIBUSTION ON SYMPTOMS AND EVENT-RALATED POTENTIAL IN IRRITABLE BOWEL SYNDROME: A RANDOMIZED CONTROLLED TRIAL. Gastroenterology, 2020. 158(6): p. S-852. |
| 576 | Liu, H., C. Bao, J. Li, Z. Hu, Y. Shi, J. Shen, et al., EFFECT OF MOXIBUSTION ON SYMPTOMS AND EVENT-RALATED POTENTIAL IN IRRITABLE BOWEL SYNDROME: a RANDOMIZED CONTROLLED TRIAL. Gastroenterology, 2020. 158(6): p. S‐852. |
| 577 | Liu, H., Regulation of acupuncture and moxibuxtion on purine receptors in ibs visceral pain. Purinergic Signalling, 2018. 14(1): p. S72-S73. |
| 578 | Liu, D., M. Ruan, C. Tong, and R. Huang, Effect of Shugan Jianpi Recipe Combined with Cross Moxibustion on Biochemical Examination Indexes and Total Score of TCM Symptoms in Patients with Spleen-Stomach Damp-Heat Diarrhea Irritable Bowel Syndrome. Comput Math Methods Med, 2022. 2022: p. 8286146. |
| 579 | Liu, C., T.T. Pang, S. Yin, J.H. Li, J.J. Yao, H.M. Li, et al., Curative efficiency and adverse events of alternative therapy and medicine for functional constipation in adults A protocol for systematic review and meta-analysis. MEDICINE, 2022. 101(14). |
| 580 | Little, J.W., Complementary and alternative medicine: Impact on dentistry. Oral Surgery, Oral Medicine, Oral Pathology, Oral Radiology, and Endodontics, 2004. 98(2): p. 137-145. |
| 581 | Linn, Y.C., Evidence-based medicine for traditional Chinese medicine: Exploring the evidence from a western medicine perspective. Proceedings of Singapore Healthcare, 2011. 20(1): p. 12-19. |
| 582 | Ling, Y., L. Yuan, Z. Haifeng, M. Xiaopeng, B. Chunhui, W. Huangan, et al., Effect of warming moxibustion Tianshu (ST 25, bilateral) and Qihai (CV 6) for the treatment of diarrhea-dominant irritable bowel syndrome: A patient-blinded pilot trial with orthogonal design. Journal of Traditional Chinese Medicine, 2017. 37(4): p. 538-545. |
| 583 | Ling, Y., L. Yuan, Z. Haifeng, M. Xiaopeng, B. Chunhui, W. Huangan, et al., Effect of warming moxibustion Tianshu (ST 25, bilateral) and Qihai (CV 6) for the treatment of diarrhea-dominant irritable bowel syndrome: a patient-blinded pilot trial with orthogonal design. Journal of traditional Chinese medicine / Chung i tsa chih ying wen pan, 2017. 37(4): p. 538‐545. |
| 584 | Linde, K. and S.N. Willich, How objective are systematic reviews? Differences between reviews on complementary medicine. JOURNAL OF THE ROYAL SOCIETY OF MEDICINE, 2003. 96(1): p. 17-22. |
| 585 | Linde, K. and S.N. Willich, How objective are systematic reviews? Differences between reviews on complementary medicine. Journal of the Royal Society of Medicine, 2003. 96(1): p. 17-22. |
| 586 | Linde, K. and S.N. Willich, How objective are systematic reviews? Differences between reviews on complementary medicine. Journal of the Royal Society of Medicine, 2003. 96(1): p. 17‐22. |
| 587 | Linde, K., G. ter Riet, M. Hondras, A. Vickers, R. Saller, and D. Melchart, Systematic reviews of complementary therapies - An annotated bibliography. Part 2: Herbal medicine. BMC Complementary and Alternative Medicine, 2001. 1. |
| 588 | Linde, K., Components of the placebo effect. Forschende Komplementarmedizin, 2008. 15(4): p. 230-232. |
| 589 | Lin, D.Z., Y.X. Ou, L.L. Li, K.X. Wu, Q. Zhang, J.Y. Yan, et al., Acupuncture for postoperative gastrointestinal dysfunction in cancer: a systematic review and meta-analysis. FRONTIERS IN ONCOLOGY, 2023. 13. |
| 590 | Lim, B., E. Manheimer, L. Lao, E. Ziea, J. Wisniewski, J. Liu, et al., Acupuncture for treatment of irritable bowel syndrome. COCHRANE DATABASE OF SYSTEMATIC REVIEWS, 2006(4). |
| 591 | Lim, B., E. Manheimer, L. Lao, E. Ziea, J. Wisniewski, J. Liu, et al., Acupuncture for treatment of irritable bowel syndrome. Cochrane Database of Systematic Reviews, 2006(4). |
| 592 | Lim, B., E. Manheimer, L. Lao, E. Ziea, J. Wisniewski, J. Liu, et al., Acupuncture for treatment of irritable bowel syndrome. Cochrane Database Syst Rev, 2006(4): p. Cd005111. |
| 593 | Liang, Y.S., Y. Zhang, S. Xie, L.P. Wang, C.L. Zhu, and C.Y. Yan, Observations on the Efficacy of Acupoint Catgut Embedding plus Taijiquan in Treating Irritable Bowel Syndrome of Constipation Type. Shanghai journal of acupuncture and moxibustion [shang hai zhen jiu za zhi], 2010. 29(10): p. 634‐635. |
| 594 | Liang, Y.S., Y. Zhang, and J.J. Feng, Observation on effect of Taijiquan exercise combined with catgut implantation at acupuncture point in the treatment of constipation type irritable bowel syndrome. Hubei journal of traditional chinese medicine [ hubei zhong yi za zhi], 2010. 32(10): p. 50‐51. |
| 595 | Liang, Y.S., S. Xie, Y. Zhang, L.P. Wang, and C.L. Zhu, Treatment of 40 cases of irritable bowel syndrome by acupoint thread burial therapy and taijiquan. Shanghai journal of traditional chinese medicine [shang hai zhong yi yao za zhi], 2010. 44(10): p. 38‐39; 43. |
| 596 | Liang, C., K.Y. Wang, B. Xu, and Z. Yu, Electroacupuncture at acupoint ST 37(Shangjuxu) improves function of the enteric nervous system in a novel mouse constipation model. BMC COMPLEMENTARY AND ALTERNATIVE MEDICINE, 2016. 16. |
| 597 | Li, Z., The treatment of irritable bowel syndrome by acupuncture. Journal of Chinese Medicine, 2004(74): p. 22-24. |
| 598 | Li, Y.L., C.J. Yao, R. Lei, F.J. Xie, Q. Xiong, L.H. Luo, et al., Acupuncture combined with Tongxieyaofang for diarrhea-type irritable bowel syndrome A protocol for meta-analysis. MEDICINE, 2020. 99(48). |
| 599 | Li, Y.L., C.J. Yao, R. Lei, F. Xie, Q. Xiong, L.H. Luo, et al., Acupuncture combined with Tongxieyaofang for diarrhea-type irritable bowel syndrome: A protocol for meta-analysis. Medicine (United States), 2020. 99(48): p. E23457. |
| 600 | Li, Y.L., C.J. Yao, R. Lei, F. Xie, Q. Xiong, L.H. Luo, et al., Acupuncture combined with Tongxieyaofang for diarrhea-type irritable bowel syndrome: A protocol for meta-analysis. Medicine (Baltimore), 2020. 99(48): p. e23457. |
| 601 | Li, Y., S. Peng, F. Liang, S. Liu, and J. Li, Effectiveness of acupuncture for irritable bowel syndrome: Protocol for a scoping review of systematic reviews and meta-analyses. Medicine (United States), 2022. 101(29): p. E29218. |
| 602 | Li, Y., S. Peng, F. Liang, S. Liu, and J. Li, Effectiveness of acupuncture for irritable bowel syndrome: Protocol for a scoping review of systematic reviews and meta-analyses. Medicine (Baltimore), 2022. 101(29): p. e29218. |
| 603 | Li, X.Q., S.Y. Mu, Q. Lu, and X. Lu, Therapeutic observation of diarrhea-predominant irritable bowel syndrome majorly treated by acupuncture with Ling Gui Ba Fa. Shanghai journal of acupuncture and moxibustion [shang hai zhen jiu za zhi], 2015. 34(1): p. 22‐24. |
| 604 | Li, X., K. Ren, X. Hong, S. Guo, S. Yu, and S. Yang, Ameliorating effects of electroacupuncture on the low-grade intestinal inflammation in rat model of diarrhea-predominant irritable bowel syndrome. Journal of Gastroenterology and Hepatology (Australia), 2022. 37(10): p. 1963-1974. |
| 605 | Li, X., K. Ren, X. Hong, S. Guo, S. Yu, and S. Yang, Ameliorating effects of electroacupuncture on the low-grade intestinal inflammation in rat model of diarrhea-predominant irritable bowel syndrome. J Gastroenterol Hepatol, 2022. 37(10): p. 1963-1974. |
| 606 | Li, P.F., Y. Luo, Q. Wang, S. Shu, K.J. Chen, D.H. Yu, et al., Efficacy and Safety of Acupuncture at Tianshu (ST25) for Functional Constipation: Evidence from 10 Randomized Controlled Trials. EVIDENCE-BASED COMPLEMENTARY AND ALTERNATIVE MEDICINE, 2020. 2020. |
| 607 | Li, K.W., H.R. Chu, J.R. Ruan, J.J. Wang, J.Y. Chen, S.S. Zhu, et al., [Effect of moxibustion on immune function homeostasis in rats with diarrhea irritable bowel syndrome based on SCF/c-kit signaling pathway]. Zhongguo Zhen Jiu, 2023. 43(2): p. 177-185. |
| 608 | Li, K., M. Guo, L. Tan, X. Li, Y. Wu, Y. Lan, et al., Comparison of effects of electroacupuncture at "Dachangshu" (BL 25) or "Tianshu" (ST 25) on visceral sensitivity, c-kit and TRPV1 of irritable bowel syndrome rats. Zhongguo zhen jiu = Chinese acupuncture & moxibustion, 2018. 38(6): p. 625-629. |
| 609 | Li, K., M. Guo, L. Tan, X. Li, Y. Wu, Y. Lan, et al., [Comparison of effects of electroacupuncture at "Dachangshu" (BL 25) or "Tianshu" (ST 25) on visceral sensitivity, c-kit and TRPV1 of irritable bowel syndrome rats]. Zhongguo Zhen Jiu, 2018. 38(6): p. 625-629. |
| 610 | Li, J., J. Lu, J. Sun, Z. Ruan, D. Xu, H. Geng, et al., Acupuncture with regulating mind and spleen for diarrhea irritable bowel syndrome and sleep quality:a randomized controlled trial. Zhongguo zhen jiu = Chinese acupuncture & moxibustion, 2017. 37(1): p. 9-13. |
| 611 | Li, J., J. Lu, J. Sun, Z. Ruan, D. Xu, H. Geng, et al., Acupuncture with regulating mind and spleen for diarrhea irritable bowel syndrome and sleep quality: a randomized controlled trial. Zhongguo zhen jiu [Chinese acupuncture & moxibustion], 2017. 37(1): p. 9‐13. |
| 612 | Li, J., J. Lu, J. Sun, Z. Ruan, D. Xu, H. Geng, et al., [Acupuncture with regulating mind and spleen for diarrhea irritable bowel syndrome and sleep quality:a randomized controlled trial]. Zhongguo Zhen Jiu, 2017. 37(1): p. 9-13. |
| 613 | Li, H.Y., Y. Chen, Z.Y. Hu, Y. Yi, J. Ye, Y.L. Zhou, et al., Comparison of acupuncture and pinaverium bromide in the treatment of irritable bowel syndrome A protocol for systematic review and meta-analysis. MEDICINE, 2021. 100(16). |
| 614 | Li, H.Y., Y. Chen, Z.Y. Hu, J.W. Jiang, J. Ye, Y.L. Zhou, et al., Effectiveness of acupuncture for anxiety and depression in irritable bowel syndrome A protocol for systematic review and meta-analysis. MEDICINE, 2021. 100(8). |
| 615 | Li, H.Y., Y. Chen, Z.Y. Hu, W. Chen, H.Y. Tang, Z.Y. Yu, et al., Meta analysis of acupuncture and moxibustion for anxiety and depression in irritable bowel syndrome. Zhen ci yan jiu = Acupuncture research, 2022. 47(9): p. 821-829. |
| 616 | Li, H.Y., Y. Chen, Z.Y. Hu, W. Chen, H.Y. Tang, Z.Y. Yu, et al., [Meta analysis of acupuncture and moxibustion for anxiety and depression in irritable bowel syndrome]. Zhen Ci Yan Jiu, 2022. 47(9): p. 821-829. |
| 617 | Li, H., L.X. Pei, J.L. Zhou, and J.H. Sun, Controlled observation on the efficacy of acupuncture and western medicine on diarrhea-type irritable bowel syndrome. World Journal of Acupuncture - Moxibustion, 2013. 23(2): p. 11-16. |
| 618 | Li, H., L.X. Pei, J.L. Zhou, and J.H. Sun, Controlled observation on the efficacy of acupuncture and western medicine on diarrhea-type irritable bowel syndrome. World journal of acupuncture - moxibustion, 2013. 23(2): p. 11‐16. |
| 619 | Li, H., L.X. Pei, and J.L. Zhou, [Comparative observation on therapeutic effects between acupuncture and western medication for diarrhea-predominant irritable bowel syndrome]. Zhongguo zhen jiu = Chinese acupuncture & moxibustion, 2012. 32(8): p. 679-682. |
| 620 | Li, H., L.X. Pei, and J.L. Zhou, Comparative observation on therapeutic effects between acupuncture and western medication for diarrhea-predominant irritable bowel syndrome. Zhongguo zhen jiu [Chinese acupuncture & moxibustion], 2012. 32(8): p. 679‐682. |
| 621 | Li, H., L.X. Pei, and J.L. Zhou, [Comparative observation on therapeutic effects between acupuncture and western medication for diarrhea-predominant irritable bowel syndrome]. Zhongguo Zhen Jiu, 2012. 32(8): p. 679-682. |
| 622 | Li, H., Y. Chen, Z. Hu, Y. Yi, J. Ye, Y. Zhou, et al., Comparison of acupuncture and pinaverium bromide in the treatment of irritable bowel syndrome: A protocol for systematic review and meta-analysis. Medicine (United States), 2021. 100(16): p. E25604. |
| 623 | Li, H., Y. Chen, Z. Hu, Y. Yi, J. Ye, Y. Zhou, et al., Comparison of acupuncture and pinaverium bromide in the treatment of irritable bowel syndrome: A protocol for systematic review and meta-analysis. Medicine (Baltimore), 2021. 100(16): p. e25604. |
| 624 | Li, C.Y. and S.C. Li, Treatment of irritable bowel syndrome in China: A review. World Journal of Gastroenterology, 2015. 21(8): p. 2315-2322. |
| 625 | Li, C.Y. and S.C. Li, Treatment of irritable bowel syndrome in China: a review. World J Gastroenterol, 2015. 21(8): p. 2315-2322. |
| 626 | Lewith, G., Acupuncture placebos. European Journal of Oriental Medicine, 2010. 6(4): p. 26-30. |
| 627 | Leung, W.D. and S. Sherman, Endoscopic Approach to the Patient with Motility Disorders of the Bile Duct and Sphincter of Oddi. Gastrointestinal Endoscopy Clinics of North America, 2013. 23(2): p. 405-434. |
| 628 | Leong, M., T.J. Smith, and A. Rowland-Seymour, Complementary and integrative medicine for older adults in palliative care. Clinics in Geriatric Medicine, 2015. 31(2): p. 177-191. |
| 629 | Lembo, A.J., L. Conboy, J.M. Kelley, R.S. Schnyer, C.A. McManus, M.T. Quilty, et al., A treatment trial of acupuncture in IBS patients. American Journal of Gastroenterology, 2009. 104(6): p. 1489-1497. |
| 630 | Lembo, A.J., L. Conboy, J.M. Kelley, R.S. Schnyer, C.A. McManus, M.T. Quilty, et al., A treatment trial of acupuncture in IBS patients. American journal of gastroenterology, 2009. 104(6): p. 1489‐1497. |
| 631 | Lembo, A.J., L. Conboy, J.M. Kelley, R.S. Schnyer, C.A. McManus, M.T. Quilty, et al., A treatment trial of acupuncture in IBS patients. Am J Gastroenterol, 2009. 104(6): p. 1489-1497. |
| 632 | Lei, C.C., L. Li, H. Zhang, J. Li, Q. Zhang, S.Q. Liu, et al., Effect of Electroacupuncture Stimulation of Sensitized Acupoints on Bowel Dysfunction in Rats with Diarrhea-predominant Irritable Bowel Syndrome. Zhen ci yan jiu = Acupuncture research, 2017. 42(5): p. 413-417. |
| 633 | Lei, C.C., L. Li, H. Zhang, J. Li, Q. Zhang, S.Q. Liu, et al., [Effect of Electroacupuncture Stimulation of Sensitized Acupoints on Bowel Dysfunction in Rats with Diarrhea-predominant Irritable Bowel Syndrome]. Zhen Ci Yan Jiu, 2017. 42(5): p. 413-417. |
| 634 | Lee, S., U.M. Jerng, Y. Liu, J.W. Kang, D. Nam, and J.D. Lee, The effectiveness and safety of moxibustion for treating cancer-related fatigue: a systematic review and meta-analyses. SUPPORTIVE CARE IN CANCER, 2014. 22(5): p. 1429-1440. |
| 635 | Lee, J.H., W.S. Sung, S.B. Bak, E.J. Kim, and Y.W. Kim, Evaluation of Xiaoyao-san for treatment of irritable bowel syndrome: A systematic review and meta-analysis of randomized controlled trials. European Journal of Integrative Medicine, 2022. 53. |
| 636 | Leahy, A. and O. Epstein, Non-pharmacological treatments in the irritable bowel syndrome. World Journal of Gastroenterology, 2001. 7(3): p. 313-316. |
| 637 | Langmead, L. and D.S. Rampton, Review article: Complementary and alternative therapies for inflammatory bowel disease. Alimentary Pharmacology and Therapeutics, 2006. 23(3): p. 341-349. |
| 638 | Langmead, L., M. Chitnis, and D.S. Rampton, Use of complementary therapies by patients with IBD may indicate psychosocial distress. Inflammatory Bowel Diseases, 2002. 8(3): p. 174-179. |
| 639 | Lam, C., Nutrition and medical acupuncture: Three case presentations. Journal of Orthomolecular Medicine, 2006. 21(4): p. 200-204. |
| 640 | Lahner, E., S. Bellentani, R. De Bastiani, C. Tosetti, M. Cicala, G. Esposito, et al., A survey of pharmacological and nonpharmacological treatment of functional gastrointestinal disorders. United European Gastroenterology Journal, 2013. 1(5): p. 385-393. |
| 641 | Lacy, B.E., K. Weiser, and R. De Lee, The treatment of irritable bowel syndrome. Therapeutic Advances in Gastroenterology, 2009. 2(4): p. 221-238. |
| 642 | Lacy, B.E. and R.D. Lee, Irritable bowel syndrome: A syndrome in evolution. Journal of Clinical Gastroenterology, 2005. 39(5 SUPPL. 3): p. S230-S242. |
| 643 | Lacy, B.E., Proton pump inhibitor nonresponders. Gastroenterology and Hepatology, 2015. 11(7): p. 483-485. |
| 644 | Kunze, M., H.J. Seidel, and G. Stübe, Comparative studies of the effectiveness of brief psychotherapy, acupuncture and papaverin therapy in patients with irritable bowel syndrome. Zeitschrift fur die gesamte innere Medizin und ihre Grenzgebiete, 1990. 45(20): p. 625‐627. |
| 645 | Kuner, R., Central mechanisms of pathological pain. Nature Medicine, 2010. 16(11): p. 1258-1266. |
| 646 | Kundu, A. and B. Berman, Acupuncture for pediatric pain and symptom management. PEDIATRIC CLINICS OF NORTH AMERICA, 2007. 54(6): p. 885-+. |
| 647 | Krasaelap, A., M.R. Sood, B.U.K. Li, R. Unteutsch, K. Yan, M. Nugent, et al., Efficacy of Auricular Neurostimulation in Adolescents With Irritable Bowel Syndrome in a Randomized, Double-Blind Trial. CLINICAL GASTROENTEROLOGY AND HEPATOLOGY, 2020. 18(9): p. 1987-+. |
| 648 | Krasaelap, A., M.R. Sood, B.U.K. Li, R. Unteutsch, K. Yan, M. Nugent, et al., Efficacy of Auricular Neurostimulation in Adolescents With Irritable Bowel Syndrome in a Randomized, Double-Blind Trial. Clinical gastroenterology and hepatology, 2020. 18(9): p. 1987‐1994.e1982. |
| 649 | Kovacic, K., K. Hainsworth, M. Sood, G. Chelimsky, R. Unteutsch, M. Nugent, et al., Neurostimulation for abdominal pain-related functional gastrointestinal disorders in adolescents: a randomised, double-blind, sham-controlled trial. LANCET GASTROENTEROLOGY & HEPATOLOGY, 2017. 2(10): p. 727-737. |
| 650 | Kovacic, K., K. Hainsworth, M. Sood, G. Chelimsky, R. Unteutsch, M. Nugent, et al., Neurostimulation for abdominal pain-related functional gastrointestinal disorders in adolescents: a randomised, double-blind, sham-controlled trial. The lancet. Gastroenterology & hepatology, 2017. 2(10): p. 727‐737. |
| 651 | Kou, Y., Q. Zhao, X.L. Wen, and B. Yang, Clinical observation on TCM triple therapy in treating diarrhea-pattern irritable bowel syndrome. Western journal of traditional chinese medicine [xi bu zhong yi yao], 2015. 28(11): p. 79‐81. |
| 652 | Kotsis, V., S. Benson, U. Bingel, M. Forsting, M. Schedlowski, E.R. Gizewski, et al., Perceived treatment group affects behavioral and neural responses to visceral pain in a deceptive placebo study. NEUROGASTROENTEROLOGY AND MOTILITY, 2012. 24(10). |
| 653 | Korzenik, J., A.K. Koch, and J. Langhorst, Complementary and Integrative Gastroenterology. Medical Clinics of North America, 2017. 101(5): p. 943-954. |
| 654 | Koretz, R.L. and M. Rotblatt, Complementary and Alternative Medicine in Gastroenterology: The Good, the Bad, and the Ugly. CLINICAL GASTROENTEROLOGY AND HEPATOLOGY, 2004. 2(11): p. 957-967. |
| 655 | Koretz, R.L. and M. Rotblatt, Complementary and alternative medicine in gastroenterology: The good, the bad, and the ugly. Clinical Gastroenterology and Hepatology, 2004. 2(11): p. 957-967. |
| 656 | Koretz, R.L. and M. Rotblatt, Complementary and alternative medicine in gastroenterology: the good, the bad, and the ugly. Clin Gastroenterol Hepatol, 2004. 2(11): p. 957-967. |
| 657 | Kokkotou, E., L.A. Conboy, D.C. Ziogas, M.T. Quilty, J.M. Kelley, R.B. Davis, et al., Serum correlates of the placebo effect in irritable bowel syndrome. NEUROGASTROENTEROLOGY AND MOTILITY, 2010. 22(3). |
| 658 | Kokkotou, E., L.A. Conboy, D.C. Ziogas, M.T. Quilty, J.M. Kelley, R.B. Davis, et al., Serum correlates of the placebo effect in irritable bowel syndrome. Neurogastroenterology and Motility, 2010. 22(3): p. 285-292+e281. |
| 659 | Kokkotou, E., L.A. Conboy, D.C. Ziogas, M.T. Quilty, J.M. Kelley, R.B. Davis, et al., Serum correlates of the placebo effect in irritable bowel syndrome. Neurogastroenterology and motility, 2010. 22(3): p. 285‐e281. |
| 660 | Kokkotou, E., L.A. Conboy, D.C. Ziogas, M.T. Quilty, J.M. Kelley, R.B. Davis, et al., Serum correlates of the placebo effect in irritable bowel syndrome. Neurogastroenterol Motil, 2010. 22(3): p. 285-e281. |
| 661 | Ko, S.J., J. Kim, J. Lee, K.J. Kim, H. Jun, T.J. Kaptchuk, et al., Influence of the patient-practitioner interaction context on acupuncture outcomes in functional dyspepsia. Integrative Medicine Research, 2020. 9. |
| 662 | Ko, S.J., J. Kim, J. Lee, K.J. Kim, H. Jun, T.J. Kaptchuk, et al., Influence of the patient-practitioner interaction context on acupuncture outcomes in functional dyspepsia. Integrative medicine research, 2020. 9. |
| 663 | Klinger, R., R. Kothe, J. Schmitz, S. Kamping, and H. Flor, Placebo effects of a sham opioid solution: a randomized controlled study in patients with chronic low back pain. PAIN, 2017. 158(10): p. 1893-1902. |
| 664 | Kirsch, I., Role of placebo in irritable bowel syndrome. J Pediatr Gastroenterol Nutr, 2011. 53 Suppl 2: p. S42-43. |
| 665 | Kim, T., J. Chung, S. Bae, J. Lee, J. Kim, J.A. Lee, et al., Efficacy and safety of different doses of moxibustion for irritable bowel syndrome: A randomised controlled pilot trial. EUROPEAN JOURNAL OF INTEGRATIVE MEDICINE, 2018. 20: p. 79-83. |
| 666 | Khan, S. and L. Chang, Diagnosis and management of IBS. Nature Reviews Gastroenterology and Hepatology, 2010. 7(10): p. 565-581. |
| 667 | Kerr, C.E., J.R. Shaw, L.A. Conboy, J.M. Kelley, E. Jacobson, and T.J. Kaptchuk, Placebo acupuncture as a form of ritual touch healing: A neurophenomenological model. Consciousness and Cognition, 2011. 20(3): p. 784-791. |
| 668 | Kerr, C.E., J.R. Shaw, L.A. Conboy, J.M. Kelley, E. Jacobson, and T.J. Kaptchuk, Placebo acupuncture as a form of ritual touch healing: a neurophenomenological model. Consciousness and cognition, 2011. 20(3): p. 784‐791. |
| 669 | Kerr, C.E., J.R. Shaw, L.A. Conboy, J.M. Kelley, E. Jacobson, and T.J. Kaptchuk, Placebo acupuncture as a form of ritual touch healing: a neurophenomenological model. Conscious Cogn, 2011. 20(3): p. 784-791. |
| 670 | Kelley, J.M., A.J. Lembo, J.S. Ablon, J.J. Villanueva, L.A. Conboy, R. Levy, et al., Patient and practitioner influences on the placebo effect in irritable bowel syndrome. Psychosomatic Medicine, 2009. 71(7): p. 789-797. |
| 671 | Kelley, J.M., A.J. Lembo, J.S. Ablon, J.J. Villanueva, L.A. Conboy, R. Levy, et al., Patient and practitioner influences on the placebo effect in irritable bowel syndrome. Psychosom Med, 2009. 71(7): p. 789-797. |
| 672 | Kelley, J.M., A.J. Lembo, J.S. Ablon, J.J. Villanueva, L.A. Conboy, R. Levy, et al., Patient and practitioner influences on the placebo effect in irritable bowel syndrome. Psychosomatic medicine, 2009. 71(7): p. 789‐797. |
| 673 | Kct, Symptom management for irritable bowel syndrome. https://trialsearch.who.int/Trial2.aspx?TrialID=KCT0002064, 2016. |
| 674 | Kaur, H. and A.S. Arunkalaivanan, Urethral pain syndrome and its management. Obstetrical and Gynecological Survey, 2007. 62(5): p. 348-351. |
| 675 | Kaspar, H., A. Abegg, and S. Reddy, Of odysseys and miracles: A narrative approach on therapeutic mobilities for ayurveda treatment. Social Science and Medicine, 2023. 334. |
| 676 | Kaptchuk, T.J. and N.J. Talley, Placebo acupuncture improved symptoms and quality of life in irritable bowel syndrome. Evidence-Based Medicine, 2008. 13(6): p. 180. |
| 677 | Kaptchuk, T.J., J. Shaw, C.E. Kerr, L.A. Conboy, J.M. Kelley, T.J. Csordas, et al., "Maybe i made up the whole thing": Placebos and patients' experiences in a randomized controlled trial. Culture, Medicine and Psychiatry, 2009. 33(3): p. 382-411. |
| 678 | Kaptchuk, T.J., J. Shaw, C.E. Kerr, L.A. Conboy, J.M. Kelley, T.J. Csordas, et al., "Maybe I made up the whole thing": placebos and patients' experiences in a randomized controlled trial. Culture, medicine and psychiatry, 2009. 33(3): p. 382‐411. |
| 679 | Kaptchuk, T.J., J. Shaw, C.E. Kerr, L.A. Conboy, J.M. Kelley, T.J. Csordas, et al., "Maybe I made up the whole thing": placebos and patients' experiences in a randomized controlled trial. Cult Med Psychiatry, 2009. 33(3): p. 382-411. |
| 680 | Kaptchuk, T.J., J.M. Kelley, L.A. Conboy, R.B. Davis, C.E. Kerr, E.E. Jacobson, et al., Components of placebo effect: randomised controlled trial inpatients with irritable bowel syndrome. BMJ-BRITISH MEDICAL JOURNAL, 2008. 336(7651): p. 999-1003. |
| 681 | Kaptchuk, T.J., J.M. Kelley, L.A. Conboy, R.B. Davis, C.E. Kerr, E.E. Jacobson, et al., Components of placebo effect: randomised controlled trial in patients with irritable bowel syndrome. Bmj, 2008. 336(7651): p. 999-1003. |
| 682 | Kaptchuk, T.J., J.M. Kelley, L.A. Conboy, R.B. Davis, C.E. Kerr, E.E. Jacobson, et al., Components of placebo effect: randomised controlled trial in patients with irritable bowel syndrome. BMJ (Clinical research ed.), 2008. 336(7651): p. 999‐1003. |
| 683 | Kaptchuk, T.J., J.M. Kelley, L.A. Conboy, R.B. Davis, C.E. Kerr, E.E. Jacobson, et al., Components of placebo effect: Randomised controlled trial in patients with irritable bowel syndrome. BMJ, 2008. 336(7651): p. 999-1003. |
| 684 | Kaiser, P., D.P. Kohen, M.L. Brown, R.L. Kajander, and A.J. Barnes, Integrating Pediatric Hypnosis with Complementary Modalities: Clinical Perspectives on Personalized Treatment. CHILDREN-BASEL, 2018. 5(8). |
| 685 | Jonas, W.B. and C. Crawford, Enhancing the patient-practitioner relationship can improve outcomes for acupuncture interventions in irritable bowel syndrome: Commentary. Focus on Alternative and Complementary Therapies, 2008. 13(3): p. 192-194. |
| 686 | Jonas, W.B. and C. Crawford, Enhancing the patient-practitioner relationship can improve outcomes for acupuncture interventions in irritable bowel syndrome: commentary. Focus on alternative and complementary therapies, 2008. 13(3): p. 192‐194. |
| 687 | Jindal, V., A. Ge, and P.J. Mansky, Safety and efficacy of acupuncture in children: A review of the evidence. Journal of Pediatric Hematology/Oncology, 2008. 30(6): p. 431-442. |
| 688 | Jin, X., Y.J. Ding, L.L. Wang, S.Q. Ding, L. Shu, Y.W. Jiang, et al., Clinical study on acupuncture for treatment of chronic functional constipation. Zhongguo zhen jiu = Chinese acupuncture & moxibustion, 2010. 30(2): p. 97-101. |
| 689 | Jin, X., Y.J. Ding, L.L. Wang, S.Q. Ding, L. Shu, Y.W. Jiang, et al., [Clinical study on acupuncture for treatment of chronic functional constipation]. Zhongguo Zhen Jiu, 2010. 30(2): p. 97-101. |
| 690 | Jin, D., Y. Liu, S. Lv, Q. Qi, M. Li, Y. Wang, et al., Electroacupuncture and Moxibustion Modulate the BDNF and TrkB Expression in the Colon and Dorsal Root Ganglia of IBS Rats with Visceral Hypersensitivity. Evidence-based Complementary and Alternative Medicine, 2021. 2021. |
| 691 | Jin, D., Y. Liu, S. Lv, Q. Qi, M. Li, Y. Wang, et al., Electroacupuncture and Moxibustion Modulate the BDNF and TrkB Expression in the Colon and Dorsal Root Ganglia of IBS Rats with Visceral Hypersensitivity. Evid Based Complement Alternat Med, 2021. 2021: p. 8137244. |
| 692 | Jiang, X.X., X.T. Guo, J.H. Zhou, and S.S. Ye, Acupuncture and Moxibustion in the Treatment of Adult Diarrhea Irritable Bowel Syndrome: A Network Meta-analysis. COMPUTATIONAL AND MATHEMATICAL METHODS IN MEDICINE, 2022. 2022. |
| 693 | Jiang, X., X. Guo, J. Zhou, and S. Ye, Acupuncture and Moxibustion in the Treatment of Adult Diarrhea Irritable Bowel Syndrome: A Network Meta-analysis. Computational and Mathematical Methods in Medicine, 2022. 2022. |
| 694 | Jiang, X., X. Guo, J. Zhou, and S. Ye, Acupuncture and Moxibustion in the Treatment of Adult Diarrhea Irritable Bowel Syndrome: A Network Meta-analysis. Comput Math Methods Med, 2022. 2022: p. 9919839. |
| 695 | Ji, M.X., M.W. Guo, Y.S. Gao, Y. Lan, S. Wang, Y.F. Wang, et al., Comparison of effects of electroacupuncture at "Tianshu" (ST25) and "Dachangshu" (BL25) on intestinal sensitivity and expression of muscarinic M3R and 5-HT3AR in irritable bowel syndrome rats. Zhen ci yan jiu = Acupuncture research, 2019. 44(4): p. 264-269. |
| 696 | Ji, M.X., M.W. Guo, Y.S. Gao, Y. Lan, S. Wang, Y.F. Wang, et al., [Comparison of effects of electroacupuncture at "Tianshu" (ST25) and "Dachangshu" (BL25) on intestinal sensitivity and expression of muscarinic M(3)R and 5-HT(3A)R in irritable bowel syndrome rats]. Zhen Ci Yan Jiu, 2019. 44(4): p. 264-269. |
| 697 | Itmctr, Clinical observation of acupoint application in the treatment of diarrhea-type irritable bowel syndrome. https://trialsearch.who.int/Trial2.aspx?TrialID=ITMCTR2200006666, 2022. |
| 698 | Itmctr, A randomized controlled study of acupuncture in the treatment of diarrhea-predominant irritable bowel syndrome. https://trialsearch.who.int/Trial2.aspx?TrialID=ITMCTR2200006702, 2022. |
| 699 | Itmctr, The brain effect and mechanism of ''Shuji acupoint'' acupuncture on the brain functional connectivity network of PATIENTS with IBS based on the rs-fMRI technique. https://trialsearch.who.int/Trial2.aspx?TrialID=ITMCTR2200005558, 2022. |
| 700 | Itmctr, Clinical efficacy evaluation of taVNS for patients with IBS-D. https://trialsearch.who.int/Trial2.aspx?TrialID=ITMCTR2200006465, 2022. |
| 701 | Isrctn, Acupuncture for irritable bowel syndrome: a pilot for a randomised controlled trial. https://trialsearch.who.int/Trial2.aspx?TrialID=ISRCTN32823720, 2006. |
| 702 | Irct20191215045743N, Evaluation of the effectiveness of neurofeedback on irritable bowel syndrome. https://trialsearch.who.int/Trial2.aspx?TrialID=IRCT20191215045743N1, 2020. |
| 703 | Irct2015043022027N, Effect of acupressure on constipation of hemodialysis patients. https://trialsearch.who.int/Trial2.aspx?TrialID=IRCT2015043022027N1, 2015. |
| 704 | Hussain, Z. and E.M.M. Quigley, Systematic review: complementary and alternative medicine in the irritable bowel syndrome. ALIMENTARY PHARMACOLOGY & THERAPEUTICS, 2006. 23(4): p. 465-471. |
| 705 | Hussain, Z. and E.M.M. Quigley, Systematic review: Complementary and alternative medicine in the irritable bowel syndrome. Alimentary Pharmacology and Therapeutics, 2006. 23(4): p. 465-471. |
| 706 | Hussain, Z. and E.M. Quigley, Systematic review: Complementary and alternative medicine in the irritable bowel syndrome. Aliment Pharmacol Ther, 2006. 23(4): p. 465-471. |
| 707 | Hung, A., N. Kang, A. Bollom, J.L. Wolf, and A. Lembo, Complementary and Alternative Medicine Use Is Prevalent Among Patients with Gastrointestinal Diseases. Digestive Diseases and Sciences, 2015. 60(7): p. 1883-1888. |
| 708 | Hummelsberger, J., Comment. Deutsche Zeitschrift fur Akupunktur, 2010. 53(2): p. 41-42. |
| 709 | Hummelsberger, J., Components of placebo effect: Randomized controlled trial in patients with irritable bowel syndrome. Revista Internacional de Acupuntura, 2010. 4(3): p. 149-150. |
| 710 | Huangfu, Y.R., W. Peng, B.J. Guo, Z.F. Shen, L. Li, S.W. Liu, et al., Effects of acupuncture in treating insomnia due to spleen-stomach disharmony syndrome and its influence on intestinal microbiome: Study protocol for a randomized controlled trial. JOURNAL OF INTEGRATIVE MEDICINE-JIM, 2019. 17(3): p. 161-166. |
| 711 | Huang, Z.D., L.A. Liang, and W.X. Zhang, Acupuncture combined with massage for treatment of irritable bowel syndrome. Zhongguo zhen jiu = Chinese acupuncture & moxibustion, 2006. 26(10): p. 717-718. |
| 712 | Huang, Z.D., L.A. Liang, and W.X. Zhang, [Acupuncture combined with massage for treatment of irritable bowel syndrome]. Zhongguo Zhen Jiu, 2006. 26(10): p. 717-718. |
| 713 | Huang, Z., Z. Lin, C. Lin, H. Chu, X. Zheng, B. Chen, et al., Transcutaneous Electrical Acustimulation Improves Irritable Bowel Syndrome With Constipation by Accelerating Colon Transit and Reducing Rectal Sensation Using Autonomic Mechanisms. American Journal of Gastroenterology, 2022. 117(9): p. 1491-1501. |
| 714 | Huang, Z., Z. Lin, C. Lin, H. Chu, X. Zheng, B. Chen, et al., Transcutaneous Electrical Acustimulation Improves Irritable Bowel Syndrome With Constipation by Accelerating Colon Transit and Reducing Rectal Sensation Using Autonomic Mechanisms. American journal of gastroenterology, 2022. 117(9): p. 1491‐1501. |
| 715 | Huang, Z., Z. Lin, and N. Dai, NEEDLELESS TRANSCUTANEOUS ELECTRICAL ACUSTIMULATION IMPROVES IRRITABLE BOWEL SYNDROME WITH CONSTIPATION VIA AUTONOMIC MECHANISMS. Gastroenterology, 2019. 156(6): p. S-465. |
| 716 | Huang, Z., Z. Lin, and N. Dai, NEEDLELESS TRANSCUTANEOUS ELECTRICAL ACUSTIMULATION IMPROVES IRRITABLE BOWEL SYNDROME WITH CONSTIPATION VIA AUTONOMIC MECHANISMS. Gastroenterology, 2019. 156(6): p. S‐465. |
| 717 | Huang, M.C., H.R. Yen, C.L. Lin, Y.C. Lee, M.F. Sun, and M.Y. Wu, Acupuncture decreased the risk of stroke among patients with fibromyalgia in Taiwan: A nationwide matched cohort study. PLoS ONE, 2020. 15(10 October). |
| 718 | Huang, K.Y., F.Y. Wang, M. Lv, X.X. Ma, X.D. Tang, and L. Lv, Irritable bowel syndrome: Epidemiology, overlap disorders, pathophysiology and treatment. World Journal of Gastroenterology, 2023. 29(26): p. 4120-4135. |
| 719 | Huang, J., M. Lu, Y. Zheng, J. Ma, X. Ma, Y. Wang, et al., Quality of Evidence Supporting the Role of Acupuncture for the Treatment of Irritable Bowel Syndrome. Pain Research and Management, 2021. 2021. |
| 720 | Huang, C.S., Y.H. Sun, Y.T. Wang, Y.H. Pan, Y.C. Huang, C.M. Hsu, et al., Repeated transcutaneous electrical nerve stimulation of nonspecific acupoints of the upper body attenuates stress-induced visceral hypersensitivity in rats. Autonomic Neuroscience: Basic and Clinical, 2019. 220. |
| 721 | Huang, C.S., Y.H. Sun, Y.T. Wang, Y.H. Pan, Y.C. Huang, C.M. Hsu, et al., Repeated transcutaneous electrical nerve stimulation of nonspecific acupoints of the upper body attenuates stress-induced visceral hypersensitivity in rats. Auton Neurosci, 2019. 220: p. 102556. |
| 722 | Hu, P., K. Sun, H. Li, X. Qi, J. Gong, Y. Zhang, et al., Transcutaneous Electrical Acustimulation Improved the Quality of Life in Patients With Diarrhea-Irritable Bowel Syndrome. Neuromodulation, 2022. 25(8): p. 1165-1172. |
| 723 | Hu, P., K. Sun, H. Li, X. Qi, J. Gong, Y. Zhang, et al., Transcutaneous Electrical Acustimulation Improved the Quality of Life in Patients With Diarrhea-Irritable Bowel Syndrome. Neuromodulation, 2022. 25(8): p. 1165‐1172. |
| 724 | Hu, P., K. Sun, H. Li, X. Qi, J. Gong, Y. Zhang, et al., Transcutaneous Electrical Acustimulation Improved the Quality of Life in Patients With Diarrhea-Irritable Bowel Syndrome. Neuromodulation, 2022. 25(8): p. 1165-1172. |
| 725 | Hu, D.K.M.F.X.J. and P. Deng, Irritable bowel syndrome with diarrhea (IBS-D) treated with moxibustion on heat-sensitive acupoints: a randomized controlled trial. 2012. 22(2): p. 1‐5. |
| 726 | Hu, D., M.F. Kang, J. Xiong, and P. Deng, Irritable bowel syndrome with diarrhea (IBS-D) treated with moxibustion on heat-sensitive acupoints: A randomized controlled trial. World Journal of Acupuncture - Moxibustion, 2012. 22(2): p. 1-5. |
| 727 | Hu, D., M.F. Kang, J. Xiong, and P. Deng, Irritable bowel syndrome with diarrhea (IBS-D) treated with moxibustion on heat-sensitive acupoints: a randomized controlled trial. World journal of acupuncture - moxibustion, 2012. 22(2): p. 1‐5. |
| 728 | Hou, Y.J., K. Wang, H.L. Jiang, Y. Chen, J.P. Yao, Y. Li, et al., Study on the mechanism of electroacupuncture repairing intestinal barrier via regulating mast cell in rats with diarrhea-predominant irritable bowel syndrome. Zhen ci yan jiu = Acupuncture research, 2023. 48(3): p. 281-286. |
| 729 | Hou, Y.J., K. Wang, H.L. Jiang, Y. Chen, J.P. Yao, Y. Li, et al., [Study on the mechanism of electroacupuncture repairing intestinal barrier via regulating mast cell in rats with diarrhea-predominant irritable bowel syndrome]. Zhen Ci Yan Jiu, 2023. 48(3): p. 281-286. |
| 730 | Hou, Y., Y. Zhao, H. Jiang, K. Wang, W. Zhang, S. Zhou, et al., Maintenance of Intestinal Homeostasis in Diarrhea-Predominant Irritable Bowel Syndrome by Electroacupuncture Through Submucosal Enteric Glial Cell-Derived S-Nitrosoglutathione. Frontiers in Physiology, 2022. 13. |
| 731 | Hou, Y., Y. Zhao, H. Jiang, K. Wang, W. Zhang, S. Zhou, et al., Maintenance of Intestinal Homeostasis in Diarrhea-Predominant Irritable Bowel Syndrome by Electroacupuncture Through Submucosal Enteric Glial Cell-Derived S-Nitrosoglutathione. Front Physiol, 2022. 13: p. 917579. |
| 732 | Hosseini, A., S. Nikfar, and M. Abdollahi, Probiotics use to treat irritable bowel syndrome. Expert Opinion on Biological Therapy, 2012. 12(10): p. 1323-1334. |
| 733 | Hopkins, R.J., C. Howard, E. Hunter-Stitt, P.E. Kaptur, B. Pleune, D. Muse, et al., Phase 3 trial evaluating the immunogenicity and safety of a three-dose BioThrax® regimen for post-exposure prophylaxis in healthy adults. Vaccine, 2014. 32(19): p. 2217‐2224. |
| 734 | Hong, Z.M., Z.L. Wang, and X.J. Chen, [Therapeutic effect of acupoint catgut embedding on irritable bowel syndrome of diarrhea type]. Zhongguo zhen jiu = Chinese acupuncture & moxibustion, 2011. 31(4): p. 311-313. |
| 735 | Hong, Z.M., Z.L. Wang, and X.J. Chen, Therapeutic effect of acupoint catgut embedding on irritable bowel syndrome of diarrhea type. Zhongguo zhen jiu [Chinese acupuncture & moxibustion], 2011. 31(4): p. 311‐313. |
| 736 | Hong, Z.M., Z.L. Wang, and X.J. Chen, [Therapeutic effect of acupoint catgut embedding on irritable bowel syndrome of diarrhea type]. Zhongguo Zhen Jiu, 2011. 31(4): p. 311-313. |
| 737 | Homma, Y., T. Ueda, H. Tomoe, A.T.L. Lin, H.C. Kuo, M.H. Lee, et al., Clinical guidelines for interstitial cystitis and hypersensitive bladder updated in 2015. International Journal of Urology, 2016. 23(7): p. 542-549. |
| 738 | Heusser, S.A. and S.A. Pless, Acid-sensing ion channels as potential therapeutic targets. Trends in Pharmacological Sciences, 2021. 42(12): p. 1035-1050. |
| 739 | Herman, P.M., B.M. Craig, and O. Caspi, Is complementary and alternative medicine (CAM) cost-effective? A systematic review. BMC Complementary and Alternative Medicine, 2005. 5. |
| 740 | Herman, P.M., B.M. Craig, and O. Caspi, Is complementary and alternative medicine (CAM) cost-effective? A systematic review. BMC complementary and alternative medicine, 2005. 5. |
| 741 | Henningsen, P., S. Zipfel, H. Sattel, and F. Creed, Management of Functional Somatic Syndromes and Bodily Distress. Psychotherapy and Psychosomatics, 2018. 87(1): p. 12-31. |
| 742 | Henningsen, P., S. Zipfel, and W. Herzog, Management of functional somatic syndromes. Lancet, 2007. 369(9565): p. 946-955. |
| 743 | He, W., Y. Tong, Y. Zhao, L. Zhang, H. Ben, Q. Qin, et al., Review of controlled clinical trials on acupuncture versus sham acupuncture in Germany. Journal of Traditional Chinese Medicine, 2013. 33(3): p. 403-407. |
| 744 | Hasler, W.L., Irritable bowel syndrome and bloating. Best Practice and Research in Clinical Gastroenterology, 2007. 21(4): p. 689-707. |
| 745 | Harris, L.R. and L. Roberts, Treatments for irritable bowel syndrome: patients' attitudes and acceptability. BMC complementary and alternative medicine, 2008. 8: p. 65. |
| 746 | Harris, L.R. and L. Roberts, Treatments for irritable bowel syndrome: patients' attitudes and acceptability. BMC Complement Altern Med, 2008. 8: p. 65. |
| 747 | Harris, L.A., S. Hansel, J. DiBaise, and M.D. Crowell, Irritable bowel syndrome and chronic constipation: Emerging drugs, devices, and surgical treatments. Current Gastroenterology Reports, 2006. 8(4): p. 282-290. |
| 748 | Harris, L.A. and L. Chang, Irritable bowel syndrome: New and emerging therapies. Current Opinion in Gastroenterology, 2006. 22(2): p. 128-135. |
| 749 | Hao, L.J. and Z.M. Shi, [Therapeutic effect of herb-separated moxibustion at Jinsuo (GV 8)-eight-diagram points on diarrhea-type irritable bowel syndrome of liver stagnation and spleen deficiency]. Zhongguo Zhen Jiu, 2020. 40(7): p. 702-706. |
| 750 | Han, B.Y., Q.F. Shao, Y. Cong, S. Guo, X.Y. Mao, R.H. Wei, et al., Transcutaneous electric nerve stimulation over acupoints for patients with diarrhea-predominant irritable bowel syndrome Protocol for systematic review and meta-analysis. MEDICINE, 2018. 97(51). |
| 751 | Han, B.Y., Q.F. Shao, Y. Cong, S. Guo, X.Y. Mao, R.H. Wei, et al., Transcutaneous electric nerve stimulation over acupoints for patients with diarrhea-predominant irritable bowel syndrome Protocol for systematic review and meta-analysis. Medicine (United States), 2018. 97(51). |
| 752 | Han, B.Y., Q.F. Shao, Y. Cong, S. Guo, X.Y. Mao, R.H. Wei, et al., Transcutaneous electric nerve stimulation over acupoints for patients with diarrhea-predominant irritable bowel syndrome: Protocol for systematic review and meta-analysis. Medicine (Baltimore), 2018. 97(51): p. e13267. |
| 753 | Halland, M. and N.J. Talley, New treatments for IBS. Nature Reviews Gastroenterology and Hepatology, 2013. 10(1): p. 13-23. |
| 754 | Hall, L., The outer limits? Occupational Health, 2005. 57(SPEC. ISS.): p. 15-18. |
| 755 | Hall, K.T., A.J. Lembo, I. Kirsch, D.C. Ziogas, J. Douaiher, K.B. Jensen, et al., Catechol-O-Methyltransferase val158met Polymorphism Predicts Placebo Effect in Irritable Bowel Syndrome. PLoS ONE, 2012. 7(10). |
| 756 | Hajer, J., [Cannabis - therapy for the future?]. Vnitr Lek, 2015. 61(7-8): p. 680-685. |
| 757 | Hagege, H., Alternative treatments in irritable bowel syndrome. GASTROENTEROLOGIE CLINIQUE ET BIOLOGIQUE, 2009. 33: p. S79-S83. |
| 758 | Hagège, H., Alternative treatments in irritable bowel syndrome. Gastroenterologie Clinique et Biologique, 2009. 33(SUPPL. 1): p. S79-S83. |
| 759 | Gurian, M.B.F., A.M. De Souza, A.P.M. Da Silva, M.L.L. De Souza Montenegro, O.B. Poli Neto, F.J.C. Dos Reis, et al., Chronic pelvic pain of musculoskeletal cause in women. Expert Review of Obstetrics and Gynecology, 2012. 7(2): p. 149-157. |
| 760 | Gur, A. and P. Oktayoglu, Advances in diagnostic and treatment options in patients with fibromyalgia syndrome. Open Access Rheumatology: Research and Reviews, 2009. 1(1): p. 193-209. |
| 761 | Gupta, S., G. Schaffer, and M. Saps, Pediatric irritable bowel syndrome and other functional abdominal pain disorders: an update of non-pharmacological treatments. Expert Review of Gastroenterology and Hepatology, 2018. 12(5): p. 447-456. |
| 762 | Gupta, S., G. Schaffer, and M. Saps, Pediatric irritable bowel syndrome and other functional abdominal pain disorders: an update of non-pharmacological treatments. Expert Rev Gastroenterol Hepatol, 2018. 12(5): p. 447-456. |
| 763 | Guo, Y., W. Wei, and J. Chen, Effects and mechanisms of acupuncture and electroacupuncture for functional dyspepsia: A systematic review. WORLD JOURNAL OF GASTROENTEROLOGY, 2020. 26(19): p. 2440-2457. |
| 764 | Guo, X.X., J.F. Chen, Y. Lu, L.Y. Wu, Z.J. Weng, L. Yang, et al., Electroacupuncture at He-Mu points reduces P2X<inf>4</inf> receptor expression in visceral hypersensitivity. Neural Regeneration Research, 2013. 8(22): p. 2069-2077. |
| 765 | Guo, X.X., J.F. Chen, Y. Lu, L.Y. Wu, Z.J. Weng, L. Yang, et al., Electroacupuncture at He-Mu points reduces P2X4 receptor expression in visceral hypersensitivity. Neural Regeneration Research, 2013. 8(22): p. 2069-2077. |
| 766 | Guo, J.L., L. Yang, J. He, and Z.M. Yang, Comparison of therapeutic effects of different acupuncture and moxibustion therapies on irritable bowel syndrome A protocol for systematic review and network meta-analysis. MEDICINE, 2021. 100(35). |
| 767 | Guo, J.B., X.X. Xing, J.N. Wu, H. Zhang, Y.E. Yun, Z.S. Qin, et al., Acupuncture for Adults with Diarrhea-Predominant Irritable Bowel Syndrome or Functional Diarrhea: A Systematic Review and Meta-Analysis. NEURAL PLASTICITY, 2020. 2020. |
| 768 | Guo, J., L. Yang, J. He, and Z. Yang, Comparison of therapeutic effects of different acupuncture and moxibustion therapies on irritable bowel syndrome: A protocol for systematic review and network meta-analysis. Medicine (United States), 2021. 100(35). |
| 769 | Guo, J., L. Yang, J. He, and Z. Yang, Comparison of therapeutic effects of different acupuncture and moxibustion therapies on irritable bowel syndrome: A protocol for systematic review and network meta-analysis. Medicine (Baltimore), 2021. 100(35): p. e26920. |
| 770 | Guo, J., X. Xing, J. Wu, H. Zhang, Y. Yun, Z. Qin, et al., Acupuncture for adults with diarrhea-predominant irritable bowel syndrome or functional diarrhea: A systematic review and meta-analysis. Neural Plasticity, 2020. 2020. |
| 771 | Guo, J., X. Xing, J. Wu, H. Zhang, Y. Yun, Z. Qin, et al., Acupuncture for Adults with Diarrhea-Predominant Irritable Bowel Syndrome or Functional Diarrhea: A Systematic Review and Meta-Analysis. Neural Plast, 2020. 2020: p. 8892184. |
| 772 | Guo, J., J.H. Sun, L. Chen, H. Geng, G.H. Yang, R.R. Shen, et al., Bidirectional regulation of acupuncture: a subgroup analysis of multicenter randomized controlled trial of acupuncture with Tiaoshen Jianpi for irritable bowel syndrome. Zhongguo zhen jiu = Chinese acupuncture & moxibustion, 2021. 41(8): p. 845-850. |
| 773 | Guo, J., J.H. Sun, L. Chen, H. Geng, G.H. Yang, R.R. Shen, et al., [Bidirectional regulation of acupuncture: a subgroup analysis of multicenter randomized controlled trial of acupuncture with Tiaoshen Jianpi for irritable bowel syndrome]. Zhongguo Zhen Jiu, 2021. 41(8): p. 845-850. |
| 774 | Guo, J., J.H. Sun, L. Chen, H. Geng, G.H. Yang, R.R. Shen, et al., Bidirectional regulation of acupuncture: a subgroup analysis of multicenter randomized controlled trial of acupuncture with Tiaoshen Jianpi for irritable bowel syndrome. Zhongguo zhen jiu [Chinese acupuncture & moxibustion], 2021. 41(8): p. 845‐850. |
| 775 | Guo, J., J.H. Sun, L. Chen, H. Geng, X.L. Wu, Y.F. Song, et al., Correlation between curative effect and 5-HTTLPR polymorphism in treatment of diarrhea-predominant irritable bowel syndrome with acupuncture for regulating shen and strengthening spleen. Zhongguo zhen jiu = Chinese acupuncture & moxibustion, 2021. 41(4): p. 365-370. |
| 776 | Guo, J., J.H. Sun, L. Chen, H. Geng, X.L. Wu, Y.F. Song, et al., [Correlation between curative effect and 5-HTTLPR polymorphism in treatment of diarrhea-predominant irritable bowel syndrome with acupuncture for regulating shen and strengthening spleen]. Zhongguo Zhen Jiu, 2021. 41(4): p. 365-370. |
| 777 | Guo, J., J.H. Sun, L. Chen, H. Geng, X.L. Wu, Y.F. Song, et al., Correlation between curative effect and 5-HTTLPR polymorphism in treatment of diarrhea-predominant irritable bowel syndrome with acupuncture for regulating shen and strengthening spleen. Zhongguo zhen jiu [Chinese acupuncture & moxibustion], 2021. 41(4): p. 365‐370. |
| 778 | Guo, J., G. Lu, L. Chen, H. Geng, X. Wu, H. Chen, et al., Regulation of serum microRNA expression by acupuncture in patients with diarrhea-predominant irritable bowel syndrome. Acupuncture in medicine : journal of the British Medical Acupuncture Society, 2022. 40(1): p. 34-42. |
| 779 | Guo, J., G. Lu, L. Chen, H. Geng, X. Wu, H. Chen, et al., Regulation of serum microRNA expression by acupuncture in patients with diarrhea-predominant irritable bowel syndrome. Acupunct Med, 2022. 40(1): p. 34-42. |
| 780 | Gu, Y., Y.T. Lai, F.R. Chang, and C.Y. Chen, Utilization patterns and prescription characteristics of traditional Chinese medicine among patients with irritable bowel syndrome in Taiwan. Frontiers in Pharmacology, 2023. 14. |
| 781 | Grundmann, O. and S.L. Yoon, Complementary and alternative medicines in irritable bowel syndrome: An integrative view. WORLD JOURNAL OF GASTROENTEROLOGY, 2014. 20(2): p. 346-362. |
| 782 | Grundmann, O. and S.L. Yoon, Complementary and alternative medicines in irritable bowel syndrome: An integrative view. World Journal of Gastroenterology, 2014. 20(2): p. 346-362. |
| 783 | Grundmann, O. and S.L. Yoon, Complementary and alternative medicines in irritable bowel syndrome: an integrative view. World J Gastroenterol, 2014. 20(2): p. 346-362. |
| 784 | Grundmann, O. and S.L. Yoon, Irritable bowel syndrome: Epidemiology, diagnosis and treatment: An update for health-care practitioners. Journal of Gastroenterology and Hepatology (Australia), 2010. 25(4): p. 691-699. |
| 785 | Grundmann, O. and S.L. Yoon, Irritable bowel syndrome: epidemiology, diagnosis and treatment: an update for health-care practitioners. J Gastroenterol Hepatol, 2010. 25(4): p. 691-699. |
| 786 | Grover, M. and D.A. Drossman, Functional abdominal pain. Current Gastroenterology Reports, 2010. 12(5): p. 391-398. |
| 787 | Grover, M., When is irritable bowel syndrome not irritable bowel syndrome? diagnosis and treatment of chronic functional abdominal pain. Current Gastroenterology Reports, 2012. 14(4): p. 290-296. |
| 788 | Gros, M., P.O. Ndong, C. Jemaï, J.L. Toscano, B.M. Florance, and T. Piche, The role of complementary alternative medicines in the irritable bowel syndrome. Hepato-Gastro et Oncologie Digestive, 2022. 29(3): p. 368-378. |
| 789 | Greenwood, M.T., Dysbiosis, Spleen Qi, Phlegm, and Complex Difficulties. Medical Acupuncture, 2017. 29(3): p. 128-137. |
| 790 | Gray, M.G., B.R. Lackey, E.F. Patrick, S.L. Gray, and S.G. Hurley, Multiple Integrated Complementary Healing Approaches: Energetics & Light for bone. Medical Hypotheses, 2016. 86: p. 18-29. |
| 791 | Graham, L., ACG releases recommendations on the management of irritable bowel syndrome. American Family Physician, 2009. 79(12): p. 1108-1112. |
| 792 | Grace, S., L. Barnes, W. Reilly, A. Vlass, and P. de Permentier, An integrative review of dietetic and naturopathic approaches to functional bowel disorders. Complement Ther Med, 2018. 41: p. 67-80. |
| 793 | Gowan, J. and L. Roller, Women's health and irritable bowel syndrome. Australian Journal of Pharmacy, 2013. 94(1114): p. 70-74. |
| 794 | Godlee, F., Reclaiming the placebo effect. BMJ, 2008. 336(7651): p. 1P. |
| 795 | Go, G.Y. and H. Park, Effects of Auricular Acupressure on Women With Irritable Bowel Syndrome. Gastroenterology nursing : the official journal of the Society of Gastroenterology Nurses and Associates, 2020. 43(2): p. E24-E34. |
| 796 | Go, G.Y. and H. Park, Effects of Auricular Acupressure on Women With Irritable Bowel Syndrome. Gastroenterology nursing, 2020. 43(2): p. E24‐E34. |
| 797 | Go, G.Y. and H. Park, Effects of Auricular Acupressure on Women With Irritable Bowel Syndrome. Gastroenterol Nurs, 2020. 43(2): p. E24-e34. |
| 798 | Glickman-Simon, R. and K. Withy, Saw Palmetto, chinese red yeast extract, music therapy, Ginkgo biloba, increased practitioner interaction. Explore: The Journal of Science and Healing, 2012. 8(2): p. 138-140. |
| 799 | Glickman-Simon, R. and J. Wallace, Acupuncture for knee osteoarthritis, chasteberry for premenstrual syndrome, probiotics for irritable bowel syndrome, yoga for hypertension, and trigger point dry needling for plantar fasciitis. Explore: The Journal of Science and Healing, 2015. 11(2): p. 157-161. |
| 800 | Glickman-Simon, R. and S. Savasta, Acupuncture for acute stroke, peppermint oil for irritable bowel syndrome, yoga for depression and anxiety, hypnotherapy for smoking cessation, and lime juice for sickle cell anemia. Explore (NY), 2014. 10(4): p. 261-264. |
| 801 | Glickman-Simon, R. and A. Mukherji, MOXIBUSTION FOR ASTHMA, ACUPUNCTURE FOR EPILEPSY, PSYCHOLOGICAL THERAPIES FOR IRRITABLE BOWEL SYNDROME, EXERCISE TRAINING FOR MULTIPLE SCLEROSIS, AND COMFREY ROOT FOR ACUTE BACK PAIN. EXPLORE-THE JOURNAL OF SCIENCE AND HEALING, 2015. 11(1): p. 67-71. |
| 802 | Glickman-Simon, R. and A. Mukherji, Moxibustion for Asthma, Acupuncture for Epilepsy, Psychological Therapies for Irritable Bowel Syndrome, Exercise Training for Multiple Sclerosis, and Comfrey Root for Acute Back Pain. Explore: The Journal of Science and Healing, 2015. 11(1): p. 67-71. |
| 803 | Glickman-Simon, R. and B.S. Alper, Acupressure and postoperative vomiting, soy and breast cancer, gingko biloba and Alzheimer's disease, acupuncture and irritable bowel syndrome, mediterranean and low-carbohydrate diets. Explore: The Journal of Science and Healing, 2013. 9(2): p. 112-115. |
| 804 | Ghoshal, U.C., Drug treatment and novel therapies in patients with functional dyspepsia. Journal of Gastroenterology and Hepatology, 2012. 27: p. 15. |
| 805 | Ghosh, M. and K. Ojha, Medical and surgical management of chronic pelvic pain. Obstetrics, Gynaecology and Reproductive Medicine, 2011. 21(9): p. 249-253. |
| 806 | Geng, L.L., H. Huang, Y.C. Xuan, J.W. Wan, X.J. Yu, X.F. Nie, et al., [Hunyuan moxibustion for diarrhea-predominant irritable bowel syndrome of spleen and kidney yang deficiency: a randomized controlled trial]. Zhongguo Zhen Jiu, 2023. 43(9): p. 1028-1032. |
| 807 | Gendi, R. and N. Jahan, Pharmacological and Non-pharmacological Treatments of Irritable Bowel Syndrome and Their Impact on the Quality of Life: A Literature Review. Cureus, 2020. 12(7): p. e9324. |
| 808 | Ge, J.J. and K.X. Zeng, Efficacy observation on warm needling for 60 cases of diarrhea irritable bowel syndrome. World Journal of Acupuncture - Moxibustion, 2013. 23(4): p. 43-51+45. |
| 809 | Ge, J.J. and K.X. Zeng, Efficacy observation on warm needling for 60 cases of diarrhea irritable bowel syndrome. World journal of acupuncture - moxibustion, 2013. 23(4): p. 43‐51+45. |
| 810 | Garland, B., Patient's page. Southern Medical Journal, 2006. 99(11): p. 1317. |
| 811 | Gao, F., [Thirty two cases of diarrhea-predominant irritable bowel syndrome treated by ginger-partitioned moxibustion and acupoint application in dog days]. Zhongguo zhen jiu = Chinese acupuncture & moxibustion, 2014. 34(3): p. 218. |
| 812 | Gao, F., [Thirty two cases of diarrhea-predominant irritable bowel syndrome treated by ginger-partitioned moxibustion and acupoint application in dog days]. Zhongguo Zhen Jiu, 2014. 34(3): p. 218. |
| 813 | Gandhi, A., A. Shah, M.P. Jones, N. Koloski, N.J. Talley, M. Morrison, et al., Methane positive small intestinal bacterial overgrowth in inflammatory bowel disease and irritable bowel syndrome: A systematic review and meta-analysis. Gut Microbes, 2021. 13(1). |
| 814 | Gan, Y., S.L. Huang, M.Q. Luo, M. Chen, and H. Zheng, Acupuncture in addition to usual care for patients with irritable bowel syndrome: a component network meta-analysis. ACUPUNCTURE IN MEDICINE, 2022. 40(5): p. 403-414. |
| 815 | Gan, Y., S.L. Huang, M.Q. Luo, M. Chen, and H. Zheng, Acupuncture in addition to usual care for patients with irritable bowel syndrome: a component network meta-analysis. Acupuncture in medicine : journal of the British Medical Acupuncture Society, 2022. 40(5): p. 403-414. |
| 816 | Gan, Y., S.L. Huang, M.Q. Luo, M. Chen, and H. Zheng, Acupuncture in addition to usual care for patients with irritable bowel syndrome: a component network meta-analysis. Acupunct Med, 2022. 40(5): p. 403-414. |
| 817 | Gabuzian, K.S., K.A. Sarkisian, N.L. Grigorian, and Z.G. Azatian, Long-term results in the treatment of patients with irritable bowel syndrome. Klinicheskaia meditsina, 1994. 72(1): p. 47-48. |
| 818 | Gabuzian, K.S., K.A. Sarkisian, N.L. Grigorian, and Z.G. Azatian, Long-term results in the treatment of patients with irritable bowel syndrome. Klinicheskaia meditsina, 1994. 72(1): p. 47‐48. |
| 819 | Fung, F.Y. and Y.C. Linn, Developing traditional Chinese medicine in the era of evidence-based medicine: Current evidences and challenges. Evidence-based Complementary and Alternative Medicine, 2015. 2015. |
| 820 | Fu, Y.M., F.X. Liang, J. Li, S. Wu, and H. Wang, [Mechanism of acupuncture-moxibustion with "Biao-Ben" acupoint combination in treating irritable bowel syndrome rats by regulating serum metabolites and metabolic pathway based on TM widely targeted metabolomics method]. Zhen Ci Yan Jiu, 2023. 48(4): p. 339-346. |
| 821 | Fu, Y., H.F. Zhang, J. Xiong, L. Li, and M.F. Kang, [Clinical efficacy observation of heat-sensitive moxibustion with different doses for irritable bowel syndrome]. 2014. p. 45-48. |
| 822 | Fu, Y., H.F. Zhang, J. Xiong, L. Li, and M.F. Kang, Clinical efficacy observation of heat-sensitive moxibustion with different doses for irritable bowel syndrome. Zhongguo zhen jiu [Chinese acupuncture & moxibustion], 2014. 34(1): p. 45‐48. |
| 823 | Fu, Y., H.F. Zhang, J. Xiong, L. Li, and M.F. Kang, [Clinical efficacy observation of heat-sensitive moxibustion with different doses for irritable bowel syndrome]. Zhongguo Zhen Jiu, 2014. 34(1): p. 45-48. |
| 824 | Fu, Y., G.Y. Xu, J. Chen, and X.Z. P. Shi, Suppressed a-type K+ current accounts for enhanced excitability of colon specific sensory neurons in a rat model of irritable bowel syndrome: Therapeutic improvements by electroacupuncture. Gastroenterology, 2012. 142(5): p. S701. |
| 825 | Frøkjaer, J.B., S. Bergmann, C. Brock, A. Madzak, A.D. Farmer, J. Ellrich, et al., Modulation of vagal tone enhances gastroduodenal motility and reduces somatic pain sensitivity. Neurogastroenterology and motility, 2016. 28(4): p. 592‐598. |
| 826 | Ford, A.C. and N.J. Talley, Irritable bowel syndrome. BMJ (Online), 2012. 345(7873). |
| 827 | Ford, A.C. and P. Moayyedi, Dyspepsia. BMJ (Online), 2013. 347(7923). |
| 828 | Ford, A.C., Acupuncture for irritable bowel syndrome. Gastroenterology, 2012. 143(6): p. 1683-1684. |
| 829 | Forbes, A., S. Jackson, C. Walter, S. Quraishi, M. Jacyna, and M. Pitcher, Acupuncture for irritable bowel syndrome: A blinded placebo-controlled trial. World Journal of Gastroenterology, 2005. 11(26): p. 4040-4044. |
| 830 | Forbes, A., S. Jackson, C. Walter, S. Quraishi, M. Jacyna, and M. Pitcher, Acupuncture for irritable bowel syndrome: a blinded placebo-controlled trial. World journal of gastroenterology, 2005. 11(26): p. 4040‐4044. |
| 831 | Forbes, A., S. Jackson, C. Walter, S. Quraishi, M. Jacyna, and M. Pitcher, Acupuncture for irritable bowel syndrome: a blinded placebo-controlled trial. World J Gastroenterol, 2005. 11(26): p. 4040-4044. |
| 832 | Forbes, A., Irritable bowel syndrome: Alternative therapeutic management. Annals of Gastroenterology, 2002. 15(4): p. 340-343. |
| 833 | Forbes, A., Irritable bowel syndrome: Alternative therapeutic management. Annals of Gastroenterology, 2002. 15(3): p. 290-293. |
| 834 | Fireman, Z., A. Segal, Y. Kopelman, A. Sternberg, and R. Carasso, Acupuncture treatment for irritable bowel syndrome: A double-blind controlled study. Digestion, 2001. 64(2): p. 100-103. |
| 835 | Fireman, Z., A. Segal, Y. Kopelman, A. Sternberg, and R. Carasso, Acupuncture treatment for irritable bowel syndrome. A double-blind controlled study. Digestion, 2001. 64(2): p. 100‐103. |
| 836 | Finset, A., Clinician-patient interaction and health outcome: A potential impact on symptoms and quality of life in patients with pain? Patient Education and Counseling, 2012. 89(2): p. 217-218. |
| 837 | Finniss, D.G., T.J. Kaptchuk, F. Miller, and F. Benedetti, Biological, clinical, and ethical advances of placebo effects. LANCET, 2010. 375(9715): p. 686-695. |
| 838 | Finniss, D.G., T.J. Kaptchuk, F. Miller, and F. Benedetti, Biological, clinical, and ethical advances of placebo effects. The Lancet, 2010. 375(9715): p. 686-695. |
| 839 | Finniss, D.G., T.J. Kaptchuk, F. Miller, and F. Benedetti, Biological, clinical, and ethical advances of placebo effects. Lancet (london, england), 2010. 375(9715): p. 686‐695. |
| 840 | Feng, F., Q. Li, L.J. Liu, X.Y. Li, R. Jin, and B.H. Yan, The study of Sanfu-moxibustion with acupuncture and moxibustion in treating irritable bowel syndrome. Liaoning journal of traditional chinese medicine [liaoning zhong yi za zhi], 2015. 42(6): p. 1322‐1324. |
| 841 | Feman, S.P.C., L.I. Nguyen, M.T. Quilty, C.E. Kerr, B.H. Nam, L.A. Conboy, et al., Effectiveness of recruitment in clinical trials: An analysis of methods used in a trial for irritable bowel syndrome patients. CONTEMPORARY CLINICAL TRIALS, 2008. 29(2): p. 241-251. |
| 842 | Fassov, J.L., L. Lundby, S. Laurberg, S. Buntzen, and K. Krogh, A randomized, controlled, crossover study of sacral nerve stimulation for irritable bowel syndrome. Annals of surgery, 2014. 260(1): p. 31‐36. |
| 843 | Farzaei, M.H., R. Bahramsoltani, M. Abdollahi, and R. Rahimi, The role of visceral hypersensitivity in irritable bowel syndrome: Pharmacological targets and novel treatments. Journal of Neurogastroenterology and Motility, 2016. 22(4): p. 558-574. |
| 844 | Farmer, A.D., G. Amersinghe, C. Brock, A. Drewes, A.M. Drewes, and Q. Aziz, Electrical vagal nerve stimulation prevents the development of acid induced esophageal hyperalgesia. Neurogastroenterology and motility, 2016. 28 (Supplement 1): p. 48. |
| 845 | Farmer, A.D., G. Amersinghe, C. Brock, A. Drewes, A.M. Drewes, and Q. Aziz, Electrical vagal nerve stimulation prevents the development of acid induced esophageal hyperalgesia. Neurogastroenterology and motility, 2016. 28: p. 48. |
| 846 | Farhadi, A., K. Bruninga, J. Fields, and A. Keshavarzian, Irritable bowel syndrome: An update on therapeutic modalities. Expert Opinion on Investigational Drugs, 2001. 10(7): p. 1211-1222. |
| 847 | Farhadi, A., K. Bruninga, J. Fields, and A. Keshavarzian, Irritable bowel syndrome: an update on therapeutic modalities. Expert opinion on investigational drugs, 2001. 10(7): p. 1211‐1222. |
| 848 | Farhadi, A., K. Bruninga, J. Fields, and A. Keshavarzian, Irritable bowel syndrome: an update on therapeutic modalities. Expert Opin Investig Drugs, 2001. 10(7): p. 1211-1222. |
| 849 | Fábián, A., M. Rutka, T. Ferenci, R. Bor, A. Bálint, K. Farkas, et al., The use of complementary and alternative medicine is less frequent in patients with inflammatory bowel disease than in patients with other chronic gastrointestinal disorders. Gastroenterology Research and Practice, 2018. 2018. |
| 850 | Evans, S., M. Moieni, B. Sternlieb, J.C.I. Tsao, and L.K. Zeltzer, Yoga for Youth in Pain The UCLA Pediatric Pain Program Model. HOLISTIC NURSING PRACTICE, 2012. 26(5): p. 262-271. |
| 851 | Evans, S., Managing chronic pelvic pain in girls and women. Medicine Today, 2013. 14(5): p. 54-58. |
| 852 | Ernst, E., J. Snyder, and R.A. Dunlop, National Center for Complementary and Alternative Medicine-funded randomised controlled trials of acupuncture: A systematic review. Focus on Alternative and Complementary Therapies, 2012. 17(1): p. 15-21. |
| 853 | Ernst, E., M.H. Pittler, B. Wider, and K. Boddy, Acupuncture: Its evidence-base is changing. American Journal of Chinese Medicine, 2007. 35(1): p. 21-25. |
| 854 | Ernst, E., Errors of alternative medicine: Lessons for general practice. European Journal of General Practice, 2012. 18(1): p. 63-66. |
| 855 | Ernst, E., Acupuncture: What Does the Most Reliable Evidence Tell Us? Journal of Pain and Symptom Management, 2009. 37(4): p. 709-714. |
| 856 | Enck, P. and N. Mazurak, Nonmedicinal therapies of irritable bowel syndrome. Gastroenterologe, 2017. 12(2): p. 141-149. |
| 857 | Enck, P., U. Martens, and S. Klosterhalfen, The psyche and the gut. World Journal of Gastroenterology, 2007. 13(25): p. 3405-3408. |
| 858 | Enck, P., B. Horing, K. Weimer, and S. Klosterhalfen, Placebo responses and placebo effects in functional bowel disorders. European Journal of Gastroenterology and Hepatology, 2012. 24(1): p. 1-8. |
| 859 | El-Salhy, M., D. Gundersen, J.G. Hatlebakk, and T. Hausken, Irritable bowel syndrome: Treatment options. Clinical Practice, 2012. 9(5): p. 591-600. |
| 860 | Eckman, P., The diagnosis and treatment of cancer with constitutional conditional acupuncture. Journal of Chinese Medicine, 2021. 2021(125): p. 3-16. |
| 861 | Eberl, S., N.M. de Olivera, D. Bourne, K. Streitberger, P. Fockens, M.W. Hollmann, et al., Effect of electroacupuncture on sedation requirements during colonoscopy: a prospective placebo-controlled randomised trial. ACUPUNCTURE IN MEDICINE, 2020. 38(3): p. 131-139. |
| 862 | Drossman, D.A., C.B. Morris, S. Schneck, Y.J.B. Hu, N.J. Norton, W.F. Norton, et al., International survey of patients with IBS: Symptom features and their severity, health status, treatments, and risk taking to achieve clinical benefit. Journal of Clinical Gastroenterology, 2009. 43(6): p. 541-550. |
| 863 | Dossett, M.L., E.M. Cohen, and J. Cohen, Integrative Medicine for Gastrointestinal Disease. Primary Care - Clinics in Office Practice, 2017. 44(2): p. 265-280. |
| 864 | Dong, Y., D. Baumeister, S. Berens, W. Eich, and J. Tesarz, High Rates of Non-Response Across Treatment Attempts in Chronic Irritable Bowel Syndrome: Results From a Follow-Up Study in Tertiary Care. Frontiers in Psychiatry, 2019. 10. |
| 865 | Dong, T., X. Li, X. Ma, X. Xue, Y. Hou, Y. Liu, et al., Moxibustion for diarrhea-predominant irritable bowel syndrome: A protocol for systematic review and network meta-analysis. Medicine (Baltimore), 2021. 100(51): p. e28373. |
| 866 | Dong, J. and H. Zhang, Clinical observation of calming-intestine scattered acupoints applying for treating diarrhea-predominant irritable bowel syndrome. Journal of new chinese medicine [xin zhong yi], 2011. 43(8): p. 106‐107. |
| 867 | Dobarrio-Sanz, I., J.M. Hernandez-Padilla, M.M. Lopez-Rodriguez, C. Fernandez-Sola, J. Granero-Molina, and M.D. Ruiz-Fernandez, Non-pharmacological interventions to improve constipation amongst older adults in long-term care settings: A systematic review of randomised controlled trials. GERIATRIC NURSING, 2020. 41(6): p. 992-999. |
| 868 | Dinning, P.G. and S.M. Scott, Novel diagnostics and therapy of colonic motor disorders. Curr Opin Pharmacol, 2011. 11(6): p. 624-629. |
| 869 | Dinning, P. and S. Scott, Novel diagnostics and therapy of colonic motor disorders. Current Opinion in Pharmacology, 2011. 11(6): p. 624-629. |
| 870 | Ding, J.H., Z. Jin, X.X. Yang, J. Lou, W.X. Shan, Y.X. Hu, et al., Role of gut microbiota via the gut-liver-brain axis in digestive diseases. World Journal of Gastroenterology, 2020. 26(40): p. 6141-6162. |
| 871 | Diehl, D.L., Acupuncture for gastrointestinal and hepatobiliary disorders. JOURNAL OF ALTERNATIVE AND COMPLEMENTARY MEDICINE, 1999. 5(1): p. 27-45. |
| 872 | Diehl, D.L., Acupuncture for gastrointestinal and hepatobiliary disorders. Journal of Alternative and Complementary Medicine, 1999. 5(1): p. 27-45. |
| 873 | Deutsch, J.K., J. Levitt, and D.J. Hass, Complementary and Alternative Medicine for Functional Gastrointestinal Disorders. American Journal of Gastroenterology, 2020. 115(3): p. 350-364. |
| 874 | Deng, D.X., J. Tan, H. Zhang, G.L. Huang, S. Li, K.K. Guo, et al., Electroacupuncture Relieves Visceral Hypersensitivity by Down-regulating Mast Cell Number，PAR-2/TRPV 1 Signaling, etc. in Colonic Tissue of Rats with Irritable Bowel Syndrome. Zhen ci yan jiu = Acupuncture research, 2018. 43(8): p. 485-491. |
| 875 | Deng, D.X., J. Tan, H. Zhang, G.L. Huang, S. Li, K.K. Guo, et al., [Electroacupuncture Relieves Visceral Hypersensitivity by Down-regulating Mast Cell Number，PAR-2/TRPV 1 Signaling, etc. in Colonic Tissue of Rats with Irritable Bowel Syndrome]. Zhen Ci Yan Jiu, 2018. 43(8): p. 485-491. |
| 876 | Deng, D., K. Guo, J. Tan, G. Huang, S. Li, Q. Jiang, et al., Acupuncture for diarrhea-predominant irritable bowel syndrome:a meta-analysis. Zhongguo zhen jiu = Chinese acupuncture & moxibustion, 2017. 37(8): p. 907-912. |
| 877 | Deng, D., K. Guo, J. Tan, G. Huang, S. Li, Q. Jiang, et al., [Acupuncture for diarrhea-predominant irritable bowel syndrome:a meta-analysis]. Zhongguo Zhen Jiu, 2017. 37(8): p. 907-912. |
| 878 | Danis, P., A. Drew, S. Lingow, and S. Kurz, Evidence-based tools for premenstrual disorders. Journal of Family Practice, 2020. 69(1): p. E9-E17. |
| 879 | Daniela, G., P. Chiara, and C. Giuseppe, The possible role of ketogenic diet in fibromyalgia treatment. Pharmacologyonline, 2020(3 Special Issue): p. 122-126. |
| 880 | Dalrymple, J. and I. Bullock, Diagnosis and management of irritable bowel syndrome in adults in primary care: Summary of NICE guidance. BMJ, 2008. 336(7643): p. 556-558. |
| 881 | Dai, Y.Q., H. Weng, Q. Wang, X.J. Guo, Q. Wu, L. Zhou, et al., Moxibustion for diarrhea-predominant irritable bowel syndrome: A systematic review and meta-analysis of randomized controlled trials. COMPLEMENTARY THERAPIES IN CLINICAL PRACTICE, 2022. 46. |
| 882 | Dai, Y.Q., H. Weng, Q. Wang, X.J. Guo, Q. Wu, L. Zhou, et al., Moxibustion for diarrhea-predominant irritable bowel syndrome: A systematic review and meta-analysis of randomized controlled trials. Complementary therapies in clinical practice, 2022. 46: p. 101532. |
| 883 | Dai, Y.Q., H. Weng, Q. Wang, X.J. Guo, Q. Wu, L. Zhou, et al., Moxibustion for diarrhea-predominant irritable bowel syndrome: A systematic review and meta-analysis of randomized controlled trials. Complement Ther Clin Pract, 2022. 46: p. 101532. |
| 884 | Dai, Y.K., Y.B. Wu, R.L. Li, W.J. Chen, C.Z. Tang, L.M. Lu, et al., Efficacy and safety of non-pharmacological interventions for irritable bowel syndrome in adults. WORLD JOURNAL OF GASTROENTEROLOGY, 2020. 26(41): p. 6488-6509. |
| 885 | Dai, Y.K., Y.B. Wu, R.L. Li, W.J. Chen, C.Z. Tang, L.M. Lu, et al., Efficacy and safety of non-pharmacological interventions for irritable bowel syndrome in adults. World Journal of Gastroenterology, 2020. 26(41): p. 6488-6509. |
| 886 | Dai, Y.K., Y.B. Wu, R.L. Li, W.J. Chen, C.Z. Tang, L.M. Lu, et al., Efficacy and safety of non-pharmacological interventions for irritable bowel syndrome in adults. World J Gastroenterol, 2020. 26(41): p. 6488-6509. |
| 887 | Cymet, T.C., A practical approach to fibromyalgia. Journal of the National Medical Association, 2003. 95(4): p. 278-285. |
| 888 | Curtiss, F.R., Irritable bowel syndrome and antidepressants. Journal of Managed Care Pharmacy, 2008. 14(9): p. 882-885. |
| 889 | Cummings, M., The pros and cons of sham acupuncture. European Journal of Oriental Medicine, 2010. 6(4): p. 16-19. |
| 890 | Cummings, M., The pros and cons of sham acupuncture. European journal of oriental medicine, 2010. 6(4): p. 16‐19. |
| 891 | Cummings, M., Is sham acupuncture effective for irritable bowel syndrome? Commentary. Focus on Alternative and Complementary Therapies, 2009. 14(3): p. 192-193. |
| 892 | Cummings, M., Is sham acupuncture effective for irritable bowel syndrome? Commentary. Focus on alternative and complementary therapies, 2009. 14(3): p. 192‐193. |
| 893 | Conboy, L.A., R.H. Wasserman, E.E. Jacobson, R.B. Davis, A.T.R. Legedza, M. Park, et al., Investigating placebo effects in irritable bowel syndrome: A novel research design. CONTEMPORARY CLINICAL TRIALS, 2006. 27(2): p. 123-134. |
| 894 | Conboy, L.A., R.H. Wasserman, E.E. Jacobson, R.B. Davis, A.T. Legedza, M. Park, et al., Investigating placebo effects in irritable bowel syndrome: a novel research design. Contemp Clin Trials, 2006. 27(2): p. 123-134. |
| 895 | Conboy, L.A., R.H. Wasserman, E.E. Jacobson, R.B. Davis, A.T. Legedza, M. Park, et al., Investigating placebo effects in irritable bowel syndrome: a novel research design. Contemporary clinical trials, 2006. 27(2): p. 123‐134. |
| 896 | Comito, D., A. Famiani, S. Calamarà, S. Cardile, V. Ferrau, A. Chiaro, et al., Efficacy of complementary therapywith partially hydrolyzed guar gum (PHGG) in functional gastrointestinal disorders (FGID): A first pediatric randomized placebo controlled trial (RCT). Digestive and Liver Disease, 2011. 43: p. S443-S444. |
| 897 | Comito, D., A. Famiani, S. Calamara, S. Cardile, V. Ferrau, A. Chiaro, et al., Efficacy of complementary therapywith partially hydrolyzed guar gum (PHGG) in functional gastrointestinal disorders (FGID): a first pediatric randomized placebo controlled trial (RCT). Digestive and liver disease, 2011. 43: p. S443‐S444. |
| 898 | Coban, Ş., E. Akbal, S. Köklü, G. Köklü, M.A. Ulaşlı, S. Erkeç, et al., Clinical trial: transcutaneous interferential electrical stimulation in individuals with irritable bowel syndrome - a prospective double-blind randomized study. Digestion, 2012. 86(2): p. 86‐93. |
| 899 | Coban, Ş., E. Akbal, S. Köklü, G. Köklü, M.A. Ulaşlı, S. Erkeç, et al., Clinical trial: transcutaneous interferential electrical stimulation in individuals with irritable bowel syndrome - a prospective double-blind randomized study. Digestion, 2012. 86(2): p. 86-93. |
| 900 | Clauw, D.J., Fibromyalgia and related conditions. Mayo Clinic Proceedings, 2015. 90(5): p. 680-692. |
| 901 | Clauw, D.J., Fibromyalgia: An Overview. American Journal of Medicine, 2009. 122(12 SUPPL.): p. S3-S13. |
| 902 | Ciciora, S.L., V.O. Yildiz, W.Y. Jin, B. Zhao, and M. Saps, Complementary and Alternative Medicine Use in Pediatric Functional Abdominal Pain Disorders at a Large Academic Center. Journal of Pediatrics, 2020. 227: p. 53-59.e51. |
| 903 | Chung, Y. and R.C. Dumont, Complementary and alternative therapies: Use in pediatric pulmonary medicine. Pediatric Pulmonology, 2011. 46(6): p. 530-544. |
| 904 | Chung, V.C.H., H.L.C. Wong, X.Y. Wu, G.Y.G. Wen, R.S.T. Ho, J.Y.L. Ching, et al., Acupuncture and related therapies for treating irritable bowel syndrome: Overview of systematic reviews and network meta-analysis. BMC Complementary and Alternative Medicine, 2017. 17. |
| 905 | Chung, V.C.H., L.T.F. Ho, and I.X.Y. Wu, Chinese medicine diagnosis and treatment for COVID-2019: Is China ready for implementing a national guideline? Advances in Integrative Medicine, 2020. 7(2): p. 51-54. |
| 906 | Chunder, R., Non-ulcerative dyspepsia: Diagnosis and management. SA Pharmaceutical Journal, 2011. 78(2): p. 30-35. |
| 907 | Chu, W.C.W., J.C.Y. Wu, D.T.W. Yew, L. Zhang, L. Shi, D.K.W. Yeung, et al., Does acupuncture therapy alter activation of neural pathway for pain perception in irritable bowel syndrome?: A comparative study of true and sham acupuncture using functional magnetic resonance imaging. Journal of Neurogastroenterology and Motility, 2012. 18(3): p. 305-316. |
| 908 | Chu, W.C.W., J.C.Y. Wu, D.T.W. Yew, L. Zhang, L. Shi, D.K.W. Yeung, et al., Does acupuncture therapy alter activation of neural pathway for pain perception in irritable bowel syndrome?: a comparative study of true and sham acupuncture using functional magnetic resonance imaging. Journal of neurogastroenterology and motility, 2012. 18(3): p. 305‐316. |
| 909 | Chu, W.C., J.C. Wu, D.T. Yew, L. Zhang, L. Shi, D.K. Yeung, et al., Does acupuncture therapy alter activation of neural pathway for pain perception in irritable bowel syndrome?: a comparative study of true and sham acupuncture using functional magnetic resonance imaging. J Neurogastroenterol Motil, 2012. 18(3): p. 305-316. |
| 910 | Chu, W.C., J.C. Wu, D.T. Yew, L. Zhang, L. Shi, and D.K. Yeung, Does acupuncture therapy alter activation of neural pathway for pain perception in irritable bowel syndrome?: a comparative study of true and sham acupuncture using functional magnetic resonance imaging. Journal of neurogastroenterology and motility, 2012. 18(3): p. 305 // 316. |
| 911 | Chu, H.R., Z.H. Wang, J. Yang, H.B. Kong, and F. Li, Observation on therapeutic effect of pecking moxibustion of specific acupoints for treatment of irritable bowel syndrome (diarrhea type). Zhongguo zhen jiu = Chinese acupuncture & moxibustion, 2009. 29(2): p. 111-113. |
| 912 | Chu, H.R., Z.H. Wang, J. Yang, H.B. Kong, and F. Li, Observation on therapeutic effect of pecking moxibustion of specific acupoints for treatment of irritable bowel syndrome (diarrhea type). Zhongguo zhen jiu [Chinese acupuncture & moxibustion], 2009. 29(2): p. 111‐113. |
| 913 | Chu, H.R., Z.H. Wang, J. Yang, H.B. Kong, and F. Li, [Observation on therapeutic effect of pecking moxibustion of specific acupoints for treatment of irritable bowel syndrome (diarrhea type)]. Zhongguo Zhen Jiu, 2009. 29(2): p. 111-113. |
| 914 | Chu, H.R., Y. Wang, L. Tong, S.B. Wu, L.B. Wu, N. Li, et al., [Effect of moxibustion on TLR4/MyD88/NF-κB signaling pathway in colon of diarrhea-predo-minant irritable bowel syndrome rats]. Zhen Ci Yan Jiu, 2020. 45(8): p. 633-639. |
| 915 | Chu, H.R., N. Li, and H.L. Cheng, Observations on the therapeutic effect of warm needling moxibustion on irritable bowel syndrome of Liver Depression and Spleen Deficiency Type. Shanghai journal of acupuncture and moxibustion [shang hai zhen jiu za zhi], 2015. 34(5): p. 424‐425. |
| 916 | Chu, D., P. Cheng, H. Xiong, J. Zhang, S. Liu, and X. Hou, Electroacupuncture at ST-36 relieves visceral hypersensitivity and decreases 5-HT3 receptor level in the colon in chronic visceral hypersensitivity rats. International Journal of Colorectal Disease, 2011. 26(5): p. 569-574. |
| 917 | Chong, L.Y. and T. Treasure, Acupuncture to relieve the pain of thoracotomy: Commentary on randomized, controlled trial. Journal of Thoracic and Cardiovascular Surgery, 2008. 136(6): p. 1470-1471. |
| 918 | Chiou, E. and S. Nurko, Functional abdominal pain and irritable bowel syndrome in children and adolescents. Therapy, 2011. 8(3): p. 315-331. |
| 919 | Chiou, E. and S. Nurko, Management of functional abdominal pain and irritable bowel syndrome in children and adolescents. Expert Review of Gastroenterology and Hepatology, 2010. 4(3): p. 293-304. |
| 920 | Chin Feman, S.P., L.T. Nguyen, M.T. Quilty, C.E. Kerr, B.H. Nam, L.A. Conboy, et al., Effectiveness of recruitment in clinical trials: An analysis of methods used in a trial for irritable bowel syndrome patients. Contemporary Clinical Trials, 2008. 29(2): p. 241-251. |
| 921 | Chin Feman, S.P., L.T. Nguyen, M.T. Quilty, C.E. Kerr, B.H. Nam, L.A. Conboy, et al., Effectiveness of recruitment in clinical trials: an analysis of methods used in a trial for irritable bowel syndrome patients. Contemp Clin Trials, 2008. 29(2): p. 241-251. |
| 922 | ChiCtr, Neuroimaging study on the systemic regulatory effect mechanism of electroacupuncture at different frequencies in the treatment of irritable bowel syndrome. https://trialsearch.who.int/Trial2.aspx?TrialID=ChiCTR2300073808, 2023. |
| 923 | ChiCtr, Acupuncture treatment based on sensitive points for Irritable bowel syndrome with diarrhea : a randomised controlled trial. https://trialsearch.who.int/Trial2.aspx?TrialID=ChiCTR2300074179, 2023. |
| 924 | ChiCtr, Effect and mechanism of percutaneous electrical nerve stimulation on functional constipation in children. https://trialsearch.who.int/Trial2.aspx?TrialID=ChiCTR2200059549, 2022. |
| 925 | ChiCtr, Clinical observation of acupoint application in the treatment of diarrhea-type irritable bowel syndrome. https://trialsearch.who.int/Trial2.aspx?TrialID=ChiCTR2200064123, 2022. |
| 926 | ChiCtr, Study on Acupuncture Optimization Scheme of Refractory Diarrhea Irritable Bowel Syndrome Based on Orthogonal Experimental Design. https://trialsearch.who.int/Trial2.aspx?TrialID=ChiCTR2200064751, 2022. |
| 927 | ChiCtr, A randomized controlled study of acupuncture in the treatment of diarrhea-predominant irritable bowel syndrome. https://trialsearch.who.int/Trial2.aspx?TrialID=ChiCTR2200064619, 2022. |
| 928 | ChiCtr, Clinical efficacy and mechanism of action of one-finger acupressure on IBS-C based on. https://trialsearch.who.int/Trial2.aspx?TrialID=ChiCTR2200066417, 2022. |
| 929 | ChiCtr, Clinical efficacy evaluation of taVNS for patients with IBS-D. https://trialsearch.who.int/Trial2.aspx?TrialID=ChiCTR2200062593, 2022. |
| 930 | ChiCtr, To explore the neural network regulatory mechanism mediated by acupuncture in diarrhea-predominant irritable bowel syndrome based on resting-state functional MRI. http://www.who.int/trialsearch/Trial2.aspx?TrialID=ChiCTR2100043967, 2021. |
| 931 | ChiCtr, A randomized controlled trial on acupuncture treatment of diarrheal-predominant irritable bowel syndrome. https://trialsearch.who.int/Trial2.aspx?TrialID=ChiCTR2100044762, 2021. |
| 932 | ChiCtr, Exploring the relevance of serum MicroRNA199 alterations between knee osteoarthritis and diarrhea-predominant irritable bowel syndrome based on the efficacy of acupoints in acupuncture treatment. https://trialsearch.who.int/Trial2.aspx?TrialID=ChiCTR2100045999, 2021. |
| 933 | ChiCtr, Acupuncture based on specific acupoint theory for the treatment of irritable bowel syndrome: a randomized controlled pilot trial. https://trialsearch.who.int/Trial2.aspx?TrialID=ChiCTR2000030670, 2020. |
| 934 | ChiCtr, Randomized controlled trial of acupuncture treatment of Irritable Bowel Syndrome. https://trialsearch.who.int/Trial2.aspx?TrialID=ChiCTR2000041215, 2020. |
| 935 | ChiCtr, The efficacy and mechanisms of transcutaneous auricular vagus nerve stimulation on symptom in patients with IBS-C. http://www.who.int/trialsearch/Trial2.aspx?TrialID=ChiCTR2000029644, 2020. |
| 936 | ChiCtr, Evaluation of the Efficacy of Acupuncture for Irritable Bowel Syndrome with predominant Diarrhea (IBS-D). http://www.who.int/trialsearch/Trial2.aspx?TrialID=ChiCTR2000032382, 2020. |
| 937 | ChiCtr, Clinical study for the acupuncture with regulating mind and spleen regulation of brain structure and function in IBS-D patients based on multimodal fMRI. http://www.who.int/trialsearch/Trial2.aspx?TrialID=ChiCTR1900024734, 2019. |
| 938 | ChiCtr, Effect and central mechanism of acupuncture for constipation-type irritable bowel syndrome based on functional magnetic resonance imaging. https://trialsearch.who.int/Trial2.aspx?TrialID=ChiCTR1800019927, 2018. |
| 939 | ChiCtr, Treatment of irritable bowel syndrome with transcutaneous stimulation of the auricular branch of the vagal nerve (auricular t-VNS): a randomized, monocentric clinical trial on efficacy and the central mechanism of fMRI. https://trialsearch.who.int/Trial2.aspx?TrialID=ChiCTR1800014554, 2018. |
| 940 | Chiarioni, G., M. Pesce, A. Fantin, and G. Sarnelli, Complementary and alternative treatment in functional dyspepsia. UNITED EUROPEAN GASTROENTEROLOGY JOURNAL, 2018. 6(1): p. 5-12. |
| 941 | Chiarioni, G., M. Pesce, A. Fantin, and G. Sarnelli, Complementary and alternative treatment in functional dyspepsia. United European Gastroenterology Journal, 2018. 6(1): p. 5-12. |
| 942 | Chi, Y., M. Wang, and J.P. Liu, Eye Acupuncture as a Pain Relief Therapy: A Systematic Review of Randomized Controlled Trials. Advances in Integrative Medicine, 2019. 6: p. S130. |
| 943 | Chi, C.T., 1H NMR-based metabolic profiling study on acupuncture treating for functional intestinal disorders. https://trialsearch.who.int/Trial2.aspx?TrialID=ChiCTR-TRC-13003537, 2013. |
| 944 | Chi, C.T., A fMRI study on acupuncture treating for functional intestinal disorders. https://trialsearch.who.int/Trial2.aspx?TrialID=ChiCTR-TRC-13003241, 2013. |
| 945 | Chi, C.T., Research on clinical Effects of acupuncture for irritable bowel syndrome and the brain and intestine interaction regulation function. https://trialsearch.who.int/Trial2.aspx?TrialID=ChiCTR-TRC-12002904, 2012. |
| 946 | Chi, C.T., Clinical Research on acupuncture and moxibustion in teating Irritable bowel syndrome. https://trialsearch.who.int/Trial2.aspx?TrialID=ChiCTR-TRC-11001349, 2011. |
| 947 | Chi, C.T., Clinical Research on Fuzi-Partition Moxibustion in Treating Diarrhea Predominated Irritable Bowel Disease. https://trialsearch.who.int/Trial2.aspx?TrialID=ChiCTR-TRC-10000887, 2010. |
| 948 | Chi, C.I., Based on microRNA199-TRPV1, the intervention mechanism of acupuncture of regulationg the mind and strenthening the sleepen treatment on visceral hypersensitivity in IBS-D patients. https://trialsearch.who.int/Trial2.aspx?TrialID=ChiCTR-IOR-17010860, 2017. |
| 949 | Chi, C.I., A study for clinical efficacy and safety of Jianpiwenshen cataplasm preparation and acupoint application in treatment of IBS-D. https://trialsearch.who.int/Trial2.aspx?TrialID=ChiCTR-IIR-17012250, 2017. |
| 950 | Chi, C.I., Effect of Acupuncture for Regulating the Mind and Strenthening the Spleen on the Clinical Efficacy of IBS-D and the Expression of Serum MicroRNA. https://trialsearch.who.int/Trial2.aspx?TrialID=ChiCTR-IOR-17010861, 2017. |
| 951 | Chi, C.I., A Pragmatic Randomised Controlled Trial Comparing an Integrated Electroacupuncture Protocol vs Sham-control in Chinese Adults with Generalized Anxiety Disorder and Diarrhea-predominant Irritable Bowel Syndrome. https://trialsearch.who.int/Trial2.aspx?TrialID=ChiCTR-IPR-15005758, 2015. |
| 952 | Chi, C.I., The Effectiveness of Electroacupuncture for Abdominal pain symptoms of irritable bowel syndrome. https://trialsearch.who.int/Trial2.aspx?TrialID=ChiCTR-IPR-15006879, 2015. |
| 953 | Chi, C.I., Soothing Gan and Invigorating Pi acupuncture treatment for irritable bowel syndrome: a multicenter randomised controlled trial. https://trialsearch.who.int/Trial2.aspx?TrialID=ChiCTR-IOR-15006259, 2015. |
| 954 | Chey, W.D., M. Maneerattaporn, and R. Saad, Pharmacologic and complementary and alternative medicine therapies for irritable bowel syndrome. Gut and Liver, 2011. 5(3): p. 253-266. |
| 955 | Chey, W.D., J. Kurlander, and S. Eswaran, Irritable bowel syndrome: A clinical review. JAMA - Journal of the American Medical Association, 2015. 313(9): p. 949-958. |
| 956 | Cheung, D. and N. Trudgill, Managing a patient with burning mouth syndrome. Frontline Gastroenterology, 2015. 6(3): p. 218-222. |
| 957 | Cherniack, E.P., Use of complementary and alternative medicine to treat constipation in the elderly. Geriatrics and Gerontology International, 2013. 13(3): p. 533-538. |
| 958 | Cheng, S., H. Li, J. Luo, J. Chi, W. Zhao, J. Lin, et al., Egg yolk antibody combined with bismuth-based quadruple therapy in Helicobacter pylori infection rescue treatment: a single-center, randomized, controlled study. Frontiers in Microbiology, 2023. 14. |
| 959 | Chen, Y.H., X.K. Chen, and X.J. Yin, [Comparison of the therapeutic effects of electroacupuncture and probiotics combined with deanxit in treating diarrhea-predominant irritable bowel syndrome]. Zhongguo Zhong xi yi jie he za zhi Zhongguo Zhongxiyi jiehe zazhi = Chinese journal of integrated traditional and Western medicine / Zhongguo Zhong xi yi jie he xue hui, Zhongguo Zhong yi yan jiu yuan zhu ban, 2012. 32(5): p. 594-598. |
| 960 | Chen, Y.H., X.K. Chen, and X.J. Yin, Comparison of the therapeutic effects of electroacupuncture and probiotics combined with deanxit in treating diarrhea-predominant irritable bowel syndrome. Zhongguo zhong xi yi jie he za zhi zhongguo zhongxiyi jiehe zazhi = chinese journal of integrated traditional and western medicine, 2012. 32(5): p. 594‐598. |
| 961 | Chen, Y.H., X.K. Chen, and X.J. Yin, [Comparison of the therapeutic effects of electroacupuncture and probiotics combined with deanxit in treating diarrhea-predominant irritable bowel syndrome]. Zhongguo Zhong Xi Yi Jie He Za Zhi, 2012. 32(5): p. 594-598. |
| 962 | Chen, Y., Y. Zhao, L. Wang, J.P. Yao, Y. Li, and S.Y. Zhou, Involvement of Pirt /TRPV1 signaling in acupuncture-induced reduction of visceral hypersensitivity in diarrhea-predominant irritable bowel syndrome rats. Zhen ci yan jiu = Acupuncture research, 2021. 46(4): p. 278-283. |
| 963 | Chen, Y., Y. Zhao, L. Wang, J.P. Yao, Y. Li, and S.Y. Zhou, [Involvement of Pirt /TRPV1 signaling in acupuncture-induced reduction of visceral hypersensitivity in diarrhea-predominant irritable bowel syndrome rats]. Zhen Ci Yan Jiu, 2021. 46(4): p. 278-283. |
| 964 | Chen, Y., Y. Zhao, D.N. Luo, H. Zheng, Y. Li, and S.Y. Zhou, Electroacupuncture Regulates Disorders of Gut-Brain Interaction by Decreasing Corticotropin-Releasing Factor in a Rat Model of IBS. Gastroenterology Research and Practice, 2019. 2019. |
| 965 | Chen, Y., Y. Zhao, D.N. Luo, H. Zheng, Y. Li, and S.Y. Zhou, Electroacupuncture Regulates Disorders of Gut-Brain Interaction by Decreasing Corticotropin-Releasing Factor in a Rat Model of IBS. Gastroenterol Res Pract, 2019. 2019: p. 1759842. |
| 966 | Chen, X.Y., Y.H. Huang, and S.F. Yu, Regulating Liver and Spleen Regimen combined with acupuncture for treating diarrhea-predominant Irritable Bowel Syndrome in 50 Cases. Shanxi journal of traditional chinese medicine [shan xi zhong yi], 2005. 26(1): p. 32‐33. |
| 967 | Chen, K. and C. Li, Acupuncture combined with medication for ulcerative colitis with damp-heat syndrome at active phase. Zhongguo zhen jiu [Chinese acupuncture & moxibustion], 2015. 35(5): p. 435‐438. |
| 968 | Chen, J.D.Z., M. Ni, and J. Yin, Electroacupuncture treatments for gut motility disorders. Neurogastroenterology and Motility, 2018. 30(7). |
| 969 | Chen, G.Y., M. Liang, S.G. Xu, and D.L. Chen, Treating IBS with Chinese medicine combined with acupuncture point injection and psychotherapy in 82 cases. Chinese journal of integrated traditional and western medicine on digestion [zhong guo zhong xi yi jie he xiao hua za zhi], 2006. 14(2): p. 124‐125. |
| 970 | Chen, G.Y., M. Liang, D. Chen, and J.H. Hua, Ping Ji San combined with acupoints injection for treating irritable bowel syndrome in 70 cases. Chinese journal of traditional medical science and technology [zhong guo zhong yi yao ke ji], 2005. 12(5): p. 322‐323. |
| 971 | Chen, G.R., X.F. Xie, and C. Peng, Treatment of Irritable Bowel Syndrome by Chinese Medicine: A Review. Chinese Journal of Integrative Medicine, 2023. 29(4): p. 377-384. |
| 972 | Chen, G.G., S. Guo, X.L. Su, Q. Liu, and W. Wei, Research progress of moxibustion in treatment of irritable bowel syndrome. World Journal of Acupuncture - Moxibustion, 2021. 31(2): p. 136-140. |
| 973 | Chen, G., J.X. Luo, and X.Y. Hu, Tiaoshen acupuncture for diarrhea type IBS in patients with chronic hepatitis B (Liver stagnation and spleen deficiency syndrome): Curative effect and influence on plasma SP and VIP. World Chinese Journal of Digestology, 2015. 23(8): p. 1303-1307. |
| 974 | Chen, G., J.X. Luo, and X.Y. Hu, Tiaoshen acupuncture for diarrhea type IBS in patients with chronic hepatitis B (Liver stagnation and spleen deficiency syndrome): curative effect and influence on plasma SP and VIP. World chinese journal of digestology, 2015. 23(8): p. 1303‐1307. |
| 975 | Chen, B., Y. Ma, C. Zhong, Y. Li, J. Mo, S. Liang, et al., Suppression effect and safety of acupuncture on colonic spasm during colonoscopy: a randomized controlled trial. Journal of Gastrointestinal Oncology, 2022. 13(3): p. 1169-1177. |
| 976 | Chen, B., Y. Ma, C. Zhong, Y. Li, J. Mo, S. Liang, et al., Suppression effect and safety of acupuncture on colonic spasm during colonoscopy: a randomized controlled trial. Journal of gastrointestinal oncology, 2022. 13(3): p. 1169‐1177. |
| 977 | Chao, G.Q. and S. Zhang, Effectiveness of acupuncture to treat irritable bowel syndrome: A meta-analysis. WORLD JOURNAL OF GASTROENTEROLOGY, 2014. 20(7): p. 1871-1877. |
| 978 | Chao, G.Q. and S. Zhang, Effectiveness of acupuncture to treat irritable bowel syndrome: A meta-analysis. World Journal of Gastroenterology, 2014. 20(7): p. 1871-1877. |
| 979 | Chao, G.Q. and S. Zhang, Effectiveness of acupuncture to treat irritable bowel syndrome: a meta-analysis. World J Gastroenterol, 2014. 20(7): p. 1871-1877. |
| 980 | Chang, J.Y. and N.J. Talley, Current and emerging therapies in irritable bowel syndrome: From pathophysiology to treatment. Trends in Pharmacological Sciences, 2010. 31(7): p. 326-334. |
| 981 | Chang, F.Y. and C.L. Lu, Irritable bowel syndrome and migraine: Bystanders or partners? Journal of Neurogastroenterology and Motility, 2013. 19(3): p. 301-311. |
| 982 | Chang, F.Y. and C.L. Lu, Treatment of Irritable Bowel Syndrome Using Complementary and Alternative Medicine. Journal of the Chinese Medical Association, 2009. 72(6): p. 294-300. |
| 983 | Chang, F.Y. and C.L. Lu, Treatment of irritable bowel syndrome using complementary and alternative medicine. J Chin Med Assoc, 2009. 72(6): p. 294-300. |
| 984 | Chan, J., I. Carr, and J.F. Mayberry, The role of acupuncture in the treatment of irritable bowel syndrome: A pilot study. Hepato-Gastroenterology, 1997. 44(17): p. 1328-1330. |
| 985 | Chan, J., I. Carr, and J.F. Mayberry, The role of acupuncture in the treatment of irritable bowel syndrome: a pilot study. Hepatogastroenterology, 1997. 44(17): p. 1328-1330. |
| 986 | Chakraborty, S. and A.E. Bharucha, Commentary. Annals of Internal Medicine, 2016. 165(12): p. JC69. |
| 987 | Cannova, T.J., C. Avena-Woods, and T.Z. Jodlowski, Irritable bowel syndrome treatment options. 2012. p. 45-48. |
| 988 | Campbell, F. and B.J. Collett, Chronic pelvic pain. British Journal of Anaesthesia, 1994. 73(5): p. 571-573. |
| 989 | Camilleri, M., Diagnosis and Treatment of Irritable Bowel Syndrome: A Review. JAMA - Journal of the American Medical Association, 2021. 325(9): p. 865-877. |
| 990 | Camilleri, M., Editorial: is adequate relief fatally flawed or adequate as an end point in irritable bowel syndrome? Am J Gastroenterol, 2009. 104(4): p. 920-922. |
| 991 | Calandre, E.P. and F. Rico-Villademoros, The role of antipsychotics in the management of fibromyalgia. CNS Drugs, 2012. 26(2): p. 135-153. |
| 992 | Buskila, D., Pediatric Fibromyalgia. Rheumatic Disease Clinics of North America, 2009. 35(2): p. 253-261. |
| 993 | Buist, M., Micromassage: The round-headed needle. Journal of Chinese Medicine, 2017. 2017-June(114): p. 69-79. |
| 994 | Bruta, K., Vanshika, K. Bhasin, and Bhawana, The role of serotonin and diet in the prevalence of irritable bowel syndrome: a systematic review. Translational Medicine Communications, 2021. 6(1). |
| 995 | Brusaferro, A., E. Farinelli, L. Zenzeri, R. Cozzali, and S. Esposito, The Management of Paediatric Functional Abdominal Pain Disorders: Latest Evidence. Pediatric Drugs, 2018. 20(3): p. 235-247. |
| 996 | Brandt, L.J., W.D. Chey, A.E. Foxx-Orenstein, E.M.M. Quigley, L.R. Schiller, P.S. Schoenfeld, et al., An evidence-based position statement on the management of irritable bowel syndrome. American Journal of Gastroenterology, 2008. 104(SUPPL. 1): p. S1-S35. |
| 997 | Brabyn, S., J. Adamson, H. Macpherson, H. Tilbrook, and D.J. Torgerson, Short message service text messaging was feasible as a tool for data collection in a trial of treatment for irritable bowel syndrome. Journal of Clinical Epidemiology, 2014. 67(9): p. 993-1000. |
| 998 | Brabyn, S., J. Adamson, H. MacPherson, H. Tilbrook, and D.J. Torgerson, Short message service text messaging was feasible as a tool for data collection in a trial of treatment for irritable bowel syndrome. Journal of clinical epidemiology, 2014. 67(9): p. 993‐1000. |
| 999 | Brabyn, S., J. Adamson, H. MacPherson, H. Tilbrook, and D.J. Torgerson, Short message service text messaging was feasible as a tool for data collection in a trial of treatment for irritable bowel syndrome. J Clin Epidemiol, 2014. 67(9): p. 993-1000. |
| 1000 | Borten, P., Raw herb powders and powdered herb extracts in the treatment of chronic idiopathic pain: A methodology and case study. Journal of Chinese Medicine, 2009(89): p. 50-57. |
| 1001 | Boregowda, G. and H.A. Shehata, Gastrointestinal and liver disease in pregnancy. Best Practice and Research: Clinical Obstetrics and Gynaecology, 2013. 27(6): p. 835-853. |
| 1002 | Borah, B.J., J.M. Naessens, A.E. Glasgow, B.A. Bauer, and T.Y. Chon, Cost-effectiveness of acupuncture in an employee population: A retrospective analysis. COMPLEMENTARY THERAPIES IN MEDICINE, 2017. 31: p. 14-19. |
| 1003 | Bonetto, S., V. Boano, E. Valenzi, G.M. Saracco, and R. Pellicano, Non-pharmacological strategies to treat irritable bowel syndrome: 2022 update. Minerva Gastroenterology, 2022. 68(4): p. 475-481. |
| 1004 | Bonetto, S., V. Boano, E. Valenzi, G.M. Saracco, and R. Pellicano, Non-pharmacological strategies to treat irritable bowel syndrome: 2022 update. Minerva Gastroenterol (Torino), 2022. 68(4): p. 475-481. |
| 1005 | Bishop, F.L., E.E. Jacobson, J.R. Shaw, and T.J. Kaptchuk, Scientific tools, fake treatments, or triggers for psychological healing: How clinical trial participants conceptualise placebos. SOCIAL SCIENCE & MEDICINE, 2012. 74(5): p. 767-774. |
| 1006 | Bishop, F.L., E.E. Jacobson, J.R. Shaw, and T.J. Kaptchuk, Scientific tools, fake treatments, or triggers for psychological healing: How clinical trial participants conceptualise placebos. Social Science and Medicine, 2012. 74(5): p. 767-774. |
| 1007 | Bishop, F.L., E.E. Jacobson, J.R. Shaw, and T.J. Kaptchuk, Scientific tools, fake treatments, or triggers for psychological healing: how clinical trial participants conceptualise placebos. Social science & medicine (1982), 2012. 74(5): p. 767‐774. |
| 1008 | Bishop, F.L., E.E. Jacobson, J.R. Shaw, and T.J. Kaptchuk, Scientific tools, fake treatments, or triggers for psychological healing: how clinical trial participants conceptualise placebos. Soc Sci Med, 2012. 74(5): p. 767-774. |
| 1009 | Bishop, F.L., E.E. Jacobson, J. Shaw, and T.J. Kaptchuk, Participants' experiences of being debriefed to placebo allocation in a clinical trial. Qualitative health research, 2012. 22(8): p. 1138-1149. |
| 1010 | Bishop, F.L., E.E. Jacobson, J. Shaw, and T.J. Kaptchuk, Participants' experiences of being debriefed to placebo allocation in a clinical trial. Qual Health Res, 2012. 22(8): p. 1138-1149. |
| 1011 | Bishop, F., E. Jacobsen, J. Shaw, and T. Kaptchuk, Debriefing to placebo allocation: A phenomenological study of participants' experiences in a randomized clinical trial. European Journal of Integrative Medicine, 2010. 2(4): p. 199. |
| 1012 | Bishop, F., E. Jacobsen, J. Shaw, and T. Kaptchuk, Debriefing to placebo allocation: a phenomenological study of participants' experiences in a randomized clinical trial. European journal of integrative medicine, 2010. 2(4): p. 199. |
| 1013 | Birch, S., M.S. Lee, T. Alraek, and T.H. Kim, Overview of Treatment Guidelines and Clinical Practical Guidelines That Recommend the Use of Acupuncture: A Bibliometric Analysis. JOURNAL OF ALTERNATIVE AND COMPLEMENTARY MEDICINE, 2018. 24(8): p. 752-769. |
| 1014 | Billings, W., K. Mathur, H.J. Craven, H.P. Xu, and A. Shin, Potential Benefit With Complementary and Alternative Medicine in irritable Bowel Syndrome: A Systematic Review and Meta-analysis. CLINICAL GASTROENTEROLOGY AND HEPATOLOGY, 2021. 19(8): p. 1538-+. |
| 1015 | Billings, W., K. Mathur, H.J. Craven, H. Xu, and A. Shin, Potential Benefit With Complementary and Alternative Medicine in Irritable Bowel Syndrome: A Systematic Review and Meta-analysis. Clinical Gastroenterology and Hepatology, 2021. 19(8): p. 1538-1553.e1514. |
| 1016 | Billings, W., K. Mathur, H.J. Craven, H. Xu, and A. Shin, Potential Benefit With Complementary and Alternative Medicine in Irritable Bowel Syndrome: A Systematic Review and Meta-analysis. Clin Gastroenterol Hepatol, 2021. 19(8): p. 1538-1553.e1514. |
| 1017 | Bian, Z.X., Stratification by sex and subgroup is necessary for RCT on IBS. GUT, 2006. 55(5): p. 743-744. |
| 1018 | Bian, Z.X., Stratification by sex and subgroup is necessary for RCT on IBS [1]. Gut, 2006. 55(5): p. 743-744. |
| 1019 | Bethards, D.M. and A. Ouyang, Irritable Bowel Syndrome: Diagnosis and management. Practical Gastroenterology, 2007. 31(3): p. 14-40. |
| 1020 | Berle, C.A., D. Cobbin, N. Smith, and C. Zaslawski, A Novel Approach to Evaluate Traditional Chinese Medicine Treatment Outcomes Using Pattern Identification. JOURNAL OF ALTERNATIVE AND COMPLEMENTARY MEDICINE, 2010. 16(4): p. 357-367. |
| 1021 | Berle, C., D. Cobbin, N. Smith, and C. Zaslawski, An Innovative Method to Accommodate Chinese Medicine Pattern Diagnosis within the Framework of Evidence-Based Medical Research. CHINESE JOURNAL OF INTEGRATIVE MEDICINE, 2011. 17(11): p. 824-833. |
| 1022 | Benninga, M.A. and E.A. Mayer, The Power of Placebo in Pediatric Functional Gastrointestinal Disease. Gastroenterology, 2009. 137(4): p. 1207-1210. |
| 1023 | Benninga, M.A. and E.A. Mayer, The Power of Placebo in Pediatric Functional Gastrointestinal Disease. Gastroenterology, 2009. 137(4): p. 1207‐1210. |
| 1024 | Bennett, R., Fibromyalgia: Shining a light on fibromyalgia treatment. Nature Reviews Rheumatology, 2016. 12(10): p. 568-569. |
| 1025 | Bauer, B.A., S.C. Litin, and J.B. Bundrick, Clinical pearls in complementary and integrative medicine (CIM). Disease-a-Month, 2014. 60(7): p. 323-331. |
| 1026 | Bassett, J.T. and B.D. Cash, A review of irritable bowel syndrome and an update on therapeutic approaches. Expert Opinion on Pharmacotherapy, 2008. 9(7): p. 1129-1143. |
| 1027 | Bao, C.H., J.Z. Zhang, J.M. Liu, H.R. Liu, L.Y. Wu, Y. Shi, et al., Moxibustion treatment for diarrhea-predominant irritable bowel syndrome: study protocol for a randomized controlled trial. BMC COMPLEMENTARY AND ALTERNATIVE MEDICINE, 2016. 16. |
| 1028 | Bao, C.H., L.Y. Wu, Y. Shi, Z. Shi, X.M. Jin, J.C. Shen, et al., Long-term effect of moxibustion on irritable bowel syndrome with diarrhea: a randomized clinical trial. THERAPEUTIC ADVANCES IN GASTROENTEROLOGY, 2022. 15. |
| 1029 | Bao, C.H., C.Y. Wang, G.N. Li, Y.L. Yan, D. Wang, X.M. Jin, et al., Effect of mild moxibustion on intestinal microbiota and NLRP6 inflammasome signaling in rats with post-inflammatory irritable bowel syndrome. World J Gastroenterol, 2019. 25(32): p. 4696-4714. |
| 1030 | Bao, C., J. Zhang, J. Liu, H. Liu, L. Wu, Y. Shi, et al., Moxibustion treatment for diarrhea-predominant irritable bowel syndrome: Study protocol for a randomized controlled trial. BMC Complementary and Alternative Medicine, 2016. 16(1). |
| 1031 | Bao, C., J. Zhang, J. Liu, H. Liu, L. Wu, Y. Shi, et al., Moxibustion treatment for diarrhea-predominant irritable bowel syndrome: study protocol for a randomized controlled trial. BMC Complement Altern Med, 2016. 16(1): p. 408. |
| 1032 | Bao, C., J. Zhang, J. Liu, H. Liu, L. Wu, Y. Shi, et al., Moxibustion treatment for diarrhea-predominant irritable bowel syndrome: study protocol for a randomized controlled trial. BMC complementary and alternative medicine, 2016. 16(1): p. 408. |
| 1033 | Bao, C., L. Wu, Y. Shi, Z. Shi, X. Jin, J. Shen, et al., Long-term effect of moxibustion on irritable bowel syndrome with diarrhea: a randomized clinical trial. Therapeutic Advances in Gastroenterology, 2022. 15. |
| 1034 | Bao, C., L. Wu, Y. Shi, Z. Shi, X. Jin, J. Shen, et al., Long-term effect of moxibustion on irritable bowel syndrome with diarrhea: a randomized clinical trial. Therap Adv Gastroenterol, 2022. 15: p. 17562848221075131. |
| 1035 | Bao, C., L. Wu, Y. Shi, Z. Shi, X. Jin, J. Shen, et al., Long-term effect of moxibustion on irritable bowel syndrome with diarrhea: a randomized clinical trial. Therapeutic advances in gastroenterology, 2022. 15: p. 17562848221075131. |
| 1036 | Banerjee, S., Irritable bowel syndrome. Journal of Pain and Palliative Care Pharmacotherapy, 2010. 24(3): p. 271-274. |
| 1037 | Ballou, S., T.J. Kaptchuk, W. Hirsch, J. Nee, J. Iturrino, K.T. Hall, et al., Open-label versus double-blind placebo treatment in irritable bowel syndrome: study protocol for a randomized controlled trial. TRIALS, 2017. 18. |
| 1038 | Ballou, S., A. Beath, T.J. Kaptchuk, W. Hirsch, T. Sommers, J. Nee, et al., Factors Associated With Response to Placebo in Patients With Irritable Bowel Syndrome and Constipation. CLINICAL GASTROENTEROLOGY AND HEPATOLOGY, 2018. 16(11): p. 1738-+. |
| 1039 | Ball, E. and K.S. Khan, Recent advances in understanding and managing chronic pelvic pain in women with special consideration to endometriosis. F1000Research, 2020. 9. |
| 1040 | Bai, Y.F., C. Gao, W.J. Li, Y. Du, and L.X. An, Transcutaneous electrical acupuncture stimulation (TEAS) for gastrointestinal dysfunction in adults undergoing abdominal surgery: study protocol for a prospective randomized controlled trial. TRIALS, 2020. 21(1). |
| 1041 | Asare, F., S. Sẗrsrud, and M. Simŕn, Meditation over medication for irritable bowel syndrome? on exercise and alternative treatments for irritable bowel syndrome. Current Gastroenterology Reports, 2012. 14(4): p. 283-289. |
| 1042 | Ao, X. and N. Wang, Auricular-plaster therapy for treatment of IBS. Journal of Traditional Chinese Medicine, 2004. 24(3): p. 166-167. |
| 1043 | Anastasi, J.K., D.J. McMahon, and G.H. Kim, Symptom Management for Irritable Bowel Syndrome A Pilot Randomized Controlled Trial of Acupuncture/Moxibustion. GASTROENTEROLOGY NURSING, 2009. 32(4): p. 243-255. |
| 1044 | Anastasi, J.K., D.J. McMahon, and G.H. Kim, Symptom management for irritable bowel syndrome: a pilot randomized controlled trial of acupuncture/moxibustion. Gastroenterology nursing : the official journal of the Society of Gastroenterology Nurses and Associates, 2009. 32(4): p. 243-255. |
| 1045 | Anastasi, J.K., D.J. McMahon, and G.H. Kim, Symptom management for irritable bowel syndrome: a pilot randomized controlled trial of acupuncture/moxibustion. Gastroenterology nursing, 2009. 32(4): p. 243‐255. |
| 1046 | Anastasi, J.K., D.J. McMahon, and G.H. Kim, Symptom management for irritable bowel syndrome: a pilot randomized controlled trial of acupuncture/moxibustion. Gastroenterol Nurs, 2009. 32(4): p. 243-255. |
| 1047 | Anastasi, J.K., B. Capili, J. Quinn, D.J. McMahon, and C. Scully, Irritable bowel syndrome subtype screening characteristics: Constipation subtype patient profiles explored. Gastroenterology Insights, 2012. 4(2): p. 69-72. |
| 1048 | Anastasi, J.K., B. Capili, J. Quinn, D.J. McMahon, and C. Scully, Irritable bowel syndrome subtype screening characteristics: constipation subtype patient profiles explored. Gastroenterology insights, 2012. 4(2): p. 69‐72. |
| 1049 | Anastasi, J.K., B. Capili, and M. Chang, Development of Acupuncture and Moxibustion Protocol in a Clinical Trial for Irritable Bowel Syndrome. JAMS Journal of Acupuncture and Meridian Studies, 2017. 10(1): p. 62-66. |
| 1050 | Anastasi, J.K., B. Capili, and M. Chang, Development of Acupuncture and Moxibustion Protocol in a Clinical Trial for Irritable Bowel Syndrome. Journal of acupuncture and meridian studies, 2017. 10(1): p. 62‐66. |
| 1051 | Anastasi, J.K., B. Capili, and M. Chang, Development of Acupuncture and Moxibustion Protocol in a Clinical Trial for Irritable Bowel Syndrome. J Acupunct Meridian Stud, 2017. 10(1): p. 62-66. |
| 1052 | An, G., N. Li, G. Zhai, H. Liu, J. Sun, and H. Liang, Evaluation of the therapeutic effect of acupuncture and moxibustion on irritable bowel syndrome. Shanghai journal of acupuncture and moxibustion, 2010. 29(6): p. 354‐356. |
| 1053 | Amsallem, F., S. Sanchez, X. Armoiry, and F. Mion, Effectiveness of Non-Pharmacological Interventions for Irritable Bowel Syndrome: A Systematic Review. EVIDENCE-BASED COMPLEMENTARY AND ALTERNATIVE MEDICINE, 2021. 2021. |
| 1054 | Amsallem, F., S. Sanchez, X. Armoiry, and F. Mion, Effectiveness of Non-Pharmacological Interventions for Irritable Bowel Syndrome: A Systematic Review. Evidence-based Complementary and Alternative Medicine, 2021. 2021. |
| 1055 | Amsallem, F., S. Sanchez, X. Armoiry, and F. Mion, Effectiveness of Non-Pharmacological Interventions for Irritable Bowel Syndrome: A Systematic Review. Evid Based Complement Alternat Med, 2021. 2021: p. 4404185. |
| 1056 | Ammoury, R.F., M.D.R. Pfefferkorn, and J.M. Croffie, Functional gastrointestinal disorders: Past and present. World Journal of Pediatrics, 2009. 5(2): p. 103-112. |
| 1057 | Almakadma, A.H., A. De Vol, M.S. Alabdaljabar, S. Aldosari, I. Muhsen, O. Alfreihi, et al., Complementary and alternative medicine use and its association with medication adherence in inflammatory bowel disease and other gastrointestinal diseases. Saudi Journal of Gastroenterology, 2023. 29(4): p. 233-239. |
| 1058 | Alammar, N. and E. Stein, Irritable Bowel Syndrome: What Treatments Really Work. Medical Clinics of North America, 2019. 103(1): p. 137-152. |
| 1059 | Adriani, A., Clinical key advances in non-pharmacological treatments for irritable bowel syndrome. Gazzetta Medica Italiana Archivio per le Scienze Mediche, 2021. 180(11): p. 629-631. |
| 1060 | Actrn, A pilot study of traditional acupuncture as an additional treatment for irritable bowel syndrome-diarrhoea. https://trialsearch.who.int/Trial2.aspx?TrialID=ACTRN12622000073707, 2022. |
| 1061 | Accarino, A.M., F. Azpiroz, and J.R. Malagelada, Selective dysfunction of mechanosensitive intestinal afferents in irritable bowel syndrome. Gastroenterology, 1995. 108(3): p. 636-643. |
| 1062 | Accarino, A.M., F. Azpiroz, and J.R. Malagelada, Selective dysfunction of mechanosensitive intestinal afferents in irritable bowel syndrome. Gastroenterology, 1995. 108(3): p. 636‐643. |
| 1063 | Aaron, L.A. and D. Buchwald, Chronic diffuse musculoskeletal pain, fibromyalgia and co-morbid unexplained clinical conditions. Bailliere's Best Practice and Research in Clinical Rheumatology, 2003. 17(4): p. 563-574. |
| 1064 | KIOM-SAR 2020 International Research Conference. Integrative Medicine Research, 2020. 9. |
| 1065 | Management of Bladder Pain Syndrome: Green-top Guideline No. 70. BJOG: An International Journal of Obstetrics and Gynaecology, 2017. 124(2): p. e46-e72. |
| 1066 | Poster - Gastroenterology. Journal of Gastroenterology and Hepatology (Australia), 2015. 30: p. 28-159. |
| 1067 | Oral. Journal of Gastroenterology and Hepatology (Australia), 2015. 30: p. 1-27. |
| 1068 | 5th European Congress for Integrative Medicine. European Journal of Integrative Medicine, 2012. 4. |
| 1069 | Irritable bowel syndrome: A mild disorder; Purely symptomatic treatment. Prescrire International, 2009. 18(100): p. 75-79. |
| 1070 | Irritable bowel syndrome: a mild disorder; purely symptomatic treatment. Prescrire Int, 2009. 18(100): p. 75-79. |
| 1071 | Chronic pelvic pain. Obstetrics and Gynecology, 2004. 103(3): p. 589-604. |
| 1072 | Clinical observation on combination of warm acupuncture and ear pressing for the treatment of 46 cases of irritable bowel syndrome. Hunan guiding journal of traditional chinese medicine [hu nan zhong yi yao dao bao], 2004. 10(3): p. 42. |
