# Supplementary material for: The effect of acupuncture on quality of life in patients with irritable bowel syndrome: A systematic review and meta-analysis
Source: PLoS One. 2025 Feb 13;20(2):e0314678. doi: 10.1371/journal.pone.0314678 (PMC11824959; doi:10.1371/journal.pone.0314678)
Supplement: S2 Table — (DOCX) [file pone.0314678.s002.docx]

**S2 Table.** List of Included and Excluded Studies.

| Included and excluded reasons | No. studies | References. |
| --- | --- | --- |
| Included studies | 14 | [1-14] |
| Study protocol | 55 | [15-52] |
| Study other than English language | 57 | [53-109] |
| Meta analysis | 28 | [110-137] |
| Animal study | 35 | [138-172] |
| Systematic review | 30 | [173-202] |
| No abstract | 116 | [203-318] |
| Irrelevant articles | 167 | [319-485] |
| Not related to inclusion criteria | 98 | [486-585] |
| Case study | 8 | [586-593] |
| Inappropriate intervention | 19 | [594-613] |
| Not original article | 15 | [614-626] |
| Without appropriate outcome | 11 | [627-638] |
| Review articles, conference articles | 41 | [639-680] |

**REFERENCES**

1. Qi, L.Y., J.W. Yang, S.Y. Yan, J.F. Tu, Y.F. She, Y. Li, et al., *Acupuncture for the Treatment of Diarrhea-Predominant Irritable Bowel Syndrome: A Pilot Randomized Clinical Trial.* JAMA Netw Open, 2022. 5(12): p. e2248817.

2. Hu, P., K. Sun, H. Li, X. Qi, J. Gong, Y. Zhang, et al., *Transcutaneous Electrical Acustimulation Improved the Quality of Life in Patients With Diarrhea-Irritable Bowel Syndrome.* Neuromodulation, 2022. 25(8): p. 1165-1172.

3. Pei, L., H. Geng, J. Guo, G. Yang, L. Wang, R. Shen, et al., *Effect of Acupuncture in Patients With Irritable Bowel Syndrome: a Randomized Controlled Trial.* Mayo Clinic proceedings, 2020. 95(8): p. 1671‐1683.

4. Mak, A.D.P., V.C.H. Chung, S.Y. Yuen, Y.K. Tse, S.Y.S. Wong, Y. Ju, et al., *Noneffectiveness of electroacupuncture for comorbid generalized anxiety disorder and irritable bowel syndrome.* Journal of Gastroenterology and Hepatology (Australia), 2019. 34(10): p. 1736-1742.

5. Lowe, C., A. Aiken, A.G. Day, W. Depew, and S.J. Vanner, *Sham acupuncture is as efficacious as true acupuncture for the treatment of IBS: A randomized placebo controlled trial.* Neurogastroenterol Motil, 2017. 29(7).

6. Zheng, H., Y. Li, W. Zhang, F. Zeng, S.Y. Zhou, H.B. Zheng, et al., *Electroacupuncture for patients with diarrhea-predominant irritable bowel syndrome or functional diarrhea: a randomized controlled trial.* Medicine, 2016. 95(24): p. e3884.

7. Li, H., L.X. Pei, J.L. Zhou, and J.H. Sun, *Controlled observation on the efficacy of acupuncture and western medicine on diarrhea-type irritable bowel syndrome.* World journal of acupuncture - moxibustion, 2013. 23(2): p. 11‐16.

8. MacPherson, H., H. Tilbrook, J.M. Bland, K. Bloor, S. Brabyn, H. Cox, et al., *Acupuncture for irritable bowel syndrome: primary care based pragmatic randomised controlled trial.* BMC gastroenterology, 2012. 12: p. 150.

9. Coban, Ş., E. Akbal, S. Köklü, G. Köklü, M.A. Ulaşlı, S. Erkeç, et al., *Clinical trial: transcutaneous interferential electrical stimulation in individuals with irritable bowel syndrome - a prospective double-blind randomized study.* Digestion, 2012. 86(2): p. 86-93.

10. Sun, J.H., X.L. Wu, C. Xia, L.Z. Xu, L.X. Pei, H. Li, et al., *Clinical evaluation of Soothing Gan and invigorating Pi acupuncture treatment on diarrhea-predominant irritable bowel syndrome.* Chin J Integr Med, 2011. 17(10): p. 780-785.

11. Lembo, A.J., L. Conboy, J.M. Kelley, R.S. Schnyer, C.A. McManus, M.T. Quilty, et al., *A treatment trial of acupuncture in IBS patients.* Am J Gastroenterol, 2009. 104(6): p. 1489-1497.

12. Schneider, A., C. Weiland, P. Enck, S. Joos, K. Streitberger, C. Maser-Gluth, et al., *Neuroendocrinological effects of acupuncture treatment in patients with irritable bowel syndrome.* Complement Ther Med, 2007. 15(4): p. 255-263.

13. Schneider, A., P. Enck, K. Streitberger, C. Weiland, S. Bagheri, S. Witte, et al., *Acupuncture treatment in irritable bowel syndrome.* Gut, 2006. 55(5): p. 649-654.

14. Forbes, A., S. Jackson, C. Walter, S. Quraishi, M. Jacyna, and M. Pitcher, *Acupuncture for irritable bowel syndrome: a blinded placebo-controlled trial.* World J Gastroenterol, 2005. 11(26): p. 4040-4044.

1. Tctr, *Efficacy and safety of mild moxibustion in the treatment of diarrhea-predominant irritable bowel syndrome (spleen deficiency and dampness excess syndrome): a study protocol for a randomized controlled trial.* https://trialsearch.who.int/Trial2.aspx?TrialID=TCTR20230523002, 2023.
2. Qi, L.Y., Y. Wang, L.Q. Wang, Y.F. She, G.X. Shi, Y. Li, et al., *Correction: acupuncture for the treatment of diarrheal-predominant irritable bowel syndrome: study protocol for a pilot randomized controlled trial (Trials, (2021), 22, 1, (253), 10.1186/s13063-021-05211-x).* Trials, 2023. **24**(1).
3. Yin, X., S. Li, M. Ouyang, R. Ou, Z. Chen, S. Wei, et al., *Acupoint application therapy for diarrhea-predominant irritable bowel syndrome: a protocol for systematic review and network meta-analysis.* Ann Palliat Med, 2022. **11**(12): p. 3785-3793.
4. Shi, Y.Z., K. Ye, M. Chen, X. Xie, X.Y. Fan, C.R. Xie, et al., *Acupuncture for irritable bowel syndrome: Study protocol of a prospective, multicentre, registry study in real-world settings.* European Journal of Integrative Medicine, 2022. **55**.
5. Qi, L.Y., J.W. Yang, S.Y. Yan, Y.F. She, H. Hu, Y. Li, et al., *Effect of acupuncture for diarrhea-predominant irritable bowel syndrome: study protocol for a randomized clinical trial.* Trials, 2022. **23**(1): p. 711.
6. Liu, C., T.T. Pang, S. Yin, J.H. Li, J.J. Yao, H.M. Li, et al., *- Curative efficiency and adverse events of alternative therapy and medicine for functional constipation in adults A protocol for systematic review and meta-analysis.* 2022. **- 101**(- 14).
7. Li, Y., S. Peng, F. Liang, S. Liu, and J. Li, *Effectiveness of acupuncture for irritable bowel syndrome: Protocol for a scoping review of systematic reviews and meta-analyses.* Medicine (Baltimore), 2022. **101**(29): p. e29218.
8. Zhong, L.L.D., T.F. Lam, W. Yang, Y. Zheng, Z. Lyu, and Z. Bian, *Electro-acupuncture for irritable bowel syndrome patients: study protocol for a single-blinded randomized sham-controlled clinical trial.* Trials, 2021. **22**(1): p. 619.
9. Zhao, J., M. Chen, X. Wang, K. Ye, S. Shi, H. Li, et al., *Efficacy of acupuncture in refractory irritable bowel syndrome: study protocol for a randomised controlled trial.* BMJ open, 2021. **11**(9): p. e045655.
10. Wang, Y.N., M.S. Sun, X.X. Ni, T. Tian, L. Liu, X. Li, et al., *- Comparison of Effects and Brain-Gut Regulatory Mechanisms of Acupuncture and Flunarizine for Migraine: Study Protocol for a Randomized Controlled Trial.* 2021. **- 2021**.
11. Sun, J.W., M.L. Sun, D. Li, J. Zhao, S.H. Shi, H.X. Li, et al., *Efficacy of acupuncture based on acupoint combination theory for irritable bowel syndrome: a study protocol for a multicenter randomized controlled trial.* Trials, 2021. **22**(1): p. 719.
12. Qi, L.Y., Y. Wang, L.Q. Wang, Y.F. She, G.X. Shi, Y. Li, et al., *Acupuncture for the treatment of diarrheal-predominant irritable bowel syndrome: study protocol for a pilot randomized controlled trial.* Trials, 2021. **22**(1): p. 253.
13. Li, H.Y., Y. Chen, Z.Y. Hu, Y. Yi, J. Ye, Y.L. Zhou, et al., *- Comparison of acupuncture and pinaverium bromide in the treatment of irritable bowel syndrome A protocol for systematic review and meta-analysis.* 2021. **- 100**(- 16).
14. Li, H.Y., Y. Chen, Z.Y. Hu, J.W. Jiang, J. Ye, Y.L. Zhou, et al., *- Effectiveness of acupuncture for anxiety and depression in irritable bowel syndrome A protocol for systematic review and meta-analysis.* 2021. **- 100**(- 8).
15. Li, H., Y. Chen, Z. Hu, Y. Yi, J. Ye, Y. Zhou, et al., *Comparison of acupuncture and pinaverium bromide in the treatment of irritable bowel syndrome: A protocol for systematic review and meta-analysis.* Medicine (Baltimore), 2021. **100**(16): p. e25604.
16. Guo, J., L. Yang, J. He, and Z. Yang, *Comparison of therapeutic effects of different acupuncture and moxibustion therapies on irritable bowel syndrome: A protocol for systematic review and network meta-analysis.* Medicine (Baltimore), 2021. **100**(35): p. e26920.
17. Dong, T., X. Li, X. Ma, X. Xue, Y. Hou, Y. Liu, et al., *Moxibustion for diarrhea-predominant irritable bowel syndrome: A protocol for systematic review and network meta-analysis.* Medicine (Baltimore), 2021. **100**(51): p. e28373.
18. Yao, C.J., Y.L. Li, M.J. Pu, L.H. Luo, and P.M. Feng, *- Traditional Chinese medicine for irritable bowel syndrome A protocol for meta-analysis.* 2020. **- 99**(- 48).
19. Wang, L., D. Wang, M.M. Xu, W. Cao, Y. Liu, T.H. Hou, et al., *- Effectiveness of different acupuncture courses for functional constipation A protocol for systematic review and network meta-analysis.* 2020. **- 99**(- 21).
20. Wang, F., S. He, J. Yan, L. Mai, and L. Yang, *Effects of herb-partitioned moxibustion for diarrhoea-predominant irritable bowel syndrome: A protocol for systematic review and meta-analysis.* Medicine (Baltimore), 2020. **99**(34): p. e21817.
21. Li, Y.L., C.J. Yao, R. Lei, F. Xie, Q. Xiong, L.H. Luo, et al., *Acupuncture combined with Tongxieyaofang for diarrhea-type irritable bowel syndrome: A protocol for meta-analysis.* Medicine (Baltimore), 2020. **99**(48): p. e23457.
22. Bai, Y.F., C. Gao, W.J. Li, Y. Du, and L.X. An, *- Transcutaneous electrical acupuncture stimulation (TEAS) for gastrointestinal dysfunction in adults undergoing abdominal surgery: study protocol for a prospective randomized controlled trial.* 2020. **- 21**(- 1).
23. Yin, T., Z.X. He, P.H. Ma, L.K. Hou, L. Chen, K.N. Xie, et al., *- Effect and cerebral mechanism of acupuncture treatment for functional constipation: study protocol for a randomized controlled clinical trial.* 2019. **- 20**.
24. Huangfu, Y.R., W. Peng, B.J. Guo, Z.F. Shen, L. Li, S.W. Liu, et al., *- Effects of acupuncture in treating insomnia due to spleen-stomach disharmony syndrome and its influence on intestinal microbiome: Study protocol for a randomized controlled trial.* 2019. **- 17**(- 3): p. - 166.
25. Zhang, Y., Z. Li, and F. Han, *Electroacupuncture for patients with irritable bowel syndrome: A systematic review and meta-analysis protocol.* Medicine (Baltimore), 2018. **97**(31): p. e11627.
26. Peng, Y., H. You, X. Chen, Y. Chen, Y. Yang, J. Huang, et al., *Effect of electroacupuncture at homotopic and heterotopic acupoints on abdominal pain in patients with irritable bowel syndrome: study protocol for a randomized controlled trial.* Trials, 2018. **19**(1): p. 559.
27. Pei, L.X., H. Geng, H. Chen, X.L. Wu, L. Chen, J.L. Zhou, et al., *Acupuncture for irritable bowel syndrome: study protocol for a multicenter randomized controlled trial.* Trials, 2018. **19**(1): p. 529.
28. Pei, L.X., H. Chen, J. Guo, L. Chen, X.L. Wu, W.L. Xu, et al., *- Effect of acupuncture and its influence on visceral hypersensitivity in IBS-D patients: Study protocol for a randomized controlled trial.* 2018. **- 97**(- 21).
29. Pei, L., H. Chen, J. Guo, L. Chen, X. Wu, W. Xu, et al., *Effect of acupuncture and its influence on visceral hypersensitivity in IBS-D patients: Study protocol for a randomized controlled trial.* Medicine (Baltimore), 2018. **97**(21): p. e10877.
30. Han, B.Y., Q.F. Shao, Y. Cong, S. Guo, X.Y. Mao, R.H. Wei, et al., *Transcutaneous electric nerve stimulation over acupoints for patients with diarrhea-predominant irritable bowel syndrome: Protocol for systematic review and meta-analysis.* Medicine (Baltimore), 2018. **97**(51): p. e13267.
31. Qin, Z., B. Li, J. Wu, J. Tian, S. Xie, Z. Mao, et al., *Acupuncture for chronic diarrhea in adults: Protocol for a systematic review.* Medicine (Baltimore), 2017. **96**(4): p. e5952.
32. Ballou, S., T.J. Kaptchuk, W. Hirsch, J. Nee, J. Iturrino, K.T. Hall, et al., *- Open-label versus double-blind placebo treatment in irritable bowel syndrome: study protocol for a randomized controlled trial.* 2017. **- 18**.
33. Anastasi, J.K., B. Capili, and M. Chang, *Development of Acupuncture and Moxibustion Protocol in a Clinical Trial for Irritable Bowel Syndrome.* J Acupunct Meridian Stud, 2017. **10**(1): p. 62-66.
34. Bao, C.H., J.Z. Zhang, J.M. Liu, H.R. Liu, L.Y. Wu, Y. Shi, et al., *- Moxibustion treatment for diarrhea-predominant irritable bowel syndrome: study protocol for a randomized controlled trial.* 2016. **- 16**.
35. Bao, C., J. Zhang, J. Liu, H. Liu, L. Wu, Y. Shi, et al., *Moxibustion treatment for diarrhea-predominant irritable bowel syndrome: study protocol for a randomized controlled trial.* BMC complementary and alternative medicine, 2016. **16**(1): p. 408.
36. Chi, C.I., *A Pragmatic Randomised Controlled Trial Comparing an Integrated Electroacupuncture Protocol vs Sham-control in Chinese Adults with Generalized Anxiety Disorder and Diarrhea-predominant Irritable Bowel Syndrome.* https://trialsearch.who.int/Trial2.aspx?TrialID=ChiCTR-IPR-15005758, 2015.
37. Zheng, C.H., G.Y. Huang, X.H. Xu, Y. Wang, M.M. Zhang, W. Wang, et al., *- Electro-acupuncture with different current intensities to treat functional constipation: a study protocol for a randomized controlled trial.* 2013. **- 14**.
38. MacPherson, H., M. Bland, K. Bloor, H. Cox, D. Geddes, A. Kang'ombe, et al., *Acupuncture for irritable bowel syndrome: a protocol for a pragmatic randomised controlled trial.* BMC gastroenterology, 2010. **10**: p. 63.
39. Zou, L., J.R. Ruan, J.Y. Chen, J.J. Wang, S.S. Zhu, L.M. Liao, et al., *[Moxibustion relieves colonic inflammation by up-regulating expression of miR-345-3p/miR-216a-5p and down-regulating NF-κB p65 in colonic tissue of rats with diarrhea-predominant irritable bowel syndrome].* Zhen Ci Yan Jiu, 2023. **48**(3): p. 226-232.
40. Zhou, X.L., H. Wang, J. Li, S. Wu, F. Wu, W. Lu, et al., *[Effect of acupuncture-moxibustion stimulation of combined "Biao-Ben" acupoints on autonomic nervous activity and related factors in rats with irritable bowel syndrome diarrhea].* Zhen Ci Yan Jiu, 2023. **48**(7): p. 635-642.
41. Zhao, Z.R., Y.X. Wang, F.Y. Xu, W.C. Zhang, Q.Y. Wang, and W. Huang, *[Herbal-moxa plaster for diarrhea type irritable bowel syndrome of spleen and kidney yang deficiency: a randomized controlled trial].* Zhongguo Zhen Jiu, 2023. **43**(6): p. 617-621.
42. Wang, Z.W., B.W. Chen, S.Z. Chen, L.L. Zhang, H.W. Sun, and X.L. Chang, *[Effect of electroacupuncture at "Zusanli" (ST36) and "Tianshu" (ST25) on intestinal function and autonomic nerve balance in rats with irritable bowel syndrome].* Zhen Ci Yan Jiu, 2023. **48**(2): p. 165-171.
43. Li, K.W., H.R. Chu, J.R. Ruan, J.J. Wang, J.Y. Chen, S.S. Zhu, et al., *[Effect of moxibustion on immune function homeostasis in rats with diarrhea irritable bowel syndrome based on SCF/c-kit signaling pathway].* Zhongguo Zhen Jiu, 2023. **43**(2): p. 177-185.
44. Hou, Y.J., K. Wang, H.L. Jiang, Y. Chen, J.P. Yao, Y. Li, et al., *[Study on the mechanism of electroacupuncture repairing intestinal barrier via regulating mast cell in rats with diarrhea-predominant irritable bowel syndrome].* Zhen Ci Yan Jiu, 2023. **48**(3): p. 281-286.
45. Geng, L.L., H. Huang, Y.C. Xuan, J.W. Wan, X.J. Yu, X.F. Nie, et al., *[Hunyuan moxibustion for diarrhea-predominant irritable bowel syndrome of spleen and kidney yang deficiency: a randomized controlled trial].* Zhongguo Zhen Jiu, 2023. **43**(9): p. 1028-1032.
46. Fu, Y.M., F.X. Liang, J. Li, S. Wu, and H. Wang, *[Mechanism of acupuncture-moxibustion with "Biao-Ben" acupoint combination in treating irritable bowel syndrome rats by regulating serum metabolites and metabolic pathway based on TM widely targeted metabolomics method].* Zhen Ci Yan Jiu, 2023. **48**(4): p. 339-346.
47. Zhu, S.S., J.J. Wang, L. Zou, J.Y. Chen, K.W. Li, L.M. Liao, et al., *[Anti-inflammation effect of moxibustion for rats with diarrhea-predominant irritable bowel syndrome based on multiple miRNAs regulating NF-κB signal pathway].* Zhongguo Zhen Jiu, 2022. **42**(6): p. 654-662.
48. Zhou, Z.X., H.F. Ma, Y.C. Yang, J.T. Chen, and Y.H. Feng, *[Effect of electroacupuncture on colonic motility and RhoA and ROCK protein expression in IBS-D rats].* Zhen Ci Yan Jiu, 2022. **47**(7): p. 611-616.
49. Zheng, X., M.J. Zhi, L.L. Zhao, L. Chen, Z.Q. He, S.S. Jing, et al., *[Effect mechanism of blistering moxibustion on visceral hypersensitivity of irritable bowel syndrome in mice based on 5-HT signal pathway].* Zhongguo Zhen Jiu, 2022. **42**(7): p. 773-778.
50. Yang, Y.C., Z.X. Zhou, T. Xue, Y.H. Feng, J.T. Chen, T.N. Wang, et al., *[Effect of electroacupuncture on visceral sensitivity and colonic NGF, TrkA, TRPV1 expression in IBS-D rats].* Zhongguo Zhen Jiu, 2022. **42**(12): p. 1395-1402.
51. Xuan, Y.C., J. Liu, Y.Y. Huang, X.Y. Lu, L.L. Geng, X.M. Jiang, et al., *[Therapeutic effect of long-snake moxibustion combined with western medication on diarrhea type irritable bowel syndrome of spleen and kidney yang deficiency].* Zhongguo Zhen Jiu, 2021. **41**(2): p. 133-136.
52. Sun, Y.Z., S.L. Wang, and T.Y. Yu, *[Tiaoshen acupuncture method combined with electroacupuncture for diarrhea-type irritable bowel syndrome : a randomized controlled trial].* Zhongguo Zhen Jiu, 2021. **41**(1): p. 13-16.
53. Shi, H., Y. Niu, Q. Huang, Z.M. Yang, and L. Yang, *[Diarrhea-predominant irritable bowel syndrome of spleen deficiency and damp excess treated with fire needling therapy with filiform needle and acupoint application therapy: a randomized controlled trial].* Zhongguo Zhen Jiu, 2021. **41**(9): p. 984-990.
54. Chen, Y., Y. Zhao, L. Wang, J.P. Yao, Y. Li, and S.Y. Zhou, *[Involvement of Pirt /TRPV1 signaling in acupuncture-induced reduction of visceral hypersensitivity in diarrhea-predominant irritable bowel syndrome rats].* Zhen Ci Yan Jiu, 2021. **46**(4): p. 278-283.
55. Yao, J.P., Y. Zhao, Y. Chen, L.P. Chen, X.M. Feng, Y. Li, et al., *[Effect of electroacupuncture on intestinal epithelial mucosal barrier function in rats with diarrhea-predominant irritable bowel syndrome].* Zhen Ci Yan Jiu, 2020. **45**(5): p. 357-362.
56. Wang, S.S., X.R. Wang, R.Y. Yang, Y. Xu, and M.Y. Li, *[Efficacy and mechanism of acupuncture combined with Tongxieyaofang for diarrhea-type irritable bowel syndrome of liver depression and spleen deficiency].* Zhongguo Zhen Jiu, 2020. **40**(6): p. 605-609.
57. Tong, L., L.B. Wu, N. Li, H.L. Cheng, R.L. Cai, and H.R. Chu, *[Moxibustion relieves abdominal hypersensitivity and diarrhea by regulating colonic 5-hydroxytryptamine signaling pathway in rats with diarrhea type irritable bowel syndrome].* Zhen Ci Yan Jiu, 2020. **45**(7): p. 535-540.
58. Qin, Y., M.W. Guo, Y. Lan, Y.F. Wang, S. Wang, M.X. Ji, et al., *[Effect of electroacupuncture of "Hegu" (LI4) and "Zusanli" (ST36) on intestinal sensitivity and motility in irritable bowel syndrome rats].* Zhen Ci Yan Jiu, 2020. **45**(4): p. 293-298.
59. Hao, L.J. and Z.M. Shi, *[Therapeutic effect of herb-separated moxibustion at Jinsuo (GV 8)-eight-diagram points on diarrhea-type irritable bowel syndrome of liver stagnation and spleen deficiency].* Zhongguo Zhen Jiu, 2020. **40**(7): p. 702-706.
60. Chu, H.R., Y. Wang, L. Tong, S.B. Wu, L.B. Wu, N. Li, et al., *[Effect of moxibustion on TLR4/MyD88/NF-κB signaling pathway in colon of diarrhea-predo-minant irritable bowel syndrome rats].* Zhen Ci Yan Jiu, 2020. **45**(8): p. 633-639.
61. Ji, M.X., M.W. Guo, Y.S. Gao, Y. Lan, S. Wang, Y.F. Wang, et al., *[Comparison of effects of electroacupuncture at "Tianshu" (ST25) and "Dachangshu" (BL25) on intestinal sensitivity and expression of muscarinic M(3)R and 5-HT(3A)R in irritable bowel syndrome rats].* Zhen Ci Yan Jiu, 2019. **44**(4): p. 264-269.
62. Wang, S., M.W. Guo, Y.S. Gao, X.X. Ren, Y. Lan, M.X. Ji, et al., *[Effect of Electroacupuncture at "Neiguan" (PC 6) and "Tianshu" (ST 25) for Colonic Motility and D 2 Receptor in Irritable Bowel Syndrome Rats].* Zhen Ci Yan Jiu, 2018. **43**(1): p. 25-29.
63. Pei, L.X., W. Zhang, Y.F. Song, H. Geng, L. Chen, X.L. Wu, et al., *[Electroacupuncture of "Tianshu" (ST 25) Suppresses Visceral Pain Possibly by Down-regulating Mast Cell Activation, and Tryptase and SP Expression in Rats with Post-infectious Irritable Bowel Syndrome].* Zhen Ci Yan Jiu, 2018. **43**(7): p. 419-423.
64. Li, K., M. Guo, L. Tan, X. Li, Y. Wu, Y. Lan, et al., *[Comparison of effects of electroacupuncture at "Dachangshu" (BL 25) or "Tianshu" (ST 25) on visceral sensitivity, c-kit and TRPV1 of irritable bowel syndrome rats].* Zhongguo Zhen Jiu, 2018. **38**(6): p. 625-629.
65. Deng, D.X., J. Tan, H. Zhang, G.L. Huang, S. Li, K.K. Guo, et al., *[Electroacupuncture Relieves Visceral Hypersensitivity by Down-regulating Mast Cell Number，PAR-2/TRPV 1 Signaling, etc. in Colonic Tissue of Rats with Irritable Bowel Syndrome].* Zhen Ci Yan Jiu, 2018. **43**(8): p. 485-491.
66. Zhang, H., F. Xie, H. Gong, H. Huang, S. Chen, M. Kang, et al., *[Effects of heat-sensitive moxibustion on HPA axis in rats with irritable bowel syndrome].* Zhongguo Zhen Jiu, 2017. **37**(12): p. 1315-1321.
67. Yang, D.Y., H. Wang, J. Li, S. Wu, J.M. Liu, D. Wang, et al., *[Effect of Electroacupuncture at "Zusanli" (ST 36) on Vimentin (A Kind of Cytoskeleton Protein Related to Smooth Muscle Contraction) in Rats with Diarrhea-predominant Irritable Bowel Syndrome].* Zhen Ci Yan Jiu, 2017. **42**(5): p. 402-406.
68. Tan, L.H., K.G. Li, Y.Y. Wu, Y. Lan, M.W. Guo, W.L. Zhu, et al., *[Effect of Electroacupuncture at "Yintang" (GV 29) and "Tianshu" (ST 25) on the Ethology and the Expression of TRPV 1 Receptor in Colon of Irritable Bowel Syndrome Rats].* Zhen Ci Yan Jiu, 2017. **42**(2): p. 136-140.
69. Qin, Y., W. Yi, S. Lin, C. Yang, and Z. Zhuang, *[Clinical effect of abdominal acupuncture for diarrhea irritable bowel syndrome].* Zhongguo Zhen Jiu, 2017. **37**(12): p. 1265-1268.
70. Li, J., J. Lu, J. Sun, Z. Ruan, D. Xu, H. Geng, et al., *[Acupuncture with regulating mind and spleen for diarrhea irritable bowel syndrome and sleep quality:a randomized controlled trial].* Zhongguo Zhen Jiu, 2017. **37**(1): p. 9-13.
71. Pei, L., L. Zhu, J. Sun, X. Wu, and L. Chen, *[Constipation predominant irritable bowel syndrome treated with acupuncture for regulating the mind and strengthening the spleen: a randomized controlled trial].* Zhongguo Zhen Jiu, 2015. **35**(11): p. 1095-1098.
72. Mearin, F., E. Rey, and A. Balboa, *[Functional and motor gastrointestinal disorders].* Gastroenterol Hepatol, 2015. **38 Suppl 1**: p. 3-12.
73. Hajer, J., *[Cannabis - therapy for the future?].* Vnitr Lek, 2015. **61**(7-8): p. 680-685.
74. Zhou, H.F., S.Q. Ding, Y.J. Ding, L.L. Wang, H. Liu, J. Fang, et al., *[Observation on effect characteristics of electroacupuncture for different types of functional constipation].* Zhongguo Zhen Jiu, 2014. **34**(5): p. 435-438.
75. Yang, M.X., L. Zhao, J. Yang, X.X. Cao, Z.N. Yu, and F.R. Liang, *[Bibliometrics analysis on researches of illness spectrum for acu-moxibustion therapy and prospect].* Zhen ci yan jiu = Acupuncture research / [Zhongguo yi xue ke xue yuan Yi xue qing bao yan jiu suo bian ji], 2014. **39**(3): p. 247-251.
76. Gao, F., *[Thirty two cases of diarrhea-predominant irritable bowel syndrome treated by ginger-partitioned moxibustion and acupoint application in dog days].* Zhongguo Zhen Jiu, 2014. **34**(3): p. 218.
77. Fu, Y., H.F. Zhang, J. Xiong, L. Li, and M.F. Kang, *[Clinical efficacy observation of heat-sensitive moxibustion with different doses for irritable bowel syndrome]*. 2014. p. 45-48.
78. Xing, L.Y., L.X. Qu, H. Chen, and S. Gao, *[Clinical observation on acupressure at Jiaji points for irritable bowel syndrome].* Zhongguo Zhen Jiu, 2013. **33**(8): p. 739-742.
79. Wu, X.L., Y.L. Wang, J.H. Sun, Y.Y. Shu, L.X. Pei, J.L. Zhou, et al., *[Clinical observation on acupuncture for diarrhea-predominant irritable bowel syndrome patients in syndrome of liver-stagnation and spleen-deficiency and its impact on Th1/Th2]*. 2013. p. 1057-1060.
80. Liu, M.R., R.F. Xiao, Z.P. Peng, H.N. Zuo, K. Zhu, and S.M. Wang, *[Effect of acupuncture at "Zusanli" (ST 36 and "Taichong" (LR 3) on gastrointestinal hormone levels in rats with diarrhea type irritable bowel syndrome].* Zhen Ci Yan Jiu, 2012. **37**(5): p. 363-368.
81. Liu, H.H., X.D. Liu, Y.J. Wang, H.Q. Guan, J.Y. Chai, J.R. Zhao, et al., *[Effects of acupoint area and non-acupoint area of eye-acupuncture on expressions of VIP and AQP 8 in colonic tissues in rats with D-IBS].* Zhongguo Zhen Jiu, 2012. **32**(10): p. 919-924.
82. Li, H., L.X. Pei, and J.L. Zhou, *[Comparative observation on therapeutic effects between acupuncture and western medication for diarrhea-predominant irritable bowel syndrome].* Zhongguo Zhen Jiu, 2012. **32**(8): p. 679-682.
83. Chen, Y.H., X.K. Chen, and X.J. Yin, *[Comparison of the therapeutic effects of electroacupuncture and probiotics combined with deanxit in treating diarrhea-predominant irritable bowel syndrome].* Zhongguo Zhong Xi Yi Jie He Za Zhi, 2012. **32**(5): p. 594-598.
84. Zhang, H.C., S.K. Han, and J.L. Tang, *[Fifty cases of irritable bowel syndrome of diarrhea type treated with scalp acupuncture].* Zhongguo Zhen Jiu, 2011. **31**(7): p. 605-606.
85. Song, S.Y., Y.J. Wang, D.S. Wang, and J.Y. Chai, *[Effects of oculo-acupuncture therapy on colonic serotonin reuptake transporter expression in rats with irritable bowel syndrome].* Zhen Ci Yan Jiu, 2011. **36**(2): p. 101-104, 115.
86. Shi, Z.M., Y.S. Zhu, Q.X. Wang, and M.N. Lei, *[Comparative study on irritable bowel syndrome treated with acupuncture and western medicine].* Zhongguo Zhen Jiu, 2011. **31**(7): p. 607-609.
87. Hong, Z.M., Z.L. Wang, and X.J. Chen, *[Therapeutic effect of acupoint catgut embedding on irritable bowel syndrome of diarrhea type].* Zhongguo Zhen Jiu, 2011. **31**(4): p. 311-313.
88. Wang, Y.J., D.S. Wang, H.Q. Guan, J. Wang, J.Y. Chai, J.R. Zhao, et al., *[Effects of eye-acupuncture therapy on serum and colonic SP and VIP contents in rats with irritable bowel syndrome].* Zhen Ci Yan Jiu, 2010. **35**(1): p. 8-11, 26.
89. Jin, X., Y.J. Ding, L.L. Wang, S.Q. Ding, L. Shu, Y.W. Jiang, et al., *[Clinical study on acupuncture for treatment of chronic functional constipation].* Zhongguo Zhen Jiu, 2010. **30**(2): p. 97-101.
90. Chu, H.R., Z.H. Wang, J. Yang, H.B. Kong, and F. Li, *[Observation on therapeutic effect of pecking moxibustion of specific acupoints for treatment of irritable bowel syndrome (diarrhea type)].* Zhongguo Zhen Jiu, 2009. **29**(2): p. 111-113.
91. Long, Z.R., C.H. Yu, Y. Yang, H.N. Wang, and X.X. Chi, *[Clinical observation on acupuncture combined with microorganism pharmaceutical preparations for treatment of irritable bowel syndrome of constipation type].* Zhongguo Zhen Jiu, 2006. **26**(6): p. 403-405.
92. Huang, Z.D., L.A. Liang, and W.X. Zhang, *[Acupuncture combined with massage for treatment of irritable bowel syndrome].* Zhongguo Zhen Jiu, 2006. **26**(10): p. 717-718.
93. Li, H.Y., Y. Chen, Z.Y. Hu, W. Chen, H.Y. Tang, Z.Y. Yu, et al., *[Meta analysis of acupuncture and moxibustion for anxiety and depression in irritable bowel syndrome].* Zhen Ci Yan Jiu, 2022. **47**(9): p. 821-829.
94. Deng, D., K. Guo, J. Tan, G. Huang, S. Li, Q. Jiang, et al., *[Acupuncture for diarrhea-predominant irritable bowel syndrome:a meta-analysis].* Zhongguo Zhen Jiu, 2017. **37**(8): p. 907-912.
95. Pei, L.X., X.C. Zhang, J.H. Sun, H. Geng, and X.L. Wu, *[Meta analysis of acupuncture-moxibustion in treatment of irritable bowel syndrome].* Zhongguo Zhen Jiu, 2012. **32**(10): p. 957-960.
96. Lin, D.Z., Y.X. Ou, L.L. Li, K.X. Wu, Q. Zhang, J.Y. Yan, et al., *- Acupuncture for postoperative gastrointestinal dysfunction in cancer: a systematic review and meta-analysis.* 2023. **- 13**.
97. Ye, Z., X.Q. Wei, S.Q. Feng, Q.H. Gu, J. Li, L. Kuai, et al., *- Effectiveness and safety of acupuncture for postoperative ileus following gastrointestinal surgery: A systematic review and meta-analysis.* 2022. **- 17**(- 7).
98. Yang, Y., K. Rao, K. Zhan, M. Shen, H. Zheng, S. Qin, et al., *Clinical evidence of acupuncture and moxibustion for irritable bowel syndrome: A systematic review and meta-analysis of randomized controlled trials.* Front Public Health, 2022. **10**: p. 1022145.
99. Wei, X., Y. Wen, Y. Wei, X. Liang, X. Ma, B. Zhang, et al., *External therapy of traditional Chinese medicine for treating irritable bowel syndrome with diarrhea: A systematic review and meta-analysis.* Front Med (Lausanne), 2022. **9**: p. 940328.
100. Wang, X., X. Shi, J. Lv, J. Zhang, Y. Huo, G. Zuo, et al., *Acupuncture and related therapies for the anxiety and depression in irritable bowel syndrome with diarrhea (IBS-D): A network meta-analysis of randomized controlled trials.* Front Psychiatry, 2022. **13**: p. 1067329.
101. Shi, Y.Z., Q.F. Tao, D. Qin, M. Chen, S.G. Yu, and H. Zheng, *Acupuncture vs. antispasmodics in the treatment of irritable bowel syndrome: An adjusted indirect treatment comparison meta-analysis.* Front Physiol, 2022. **13**: p. 1001978.
102. Lee, J.H., W.S. Sung, S.B. Bak, E.J. Kim, and Y.W. Kim, *Evaluation of Xiaoyao-san for treatment of irritable bowel syndrome: A systematic review and meta-analysis of randomized controlled trials.* European Journal of Integrative Medicine, 2022. **53**.
103. Jiang, X., X. Guo, J. Zhou, and S. Ye, *Acupuncture and Moxibustion in the Treatment of Adult Diarrhea Irritable Bowel Syndrome: A Network Meta-analysis.* Comput Math Methods Med, 2022. **2022**: p. 9919839.
104. Gan, Y., S.L. Huang, M.Q. Luo, M. Chen, and H. Zheng, *Acupuncture in addition to usual care for patients with irritable bowel syndrome: a component network meta-analysis.* Acupunct Med, 2022. **40**(5): p. 403-414.
105. Dai, Y.Q., H. Weng, Q. Wang, X.J. Guo, Q. Wu, L. Zhou, et al., *Moxibustion for diarrhea-predominant irritable bowel syndrome: A systematic review and meta-analysis of randomized controlled trials.* Complement Ther Clin Pract, 2022. **46**: p. 101532.
106. Wang, X.Y., H. Wang, Y.Y. Guan, R.L. Cai, and G.M. Shen, *Acupuncture for functional gastrointestinal disorders: A systematic review and meta-analysis.* J Gastroenterol Hepatol, 2021. **36**(11): p. 3015-3026.
107. Gandhi, A., A. Shah, M.P. Jones, N. Koloski, N.J. Talley, M. Morrison, et al., *Methane positive small intestinal bacterial overgrowth in inflammatory bowel disease and irritable bowel syndrome: A systematic review and meta-analysis.* Gut Microbes, 2021. **13**(1).
108. Billings, W., K. Mathur, H.J. Craven, H. Xu, and A. Shin, *Potential Benefit With Complementary and Alternative Medicine in Irritable Bowel Syndrome: A Systematic Review and Meta-analysis.* Clin Gastroenterol Hepatol, 2021. **19**(8): p. 1538-1553.e1514.
109. Wu, J., Q. Fu, S. Yang, H. Wang, and Y. Li, *Efficacy and safety of acupoint catgut embedding for diarrhea-predominant irritable bowel syndrome and constipation-predominant irritable bowel syndrome: A systematic review and meta-analysis.* Evidence-based Complementary and Alternative Medicine, 2020. **2020**.
110. Wang, L., M.M. Xu, Q.H. Zheng, W. Zhang, and Y. Li, *- The Effectiveness of Acupuncture in Management of Functional Constipation: A Systematic Review and Meta-Analysis.* 2020. **- 2020**.
111. Guo, J., X. Xing, J. Wu, H. Zhang, Y. Yun, Z. Qin, et al., *Acupuncture for Adults with Diarrhea-Predominant Irritable Bowel Syndrome or Functional Diarrhea: A Systematic Review and Meta-Analysis.* Neural Plast, 2020. **2020**: p. 8892184.
112. Zheng, H.Z., R.X. Chen, X.F. Zhao, G.H. Li, Y. Liang, H. Zhang, et al., *- Comparison between the Effects of Acupuncture Relative to Other Controls on Irritable Bowel Syndrome: A Meta-Analysis.* 2019. **- 2019**.
113. Zheng, H., R. Chen, X. Zhao, G. Li, Y. Liang, H. Zhang, et al., *Comparison between the Effects of Acupuncture Relative to Other Controls on Irritable Bowel Syndrome: A Meta-Analysis.* Pain Res Manag, 2019. **2019**: p. 2871505.
114. Zheng, H., Q. Chen, M. Chen, X. Wu, T.W. She, J. Li, et al., *- Nonpharmacological conservative treatments for chronic functional constipation: A systematic review and network meta-analysis.* 2019. **- 31**(- 1).
115. Yan, J., Z.W. Miao, J. Lu, F. Ge, L.H. Yu, W.B. Shang, et al., *Acupuncture plus Chinese Herbal Medicine for Irritable Bowel Syndrome with Diarrhea: A Systematic Review and Meta-Analysis.* Evid Based Complement Alternat Med, 2019. **2019**: p. 7680963.
116. Wu, I.X.Y., C.H.L. Wong, R.S.T. Ho, W.K.W. Cheung, A.C. Ford, J.C.Y. Wu, et al., *Acupuncture and related therapies for treating irritable bowel syndrome: overview of systematic reviews and network meta-analysis.* Therap Adv Gastroenterol, 2019. **12**: p. 1756284818820438.
117. Zhu, L., Y. Ma, S. Ye, and Z. Shu, *Acupuncture for Diarrhoea-Predominant Irritable Bowel Syndrome: A Network Meta-Analysis.* Evidence-based Complementary and Alternative Medicine, 2018. **2018**.
118. Zhu, L., Y. Ma, and X. Deng, *Comparison of acupuncture and other drugs for chronic constipation: A network meta-analysis.* PLoS ONE, 2018. **13**(4).
119. Park, J.Y., Y.K. Kim, S.Y. Kim, H. Lee, C.J. Choi, Y. Chae, et al., *Acupuncture modulates brain neural activity in patients: a systematic review and meta-analysis.* Oriental Pharmacy and Experimental Medicine, 2017. **17**(2): p. 111-126.
120. Chung, V.C.H., H.L.C. Wong, X.Y. Wu, G.Y.G. Wen, R.S.T. Ho, J.Y.L. Ching, et al., *Acupuncture and related therapies for treating irritable bowel syndrome: Overview of systematic reviews and network meta-analysis.* BMC Complementary and Alternative Medicine, 2017. **17**.
121. Chao, G.Q. and S. Zhang, *Effectiveness of acupuncture to treat irritable bowel syndrome: a meta-analysis.* World J Gastroenterol, 2014. **20**(7): p. 1871-1877.
122. Park, J.W., B.H. Lee, and H. Lee, *Moxibustion in the management of irritable bowel syndrome: systematic review and meta-analysis.* BMC Complement Altern Med, 2013. **13**: p. 247.
123. Manheimer, E., L.S. Wieland, K. Cheng, S.M. Li, X. Shen, B.M. Berman, et al., *Acupuncture for irritable bowel syndrome: systematic review and meta-analysis.* Am J Gastroenterol, 2012. **107**(6): p. 835-847; quiz 848.
124. Liu, X., J.N. Cao, T. Liu, H. Zhong, M. Liu, X.R. Chang, et al., *Effect of herb-partitioned moxibustion on structure and functional prediction of gut microbiota in rats with irritable bowel syndrome with diarrhea.* World Journal of Traditional Chinese Medicine, 2023. **9**(2): p. 141-149.
125. Qi, Q., H. Wu, X. Jin, D. Jin, Y. Wang, C. Wang, et al., *Effect of moxibustion on the expression of GDNF and its receptor GFRα3 in the colon and spinal cord of rats with irritable bowel syndrome.* Acupunct Med, 2019. **37**(4): p. 244-251.
126. Bao, C.H., C.Y. Wang, G.N. Li, Y.L. Yan, D. Wang, X.M. Jin, et al., *Effect of mild moxibustion on intestinal microbiota and NLRP6 inflammasome signaling in rats with post-inflammatory irritable bowel syndrome.* World J Gastroenterol, 2019. **25**(32): p. 4696-4714.
127. Wang, X., Q. Qi, Y. Wang, H. Wu, X. Jin, H. Yao, et al., *Gut microbiota was modulated by moxibustion stimulation in rats with irritable bowel syndrome.* Chin Med, 2018. **13**: p. 63.
128. Wang, X., Q. Qi, H. Wu, Y. Liu, Y. Wang, C. Wang, et al., *Moxibustion modulates the gut microbiota in rats with irritable bowel syndrome.* Gastroenterology, 2017. **152**(5): p. S632-S633.
129. Weng, Z.J., L.Y. Wu, C.L. Zhou, C.Z. Dou, Y. Shi, H.R. Liu, et al., *Effect of electroacupuncture on P2X3 receptor regulation in the peripheral and central nervous systems of rats with visceral pain caused by irritable bowel syndrome.* Purinergic Signal, 2015. **11**(3): p. 321-329.
130. Zhu, L., Z. Li, B. Xu, C. Xia, J. Cheng, X. Xiang, et al., *Effects of electroacupuncture at Zusanli (ST 36) on neurons in the colonic myenteric plexus in rats with irritable bowel syndrome with constipation.* Neural Regeneration Research, 2011. **6**(33): p. 2605-2609.
131. Wang, Y.J., H.H. Liu, X.D. Liu, J.Y. Chai, J.R. Zhao, and D.S. Wang, *Eye acupuncture therapy up-regulates aquaporin 3 expression in the colon of rats with diarrhea-predominant irritable bowel syndrome.* World Chinese Journal of Digestology, 2011. **19**(9): p. 899-904.
132. Xiao-Peng, M., L.Y. Tan, Y. Yang, H.G. Wu, B. Jiang, H.R. Liu, et al., *Effect of electro-acupuncture on substance P, its receptor and corticotropin-releasing hormone in rats with irritable bowel syndrome.* World Journal of Gastroenterology, 2009. **15**(41): p. 5211-5217.
133. Ma, X.P., L.Y. Tan, Y. Yang, H.G. Wu, B. Jiang, H.R. Liu, et al., *Effect of electro-acupuncture on substance P, its receptor and corticotropin-releasing hormone in rats with irritable bowel syndrome.* World J Gastroenterol, 2009. **15**(41): p. 5211-5217.
134. Sun, M., Y. Zhang, Y. Song, J. Guo, Y. Wang, C. Xin, et al., *Electroacupuncture alleviates water avoidance stress-induced irritable bowel syndrome in mice by improving intestinal barrier functions and suppressing the expression of inflammatory cytokines.* Journal of Traditional Chinese Medicine, 2023. **43**(3): p. 494-500.
135. Mengzhu, S., Z. Yujie, S. Yafang, G. Jing, W. Yuhang, X. Chen, et al., *Electroacupuncture alleviates water avoidance stress-induced irritable bowel syndrome in mice by improving intestinal barrier functions and suppressing the expression of inflammatory cytokines.* J Tradit Chin Med, 2023. **43**(3): p. 494-500.
136. Zhao, Y., H.L. Jiang, Y. Shi, W. Zhang, L.X. Zhang, Y.J. Hou, et al., *Electroacupuncture Alleviates Visceral Hypersensitivity in IBS-D Rats by Inhibiting EGCs Activity through Regulating BDNF/TrkB Signaling Pathway.* Evid Based Complement Alternat Med, 2022. **2022**: p. 2497430.
137. Sun, M., Y. Zhang, Y. Song, J. Guo, T. Zhao, Y. Wang, et al., *Electroacupuncture at Tianshu (ST25) and Zusanli (ST36) alleviates stress-induced irritable bowel syndrome in mice by modulating gut microbiota and corticotropin-releasing factor.* Journal of Traditional Chinese Medicine, 2022. **42**(5): p. 732-740.
138. Mengzhu, S., Z. Yujie, S. Yafang, G. Jing, Z. Tingting, W. Yuhang, et al., *Electroacupuncture at Tianshu (ST25) and Zusanli (ST36) alleviates stress-induced irritable bowel syndrome in mice by modulating gut microbiota and corticotropin-releasing factor.* J Tradit Chin Med, 2022. **42**(5): p. 732-740.
139. Jin, D., Y. Liu, S. Lv, Q. Qi, M. Li, Y. Wang, et al., *Electroacupuncture and Moxibustion Modulate the BDNF and TrkB Expression in the Colon and Dorsal Root Ganglia of IBS Rats with Visceral Hypersensitivity.* Evid Based Complement Alternat Med, 2021. **2021**: p. 8137244.
140. Yang, J., B. Shang, H. Shi, S. Zhu, G. Lu, and F. Dai, *The role of toll-like receptor 4 and mast cell in the ameliorating effect of electroacupuncture on visceral hypersensitivity in rats.* Neurogastroenterol Motil, 2019. **31**(6): p. e13583.
141. Tan, L.H., K.G. Li, Y.Y. Wu, M.W. Guo, Y. Lan, S. Wang, et al., *Effect of Electroacupuncture at Different Acupoints on the Expression of NMDA Receptors in ACC and Colon in IBS Rats.* Evidence-based Complementary and Alternative Medicine, 2019. **2019**.
142. Lv, P.R., Y.S. Su, W. He, X.Y. Wang, H. Shi, X.N. Zhang, et al., *Electroacupuncture Alleviated Referral Hindpaw Hyperalgesia via Suppressing Spinal Long-Term Potentiation (LTP) in TNBS-Induced Colitis Rats.* Neural Plast, 2019. **2019**: p. 2098083.
143. Huang, C.S., Y.H. Sun, Y.T. Wang, Y.H. Pan, Y.C. Huang, C.M. Hsu, et al., *Repeated transcutaneous electrical nerve stimulation of nonspecific acupoints of the upper body attenuates stress-induced visceral hypersensitivity in rats.* Auton Neurosci, 2019. **220**: p. 102556.
144. Zhang, Y., H. Zhao, Y. Wang, Y. Lu, and S. Wang, *Effect of Electroacupuncture Intervention on Constipation-predominant Irritable Bow l Syndrome and Colonic CGRP and SP Expression in Rats.* Zhen ci yan jiu = Acupuncture research / [Zhongguo yi xue ke xue yuan Yi xue qing bao yan jiu suo bian ji], 2016. **41**(1): p. 31-34.
145. Liang, C., K.Y. Wang, B. Xu, and Z. Yu, *- Electroacupuncture at acupoint ST 37(Shangjuxu) improves function of the enteric nervous system in a novel mouse constipation model.* 2016. **- 16**.
146. Liu, H.R., X.Y. Fang, H.G. Wu, L.Y. Wu, J. Li, Z.J. Weng, et al., *Effects of electroacupuncture on corticotropin-releasing hormone in rats with chronic visceral hypersensitivity.* World J Gastroenterol, 2015. **21**(23): p. 7181-7190.
147. Zhou, Y.Y., N.J. Wanner, Y. Xiao, X.Z. Shi, X.H. Jiang, J.G. Gu, et al., *Electroacupuncture alleviates stress-induced visceral hypersensitivity through an opioid system in rats.* World J Gastroenterol, 2012. **18**(48): p. 7201-7211.
148. Chu, D., P. Cheng, H. Xiong, J. Zhang, S. Liu, and X. Hou, *Electroacupuncture at ST-36 relieves visceral hypersensitivity and decreases 5-HT3 receptor level in the colon in chronic visceral hypersensitivity rats.* International Journal of Colorectal Disease, 2011. **26**(5): p. 569-574.
149. Zhang, J., H. Xiong, D. Chu, P. Cheng, W. Qian, and S. Liu, *Effect and mechanism of electroacupuncture at ST-36 on visceral hypersensitivity in rats.* Chinese Journal of Gastroenterology, 2010. **15**(11): p. 665-668.
150. Wang, X., H. Liu, G. Ding, Y. Chen, H. Wu, N. Li, et al., *Effects of electroacupuncture on c-Fos expression in the spinal cord and brain of rats with chronic visceral hypersensitivity.* Neural Regeneration Research, 2009. **4**(5): p. 339-343.
151. Liu, J., R. Peng, Q. Tan, B. Li, J. Chen, G. Liu, et al., *Proteomic analysis of rat colonic mucosa following acupuncture treatment for irritable bowel syndrome with diarrhea.* PLoS One, 2022. **17**(9): p. e0273853.
152. Xu, G.Y., J.H. Winston, and J.D.Z. Chen, *Electroacupuncture attenuates visceral hyperalgesia and inhibits the enhanced excitability of colon specific sensory neurons in a rat model of irritable bowel syndrome.* Neurogastroenterology and Motility, 2009. **21**(12): p. 1302-1308+e1125.
153. Fu, Y., G.Y. Xu, J. Chen, and X.Z. P. Shi, *Suppressed a-type K+ current accounts for enhanced excitability of colon specific sensory neurons in a rat model of irritable bowel syndrome: Therapeutic improvements by electroacupuncture.* Gastroenterology, 2012. **142**(5): p. S701.
154. Sun, J., X. Wu, Y. Meng, J. Cheng, H. Ning, Y. Peng, et al., *Electro-acupuncture decreases 5-HT, CGRP and increases NPY in the brain-gut axis in two rat models of Diarrhea-predominant irritable bowel syndrome(D-IBS).* BMC Complement Altern Med, 2015. **15**: p. 340.
155. Verástegui Escolano, C., *Electro-acupuncture decreases 5-HT, CGRP and increases NPY in the brain-gut axis in two rat models of Diarrhea-predominant irritable bowel syndrome(D-IBS).* Revista Internacional de Acupuntura, 2016. **10**(4): p. 155-157.
156. Chen, Y., Y. Zhao, D.N. Luo, H. Zheng, Y. Li, and S.Y. Zhou, *Electroacupuncture Regulates Disorders of Gut-Brain Interaction by Decreasing Corticotropin-Releasing Factor in a Rat Model of IBS.* Gastroenterol Res Pract, 2019. **2019**: p. 1759842.
157. Zhang, F., Z. Ma, Z. Weng, M. Zhao, H. Zheng, L. Wu, et al., *P2X3Receptor in Primary Afferent Neurons Mediates the Relief of Visceral Hypersensitivity by Electroacupuncture in an Irritable Bowel Syndrome Rat Model.* Gastroenterology Research and Practice, 2020. **2020**.
158. Li, X., K. Ren, X. Hong, S. Guo, S. Yu, and S. Yang, *Ameliorating effects of electroacupuncture on the low-grade intestinal inflammation in rat model of diarrhea-predominant irritable bowel syndrome.* J Gastroenterol Hepatol, 2022. **37**(10): p. 1963-1974.
159. Wal, A., P. Wal, N. Verma, A.K. Rai, and P. Vatsha, *Mechanism, Pathophysiology and Herbal Management-A Review on Irritable Bowel Syndrome.* Natural Products Journal, 2023. **13**(3): p. 16-30.
160. Chen, G.R., X.F. Xie, and C. Peng, *Treatment of Irritable Bowel Syndrome by Chinese Medicine: A Review.* Chinese Journal of Integrative Medicine, 2023. **29**(4): p. 377-384.
161. Zhao, J., X. Li, X. Chen, H. Zheng, K. Ye, Y. Shi, et al., *Common traditional Chinese medicine therapies for diarrhea-predominant irritable bowel syndrome: An overview of systematic reviews.* Journal of Traditional Chinese Medical Sciences, 2022. **9**(3): p. 330-339.
162. Zhang, G., T. Zhang, Z. Cao, Z. Tao, T. Wan, M. Yao, et al., *Effects and Mechanisms of Acupuncture on Diarrhea-Predominant Irritable Bowel Syndrome: A Systematic Review.* Front Neurosci, 2022. **16**: p. 918701.
163. Scaciota, A.C.L., D. Matos, M.M.B. Rosa, M.E.S. Colovati, E.F.B.C. Bellotto, and A.L.C. Martimbianco, *Interventions for the treatment of irritable bowel syndrome: A review of cochrane systematic reviews.* Arquivos de Gastroenterologia, 2021. **58**(1): p. 120-126.
164. Nee, J. and A. Lembo, *Review Article: Current and future treatment approaches for IBS with diarrhoea (IBS-D) and IBS mixed pattern (IBS-M).* Aliment Pharmacol Ther, 2021. **54 Suppl 1**: p. S63-s74.
165. Camilleri, M., *Diagnosis and Treatment of Irritable Bowel Syndrome: A Review.* JAMA - Journal of the American Medical Association, 2021. **325**(9): p. 865-877.
166. Bruta, K., Vanshika, K. Bhasin, and Bhawana, *The role of serotonin and diet in the prevalence of irritable bowel syndrome: a systematic review.* Translational Medicine Communications, 2021. **6**(1).
167. Amsallem, F., S. Sanchez, X. Armoiry, and F. Mion, *Effectiveness of Non-Pharmacological Interventions for Irritable Bowel Syndrome: A Systematic Review.* Evid Based Complement Alternat Med, 2021. **2021**: p. 4404185.
168. Gendi, R. and N. Jahan, *Pharmacological and Non-pharmacological Treatments of Irritable Bowel Syndrome and Their Impact on the Quality of Life: A Literature Review.* Cureus, 2020. **12**(7): p. e9324.
169. Pacheco, R.L., A. Roizenblatt, A.F.T. Góis, C.O.C. Latorraca, C.F.M.G.P. Mota, and R. Riera, *What do cochrane systematic reviews say about the management of irritable bowel syndrome?* Sao Paulo Medical Journal, 2019. **137**(1): p. 82-91.
170. Li, C.Y. and S.C. Li, *Treatment of irritable bowel syndrome in China: a review.* World J Gastroenterol, 2015. **21**(8): p. 2315-2322.
171. Chey, W.D., J. Kurlander, and S. Eswaran, *Irritable bowel syndrome: A clinical review.* JAMA - Journal of the American Medical Association, 2015. **313**(9): p. 949-958.
172. Bassett, J.T. and B.D. Cash, *A review of irritable bowel syndrome and an update on therapeutic approaches.* Expert Opinion on Pharmacotherapy, 2008. **9**(7): p. 1129-1143.
173. Podovei, M. and B. Kuo, *Irritable bowel syndrome: A practical review.* Southern Medical Journal, 2006. **99**(11): p. 1235-1243.
174. Olden, K.W., *Review of acupuncture treatment in irritable bowel syndrome: Commentary.* Evidence-Based Gastroenterology, 2006. **7**(4): p. 102-103.
175. Hussain, Z. and E.M.M. Quigley, *Systematic review: Complementary and alternative medicine in the irritable bowel syndrome.* Alimentary Pharmacology and Therapeutics, 2006. **23**(4): p. 465-471.
176. Southwell, B.R., *Electro-Neuromodulation for Colonic Disorders-Review of Meta-Analyses, Systematic Reviews, and RCTs.* Neuromodulation, 2020. **23**(8): p. 1061-1081.
177. Guo, Y., W. Wei, and J. Chen, *- Effects and mechanisms of acupuncture and electroacupuncture for functional dyspepsia: A systematic review.* 2020. **- 26**(- 19): p. - 2457.
178. Dobarrio-Sanz, I., J.M. Hernandez-Padilla, M.M. Lopez-Rodriguez, C. Fernandez-Sola, J. Granero-Molina, and M.D. Ruiz-Fernandez, *- Non-pharmacological interventions to improve constipation amongst older adults in long-term care settings: A systematic review of randomised controlled trials.* 2020. **- 41**(- 6): p. - 999.
179. Chi, Y., M. Wang, and J.P. Liu, *Eye Acupuncture as a Pain Relief Therapy: A Systematic Review of Randomized Controlled Trials.* Advances in Integrative Medicine, 2019. **6**: p. S130.
180. McPartland, J.M., G.W. Guy, and V. Di Marzo, *Care and feeding of the endocannabinoid system: A systematic review of potential clinical interventions that upregulate the endocannabinoid system.* PLoS ONE, 2014. **9**(3).
181. Lee, S., U.M. Jerng, Y. Liu, J.W. Kang, D. Nam, and J.D. Lee, *- The effectiveness and safety of moxibustion for treating cancer-related fatigue: a systematic review and meta-analyses.* 2014. **- 22**(- 5): p. - 1440.
182. Ernst, E., J. Snyder, and R.A. Dunlop, *National Center for Complementary and Alternative Medicine-funded randomised controlled trials of acupuncture: A systematic review.* Focus on Alternative and Complementary Therapies, 2012. **17**(1): p. 15-21.
183. Zhang, H., Z. Bian, and Z. Lin, *Are acupoints specific for diseases? A systematic review of the randomized controlled trials with sham acupuncture controls.* Chinese Medicine, 2010. **5**.
184. Schneider, A., K. Streitberger, and S. Joos, *Acupuncture treatment in gastrointestinal diseases: a systematic review.* World J Gastroenterol, 2007. **13**(25): p. 3417-3424.
185. Langmead, L. and D.S. Rampton, *Review article: Complementary and alternative therapies for inflammatory bowel disease.* Alimentary Pharmacology and Therapeutics, 2006. **23**(3): p. 341-349.
186. Herman, P.M., B.M. Craig, and O. Caspi, *Is complementary and alternative medicine (CAM) cost-effective? A systematic review.* BMC complementary and alternative medicine, 2005. **5**.
187. Linde, K. and S.N. Willich, *How objective are systematic reviews? Differences between reviews on complementary medicine.* Journal of the Royal Society of Medicine, 2003. **96**(1): p. 17‐22.
188. Linde, K., G. ter Riet, M. Hondras, A. Vickers, R. Saller, and D. Melchart, *Systematic reviews of complementary therapies - An annotated bibliography. Part 2: Herbal medicine.* BMC Complementary and Alternative Medicine, 2001. **1**.
189. Zheng, Q.H., J. Wang, H. Zheng, L.Y. Lu, S.Y. Zhou, X.Y. Hao, et al., *- What types of patients with chronic diarrhea benefit more from acupuncture treatment? A secondary analysis of a randomized controlled trial.* 2020. **- 35**.
190. Zheng, H., J. Xu, X. Sun, F. Zeng, Y. Li, X. Wu, et al., *- Electroacupuncture for patients with refractory functional dyspepsia: A randomized controlled trial.* 2018. **- 30**(- 7).
191. Zheng, H., Z.S. Liu, W. Zhang, M. Chen, F. Zhong, X.H. Jing, et al., *- Acupuncture for patients with chronic functional constipation: A randomized controlled trial.* 2018. **- 30**(- 7).
192. Zhang, R.X., L.X. Lao, K. Ren, and B.M. Berman, *- Mechanisms of Acupuncture-Electroacupuncture on Persistent Pain.* 2014. **- 120**(- 2): p. - 503.
193. Zhang, H.C., S.K. Han, and J.L. Tang, *Tonifying qi Invigorating blood Dispersing wind combined with scalp acupuncture for treating diarrhea-predominant irritable bowel syndrome in 50 Cases.* Journal of traditional chinese medicine [zhong yi za zhi], 2010. **51**(Sl): p. 220‐221.
194. Yik, Y.I., L. Stathopoulos, J.M. Hutson, and B.R. Southwell, *- Home Transcutaneous Electrical Stimulation Therapy to Treat Children With Anorectal Retention: A Pilot Study.* 2016. **- 19**(- 5): p. - 521.
195. Yang, N.N., J.W. Yang, C.X. Tan, Y.J. Li, Y. Wang, L.Y. Qi, et al., *- The Influence of Psychological Status on Acupuncture for Postprandial Distress Syndrome: A Subgroup Analysis of a Multicenter, Randomized Controlled Trial.* 2022. **- 2022**.
196. Yang, L., Y. Lu, H.F. Zhang, X.P. Ma, C.H. Bao, H.G. Wu, et al., *- Effect of warming moxibustion Tianshu (ST 25, bilateral) and Qihai (CV 6) for the treatment of diarrhea-dominant irritable bowel syndrome: a patient-blinded pilot trial with orthogonal design.* 2017. **- 37**(- 4): p. - 545.
197. Xiong, X.R. and Y. Lin, *Acupuncture and moxibustion and TCM simultaneously for treating Diarrhea-predominant irritable bowel syndrome in 42 Cases.* Journal of fujian university of TCM [fu jian zhong yi xue yuan xue bao], 2008. **18**(2): p. 38‐40.
198. Xiong, P. and W. Yang, *The high prevalence and burden of irritable bowel syndrome among university students.* General Hospital Psychiatry, 2023. **84**: p. 253-255.
199. Wu, X., C.H. Zheng, X.H. Xu, P. Ding, F. Xiong, M. Tian, et al., *- Electroacupuncture for Functional Constipation: A Multicenter, Randomized, Control Trial.* 2017. **- 2017**.
200. Whitfield, K.L. and R.J. Schulman, *Treatment options for functional gastrointestinal disorders: From empiric to complementary approaches.* Pediatric Annals, 2009. **38**(5): p. 288-294.
201. Vlieger, A.M. and M.A. Benninga, *Complementary therapies for pediatric functional gastrointestinal disorders.* Journal of Pediatric Gastroenterology and Nutrition, 2008. **47**(5): p. 707-709.
202. Villalon-Gomez, J.M., *How to meet the challenges of managing patients with IBS.* Journal of Family Practice, 2021. **70**(9): p. 431-441.
203. Vase, L., S. Baram, N. Takakura, H. Yajima, M. Takayama, T.J. Kaptchuk, et al., *- Specifying the nonspecific components of acupuncture analgesia.* 2013. **- 154**(- 9): p. - 1667.
204. Valenzuela, J., J. Alvarado, H. Cohen, A. Damiao, C. Francisconi, L. Frugone, et al., *Latin-American consensus document on irritable bowel syndrome.* Gastroenterologia y Hepatologia, 2004. **27**(5): p. 325-343.
205. Tort, S., A. Balboa, M. Marzo, R. Carrillo, M. Mínguez, J. Valdepérez, et al., *Clinical practice guideline for irritable bowel syndrome.* Gastroenterologia y Hepatologia, 2006. **29**(8): p. 467-521.
206. Tingting, M., *Ideas on trial design in acupuncture for IBS.* Am J Gastroenterol, 2010. **105**(3): p. 699.
207. Till, S.R., R. Nakamura, A. Schrepf, and S. As-Sanie, *Approach to Diagnosis and Management of Chronic Pelvic Pain in Women: Incorporating Chronic Overlapping Pain Conditions in Assessment and Management.* Obstetrics and Gynecology Clinics of North America, 2022. **49**(2): p. 219-239.
208. Tick, E., *Asklepian Dream Healing of Irritable Bowel Syndrome.* Explore: The Journal of Science and Healing, 2005. **1**(4): p. 290-291.
209. Teixeira, M.Z., C. Guedes, P.V. Barreto, and M.A. Martins, *- The placebo effect and homeopathy.* 2010. **- 99**(- 2): p. - 129.
210. Tao, B., S. Chao, C.H. Zheng, and G.Y. Huang, *- Acupuncture for the treatment of functional constipation.* 2016. **- 36**(- 5): p. - 587.
211. Tang, Z.P., *Traditional Chinese medicine clinical experience of the treatment for irritable bowel syndrome.* Chinese Journal of Integrative Medicine, 2009. **15**(2): p. 93-94.
212. Strobele, S. and E. Nel, *Functional abdominal pain - Not exclusively an adult condition.* South African Gastroenterology Review, 2013. **11**(1): p. 33-36.
213. Stones, R.W. and C. Price, *Health services for women with chronic pelvic pain.* Journal of the Royal Society of Medicine, 2002. **95**(11): p. 531-535.
214. Staud, R. and D.D. Price, *- Mechanisms of acupuncture analgesia for clinical and experimental pain.* 2006. **- 6**(- 5): p. - 667.
215. Staud, R., *- Effectiveness of CAM Therapy: Understanding the Evidence.* 2011. **- 37**(- 1): p. - +.
216. Sohn, L., D. Belvis, and S. Suresh, *Neuropathic pain or somatoform disorder: Is the verdict in the differential block?* Paediatric Anaesthesia, 2009. **19**(6): p. 630-631.
217. Shuai, P., X.H. Zhou, L.X. Lao, and X.S. Li, *- Issues of design and statistical analysis in controlled clinical acupuncture trials: an analysis of English-language reports from Western journals.* 2012. **- 31**(- 7): p. - 618.
218. Schneider, A., P. Enck, and K. Streitberger, *- Stratification by sex and subgroup is necessary for RCT on IBS - Reply.* 2006. **- 55**(- 5): p. - 744.
219. Schneider, A., P. Enck, and K. Streitberger, *Authors' reply [2].* Gut, 2006. **55**(5): p. 744.
220. Saps, M. and C. Di Lorenzo, *Probiotics for abdominal pain disorders in children - Safe to use but are they helpful?: Commentary.* Nature Clinical Practice Gastroenterology and Hepatology, 2007. **4**(8): p. 430-431.
221. Piche, T., M. Dapoigny, C. Bouteloup, P. Chassagne, B. Coffin, V. Desfourneaux, et al., *Recommendations for clinical practice in the management and treatment of chronic constipation of adults.* Gastroenterologie Clinique et Biologique, 2007. **31**(2): p. 125-135.
222. Pettit, J. and R. Glickman-Simon, *Osteopathic Manipulative Therapy for Preterm Infants, Acupuncture for Menopausal Symptoms, Mindfulness-Based Stress Reduction for Chronic Low Back Pain, Chocolate for Ischemic Heart Disease, Berberine for Irritable Bowel Syndrome.* Explore, 2016. **12**(5): p. 388-392.
223. Patel, M., I. Urits, A.D. Kaye, and O. Viswanath, *- The role of acupuncture in the treatment of chronic pain.* 2020. **- 34**(- 3): p. - 616.
224. Park, J., K. Linde, E. Manheimer, A. Molsberger, K. Sherman, C. Smith, et al., *- The Status and Future of Acupuncture Clinical Research.* 2008. **- 14**(- 7): p. - 881.
225. Noddin, L., M. Callahan, and B.E. Lacy, *Irritable bowel syndrome and functional dyspepsia: Different diseases or a single disorder with different manifestations?* MedGenMed Medscape General Medicine, 2005. **7**(3).
226. Nerurkar, A., *"The gut-brain axis: How to manage pain caused by this cross-talk": An overview of the symposium.* Global Advances In Health and Medicine, 2015. **4**(6): p. 61-64.
227. Mullin, G.E. and J.O. Clarke, *Role of Complementary and Alternative Medicine in Managing Gastrointestinal Motility Disorders.* Nutrition in Clinical Practice, 2010. **25**(1): p. 85-87.
228. Moynihan, N.T., M.J. Callahan, B. Kalsmith, and P.L. Moses, *How do you spell relief for irritable bowel syndrome?* Journal of Family Practice, 2008. **57**(2): p. 100-108.
229. Mora, R.M., *Doctor-patient relationship is the most established component of placebo effect.* FMC formacion medica continuada en atencion primaria, 2009. **16**(3): p. 178.
230. Meissner, K., *Journal Club.* Complementary Medicine Research, 2022. **29**(2): p. 93-94.
231. Meisler, J.G., *Toward optimal health: The experts discuss fibromyalgia.* Journal of Women's Health and Gender-Based Medicine, 2000. **9**(10): p. 1055-1060.
232. Mearin, F., C. Ciriza, M. Minguez, E. Rey, J.J. Mascort, E. Pena, et al., *- Irritable bowel syndrome with constipation and functional constipation in adults: Treatment (Part 2 of 2).* 2017. **- 49**(- 3): p. - 194.
233. Masuy, I., J. Pannemans, and J. Tack, *- Irritable bowel syndrome: diagnosis and management.* 2020. **- 66**(- 2): p. - 150.
234. MacPherson, H., D.G. Altman, R. Hammerschlag, Y.P. Li, T.X. Wu, A. White, et al., *- Revised STandards for Reporting Interventions in Clinical Trials of Acupuncture (STRICTA): Extending the CONSORT Statement.* 2010. **- 7**(- 6).
235. Ma, X.P., J. Hong, C.P. An, D. Zhang, Y. Huang, H.G. Wu, et al., *- Acupuncture-moxibustion in treating irritable bowel syndrome: How does it work?* 2014. **- 20**(- 20): p. - 6054.
236. Ma, T.T., F. Zeng, Y. Li, C.M. Wang, X.P. Tian, S.Y. Yu, et al., *- Which Subtype of Functional Dyspepsia Patients Responses Better to Acupuncture? A Retrospective Analysis of a Randomized Controlled Trial.* 2015. **- 22**(- 2): p. - 100.
237. Ma, T.H. and S.J. Zhang, *Shenling Baizhu San combined with acupoint RN 8 application for treating diarrhea-predominant irritable bowel syndrome in 72 Cases.* Hebei journal of traditional chinese medicine [he bei zhong yi], 2011. **31**(1): p. 48‐49.
238. Luigi, G.V.M.A.V. and F. Fabio, *Randomised controlled trial comparing acupuncture with placebo acupuncture for the treatment of irritable bowel syndrome.* 2012. **4**: p. 121.
239. Lowe, C., W. Depew, and S. Vanner, *A placebo-controlled, double-blind trial of acupuncture in the treatment of irritable bowel syndrome (IBS).* Gastroenterology, 2000. **118**(4 Suppl 2): p. A3168.
240. Liu, N. and J. Wang, *Clinical study of acupuncture at Shangjuxu (ST37) in treating irritable bowel syndrome.* International journal of clinical acupuncture, 2013. **22**(3): p. 135‐137.
241. Liu, J.J., C.A.Y. Chen, E. Giovannucci, and C.Y. Wu, *- Subsequent Primary Cancers of the Digestive System Among Childhood and Adolescent Cancer Survivors From 1975 to 2015 in the United States.* 2021. **- 116**(- 5): p. - 1071.
242. Linde, K., *Components of the placebo effect.* Forschende Komplementarmedizin, 2008. **15**(4): p. 230-232.
243. Liang, Y.S., Y. Zhang, and J.J. Feng, *Observation on effect of Taijiquan exercise combined with catgut implantation at acupuncture point in the treatment of constipation type irritable bowel syndrome.* Hubei journal of traditional chinese medicine [ hubei zhong yi za zhi], 2010. **32**(10): p. 50‐51.
244. Liang, Y.S., S. Xie, Y. Zhang, L.P. Wang, and C.L. Zhu, *Treatment of 40 cases of irritable bowel syndrome by acupoint thread burial therapy and taijiquan.* Shanghai journal of traditional chinese medicine [shang hai zhong yi yao za zhi], 2010. **44**(10): p. 38‐39; 43.
245. Li, Z., *The treatment of irritable bowel syndrome by acupuncture.* Journal of Chinese Medicine, 2004(74): p. 22-24.
246. Li, P.F., Y. Luo, Q. Wang, S. Shu, K.J. Chen, D.H. Yu, et al., *- Efficacy and Safety of Acupuncture at Tianshu (ST25) for Functional Constipation: Evidence from 10 Randomized Controlled Trials.* 2020. **- 2020**.
247. Lacy, B.E., *Proton pump inhibitor nonresponders.* Gastroenterology and Hepatology, 2015. **11**(7): p. 483-485.
248. Kundu, A. and B. Berman, *- Acupuncture for pediatric pain and symptom management.* 2007. **- 54**(- 6): p. - +.
249. Kotsis, V., S. Benson, U. Bingel, M. Forsting, M. Schedlowski, E.R. Gizewski, et al., *- Perceived treatment group affects behavioral and neural responses to visceral pain in a deceptive placebo study.* 2012. **- 24**(- 10).
250. Klinger, R., R. Kothe, J. Schmitz, S. Kamping, and H. Flor, *- Placebo effects of a sham opioid solution: a randomized controlled study in patients with chronic low back pain.* 2017. **- 158**(- 10): p. - 1902.
251. Kirsch, I., *Role of placebo in irritable bowel syndrome.* J Pediatr Gastroenterol Nutr, 2011. **53 Suppl 2**: p. S42-43.
252. Kim, T., J. Chung, S. Bae, J. Lee, J. Kim, J.A. Lee, et al., *- Efficacy and safety of different doses of moxibustion for irritable bowel syndrome: A randomised controlled pilot trial.* 2018. **- 20**: p. - 83.
253. Kaptchuk, T.J. and N.J. Talley, *Placebo acupuncture improved symptoms and quality of life in irritable bowel syndrome.* Evidence-Based Medicine, 2008. **13**(6): p. 180.
254. Kaiser, P., D.P. Kohen, M.L. Brown, R.L. Kajander, and A.J. Barnes, *- Integrating Pediatric Hypnosis with Complementary Modalities: Clinical Perspectives on Personalized Treatment.* 2018. **- 5**(- 8).
255. Jonas, W.B. and C. Crawford, *Enhancing the patient-practitioner relationship can improve outcomes for acupuncture interventions in irritable bowel syndrome: commentary.* Focus on alternative and complementary therapies, 2008. **13**(3): p. 192‐194.
256. Hummelsberger, J., *Comment.* Deutsche Zeitschrift fur Akupunktur, 2010. **53**(2): p. 41-42.
257. Hall, L., *The outer limits?* Occupational Health, 2005. **57**(SPEC. ISS.): p. 15-18.
258. Graham, L., *ACG releases recommendations on the management of irritable bowel syndrome.* American Family Physician, 2009. **79**(12): p. 1108-1112.
259. Gowan, J. and L. Roller, *Women's health and irritable bowel syndrome.* Australian Journal of Pharmacy, 2013. **94**(1114): p. 70-74.
260. Godlee, F., *Reclaiming the placebo effect.* BMJ, 2008. **336**(7651): p. 1P.
261. Glickman-Simon, R. and K. Withy, *Saw Palmetto, chinese red yeast extract, music therapy, Ginkgo biloba, increased practitioner interaction.* Explore: The Journal of Science and Healing, 2012. **8**(2): p. 138-140.
262. Glickman-Simon, R. and J. Wallace, *Acupuncture for knee osteoarthritis, chasteberry for premenstrual syndrome, probiotics for irritable bowel syndrome, yoga for hypertension, and trigger point dry needling for plantar fasciitis.* Explore: The Journal of Science and Healing, 2015. **11**(2): p. 157-161.
263. Glickman-Simon, R. and S. Savasta, *Acupuncture for acute stroke, peppermint oil for irritable bowel syndrome, yoga for depression and anxiety, hypnotherapy for smoking cessation, and lime juice for sickle cell anemia.* Explore (NY), 2014. **10**(4): p. 261-264.
264. Glickman-Simon, R. and A. Mukherji, *Moxibustion for Asthma, Acupuncture for Epilepsy, Psychological Therapies for Irritable Bowel Syndrome, Exercise Training for Multiple Sclerosis, and Comfrey Root for Acute Back Pain.* Explore: The Journal of Science and Healing, 2015. **11**(1): p. 67-71.
265. Glickman-Simon, R. and B.S. Alper, *Acupressure and postoperative vomiting, soy and breast cancer, gingko biloba and Alzheimer's disease, acupuncture and irritable bowel syndrome, mediterranean and low-carbohydrate diets.* Explore: The Journal of Science and Healing, 2013. **9**(2): p. 112-115.
266. Garland, B., *Patient's page.* Southern Medical Journal, 2006. **99**(11): p. 1317.
267. Ford, A.C. and N.J. Talley, *Irritable bowel syndrome.* BMJ (Online), 2012. **345**(7873).
268. Ford, A.C. and P. Moayyedi, *Dyspepsia.* BMJ (Online), 2013. **347**(7923).
269. Ford, A.C., *Acupuncture for irritable bowel syndrome.* Gastroenterology, 2012. **143**(6): p. 1683-1684.
270. Finset, A., *Clinician-patient interaction and health outcome: A potential impact on symptoms and quality of life in patients with pain?* Patient Education and Counseling, 2012. **89**(2): p. 217-218.
271. Feman, S.P.C., L.I. Nguyen, M.T. Quilty, C.E. Kerr, B.H. Nam, L.A. Conboy, et al., *- Effectiveness of recruitment in clinical trials: An analysis of methods used in a trial for irritable bowel syndrome patients.* 2008. **- 29**(- 2): p. - 251.
272. Evans, S., M. Moieni, B. Sternlieb, J.C.I. Tsao, and L.K. Zeltzer, *- Yoga for Youth in Pain The UCLA Pediatric Pain Program Model.* 2012. **- 26**(- 5): p. - 271.
273. Ernst, E., *Errors of alternative medicine: Lessons for general practice.* European Journal of General Practice, 2012. **18**(1): p. 63-66.
274. Eberl, S., N.M. de Olivera, D. Bourne, K. Streitberger, P. Fockens, M.W. Hollmann, et al., *- Effect of electroacupuncture on sedation requirements during colonoscopy: a prospective placebo-controlled randomised trial.* 2020. **- 38**(- 3): p. - 139.
275. Dong, J. and H. Zhang, *Clinical observation of calming-intestine scattered acupoints applying for treating diarrhea-predominant irritable bowel syndrome.* Journal of new chinese medicine [xin zhong yi], 2011. **43**(8): p. 106‐107.
276. Danis, P., A. Drew, S. Lingow, and S. Kurz, *Evidence-based tools for premenstrual disorders.* Journal of Family Practice, 2020. **69**(1): p. E9-E17.
277. Dalrymple, J. and I. Bullock, *Diagnosis and management of irritable bowel syndrome in adults in primary care: Summary of NICE guidance.* BMJ, 2008. **336**(7643): p. 556-558.
278. Curtiss, F.R., *Irritable bowel syndrome and antidepressants.* Journal of Managed Care Pharmacy, 2008. **14**(9): p. 882-885.
279. Cummings, M., *The pros and cons of sham acupuncture.* European journal of oriental medicine, 2010. **6**(4): p. 16‐19.
280. Cummings, M., *Is sham acupuncture effective for irritable bowel syndrome? Commentary.* Focus on alternative and complementary therapies, 2009. **14**(3): p. 192‐193.
281. Conboy, L.A., R.H. Wasserman, E.E. Jacobson, R.B. Davis, A.T.R. Legedza, M. Park, et al., *- Investigating placebo effects in irritable bowel syndrome: A novel research design.* 2006. **- 27**(- 2): p. - 134.
282. Chong, L.Y. and T. Treasure, *Acupuncture to relieve the pain of thoracotomy: Commentary on randomized, controlled trial.* Journal of Thoracic and Cardiovascular Surgery, 2008. **136**(6): p. 1470-1471.
283. Chen, X.Y., Y.H. Huang, and S.F. Yu, *Regulating Liver and Spleen Regimen combined with acupuncture for treating diarrhea-predominant Irritable Bowel Syndrome in 50 Cases.* Shanxi journal of traditional chinese medicine [shan xi zhong yi], 2005. **26**(1): p. 32‐33.
284. Chen, G.Y., M. Liang, S.G. Xu, and D.L. Chen, *Treating IBS with Chinese medicine combined with acupuncture point injection and psychotherapy in 82 cases.* Chinese journal of integrated traditional and western medicine on digestion [zhong guo zhong xi yi jie he xiao hua za zhi], 2006. **14**(2): p. 124‐125.
285. Chen, G.Y., M. Liang, D. Chen, and J.H. Hua, *Ping Ji San combined with acupoints injection for treating irritable bowel syndrome in 70 cases.* Chinese journal of traditional medical science and technology [zhong guo zhong yi yao ke ji], 2005. **12**(5): p. 322‐323.
286. Chakraborty, S. and A.E. Bharucha, *Commentary.* Annals of Internal Medicine, 2016. **165**(12): p. JC69.
287. Campbell, F. and B.J. Collett, *Chronic pelvic pain.* British Journal of Anaesthesia, 1994. **73**(5): p. 571-573.
288. Brandt, L.J., W.D. Chey, A.E. Foxx-Orenstein, E.M.M. Quigley, L.R. Schiller, P.S. Schoenfeld, et al., *An evidence-based position statement on the management of irritable bowel syndrome.* American Journal of Gastroenterology, 2008. **104**(SUPPL. 1): p. S1-S35.
289. Borah, B.J., J.M. Naessens, A.E. Glasgow, B.A. Bauer, and T.Y. Chon, *- Cost-effectiveness of acupuncture in an employee population: A retrospective analysis.* 2017. **- 31**: p. - 19.
290. Birch, S., M.S. Lee, T. Alraek, and T.H. Kim, *- Overview of Treatment Guidelines and Clinical Practical Guidelines That Recommend the Use of Acupuncture: A Bibliometric Analysis.* 2018. **- 24**(- 8): p. - 769.
291. Bian, Z.X., *- Stratification by sex and subgroup is necessary for RCT on IBS.* 2006. **- 55**(- 5): p. - 744.
292. Berle, C.A., D. Cobbin, N. Smith, and C. Zaslawski, *- A Novel Approach to Evaluate Traditional Chinese Medicine Treatment Outcomes Using Pattern Identification.* 2010. **- 16**(- 4): p. - 367.
293. Berle, C., D. Cobbin, N. Smith, and C. Zaslawski, *- An Innovative Method to Accommodate Chinese Medicine Pattern Diagnosis within the Framework of Evidence-Based Medical Research.* 2011. **- 17**(- 11): p. - 833.
294. Benninga, M.A. and E.A. Mayer, *The Power of Placebo in Pediatric Functional Gastrointestinal Disease.* Gastroenterology, 2009. **137**(4): p. 1207‐1210.
295. Bennett, R., *Fibromyalgia: Shining a light on fibromyalgia treatment.* Nature Reviews Rheumatology, 2016. **12**(10): p. 568-569.
296. Bauer, B.A., S.C. Litin, and J.B. Bundrick, *Clinical pearls in complementary and integrative medicine (CIM).* Disease-a-Month, 2014. **60**(7): p. 323-331.
297. Ballou, S., A. Beath, T.J. Kaptchuk, W. Hirsch, T. Sommers, J. Nee, et al., *- Factors Associated With Response to Placebo in Patients With Irritable Bowel Syndrome and Constipation.* 2018. **- 16**(- 11): p. - +.
298. Ao, X. and N. Wang, *Auricular-plaster therapy for treatment of IBS.* Journal of Traditional Chinese Medicine, 2004. **24**(3): p. 166-167.
299. An, G., N. Li, G. Zhai, H. Liu, J. Sun, and H. Liang, *Evaluation of the therapeutic effect of acupuncture and moxibustion on irritable bowel syndrome.* Shanghai journal of acupuncture and moxibustion, 2010. **29**(6): p. 354‐356.
300. Adriani, A., *Clinical key advances in non-pharmacological treatments for irritable bowel syndrome.* Gazzetta Medica Italiana Archivio per le Scienze Mediche, 2021. **180**(11): p. 629-631.
301. *Management of Bladder Pain Syndrome: Green-top Guideline No. 70.* BJOG: An International Journal of Obstetrics and Gynaecology, 2017. **124**(2): p. e46-e72.
302. *Poster - Gastroenterology.* Journal of Gastroenterology and Hepatology (Australia), 2015. **30**: p. 28-159.
303. *Oral.* Journal of Gastroenterology and Hepatology (Australia), 2015. **30**: p. 1-27.
304. *Clinical observation on combination of warm acupuncture and ear pressing for the treatment of 46 cases of irritable bowel syndrome.* Hunan guiding journal of traditional chinese medicine [hu nan zhong yi yao dao bao], 2004. **10**(3): p. 42.
305. Kaur, H. and A.S. Arunkalaivanan, *Urethral pain syndrome and its management.* Obstetrical and Gynecological Survey, 2007. **62**(5): p. 348-351.
306. Nakano, M., A. Maeda, M. Sakamoto, S. Yamazaki, T. Shibaji, and N. Suzuki, *Management of intractable toothache based on personality disorder: A case report.* Journal of Japanese Dental Society of Anesthesiology, 2005. **33**(3): p. 398-402.
307. Liu, W.H., C. Chen, F. Wang, S.N. Guo, Y. Hao, and S.D. Li, *Development trend and current situation of acupuncture-moxibustion indications.* World Journal of Acupuncture - Moxibustion, 2020. **30**(4): p. 245-250.
308. Siedentopf, F., P. Weijenborg, M. Engman, B. Maier, A. Cagnacci, S. Mimoun, et al., *ISPOG European Consensus Statement-chronic pelvic pain in women (short version).* Journal of Psychosomatic Obstetrics and Gynecology, 2015. **36**(4): p. 161-170.
309. Heusser, S.A. and S.A. Pless, *Acid-sensing ion channels as potential therapeutic targets.* Trends in Pharmacological Sciences, 2021. **42**(12): p. 1035-1050.
310. Lewith, G., *Acupuncture placebos.* European Journal of Oriental Medicine, 2010. **6**(4): p. 26-30.
311. Santucci, N.R., M. Saps, and M.A. van Tilburg, *New advances in the treatment of paediatric functional abdominal pain disorders.* Lancet Gastroenterol Hepatol, 2020. **5**(3): p. 316-328.
312. Thomas, L.V., K. Suzuki, and J. Zhao, *Probiotics: A proactive approach to health. A symposium report.* British Journal of Nutrition, 2015. **114**(S1): p. S1-S15.
313. Aaron, L.A. and D. Buchwald, *Chronic diffuse musculoskeletal pain, fibromyalgia and co-morbid unexplained clinical conditions.* Bailliere's Best Practice and Research in Clinical Rheumatology, 2003. **17**(4): p. 563-574.
314. Boregowda, G. and H.A. Shehata, *Gastrointestinal and liver disease in pregnancy.* Best Practice and Research: Clinical Obstetrics and Gynaecology, 2013. **27**(6): p. 835-853.
315. Linn, Y.C., *Evidence-based medicine for traditional Chinese medicine: Exploring the evidence from a western medicine perspective.* Proceedings of Singapore Healthcare, 2011. **20**(1): p. 12-19.
316. Buist, M., *Micromassage: The round-headed needle.* Journal of Chinese Medicine, 2017. **2017-June**(114): p. 69-79.
317. Shakeel, M., A. Trinidade, and K.W. Ah-See, *Complementary and alternative medicine use by otolaryngology patients: A paradigm for practitioners in all surgical specialties.* European Archives of Oto-Rhino-Laryngology, 2010. **267**(6): p. 961-971.
318. Nct, *Effect of Transcutaneous Auricular Vagal Nerve Stimulation on Chronic Constipation.* https://clinicaltrials.gov/show/NCT05723731, 2023.
319. Rudolph, C.D. and A. Miranda, *Treatment Options for Functional Abdominal Pain.* Pediatric Annals, 2004. **33**(2): p. 105-112.
320. Deutsch, J.K., J. Levitt, and D.J. Hass, *Complementary and Alternative Medicine for Functional Gastrointestinal Disorders.* American Journal of Gastroenterology, 2020. **115**(3): p. 350-364.
321. Nct, *A Study of Randomized Sham-control Auricular TENS Unit Stimulation in Pediatric Functional Gastrointestinal Disorders.* https://clinicaltrials.gov/show/NCT04247100, 2020.
322. *KIOM-SAR 2020 International Research Conference.* Integrative Medicine Research, 2020. **9**.
323. *5th European Congress for Integrative Medicine.* European Journal of Integrative Medicine, 2012. **4**.
324. Enck, P., B. Horing, K. Weimer, and S. Klosterhalfen, *Placebo responses and placebo effects in functional bowel disorders.* European Journal of Gastroenterology and Hepatology, 2012. **24**(1): p. 1-8.
325. Thukral, C. and J.L. Wolf, *Therapy insight: Drugs for gastrointestinal disorders in pregnant women.* Nature Clinical Practice Gastroenterology and Hepatology, 2006. **3**(5): p. 256-266.
326. Liu, H., Y. Zhang, D. Qi, and W. Li, *Downregulation of the spinal NMDA receptor NR2B subunit during electro-acupuncture relief of chronic visceral hyperalgesia.* J Physiol Sci, 2017. **67**(1): p. 197-206.
327. Greenwood, M.T., *Dysbiosis, Spleen Qi, Phlegm, and Complex Difficulties.* Medical Acupuncture, 2017. **29**(3): p. 128-137.
328. Ding, J.H., Z. Jin, X.X. Yang, J. Lou, W.X. Shan, Y.X. Hu, et al., *Role of gut microbiota via the gut-liver-brain axis in digestive diseases.* World Journal of Gastroenterology, 2020. **26**(40): p. 6141-6162.
329. Talotta, R., F. Atzeni, L. Bazzichi, C. Giacomelli, M. Di Franco, F. Salaffi, et al., *Algo-dysfunctional syndromes: A critical digest of the recent literature.* Clinical and Experimental Rheumatology, 2015. **33**: p. S102-S108.
330. Ernst, E., M.H. Pittler, B. Wider, and K. Boddy, *Acupuncture: Its evidence-base is changing.* American Journal of Chinese Medicine, 2007. **35**(1): p. 21-25.
331. Zaslawski, C., *Clinical reasoning in traditional Chinese medicine: Implications for clinical research.* Clinical Acupuncture and Oriental Medicine, 2003. **4**(2-3): p. 94-101.
332. Zhang, N., Y. Cheng, and L. Wang, *Mechanism of Traditional Chinese Medicine in Treating Depressive-related Syndromes Based on Intestinal Flora:A Review.* Chinese Journal of Experimental Traditional Medical Formulae, 2022. **28**(19): p. 267-273.
333. Daniela, G., P. Chiara, and C. Giuseppe, *The possible role of ketogenic diet in fibromyalgia treatment.* Pharmacologyonline, 2020(3 Special Issue): p. 122-126.
334. Nct, *Transcutaneous Electric Nerve Stimulation (TENS) for Vagal Modulation.* https://clinicaltrials.gov/ct2/show/NCT05987813, 2023.
335. Milgrom, L.R., *On the observed specific and non-specific effects of complex therapeutic interventions: Truly separate or complementary?* 2015. p. 79-92.
336. Leung, W.D. and S. Sherman, *Endoscopic Approach to the Patient with Motility Disorders of the Bile Duct and Sphincter of Oddi.* Gastrointestinal Endoscopy Clinics of North America, 2013. **23**(2): p. 405-434.
337. Stone, J.A.M., *The status of acupuncture and oriental medicine in the United States.* Chinese Journal of Integrative Medicine, 2014. **20**(4): p. 243-249.
338. Vercellini, P., P. Viganò, E. Somigliana, A. Abbiati, G. Barbara, and L. Fedele, *Medical, surgical and alternative treatments for chronic pelvic pain in women: A descriptive review.* Gynecological Endocrinology, 2009. **25**(4): p. 208-221.
339. Enck, P., U. Martens, and S. Klosterhalfen, *The psyche and the gut.* World Journal of Gastroenterology, 2007. **13**(25): p. 3405-3408.
340. Brusaferro, A., E. Farinelli, L. Zenzeri, R. Cozzali, and S. Esposito, *The Management of Paediatric Functional Abdominal Pain Disorders: Latest Evidence.* Pediatric Drugs, 2018. **20**(3): p. 235-247.
341. Bishop, F., E. Jacobsen, J. Shaw, and T. Kaptchuk, *Debriefing to placebo allocation: a phenomenological study of participants' experiences in a randomized clinical trial.* European journal of integrative medicine, 2010. **2**(4): p. 199.
342. Sack, K., *The pain that never heals: Diagnosing and managing patients with fibromyalgia.* Advanced Studies in Medicine, 2004. **4**(8): p. 401-408.
343. Pontari, M. and L. Giusto, *New developments in the diagnosis and treatment of chronic prostatitis/chronic pelvic pain syndrome.* Current Opinion in Urology, 2013. **23**(6): p. 565-569.
344. Shi, J.Y., J.A. Paredes Mogica, and E.J.B. De, *Non-Surgical Management of Chronic Pelvic Pain in Females.* Current Urology Reports, 2022. **23**(10): p. 245-254.
345. Ye, Y., C.C. Zhou, H.Q. Hu, I. Fukuzawa, and H.L. Zhang, *Underlying mechanisms of acupuncture therapy on polycystic ovary syndrome: Evidences from animal and clinical studies.* Frontiers in Endocrinology, 2022. **13**.
346. Bishop, F.L., E.E. Jacobson, J.R. Shaw, and T.J. Kaptchuk, *Scientific tools, fake treatments, or triggers for psychological healing: how clinical trial participants conceptualise placebos.* Soc Sci Med, 2012. **74**(5): p. 767-774.
347. Nelson, P., G. Apte, R. Justiz, J.M. Brismeé, G. Dedrick, and P.S. Sizer, *Chronic Female Pelvic Pain-Part 2: Differential Diagnosis and Management.* Pain Practice, 2012. **12**(2): p. 111-141.
348. Showalter, K., A. Hoffmann, N. Decredico, A. Thakrar, E. Arroyo, I. Goldberg, et al., *Complementary therapies for patients with systemic sclerosis.* Journal of Scleroderma and Related Disorders, 2019. **4**(3): p. 187-199.
349. Kaptchuk, T.J., J. Shaw, C.E. Kerr, L.A. Conboy, J.M. Kelley, T.J. Csordas, et al., *"Maybe I made up the whole thing": placebos and patients' experiences in a randomized controlled trial.* Cult Med Psychiatry, 2009. **33**(3): p. 382-411.
350. Bishop, F.L., E.E. Jacobson, J. Shaw, and T.J. Kaptchuk, *Participants' experiences of being debriefed to placebo allocation in a clinical trial.* Qual Health Res, 2012. **22**(8): p. 1138-1149.
351. Zhang, J., X. Ge, K. Zhang, Y. Qi, S. Ren, and X. Zhai, *Acupuncture for Parkinson's disease-related constipation: current evidence and perspectives.* Frontiers in Neurology, 2023. **14**.
352. Molfetta, L., G. Saviola, D. Fornasari, P.A. Grossi, A. Corcione, and B. Frediani, *Osteoarticular pain: therapeutic approach by paradigms.* European Review for Medical and Pharmacological Sciences, 2022. **26**(11): p. 4054-4068.
353. Ma, Z.B., Y.Y. Zheng, L.X. Ma, N.N. Guo, C. Li, Y.P. Wang, et al., *Clinical studies on the indications of 33 acupoints.* Medical Acupuncture, 2008. **20**(4): p. 269-275.
354. Grace, S., L. Barnes, W. Reilly, A. Vlass, and P. de Permentier, *An integrative review of dietetic and naturopathic approaches to functional bowel disorders.* Complement Ther Med, 2018. **41**: p. 67-80.
355. Chung, V.C.H., L.T.F. Ho, and I.X.Y. Wu, *Chinese medicine diagnosis and treatment for COVID-2019: Is China ready for implementing a national guideline?* Advances in Integrative Medicine, 2020. **7**(2): p. 51-54.
356. Chen, K. and C. Li, *Acupuncture combined with medication for ulcerative colitis with damp-heat syndrome at active phase.* Zhongguo zhen jiu [Chinese acupuncture & moxibustion], 2015. **35**(5): p. 435‐438.
357. He, W., Y. Tong, Y. Zhao, L. Zhang, H. Ben, Q. Qin, et al., *Review of controlled clinical trials on acupuncture versus sham acupuncture in Germany.* Journal of Traditional Chinese Medicine, 2013. **33**(3): p. 403-407.
358. Ciciora, S.L., V.O. Yildiz, W.Y. Jin, B. Zhao, and M. Saps, *Complementary and Alternative Medicine Use in Pediatric Functional Abdominal Pain Disorders at a Large Academic Center.* Journal of Pediatrics, 2020. **227**: p. 53-59.e51.
359. Sipaviciute, A., T. Aukstikalnis, N.E. Samalavicius, and A. Dulskas, *The Role of Traditional Acupuncture in Patients with Fecal Incontinence-Mini-Review.* Int J Environ Res Public Health, 2021. **18**(4).
360. Ma, Y., M. Dong, K. Zhou, C. Mita, J. Liu, and P.M. Wayne, *Publication trends in acupuncture research: a 20-year bibliometric analysis based on pubMed.* PloS one, 2016. **11**(12) (no pagination).
361. Wang, L., C.C. Yu, J. Li, Q. Tian, and Y.J. Du, *Mechanism of Action of Acupuncture in Obesity: A Perspective From the Hypothalamus.* Frontiers in Endocrinology, 2021. **12**.
362. Winter, J.W. and R.C. Heading, *The nonerosive reflux disease-gastroesophageal reflux disease controversy.* Current Opinion in Gastroenterology, 2008. **24**(4): p. 509-515.
363. Srinivasan, A.K., J.D. Kaye, and R. Moldwin, *Myofascial dysfunction associated with chronic pelvic floor pain: Management strategies.* Current Pain and Headache Reports, 2007. **11**(5): p. 359-364.
364. Nct, *Effects of an Integrative Treatment Model to Reduce Anxiety and Depression in Minor Mental Health Problems and Medically Unexplained Symptoms.* https://clinicaltrials.gov/show/NCT01631500, 2011.
365. Sarzi-Puttini, P., F. Atzeni, M. Di Franco, D. Buskila, A. Alciati, C. Giacomelli, et al., *Dysfunctional syndromes and fibromyalgia: A 2012 critical digest.* Clinical and Experimental Rheumatology, 2012. **30**(SUPPL.74): p. S143-S151.
366. Ernst, E., *Acupuncture: What Does the Most Reliable Evidence Tell Us?* Journal of Pain and Symptom Management, 2009. **37**(4): p. 709-714.
367. Shaver, J.L., *Fibromyalgia syndrome in women.* Nursing Clinics of North America, 2004. **39**(1): p. 195-204.
368. Cherniack, E.P., *Use of complementary and alternative medicine to treat constipation in the elderly.* Geriatrics and Gerontology International, 2013. **13**(3): p. 533-538.
369. Ma, S.S., H. Yu, Y.Q. Li, X.W. Zhang, N. Liang, Z. Li, et al., *Qualitative research on TCM complex interventions - the experience and cognition of Chinese patients.* European Journal of Integrative Medicine, 2023. **59**.
370. Van Haselen, R. and R. Jütte, *The placebo effect and its ramifications for clinical practice and research. Villa La Collina at Lake Como, Italy, 4-6 May 2012.* Complementary Therapies in Medicine, 2013. **21**(2): p. 85-93.
371. Ruiz, C., A. Yousefi, R. Hu, C. McLaughlin, E. Kokkoutou, and L. Conboy, *Using machine learning techniques to understand the complexity of pain experience with acupuncture treatment.* Journal of Alternative and Complementary Medicine, 2021. **27**(11): p. A6-A7.
372. Patel, N.K., E. Kusema, M.D. Crowell, and B.E. Lacy, *The placebo response in complementary and alternative medicine trials for functional dyspepsia.* American Journal of Gastroenterology, 2017. **112**: p. S249-S250.
373. Hung, A., N. Kang, A. Bollom, J.L. Wolf, and A. Lembo, *Complementary and Alternative Medicine Use Is Prevalent Among Patients with Gastrointestinal Diseases.* Digestive Diseases and Sciences, 2015. **60**(7): p. 1883-1888.
374. Farmer, A.D., G. Amersinghe, C. Brock, A. Drewes, A.M. Drewes, and Q. Aziz, *Electrical vagal nerve stimulation prevents the development of acid induced esophageal hyperalgesia.* Neurogastroenterology and motility, 2016. **28**: p. 48.
375. Chiarioni, G., M. Pesce, A. Fantin, and G. Sarnelli, *Complementary and alternative treatment in functional dyspepsia.* United European Gastroenterology Journal, 2018. **6**(1): p. 5-12.
376. Zheng, L., *New insights into the interplay between intestinal flora and bile acids in inflammatory bowel disease.* World Journal of Clinical Cases, 2022. **10**(30): p. 10823-10839.
377. Ntr, *Functional abdominal pain (FAP) within the context of internalizing disorders in childhood; A randomized controlled cognitive-behavioural family intervention.* https://trialsearch.who.int/Trial2.aspx?TrialID=NTR1613, 2009.
378. ChiCtr, *Effect and mechanism of percutaneous electrical nerve stimulation on functional constipation in children.* https://trialsearch.who.int/Trial2.aspx?TrialID=ChiCTR2200059549, 2022.
379. Irct2015043022027N, *Effect of acupressure on constipation of hemodialysis patients.* https://trialsearch.who.int/Trial2.aspx?TrialID=IRCT2015043022027N1, 2015.
380. Chi, C.T., *1H NMR-based metabolic profiling study on acupuncture treating for functional intestinal disorders.* https://trialsearch.who.int/Trial2.aspx?TrialID=ChiCTR-TRC-13003537, 2013.
381. Chi, C.T., *A fMRI study on acupuncture treating for functional intestinal disorders.* https://trialsearch.who.int/Trial2.aspx?TrialID=ChiCTR-TRC-13003241, 2013.
382. Kaspar, H., A. Abegg, and S. Reddy, *Of odysseys and miracles: A narrative approach on therapeutic mobilities for ayurveda treatment.* Social Science and Medicine, 2023. **334**.
383. Zhu, M., J. Gong, and Y. Liu, *On the natural medical features of traditional Chinese medicine.* Journal of Traditional Chinese Medicine, 2007. **27**(2): p. 158-160.
384. Nct, *Effect of Acupressure on Bowel Functions After Cesarean Section.* https://clinicaltrials.gov/show/NCT05251818, 2022.
385. Nct, *Acupuncture in the Treatment of Gulf War Illness.* https://clinicaltrials.gov/show/NCT01305811, 2011.
386. Rabitti, S., C.M. Giovanardi, and D. Colussi, *Acupuncture and Related Therapies for the Treatment of Gastrointestinal Diseases.* Journal of Clinical Gastroenterology, 2021. **55**(3): p. 207-217.
387. Dossett, M.L., E.M. Cohen, and J. Cohen, *Integrative Medicine for Gastrointestinal Disease.* Primary Care - Clinics in Office Practice, 2017. **44**(2): p. 265-280.
388. Henningsen, P., S. Zipfel, H. Sattel, and F. Creed, *Management of Functional Somatic Syndromes and Bodily Distress.* Psychotherapy and Psychosomatics, 2018. **87**(1): p. 12-31.
389. Petzke, F., *CNS processing of pain in functional somatic syndromes.* Schmerz, 2010. **24**(2): p. 146-155.
390. Mizuta, Y., S. Shikuwa, H. Isomoto, R. Mishima, Y. Akazawa, J.I. Masuda, et al., *Recent insights into digestive motility in functional dyspepsia.* Journal of Gastroenterology, 2006. **41**(11): p. 1025-1040.
391. Takahashi, T., *Acupuncture for functional gastrointestinal disorders.* Journal of Gastroenterology, 2006. **41**(5): p. 408-417.
392. Chen, J.D.Z., M. Ni, and J. Yin, *Electroacupuncture treatments for gut motility disorders.* Neurogastroenterology and Motility, 2018. **30**(7).
393. Yang, N.N., C.X. Tan, L.L. Lin, X.T. Su, Y.J. Li, L.Y. Qi, et al., *Potential Mechanisms of Acupuncture for Functional Dyspepsia Based on Pathophysiology.* Frontiers in Neuroscience, 2022. **15**.
394. Ghoshal, U.C., *Drug treatment and novel therapies in patients with functional dyspepsia.* Journal of Gastroenterology and Hepatology, 2012. **27**: p. 15.
395. Perez, M.E. and N.N. Youssef, *Dyspepsia in childhood and adolescence: Insights and treatment considerations.* Current Gastroenterology Reports, 2007. **9**(6): p. 447-455.
396. Grover, M. and D.A. Drossman, *Functional abdominal pain.* Current Gastroenterology Reports, 2010. **12**(5): p. 391-398.
397. Finniss, D.G., T.J. Kaptchuk, F. Miller, and F. Benedetti, *Biological, clinical, and ethical advances of placebo effects.* Lancet (london, england), 2010. **375**(9715): p. 686‐695.
398. Nct, *Auricular Vagus Nerve Stimulation For Fibromyalgia Syndrome.* https://clinicaltrials.gov/show/NCT04260906, 2020.
399. Mease, P., *Fibromyalgia syndrome: Review of clinical presentation, pathogenesis, outcome measures, and treatment.* Journal of Rheumatology, 2005. **32**(SUPPL. 75): p. 6-21.
400. Cymet, T.C., *A practical approach to fibromyalgia.* Journal of the National Medical Association, 2003. **95**(4): p. 278-285.
401. Clauw, D.J., *Fibromyalgia: An Overview.* American Journal of Medicine, 2009. **122**(12 SUPPL.): p. S3-S13.
402. Clauw, D.J., *Fibromyalgia and related conditions.* Mayo Clinic Proceedings, 2015. **90**(5): p. 680-692.
403. Buskila, D., *Pediatric Fibromyalgia.* Rheumatic Disease Clinics of North America, 2009. **35**(2): p. 253-261.
404. Calandre, E.P. and F. Rico-Villademoros, *The role of antipsychotics in the management of fibromyalgia.* CNS Drugs, 2012. **26**(2): p. 135-153.
405. Metyas, S., C. Chen, M. Joseph, N. Hanna, J. Basta, and A. Khalil, *Subcategories of Fibromyalgia-A New Concept.* Current Rheumatology Reviews, 2023. **19**(1): p. 18-25.
406. Gur, A. and P. Oktayoglu, *Advances in diagnostic and treatment options in patients with fibromyalgia syndrome.* Open Access Rheumatology: Research and Reviews, 2009. **1**(1): p. 193-209.
407. Puttini, P.S., W. Häuser, K. Lawson, and H. Sprott, *Topical seminar summary: Fibromyalgia syndrome.* European Journal of Pain, 2009. **13**: p. S5-S6.
408. Borten, P., *Raw herb powders and powdered herb extracts in the treatment of chronic idiopathic pain: A methodology and case study.* Journal of Chinese Medicine, 2009(89): p. 50-57.
409. Fung, F.Y. and Y.C. Linn, *Developing traditional Chinese medicine in the era of evidence-based medicine: Current evidences and challenges.* Evidence-based Complementary and Alternative Medicine, 2015. **2015**.
410. Kerr, C.E., J.R. Shaw, L.A. Conboy, J.M. Kelley, E. Jacobson, and T.J. Kaptchuk, *Placebo acupuncture as a form of ritual touch healing: a neurophenomenological model.* Conscious Cogn, 2011. **20**(3): p. 784-791.
411. Nct, *Trial for Quantity-Effect Relationship of Acupuncture With Two-ways Regulation to Treat Functional Enteropathy.* https://clinicaltrials.gov/show/NCT01274793, 2011.
412. Guo, X.X., J.F. Chen, Y. Lu, L.Y. Wu, Z.J. Weng, L. Yang, et al., *Electroacupuncture at He-Mu points reduces P2X4 receptor expression in visceral hypersensitivity.* Neural Regeneration Research, 2013. **8**(22): p. 2069-2077.
413. Chunder, R., *Non-ulcerative dyspepsia: Diagnosis and management.* SA Pharmaceutical Journal, 2011. **78**(2): p. 30-35.
414. Overland, M.K., *Dyspepsia.* Medical Clinics of North America, 2014. **98**(3): p. 549-564.
415. Michael Wolfe, M. and R.C. Lowe, *Investing in the future of GERD.* Journal of Clinical Gastroenterology, 2007. **41**(SUPPL.2): p. S209-S216.
416. Eckman, P., *The diagnosis and treatment of cancer with constitutional conditional acupuncture.* Journal of Chinese Medicine, 2021. **2021**(125): p. 3-16.
417. Włodarczyk, J., A. Waśniewska, J. Fichna, A. Dziki, Ł. Dziki, and M. Włodarczyk, *Current overview on clinical management of chronic constipation.* Journal of Clinical Medicine, 2021. **10**(8).
418. Van Wunnik, B.P.W., C.G.M.I. Baeten, and B.R. Southwell, *Neuromodulation for constipation: Sacral and transcutaneous stimulation.* Best Practice and Research: Clinical Gastroenterology, 2011. **25**(1): p. 181-191.
419. Youssef, N.N., *Childhood and adolescent constipation: Review and advances in management.* Current Treatment Options in Gastroenterology, 2007. **10**(5): p. 401-411.
420. Okumo, T., Y. Takayama, K. Maruyama, M. Kato, and M. Sunagawa, *Senso-Immunologic Prospects for Complex Regional Pain Syndrome Treatment.* Frontiers in Immunology, 2021. **12**.
421. Korzenik, J., A.K. Koch, and J. Langhorst, *Complementary and Integrative Gastroenterology.* Medical Clinics of North America, 2017. **101**(5): p. 943-954.
422. Leong, M., T.J. Smith, and A. Rowland-Seymour, *Complementary and integrative medicine for older adults in palliative care.* Clinics in Geriatric Medicine, 2015. **31**(2): p. 177-191.
423. Little, J.W., *Complementary and alternative medicine: Impact on dentistry.* Oral Surgery, Oral Medicine, Oral Pathology, Oral Radiology, and Endodontics, 2004. **98**(2): p. 137-145.
424. Michelfelder, A.J., K.C. Lee, and E.M. Bading, *Integrative medicine and gastrointestinal disease.* Primary Care - Clinics in Office Practice, 2010. **37**(2): p. 255-267.
425. Dinning, P.G. and S.M. Scott, *Novel diagnostics and therapy of colonic motor disorders.* Curr Opin Pharmacol, 2011. **11**(6): p. 624-629.
426. Homma, Y., T. Ueda, H. Tomoe, A.T.L. Lin, H.C. Kuo, M.H. Lee, et al., *Clinical guidelines for interstitial cystitis and hypersensitive bladder updated in 2015.* International Journal of Urology, 2016. **23**(7): p. 542-549.
427. Xiang, H., T. Zhang, A. Al-Danakh, D. Yang, and L. Wang, *Neuromodulation in Chronic Pelvic Pain: A Narrative Review.* Pain and Therapy, 2022. **11**(3): p. 789-816.
428. *Chronic pelvic pain.* Obstetrics and Gynecology, 2004. **103**(3): p. 589-604.
429. Gurian, M.B.F., A.M. De Souza, A.P.M. Da Silva, M.L.L. De Souza Montenegro, O.B. Poli Neto, F.J.C. Dos Reis, et al., *Chronic pelvic pain of musculoskeletal cause in women.* Expert Review of Obstetrics and Gynecology, 2012. **7**(2): p. 149-157.
430. Nct, *Acupuncture for Female With Non-cyclic Chronic Pelvic Pain.* https://clinicaltrials.gov/show/NCT04553562, 2020.
431. Ghosh, M. and K. Ojha, *Medical and surgical management of chronic pelvic pain.* Obstetrics, Gynaecology and Reproductive Medicine, 2011. **21**(9): p. 249-253.
432. Ball, E. and K.S. Khan, *Recent advances in understanding and managing chronic pelvic pain in women with special consideration to endometriosis.* F1000Research, 2020. **9**.
433. Kuner, R., *Central mechanisms of pathological pain.* Nature Medicine, 2010. **16**(11): p. 1258-1266.
434. Yazdani, S. and L. Zeltzer, *Treatment of chronic pain in children and adolescents.* Pain Management, 2013. **3**(4): p. 303-314.
435. Ziessman, H.A., *Functional hepatobiliary disease: Chronic acalculous gallbladder and chronic acalculous biliary disease.* Seminars in Nuclear Medicine, 2006. **36**(2): p. 119-132.
436. MacPherson, H., *Pragmatic clinical trials.* Complementary Therapies in Medicine, 2004. **12**(2-3): p. 136-140.
437. Shi, X., Y. Hu, B. Zhang, W. Li, J.D. Chen, and F. Liu, *Ameliorating effects and mechanisms of transcutaneous auricular vagal nerve stimulation on abdominal pain and constipation.* JCI insight, 2021. **6**(14).
438. Varghese, C., W. Xu, C. Daker, I.P. Bissett, and C. Cederwall, *Clinical utility of Gastric Alimetry® in the management of intestinal failure patients with possible underlying gut motility disorders.* Clinical Nutrition Open Science, 2023. **51**: p. 15-25.
439. Mahabir, V.K., C.S. Smith, C. Vannabouathong, J.J. Merchant, and A.L. Garibaldi, *Comparing medical cannabis use in 5 US states: a retrospective database study.* Journal of Cannabis Research, 2021. **3**(1).
440. Lahner, E., S. Bellentani, R. De Bastiani, C. Tosetti, M. Cicala, G. Esposito, et al., *A survey of pharmacological and nonpharmacological treatment of functional gastrointestinal disorders.* United European Gastroenterology Journal, 2013. **1**(5): p. 385-393.
441. Hopkins, R.J., C. Howard, E. Hunter-Stitt, P.E. Kaptur, B. Pleune, D. Muse, et al., *Phase 3 trial evaluating the immunogenicity and safety of a three-dose BioThrax® regimen for post-exposure prophylaxis in healthy adults.* Vaccine, 2014. **32**(19): p. 2217‐2224.
442. Frøkjaer, J.B., S. Bergmann, C. Brock, A. Madzak, A.D. Farmer, J. Ellrich, et al., *Modulation of vagal tone enhances gastroduodenal motility and reduces somatic pain sensitivity.* Neurogastroenterology and motility, 2016. **28**(4): p. 592‐598.
443. Cheng, S., H. Li, J. Luo, J. Chi, W. Zhao, J. Lin, et al., *Egg yolk antibody combined with bismuth-based quadruple therapy in Helicobacter pylori infection rescue treatment: a single-center, randomized, controlled study.* Frontiers in Microbiology, 2023. **14**.
444. Manheimer, E., S. Wieland, E. Kimbrough, K. Cheng, and B.M. Berman, *Evidence from the cochrane collaboration for traditional chinese medicine therapies.* Journal of Alternative and Complementary Medicine, 2009. **15**(9): p. 1001-1014.
445. Ko, S.J., J. Kim, J. Lee, K.J. Kim, H. Jun, T.J. Kaptchuk, et al., *Influence of the patient-practitioner interaction context on acupuncture outcomes in functional dyspepsia.* Integrative Medicine Research, 2020. **9**.
446. Chen, B., Y. Ma, C. Zhong, Y. Li, J. Mo, S. Liang, et al., *Suppression effect and safety of acupuncture on colonic spasm during colonoscopy: a randomized controlled trial.* Journal of gastrointestinal oncology, 2022. **13**(3): p. 1169‐1177.
447. Wauters, L., R. Dickman, V. Drug, A. Mulak, J. Serra, P. Enck, et al., *United European Gastroenterology (UEG) and European Society for Neurogastroenterology and Motility (ESNM) consensus on functional dyspepsia.* Neurogastroenterology and Motility, 2021. **33**(9).
448. Van Tilburg, M.A.L., O.S. Palsson, R.L. Levy, A.D. Feld, M.J. Turner, D.A. Drossman, et al., *Complementary and alternative medicine use and cost in functional bowel disorders: A six month prospective study in a large HMO.* BMC Complementary and Alternative Medicine, 2008. **8**.
449. Singh, B.B., R. Khorsan, and S.P. Vinjamury, *Influence of comorbidities on improvement of fibromyalgia symptoms when treated with acupuncture: a short report.* Altern Ther Health Med, 2008. **14**(5): p. 24-25.
450. Huang, M.C., H.R. Yen, C.L. Lin, Y.C. Lee, M.F. Sun, and M.Y. Wu, *Acupuncture decreased the risk of stroke among patients with fibromyalgia in Taiwan: A nationwide matched cohort study.* PLoS ONE, 2020. **15**(10 October).
451. Henningsen, P., S. Zipfel, and W. Herzog, *Management of functional somatic syndromes.* Lancet, 2007. **369**(9565): p. 946-955.
452. Xu, S. and L. Lao, *From basic science studies to clinical trials: What recent acupuncture research tells us.* Medical Acupuncture, 2012. **24**(1): p. 10-14.
453. Jindal, V., A. Ge, and P.J. Mansky, *Safety and efficacy of acupuncture in children: A review of the evidence.* Journal of Pediatric Hematology/Oncology, 2008. **30**(6): p. 431-442.
454. Zhou, J., B. Zhou, X. Kou, T. Jian, L. Chen, X. Lei, et al., *Effect of summer acupoint application treatment (SAAT) on gut microbiota in healthy Asian adults: A randomized controlled trial.* Medicine, 2023. **102**(9): p. e32951.
455. Zeiter, D.K., *Abdominal Pain in Children: From the Eternal City to the Examination Room.* Pediatric Clinics of North America, 2017. **64**(3): p. 525-541.
456. Gray, M.G., B.R. Lackey, E.F. Patrick, S.L. Gray, and S.G. Hurley, *Multiple Integrated Complementary Healing Approaches: Energetics & Light for bone.* Medical Hypotheses, 2016. **86**: p. 18-29.
457. Wiebelitz, K.R. and A.M. Beer, *Phytotherapy of chronic abdominal pain following pancreatic carcinoma surgery: A single case observation.* International Journal of General Medicine, 2012. **5**: p. 845-848.
458. Koretz, R.L. and M. Rotblatt, *Complementary and alternative medicine in gastroenterology: the good, the bad, and the ugly.* Clin Gastroenterol Hepatol, 2004. **2**(11): p. 957-967.
459. Cheung, D. and N. Trudgill, *Managing a patient with burning mouth syndrome.* Frontline Gastroenterology, 2015. **6**(3): p. 218-222.
460. Evans, S., *Managing chronic pelvic pain in girls and women.* Medicine Today, 2013. **14**(5): p. 54-58.
461. Chung, Y. and R.C. Dumont, *Complementary and alternative therapies: Use in pediatric pulmonary medicine.* Pediatric Pulmonology, 2011. **46**(6): p. 530-544.
462. Gros, M., P.O. Ndong, C. Jemaï, J.L. Toscano, B.M. Florance, and T. Piche, *The role of complementary alternative medicines in the irritable bowel syndrome.* Hepato-Gastro et Oncologie Digestive, 2022. **29**(3): p. 368-378.
463. Luty, J., *Medically unexplained syndromes: Irritable bowel syndrome, fibromyalgia and chronic fatigue.* BJPsych Advances, 2018. **24**(4): p. 252-263.
464. Camilleri, M., *Editorial: is adequate relief fatally flawed or adequate as an end point in irritable bowel syndrome?* Am J Gastroenterol, 2009. **104**(4): p. 920-922.
465. Nct, *Efficacy of Acupuncture in Refractory Irritable Bowel Syndrome.* https://clinicaltrials.gov/show/NCT04276961, 2020.
466. Xue, H. and S. Shao, *Effect of acupuncture combined with Astragalus injection on peripheral blood inflammatory factors in children with diarrhea-predominant irritable bowel syndrome.* European journal of inflammation, 2019. **17**.
467. Mulak, A. and L. Paradowski, *Therapy for irritable bowel syndrome - Present possibilities and new perspectives.* Gastroenterologia Polska, 2004. **11**(6): p. 521-527.
468. Wu, H.G., B. Jiang, E.H. Zhou, Z. Shi, D.R. Shi, Y.H. Cui, et al., *Regulatory mechanism of electroacupuncture in irritable bowel syndrome: preventing MC activation and decreasing SP VIP secretion.* Dig Dis Sci, 2008. **53**(6): p. 1644-1651.
469. Quilty, M. and L. Conboy, *Relationship of treatment beliefs to subject blinding: the case of a placebo-controlled RCT in Irritable Bowel Syndrome.* BMC complementary and alternative medicine, 2012. **12**.
470. ChiCtr, *Clinical efficacy evaluation of taVNS for patients with IBS-D.* https://trialsearch.who.int/Trial2.aspx?TrialID=ChiCTR2200062593, 2022.
471. Gu, Y., Y.T. Lai, F.R. Chang, and C.Y. Chen, *Utilization patterns and prescription characteristics of traditional Chinese medicine among patients with irritable bowel syndrome in Taiwan.* Frontiers in Pharmacology, 2023. **14**.
472. Ammoury, R.F., M.D.R. Pfefferkorn, and J.M. Croffie, *Functional gastrointestinal disorders: Past and present.* World Journal of Pediatrics, 2009. **5**(2): p. 103-112.
473. Diehl, D.L., *Acupuncture for gastrointestinal and hepatobiliary disorders.* Journal of Alternative and Complementary Medicine, 1999. **5**(1): p. 27-45.
474. Drossman, D.A., C.B. Morris, S. Schneck, Y.J.B. Hu, N.J. Norton, W.F. Norton, et al., *International survey of patients with IBS: Symptom features and their severity, health status, treatments, and risk taking to achieve clinical benefit.* Journal of Clinical Gastroenterology, 2009. **43**(6): p. 541-550.
475. Fábián, A., M. Rutka, T. Ferenci, R. Bor, A. Bálint, K. Farkas, et al., *The use of complementary and alternative medicine is less frequent in patients with inflammatory bowel disease than in patients with other chronic gastrointestinal disorders.* Gastroenterology Research and Practice, 2018. **2018**.
476. Halland, M. and N.J. Talley, *New treatments for IBS.* Nature Reviews Gastroenterology and Hepatology, 2013. **10**(1): p. 13-23.
477. Khan, S. and L. Chang, *Diagnosis and management of IBS.* Nature Reviews Gastroenterology and Hepatology, 2010. **7**(10): p. 565-581.
478. Kovacic, K., K. Hainsworth, M. Sood, G. Chelimsky, R. Unteutsch, M. Nugent, et al., *Neurostimulation for abdominal pain-related functional gastrointestinal disorders in adolescents: a randomised, double-blind, sham-controlled trial.* The lancet. Gastroenterology & hepatology, 2017. **2**(10): p. 727‐737.
479. Lam, C., *Nutrition and medical acupuncture: Three case presentations.* Journal of Orthomolecular Medicine, 2006. **21**(4): p. 200-204.
480. Langmead, L., M. Chitnis, and D.S. Rampton, *Use of complementary therapies by patients with IBD may indicate psychosocial distress.* Inflammatory Bowel Diseases, 2002. **8**(3): p. 174-179.
481. Li, P.F., Y. Luo, Q. Wang, S. Shu, K.J. Chen, D.H. Yu, et al., *- Efficacy and Safety of Acupuncture at Tianshu (ST25) for Functional Constipation: Evidence from 10 Randomized Controlled Trials.* 2020. **- 2020**.
482. Mou, J.J., Q. Wang, H.Y. Luo, and Q.K. Feng, *The observation of clinical efficacy of treating IBS-D with needle warming therapy.* World chinese medicine [shi jie zhong yi yao], 2016. **11**(11): p. 2404‐2407.
483. Mullin, G.E., O. Pickett-Blakely, and J.O. Clarke, *Integrative medicine in gastrointestinal disease: Evaluating the evidence.* Expert Review of Gastroenterology and Hepatology, 2008. **2**(2): p. 261-280.
484. Paul, S.P. and D. Basude, *Non-pharmacological management of abdominal pain-related functional gastrointestinal disorders in children.* World Journal of Pediatrics, 2016. **12**(4): p. 389-398.
485. Srinath, A.I., C. Walter, M.C. Newara, and E.M. Szigethy, *Pain management in patients with inflammatory bowel disease: Insights for the clinician.* Therapeutic Advances in Gastroenterology, 2012. **5**(5): p. 339-357.
486. Takahashi, T., *Effect and mechanism of acupuncture on gastrointestinal diseases*. 2013. p. 273-294.
487. Tillisch, K., *Complementary and alternative medicine for gastrointestinal disorders.* Clinical Medicine, Journal of the Royal College of Physicians of London, 2007. **7**(3): p. 224-227.
488. Wadhera, V., D.A. Lemberg, S.T. Leach, and A.S. Day, *Complementary and alternative medicine in children attending gastroenterology clinics: Usage patterns and reasons for use.* Journal of Paediatrics and Child Health, 2011. **47**(12): p. 904-910.
489. Accarino, A.M., F. Azpiroz, and J.R. Malagelada, *Selective dysfunction of mechanosensitive intestinal afferents in irritable bowel syndrome.* Gastroenterology, 1995. **108**(3): p. 636-643.
490. Actrn, *A pilot study of traditional acupuncture as an additional treatment for irritable bowel syndrome-diarrhoea.* https://trialsearch.who.int/Trial2.aspx?TrialID=ACTRN12622000073707, 2022.
491. Anastasi, J.K., B. Capili, J. Quinn, D.J. McMahon, and C. Scully, *Irritable bowel syndrome subtype screening characteristics: constipation subtype patient profiles explored.* Gastroenterology insights, 2012. **4**(2): p. 69‐72.
492. Asare, F., S. Sẗrsrud, and M. Simŕn, *Meditation over medication for irritable bowel syndrome? on exercise and alternative treatments for irritable bowel syndrome.* Current Gastroenterology Reports, 2012. **14**(4): p. 283-289.
493. Banerjee, S., *Irritable bowel syndrome.* Journal of Pain and Palliative Care Pharmacotherapy, 2010. **24**(3): p. 271-274.
494. Bonetto, S., V. Boano, E. Valenzi, G.M. Saracco, and R. Pellicano, *Non-pharmacological strategies to treat irritable bowel syndrome: 2022 update.* Minerva Gastroenterol (Torino), 2022. **68**(4): p. 475-481.
495. Brabyn, S., J. Adamson, H. MacPherson, H. Tilbrook, and D.J. Torgerson, *Short message service text messaging was feasible as a tool for data collection in a trial of treatment for irritable bowel syndrome.* J Clin Epidemiol, 2014. **67**(9): p. 993-1000.
496. Cannova, T.J., C. Avena-Woods, and T.Z. Jodlowski, *Irritable bowel syndrome treatment options*. 2012. p. 45-48.
497. Chan, J., I. Carr, and J.F. Mayberry, *The role of acupuncture in the treatment of irritable bowel syndrome: a pilot study.* Hepatogastroenterology, 1997. **44**(17): p. 1328-1330.
498. Chen, G., J.X. Luo, and X.Y. Hu, *Tiaoshen acupuncture for diarrhea type IBS in patients with chronic hepatitis B (Liver stagnation and spleen deficiency syndrome): curative effect and influence on plasma SP and VIP.* World chinese journal of digestology, 2015. **23**(8): p. 1303‐1307.
499. ChiCtr, *Neuroimaging study on the systemic regulatory effect mechanism of electroacupuncture at different frequencies in the treatment of irritable bowel syndrome.* https://trialsearch.who.int/Trial2.aspx?TrialID=ChiCTR2300073808, 2023.
500. Chu, H.R., N. Li, and H.L. Cheng, *Observations on the therapeutic effect of warm needling moxibustion on irritable bowel syndrome of Liver Depression and Spleen Deficiency Type.* Shanghai journal of acupuncture and moxibustion [shang hai zhen jiu za zhi], 2015. **34**(5): p. 424‐425.
501. Chu, W.C.W., J.C.Y. Wu, D.T.W. Yew, L. Zhang, L. Shi, D.K.W. Yeung, et al., *Does acupuncture therapy alter activation of neural pathway for pain perception in irritable bowel syndrome?: A comparative study of true and sham acupuncture using functional magnetic resonance imaging.* Journal of Neurogastroenterology and Motility, 2012. **18**(3): p. 305-316.
502. Coban, Ş., E. Akbal, S. Köklü, G. Köklü, M.A. Ulaşlı, S. Erkeç, et al., *Clinical trial: transcutaneous interferential electrical stimulation in individuals with irritable bowel syndrome - a prospective double-blind randomized study.* Digestion, 2012. **86**(2): p. 86-93.
503. Dai, Y.K., Y.B. Wu, R.L. Li, W.J. Chen, C.Z. Tang, L.M. Lu, et al., *Efficacy and safety of non-pharmacological interventions for irritable bowel syndrome in adults.* World J Gastroenterol, 2020. **26**(41): p. 6488-6509.
504. Dong, Y., D. Baumeister, S. Berens, W. Eich, and J. Tesarz, *High Rates of Non-Response Across Treatment Attempts in Chronic Irritable Bowel Syndrome: Results From a Follow-Up Study in Tertiary Care.* Frontiers in Psychiatry, 2019. **10**.
505. Enck, P. and N. Mazurak, *Nonmedicinal therapies of irritable bowel syndrome.* Gastroenterologe, 2017. **12**(2): p. 141-149.
506. Fassov, J.L., L. Lundby, S. Laurberg, S. Buntzen, and K. Krogh, *A randomized, controlled, crossover study of sacral nerve stimulation for irritable bowel syndrome.* Annals of surgery, 2014. **260**(1): p. 31‐36.
507. Feng, F., Q. Li, L.J. Liu, X.Y. Li, R. Jin, and B.H. Yan, *The study of Sanfu-moxibustion with acupuncture and moxibustion in treating irritable bowel syndrome.* Liaoning journal of traditional chinese medicine [liaoning zhong yi za zhi], 2015. **42**(6): p. 1322‐1324.
508. Forbes, A., *Irritable bowel syndrome: Alternative therapeutic management.* Annals of Gastroenterology, 2002. **15**(3): p. 290-293.
509. Gabuzian, K.S., K.A. Sarkisian, N.L. Grigorian, and Z.G. Azatian, *Long-term results in the treatment of patients with irritable bowel syndrome.* Klinicheskaia meditsina, 1994. **72**(1): p. 47‐48.
510. Go, G.Y. and H. Park, *Effects of Auricular Acupressure on Women With Irritable Bowel Syndrome.* Gastroenterol Nurs, 2020. **43**(2): p. E24-e34.
511. Grover, M., *When is irritable bowel syndrome not irritable bowel syndrome? diagnosis and treatment of chronic functional abdominal pain.* Current Gastroenterology Reports, 2012. **14**(4): p. 290-296.
512. Guo, J., G. Lu, L. Chen, H. Geng, X. Wu, H. Chen, et al., *Regulation of serum microRNA expression by acupuncture in patients with diarrhea-predominant irritable bowel syndrome.* Acupunct Med, 2022. **40**(1): p. 34-42.
513. Guo, J., J.H. Sun, L. Chen, H. Geng, X.L. Wu, Y.F. Song, et al., *Correlation between curative effect and 5-HTTLPR polymorphism in treatment of diarrhea-predominant irritable bowel syndrome with acupuncture for regulating shen and strengthening spleen.* Zhongguo zhen jiu [Chinese acupuncture & moxibustion], 2021. **41**(4): p. 365‐370.
514. Hagège, H., *Alternative treatments in irritable bowel syndrome.* Gastroenterologie Clinique et Biologique, 2009. **33**(SUPPL. 1): p. S79-S83.
515. Hall, K.T., A.J. Lembo, I. Kirsch, D.C. Ziogas, J. Douaiher, K.B. Jensen, et al., *Catechol-O-Methyltransferase val158met Polymorphism Predicts Placebo Effect in Irritable Bowel Syndrome.* PLoS ONE, 2012. **7**(10).
516. Harris, L.R. and L. Roberts, *Treatments for irritable bowel syndrome: patients' attitudes and acceptability.* BMC Complement Altern Med, 2008. **8**: p. 65.
517. Hasler, W.L., *Irritable bowel syndrome and bloating.* Best Practice and Research in Clinical Gastroenterology, 2007. **21**(4): p. 689-707.
518. Hou, Y., Y. Zhao, H. Jiang, K. Wang, W. Zhang, S. Zhou, et al., *Maintenance of Intestinal Homeostasis in Diarrhea-Predominant Irritable Bowel Syndrome by Electroacupuncture Through Submucosal Enteric Glial Cell-Derived S-Nitrosoglutathione.* Front Physiol, 2022. **13**: p. 917579.
519. Huang, Z., Z. Lin, and N. Dai, *NEEDLELESS TRANSCUTANEOUS ELECTRICAL ACUSTIMULATION IMPROVES IRRITABLE BOWEL SYNDROME WITH CONSTIPATION VIA AUTONOMIC MECHANISMS.* Gastroenterology, 2019. **156**(6): p. S‐465.
520. Huang, Z., Z. Lin, C. Lin, H. Chu, X. Zheng, B. Chen, et al., *Transcutaneous Electrical Acustimulation Improves Irritable Bowel Syndrome With Constipation by Accelerating Colon Transit and Reducing Rectal Sensation Using Autonomic Mechanisms.* American journal of gastroenterology, 2022. **117**(9): p. 1491‐1501.
521. Hummelsberger, J., *Components of placebo effect: Randomized controlled trial in patients with irritable bowel syndrome.* Revista Internacional de Acupuntura, 2010. **4**(3): p. 149-150.
522. Irct20191215045743N, *Evaluation of the effectiveness of neurofeedback on irritable bowel syndrome.* https://trialsearch.who.int/Trial2.aspx?TrialID=IRCT20191215045743N1, 2020.
523. Isrctn, *Acupuncture for irritable bowel syndrome: a pilot for a randomised controlled trial.* https://trialsearch.who.int/Trial2.aspx?TrialID=ISRCTN32823720, 2006.
524. Itmctr, *The brain effect and mechanism of ''Shuji acupoint'' acupuncture on the brain functional connectivity network of PATIENTS with IBS based on the rs-fMRI technique.* https://trialsearch.who.int/Trial2.aspx?TrialID=ITMCTR2200005558, 2022.
525. Kct, *Symptom management for irritable bowel syndrome.* https://trialsearch.who.int/Trial2.aspx?TrialID=KCT0002064, 2016.
526. Kelley, J.M., A.J. Lembo, J.S. Ablon, J.J. Villanueva, L.A. Conboy, R. Levy, et al., *Patient and practitioner influences on the placebo effect in irritable bowel syndrome.* Psychosom Med, 2009. **71**(7): p. 789-797.
527. Kokkotou, E., L.A. Conboy, D.C. Ziogas, M.T. Quilty, J.M. Kelley, R.B. Davis, et al., *Serum correlates of the placebo effect in irritable bowel syndrome.* Neurogastroenterol Motil, 2010. **22**(3): p. 285-e281.
528. Kou, Y., Q. Zhao, X.L. Wen, and B. Yang, *Clinical observation on TCM triple therapy in treating diarrhea-pattern irritable bowel syndrome.* Western journal of traditional chinese medicine [xi bu zhong yi yao], 2015. **28**(11): p. 79‐81.
529. Krasaelap, A., M.R. Sood, B.U.K. Li, R. Unteutsch, K. Yan, M. Nugent, et al., *Efficacy of Auricular Neurostimulation in Adolescents With Irritable Bowel Syndrome in a Randomized, Double-Blind Trial.* Clinical gastroenterology and hepatology, 2020. **18**(9): p. 1987‐1994.e1982.
530. Kunze, M., H.J. Seidel, and G. Stübe, *Comparative studies of the effectiveness of brief psychotherapy, acupuncture and papaverin therapy in patients with irritable bowel syndrome.* Zeitschrift fur die gesamte innere Medizin und ihre Grenzgebiete, 1990. **45**(20): p. 625‐627.
531. Li, X.Q., S.Y. Mu, Q. Lu, and X. Lu, *Therapeutic observation of diarrhea-predominant irritable bowel syndrome majorly treated by acupuncture with Ling Gui Ba Fa.* Shanghai journal of acupuncture and moxibustion [shang hai zhen jiu za zhi], 2015. **34**(1): p. 22‐24.
532. Ling, Y., L. Yuan, Z. Haifeng, M. Xiaopeng, B. Chunhui, W. Huangan, et al., *Effect of warming moxibustion Tianshu (ST 25, bilateral) and Qihai (CV 6) for the treatment of diarrhea-dominant irritable bowel syndrome: a patient-blinded pilot trial with orthogonal design.* Journal of traditional Chinese medicine / Chung i tsa chih ying wen pan, 2017. **37**(4): p. 538‐545.
533. Liu, D., M. Ruan, C. Tong, and R. Huang, *Effect of Shugan Jianpi Recipe Combined with Cross Moxibustion on Biochemical Examination Indexes and Total Score of TCM Symptoms in Patients with Spleen-Stomach Damp-Heat Diarrhea Irritable Bowel Syndrome.* Comput Math Methods Med, 2022. **2022**: p. 8286146.
534. Liu, H., *Regulation of acupuncture and moxibuxtion on purine receptors in ibs visceral pain.* Purinergic Signalling, 2018. **14**(1): p. S72-S73.
535. Liu, H., C. Bao, J. Li, Z. Hu, Y. Shi, J. Shen, et al., *EFFECT OF MOXIBUSTION ON SYMPTOMS AND EVENT-RALATED POTENTIAL IN IRRITABLE BOWEL SYNDROME: A RANDOMIZED CONTROLLED TRIAL.* Gastroenterology, 2020. **158**(6): p. S-852.
536. Liu, Y.C., *Clinical study of ascending-descending acupuncture method regulation of the spleen and stomach for treatment of Spleen Deficiency-type diarrhea in irritable bowel syndrome.* Shanghai journal of acupuncture and moxibustion [shang hai zhen jiu za zhi], 2015. **34**(1): p. 25‐27.
537. Lu, C.L. and F.Y. Chang, *Placebo effect in patients with irritable bowel syndrome.* Journal of Gastroenterology and Hepatology (Australia), 2011. **26**(SUPPL. 3): p. 116-118.
538. MacPherson, H., H. Tilbrook, D. Agbedjro, H. Buckley, C. Hewitt, and C. Frost, *Acupuncture for irritable bowel syndrome: 2-year follow-up of a randomised controlled trial.* Acupunct Med, 2017. **35**(1): p. 17-23.
539. Minocha, A. and T. Abell, *Irritable bowel syndrome in the older adult.* Geriatrics and Aging, 2004. **7**(10): p. 15-19.
540. Mion, F., S. Pellissier, A. Garros, H. Damon, S. Roman, and B. Bonaz, *Transcutaneous auricular vagus nerve stimulation for the treatment of irritable bowel syndrome: a pilot, open-label study.* Bioelectronics in medicine, 2020. **3**(1): p. 167‐174.
541. Moayyedi, P., C.N. Andrews, G. MacQueen, C. Korownyk, M. Marsiglio, L. Graff, et al., *Canadian Association of Gastroenterology Clinical Practice Guideline for the Management of Irritable Bowel Syndrome (IBS).* J Can Assoc Gastroenterol, 2019. **2**(1): p. 6-29.
542. Mountifield, R. and J.M. Andrews, *Managing irritable bowel syndrome.* Medicine Today, 2010. **11**(2): p. 32-40.
543. Nct, *Acupuncture for Irritable Bowel Syndrome.* https://clinicaltrials.gov/show/NCT00065403, 2003.
544. Nct, *Acupuncture for Irritable Bowel Syndrome (IBS).* https://clinicaltrials.gov/show/NCT00093327, 2004.
545. Nct, *Effect of Acupuncture on Symptoms of Diarrhea and Pain in IBS.* https://clinicaltrials.gov/show/NCT00219505, 2005.
546. Nct, *Acupuncture/Moxibustion for Irritable Bowel Syndrome (IBS).* https://clinicaltrials.gov/show/NCT00945074, 2009.
547. Nct, *Effect of Electroacupuncture in Patients With Irritable Bowel Syndrome.* https://clinicaltrials.gov/show/NCT00900965, 2009.
548. Nct, *Symptom Management for Irritable Bowel Syndrome.* https://clinicaltrials.gov/show/NCT02079857, 2014.
549. Nct, *Percutaneous Electrical Nerve Field Stimulation for Adults With Irritable Bowel Syndrome.* https://clinicaltrials.gov/show/NCT04428619, 2020.
550. Nct, *Acupuncture for Irritable Bowel Syndrome Patients.* https://clinicaltrials.gov/show/NCT04387383, 2020.
551. Nct, *Acupuncture-like Transcutaneous Electrical Nerve Stimulation on Bowel Symptom and Quality of Life in Patients With Irritable Bowel Syndrome.* https://clinicaltrials.gov/show/NCT05042661, 2021.
552. Nct, *Self- Administered Acupressure for Diarrhea Predominant Irritable Bowel Syndrome.* https://clinicaltrials.gov/show/NCT05702255, 2023.
553. Osemene, N.I., *Irritable bowel syndrome in children and adolescents*. 2015. p. 46-50.
554. Qu, L., L. Xing, W. Norman, H. Chen, and S. Gao, *Irritable bowel syndrome treated by traditional Chinese spinal orthopedic manipulation.* Journal of traditional chinese medicine = chung i tsa chih ying wen pan, 2012. **32**(4): p. 565‐570.
555. Reynolds, J.A., J.M. Bland, and H. MacPherson, *Acupuncture for irritable bowel syndrome - An exploratory randomised controlled trial.* Acupuncture in Medicine, 2008. **26**(1): p. 8-16.
556. Shen, J.H., Y.M. Ye, K.X. Zhu, and S.S. Li, *Acupuncture for diarrhea-predominant irritable bowel syndrome: a randomized control study.* World journal of acupuncture - moxibustion, 2022. **32**(2): p. 123‐130.
557. Smith, G.D., D.T. Steinke, M. Kinnear, K.I. Penny, N. Pathmanathan, and I.D. Penman, *A comparison of irritable bowel syndrome patients managed in primary and secondary care: The episode IBS study.* British Journal of General Practice, 2004. **54**(504): p. 503-507.
558. Stamuli, E., K. Bloor, H. MacPherson, H. Tilbrook, T. Stuardi, S. Brabyn, et al., *Cost-effectiveness of acupuncture for irritable bowel syndrome: findings from an economic evaluation conducted alongside a pragmatic randomised controlled trial in primary care.* BMC Gastroenterol, 2012. **12**: p. 149.
559. Stuardi, T. and H. MacPherson, *Acupuncture for irritable bowel syndrome: diagnosis and treatment of patients in a pragmatic trial.* J Altern Complement Med, 2012. **18**(11): p. 1021-1027.
560. Sun, Y.Z. and J. Song, *Therapeutic observation of acupuncture at Jiaji (EX-B2) for irritable bowel syndrome.* Shanghai journal of acupuncture and moxibustion [shang hai zhen jiu za zhi], 2015. **34**(9): p. 856‐857.
561. Videlock, E.J., F. Cremonini, E.A. Friedlander, T. Kaptchuk, and A. Lembo, *Is the McGill pain questionnaire less sensitive to change in response to a placebo treatment in irritable bowel syndrome in comparison to a single item pain severity scale?* Gastroenterology, 2011. **140**(5): p. S610‐S611.
562. Wang, L. and S. Zhang, *Therapeutic study on scalp acupuncture plus body acupuncture for diarrhea-predominant irritable bowel syndrome.* Shanghai journal of acupuncture and moxibustion [shang hai zheng jiu za zhi], 2016. **35**(4): p. 405‐407.
563. Wang, R.S., A.J. Lembo, T.J. Kaptchuk, V. Cheng, J. Nee, J. Iturrino, et al., *Genomic Effects Associated With Response to Placebo Treatment in a Randomized Trial of Irritable Bowel Syndrome.* Front Pain Res (Lausanne), 2021. **2**: p. 775386.
564. Wu, J.C., E.T. Ziea, L. Lao, E.F. Lam, C.S. Chan, A.Y. Liang, et al., *Effect of electroacupuncture on visceral hyperalgesia, serotonin and fos expression in an animal model of irritable bowel syndrome.* J Neurogastroenterol Motil, 2010. **16**(3): p. 306-314.
565. Xiao, W.B. and Y.L. Liu, *Rectal hypersensitivity reduced by acupoint TENS in patients with diarrhea-predominant irritable bowel syndrome: a pilot study.* Dig Dis Sci, 2004. **49**(2): p. 312-319.
566. Yaklai, K., S. Pattanakuhar, N. Chattipakorn, and S.C. Chattipakorn, *The Role of Acupuncture on the Gut-Brain-Microbiota Axis in Irritable Bowel Syndrome.* American Journal of Chinese Medicine, 2021. **49**(2): p. 285-314.
567. Yang, J.Y., D.Q. Tao, Y. Zeng, J. Yang, D. Xu, T. Shi, et al., *Modified Shenling Baizhu powder combined with electroacupuncture for treatment of diarrhea-predominant irritable bowel syndrome: Effect on clinical symptoms and psychological state.* World Chinese Journal of Digestology, 2017. **25**(12): p. 1115-1122.
568. Yu, L.M., Y.L. Zhang, Y.W. Wang, W. Ye, and B. Lu, *Umbilicus acupuncture for treatment of diarrhea-type irritable bowel syndrome: efficacy and impact on brain-gut peptides.* World chinese journal of digestology, 2020. **28**(13): p. 538‐543.
569. Zhan, D.W., J.H. Sun, K.T. Luo, L.Z. Xu, J.L. Zhou, L.X. Pei, et al., *Effects and efficacy observation of acupuncture on serum 5-HT in patients with diarrhea-predominant irritable bowel syndrome.* Zhongguo zhen jiu [Chinese acupuncture & moxibustion], 2014. **34**(2): p. 135‐138.
570. Zhao, J.M., L.Y. Wu, H.R. Liu, H.Y. Hu, J.Y. Wang, R.J. Huang, et al., *Factorial study of moxibustion in treatment of diarrhea-predominant irritable bowel syndrome.* World journal of gastroenterology, 2014. **20**(37): p. 13563‐13572.
571. Zhao, M., Z. Wang, Z. Weng, F. Zhang, G. Li, Z. Ma, et al., *Electroacupuncture Improves IBS Visceral Hypersensitivity by Inhibiting the Activation of Astrocytes in the Medial Thalamus and Anterior Cingulate Cortex.* Evid Based Complement Alternat Med, 2020. **2020**: p. 2562979.
572. Xiong, X.R. and Y. Lin, *Acupuncture and moxibustion and TCM simultaneously for treating Diarrhea-predominant irritable bowel syndrome in 42 Cases.* Journal of fujian university of TCM [fu jian zhong yi xue yuan xue bao], 2008. **18**(2): p. 38‐40.
573. Passos, M.C.F., A.J. Lembo, L.A. Conboy, T.J. Kaptchuk, J.M. Kelly, M.T. Quilty, et al., *Adequate relief in a treatment trial with IBS patients: A prospective assessment.* American Journal of Gastroenterology, 2009. **104**(4): p. X912-919.
574. Liang, Y.S., S. Xie, Y. Zhang, L.P. Wang, and C.L. Zhu, *Treatment of 40 cases of irritable bowel syndrome by acupoint thread burial therapy and taijiquan.* Shanghai journal of traditional chinese medicine [shang hai zhong yi yao za zhi], 2010. **44**(10): p. 38‐39; 43.
575. Zhang, H.C., S.K. Han, and J.L. Tang, *Tonifying qi Invigorating blood Dispersing wind combined with scalp acupuncture for treating diarrhea-predominant irritable bowel syndrome in 50 Cases.* Journal of traditional chinese medicine [zhong yi za zhi], 2010. **51**(Sl): p. 220‐221.
576. Comito, D., A. Famiani, S. Calamara, S. Cardile, V. Ferrau, A. Chiaro, et al., *Efficacy of complementary therapywith partially hydrolyzed guar gum (PHGG) in functional gastrointestinal disorders (FGID): a first pediatric randomized placebo controlled trial (RCT).* Digestive and liver disease, 2011. **43**: p. S443‐S444.
577. Ma, T.H. and S.J. Zhang, *Shenling Baizhu San combined with acupoint RN 8 application for treating diarrhea-predominant irritable bowel syndrome in 72 Cases.* Hebei journal of traditional chinese medicine [he bei zhong yi], 2011. **31**(1): p. 48‐49.
578. Ge, J.J. and K.X. Zeng, *Efficacy observation on warm needling for 60 cases of diarrhea irritable bowel syndrome.* World journal of acupuncture - moxibustion, 2013. **23**(4): p. 43‐51+45.
579. Wang, S.W., *Forty-two cases of ulcerative colitis treated with comprehensive TCM therapy.* Henan traditional chinese medicine [henan zhong yi], 2016. **36**(4): p. 643‐645.
580. *Clinical observation on combination of warm acupuncture and ear pressing for the treatment of 46 cases of irritable bowel syndrome.* Hunan guiding journal of traditional chinese medicine [hu nan zhong yi yao dao bao], 2004. **10**(3): p. 42.
581. Chen, G.Y., M. Liang, D. Chen, and J.H. Hua, *Ping Ji San combined with acupoints injection for treating irritable bowel syndrome in 70 cases.* Chinese journal of traditional medical science and technology [zhong guo zhong yi yao ke ji], 2005. **12**(5): p. 322‐323.
582. Chen, X.Y., Y.H. Huang, and S.F. Yu, *Regulating Liver and Spleen Regimen combined with acupuncture for treating diarrhea-predominant Irritable Bowel Syndrome in 50 Cases.* Shanxi journal of traditional chinese medicine [shan xi zhong yi], 2005. **26**(1): p. 32‐33.
583. Chen, G.Y., M. Liang, S.G. Xu, and D.L. Chen, *Treating IBS with Chinese medicine combined with acupuncture point injection and psychotherapy in 82 cases.* Chinese journal of integrated traditional and western medicine on digestion [zhong guo zhong xi yi jie he xiao hua za zhi], 2006. **14**(2): p. 124‐125.
584. Anastasi, J.K., D.J. McMahon, and G.H. Kim, *Symptom management for irritable bowel syndrome: a pilot randomized controlled trial of acupuncture/moxibustion.* Gastroenterol Nurs, 2009. **32**(4): p. 243-255.
585. Hu, D., M.F. Kang, J. Xiong, and P. Deng, *Irritable bowel syndrome with diarrhea (IBS-D) treated with moxibustion on heat-sensitive acupoints: a randomized controlled trial.* World journal of acupuncture - moxibustion, 2012. **22**(2): p. 1‐5.
586. Park, H.J. and C. Cha, *The effect of Korean hand acupuncture on young, single Korean students with irritable bowel syndrome.* Gastroenterol Nurs, 2012. **35**(6): p. 403-414.
587. Zhu, Y., Z. Wu, X. Ma, H. Liu, C. Bao, L. Yang, et al., *Brain regions involved in moxibustion-induced analgesia in irritable bowel syndrome with diarrhea: a functional magnetic resonance imaging study.* BMC Complement Altern Med, 2014. **14**: p. 500.
588. Shi, Y., Y.H. Chen, X.J. Yin, A.Q. Wang, X.K. Chen, J.H. Lu, et al., *Electroacupuncture versus moxibustion for irritable bowel syndrome: a randomized, parallel-controlled trial.* Evidence-based complementary and alternative medicine, 2015. **2015**.
589. Zhao, J.M., J.H. Lu, X.J. Yin, X.K. Chen, Y.H. Chen, W.J. Tang, et al., *Comparison of electroacupuncture and moxibustion on brain-gut function in patients with diarrhea-predominant irritable bowel syndrome: A randomized controlled trial.* Chin J Integr Med, 2015. **21**(11): p. 855-865.
590. Zhenzhong, L., Y. Xiaojun, T. Weijun, C. Yuehua, S. Jie, Z. Jimeng, et al., *Comparative effect of electroacupuncture and moxibustion on the expression of substance P and vasoactive intestinal peptide in patients with irritable bowel syndrome.* J Tradit Chin Med, 2015. **35**(4): p. 402-410.
591. Zhang, W. and F. Zhong, *Two-way adjusting effect of acupuncture on Tianshu (ST25) and Dachangshu (BL25) for treating functional bowel disease.* Planta Medica, 2016. **82**(5).
592. Liu, H., C. Bao, C. Wang, Y. Yan, J. Li, Z. Hu, et al., *LONG-TERM EFFECT OF MOXIBUSTION ON IRRITABLE BOWEL SYNDROME: a RANDOMIZED CLINCAL TRIAL.* Gastroenterology, 2018. **154**(6): p. S‐196.
593. Zhang, C., L. Guo, Y. Wang, L. Zhang, and T. Chang, *Electroacupuncture combined with qibei mixture for diarrhea-predominant irritable bowel syndrome: a randomized controlled trial.* World journal of acupuncture - moxibustion, 2018. **28**(1): p. 19‐24.
594. Zhao, J.M., J.H. Lu, X.J. Yin, L.Y. Wu, C.H. Bao, X.K. Chen, et al., *Comparison of Electroacupuncture and Mild-Warm Moxibustion on Brain-Gut Function in Patients with Constipation-Predominant Irritable Bowel Syndrome: a Randomized Controlled Trial.* Chinese journal of integrative medicine, 2018. **24**(5): p. 328‐335.
595. Guo, J., J.H. Sun, L. Chen, H. Geng, G.H. Yang, R.R. Shen, et al., *Bidirectional regulation of acupuncture: a subgroup analysis of multicenter randomized controlled trial of acupuncture with Tiaoshen Jianpi for irritable bowel syndrome.* Zhongguo zhen jiu [Chinese acupuncture & moxibustion], 2021. **41**(8): p. 845‐850.
596. Bao, C., L. Wu, Y. Shi, Z. Shi, X. Jin, J. Shen, et al., *Long-term effect of moxibustion on irritable bowel syndrome with diarrhea: a randomized clinical trial.* Therap Adv Gastroenterol, 2022. **15**: p. 17562848221075131.
597. Wang, Z., M. Xu, Z. Shi, C. Bao, H. Liu, C. Zhou, et al., *Mild moxibustion for Irritable Bowel Syndrome with Diarrhea (IBS-D): a randomized controlled trial.* Journal of ethnopharmacology, 2022. **289**: p. 115064.
598. Almakadma, A.H., A. De Vol, M.S. Alabdaljabar, S. Aldosari, I. Muhsen, O. Alfreihi, et al., *Complementary and alternative medicine use and its association with medication adherence in inflammatory bowel disease and other gastrointestinal diseases.* Saudi Journal of Gastroenterology, 2023. **29**(4): p. 233-239.
599. Liu, H., C. Bao, J. Li, Z. Hu, Y. Shi, J. Shen, et al., *EFFECT OF MOXIBUSTION ON SYMPTOMS AND EVENT-RALATED POTENTIAL IN IRRITABLE BOWEL SYNDROME: A RANDOMIZED CONTROLLED TRIAL.* Gastroenterology, 2020. **158**(6): p. S-852.
600. Nct, *Acupuncture for Patients With Diarrhea-predominant IBS or Functional Diarrhea: a Randomized Controlled Trial.* https://clinicaltrials.gov/show/NCT01350570, 2011.
601. ChiCtr, *A randomized controlled trial on acupuncture treatment of diarrheal-predominant irritable bowel syndrome.* https://trialsearch.who.int/Trial2.aspx?TrialID=ChiCTR2100044762, 2021.
602. ChiCtr, *Clinical observation of acupoint application in the treatment of diarrhea-type irritable bowel syndrome.* https://trialsearch.who.int/Trial2.aspx?TrialID=ChiCTR2200064123, 2022.
603. ChiCtr, *A randomized controlled study of acupuncture in the treatment of diarrhea-predominant irritable bowel syndrome.* https://trialsearch.who.int/Trial2.aspx?TrialID=ChiCTR2200064619, 2022.
604. Itmctr, *Clinical observation of acupoint application in the treatment of diarrhea-type irritable bowel syndrome.* https://trialsearch.who.int/Trial2.aspx?TrialID=ITMCTR2200006666, 2022.
605. Itmctr, *A randomized controlled study of acupuncture in the treatment of diarrhea-predominant irritable bowel syndrome.* https://trialsearch.who.int/Trial2.aspx?TrialID=ITMCTR2200006702, 2022.
606. Itmctr, *Clinical efficacy evaluation of taVNS for patients with IBS-D.* https://trialsearch.who.int/Trial2.aspx?TrialID=ITMCTR2200006465, 2022.
607. ChiCtr, *Acupuncture treatment based on sensitive points for Irritable bowel syndrome with diarrhea : a randomised controlled trial.* https://trialsearch.who.int/Trial2.aspx?TrialID=ChiCTR2300074179, 2023.
608. Nct, *Aerobic Exercise Verus Acupuncture on the Quality of Life in Women Suffering From Irritable Bowel Syndrome.* https://clinicaltrials.gov/ct2/show/NCT05947929, 2023.
609. Nct, *Laser Acupuncture on Irritable Bowel Syndrome in Females.* https://clinicaltrials.gov/show/NCT05757037, 2023.
610. Actrn, *A pilot study of traditional acupuncture as an additional treatment for irritable bowel syndrome-diarrhoea.* https://trialsearch.who.int/Trial2.aspx?TrialID=ACTRN12622000073707, 2022.
611. Nct, *Acupuncture-like Transcutaneous Electrical Nerve Stimulation on Bowel Symptom and Quality of Life in Patients With Irritable Bowel Syndrome.* https://clinicaltrials.gov/show/NCT05042661, 2021.
612. Nct, *Acupuncture for Irritable Bowel Syndrome Patients.* https://clinicaltrials.gov/show/NCT04387383, 2020.
613. Fireman, Z., A. Segal, Y. Kopelman, A. Sternberg, and R. Carasso, *Acupuncture treatment for irritable bowel syndrome. A double-blind controlled study.* Digestion, 2001. **64**(2): p. 100‐103.
614. Chen, X.Y., Y.H. Huang, and S.F. Yu, *Regulating Liver and Spleen Regimen combined with acupuncture for treating diarrhea-predominant Irritable Bowel Syndrome in 50 Cases.* Shanxi journal of traditional chinese medicine [shan xi zhong yi], 2005. **26**(1): p. 32‐33.
615. Kaptchuk, T.J., J.M. Kelley, L.A. Conboy, R.B. Davis, C.E. Kerr, E.E. Jacobson, et al., *Components of placebo effect: randomised controlled trial in patients with irritable bowel syndrome.* BMJ (Clinical research ed.), 2008. **336**(7651): p. 999‐1003.
616. Schneider, A., P. Enck, K. Streitberger, S. Joos, C. Weiland, S. Bagheri, et al., *Specific physiological and unspecific psychological effects during acupuncture treatment in patients with irritable bowel syndrome: results of a randomised controlled trial.* Revista internacional de acupuntura, 2008. **2**(3): p. 146‐154.
617. Dong, J. and H. Zhang, *Clinical observation of calming-intestine scattered acupoints applying for treating diarrhea-predominant irritable bowel syndrome.* Journal of new chinese medicine [xin zhong yi], 2011. **43**(8): p. 106‐107.
618. Rafiei, R., M. Ataie, M.A. Ramezani, A. Etemadi, B. Ataei, H. Nikyar, et al., *A new acupuncture method for management of irritable bowel syndrome: A randomized double blind clinical trial.* J Res Med Sci, 2014. **19**(10): p. 913-917.
619. Wu, X., C.H. Zheng, X.H. Xu, P. Ding, F. Xiong, M. Tian, et al., *- Electroacupuncture for Functional Constipation: A Multicenter, Randomized, Control Trial.* 2017. **- 2017**.
620. Pei, L., H. Chen, J. Guo, L. Chen, X. Wu, W. Xu, et al., *Effect of acupuncture and its influence on visceral hypersensitivity in IBS-D patients: Study protocol for a randomized controlled trial.* Medicine (Baltimore), 2018. **97**(21): p. e10877.
621. Pei, L.X., H. Chen, J. Guo, L. Chen, X.L. Wu, W.L. Xu, et al., *- Effect of acupuncture and its influence on visceral hypersensitivity in IBS-D patients: Study protocol for a randomized controlled trial.* 2018. **- 97**(- 21).
622. Zheng, H., Z.S. Liu, W. Zhang, M. Chen, F. Zhong, X.H. Jing, et al., *- Acupuncture for patients with chronic functional constipation: A randomized controlled trial.* 2018. **- 30**(- 7).
623. Zheng, H., J. Xu, X. Sun, F. Zeng, Y. Li, X. Wu, et al., *- Electroacupuncture for patients with refractory functional dyspepsia: A randomized controlled trial.* 2018. **- 30**(- 7).
624. Luo, Q.P., S.S. Kuang, X.F. Yang, L.M. Jin, P.B. Chen, Y. Chen, et al., *Bowls-unblocking and mind-regulating acupuncture therapy for constipation-predominant irritable bowel syndrome: an exploratory randomized clinical trial 通腑调神针法治疗便秘型肠易激综合征: 随机对照试验.* World journal of acupuncture - moxibustion, 2023.
625. *Irritable bowel syndrome: A mild disorder; Purely symptomatic treatment.* Prescrire International, 2009. **18**(100): p. 75-79.
626. Alammar, N. and E. Stein, *Irritable Bowel Syndrome: What Treatments Really Work.* Medical Clinics of North America, 2019. **103**(1): p. 137-152.
627. Bethards, D.M. and A. Ouyang, *Irritable Bowel Syndrome: Diagnosis and management.* Practical Gastroenterology, 2007. **31**(3): p. 14-40.
628. Chang, F.Y. and C.L. Lu, *Treatment of irritable bowel syndrome using complementary and alternative medicine.* J Chin Med Assoc, 2009. **72**(6): p. 294-300.
629. Chang, F.Y. and C.L. Lu, *Irritable bowel syndrome and migraine: Bystanders or partners?* Journal of Neurogastroenterology and Motility, 2013. **19**(3): p. 301-311.
630. Chang, J.Y. and N.J. Talley, *Current and emerging therapies in irritable bowel syndrome: From pathophysiology to treatment.* Trends in Pharmacological Sciences, 2010. **31**(7): p. 326-334.
631. Chen, G.G., S. Guo, X.L. Su, Q. Liu, and W. Wei, *Research progress of moxibustion in treatment of irritable bowel syndrome.* World Journal of Acupuncture - Moxibustion, 2021. **31**(2): p. 136-140.
632. Chey, W.D., M. Maneerattaporn, and R. Saad, *Pharmacologic and complementary and alternative medicine therapies for irritable bowel syndrome.* Gut and Liver, 2011. **5**(3): p. 253-266.
633. Chin Feman, S.P., L.T. Nguyen, M.T. Quilty, C.E. Kerr, B.H. Nam, L.A. Conboy, et al., *Effectiveness of recruitment in clinical trials: an analysis of methods used in a trial for irritable bowel syndrome patients.* Contemp Clin Trials, 2008. **29**(2): p. 241-251.
634. Chiou, E. and S. Nurko, *Management of functional abdominal pain and irritable bowel syndrome in children and adolescents.* Expert Review of Gastroenterology and Hepatology, 2010. **4**(3): p. 293-304.
635. Chiou, E. and S. Nurko, *Functional abdominal pain and irritable bowel syndrome in children and adolescents.* Therapy, 2011. **8**(3): p. 315-331.
636. El-Salhy, M., D. Gundersen, J.G. Hatlebakk, and T. Hausken, *Irritable bowel syndrome: Treatment options.* Clinical Practice, 2012. **9**(5): p. 591-600.
637. Farhadi, A., K. Bruninga, J. Fields, and A. Keshavarzian, *Irritable bowel syndrome: an update on therapeutic modalities.* Expert Opin Investig Drugs, 2001. **10**(7): p. 1211-1222.
638. Farzaei, M.H., R. Bahramsoltani, M. Abdollahi, and R. Rahimi, *The role of visceral hypersensitivity in irritable bowel syndrome: Pharmacological targets and novel treatments.* Journal of Neurogastroenterology and Motility, 2016. **22**(4): p. 558-574.
639. Grundmann, O. and S.L. Yoon, *Irritable bowel syndrome: epidemiology, diagnosis and treatment: an update for health-care practitioners.* J Gastroenterol Hepatol, 2010. **25**(4): p. 691-699.
640. Grundmann, O. and S.L. Yoon, *Complementary and alternative medicines in irritable bowel syndrome: an integrative view.* World J Gastroenterol, 2014. **20**(2): p. 346-362.
641. Gupta, S., G. Schaffer, and M. Saps, *Pediatric irritable bowel syndrome and other functional abdominal pain disorders: an update of non-pharmacological treatments.* Expert Rev Gastroenterol Hepatol, 2018. **12**(5): p. 447-456.
642. Harris, L.A. and L. Chang, *Irritable bowel syndrome: New and emerging therapies.* Current Opinion in Gastroenterology, 2006. **22**(2): p. 128-135.
643. Harris, L.A., S. Hansel, J. DiBaise, and M.D. Crowell, *Irritable bowel syndrome and chronic constipation: Emerging drugs, devices, and surgical treatments.* Current Gastroenterology Reports, 2006. **8**(4): p. 282-290.
644. Hosseini, A., S. Nikfar, and M. Abdollahi, *Probiotics use to treat irritable bowel syndrome.* Expert Opinion on Biological Therapy, 2012. **12**(10): p. 1323-1334.
645. Huang, J., M. Lu, Y. Zheng, J. Ma, X. Ma, Y. Wang, et al., *Quality of Evidence Supporting the Role of Acupuncture for the Treatment of Irritable Bowel Syndrome.* Pain Research and Management, 2021. **2021**.
646. Huang, K.Y., F.Y. Wang, M. Lv, X.X. Ma, X.D. Tang, and L. Lv, *Irritable bowel syndrome: Epidemiology, overlap disorders, pathophysiology and treatment.* World Journal of Gastroenterology, 2023. **29**(26): p. 4120-4135.
647. Lacy, B.E. and R.D. Lee, *Irritable bowel syndrome: A syndrome in evolution.* Journal of Clinical Gastroenterology, 2005. **39**(5 SUPPL. 3): p. S230-S242.
648. Lacy, B.E., K. Weiser, and R. De Lee, *The treatment of irritable bowel syndrome.* Therapeutic Advances in Gastroenterology, 2009. **2**(4): p. 221-238.
649. Leahy, A. and O. Epstein, *Non-pharmacological treatments in the irritable bowel syndrome.* World Journal of Gastroenterology, 2001. **7**(3): p. 313-316.
650. Lim, B., E. Manheimer, L. Lao, E. Ziea, J. Wisniewski, J. Liu, et al., *Acupuncture for treatment of irritable bowel syndrome.* Cochrane Database Syst Rev, 2006(4): p. Cd005111.
651. Magge, S. and A. Lembo, *Complementary and alternative medicine for the irritable bowel syndrome.* Gastroenterol Clin North Am, 2011. **40**(1): p. 245-253.
652. Magge, S.S. and J.L. Wolf, *Complementary and alternative medicine and mind-body therapies for treatment of irritable bowel syndrome in women.* Womens Health (Lond), 2013. **9**(6): p. 557-567.
653. Malone, M.A., *Irritable Bowel Syndrome.* Primary Care - Clinics in Office Practice, 2011. **38**(3): p. 433-447.
654. Manheimer, E., K. Cheng, L.S. Wieland, L.S. Min, X. Shen, B.M. Berman, et al., *Acupuncture for treatment of irritable bowel syndrome.* Cochrane Database Syst Rev, 2012. **5**(5): p. Cd005111.
655. Mearin, F., C. Ciriza, M. Mínguez, E. Rey, J.J. Mascort, E. Peña, et al., *Clinical Practice Guideline: Irritable bowel syndrome with constipation and functional constipation in the adult.* Rev Esp Enferm Dig, 2016. **108**(6): p. 332-363.
656. Moayyedi, P., M. Marsiglio, C.N. Andrews, L.A. Graff, C. Korownyk, B. Kvern, et al., *Patient engagement and multidisciplinary involvement has an impact on clinical guideline development and decisions: A comparison of two irritable bowel syndrome guidelines using the same data.* Journal of the Canadian Association of Gastroenterology, 2019. **2**(1): p. 30-36.
657. Pisipati, S., B.A. Connor, and M.S. Riddle, *Updates on the epidemiology, pathogenesis, diagnosis, and management of postinfectious irritable bowel syndrome.* Curr Opin Infect Dis, 2020. **33**(5): p. 411-418.
658. Rao, V.L., A.S. Cifu, and L.W. Yang, *Pharmacologic management of irritable bowel syndrome.* JAMA - Journal of the American Medical Association, 2015. **314**(24): p. 2684-2685.
659. Sandhu, B.K. and S.P. Paul, *Irritable bowel syndrome in children: Pathogenesis, diagnosis and evidence-based treatment.* World Journal of Gastroenterology, 2014. **20**(20): p. 6013-6023.
660. Shen, Y.H. and R. Nahas, *Complementary and alternative medicine for treatment of irritable bowel syndrome.* Can Fam Physician, 2009. **55**(2): p. 143-148.
661. Tillisch, K. and L. Chang, *Diagnosis and treatment of irritable bowel syndrome: State of the art.* Current Gastroenterology Reports, 2005. **7**(4): p. 249-256.
662. Usai-Satta, P., M. Bellini, M. Lai, F. Oppia, and F. Cabras, *Therapeutic approach for irritable bowel syndrome: Old and new Strategies.* Current Clinical Pharmacology, 2018. **13**(3): p. 164-172.
663. Wang, X., X. Shi, J. Lv, J. Zhang, Y. Huo, G. Zuo, et al., *Acupuncture and related therapies for the anxiety and depression in irritable bowel syndrome with diarrhea (IBS-D): A network meta-analysis of randomized controlled trials.* Front Psychiatry, 2022. **13**: p. 1067329.
664. Wu, J.C.Y., *Complementary and alternative medicine modalities for the treatment of irritable bowel syndrome: Facts or myths?* Gastroenterology and Hepatology, 2010. **6**(11): p. 705-711.
665. Yan, H. and Q. Du, *Advances in the treatment of irritable bowel syndrome.* Chinese Journal of Gastroenterology, 2007. **12**(7): p. 442-445.
666. Yoon, S.L., O. Grundmann, L. Koepp, and L. Farrell, *Management of irritable bowel syndrome (IBS) in adults: conventional and complementary/alternative approaches.* Altern Med Rev, 2011. **16**(2): p. 134-151.
