# Supplementary material for: The effect of acupuncture on quality of life in patients with irritable bowel syndrome: A systematic review and meta-analysis
Source: PLoS One. 2025 Feb 13;20(2):e0314678. doi: 10.1371/journal.pone.0314678 (PMC11824959; doi:10.1371/journal.pone.0314678)
Supplement: S2 File — (DOCX) [file pone.0314678.s006.docx]

**Supplementary File 2. Search strategy used in different databases**

| **Pubmed** | | |
| --- | --- | --- |
| **#1** | Irritable Bowel Syndrome[MeSH Terms] | **9,490** |
| **#2** | (Irritable Bowel Syndromes[Title/Abstract]) OR (Syndrome, Irritable Bowel[Title/Abstract]) OR (Syndromes, Irritable Bowel[Title/Abstract]) OR (Colon, Irritable[Title/Abstract]) OR (Irritable Colon[Title/Abstract]) OR (Colitis, Mucous[Title/Abstract]) OR (Colitides, Mucous[Title/Abstract]) OR (Mucous Colitides[Title/Abstract]) OR (Mucous Colitis[Title/Abstract]) OR (IBS[Title/Abstract]) | **12,100** |
| **#3** | acupuncture[MeSH Terms] | **30,138** |
| **#4** | "acupuncture"[Title/Abstract] OR "Auriculotherapy"[Title/Abstract] OR "Acupressure"[Title/Abstract] OR "transcutaneous electric nerve stimulation"[Title/Abstract] OR "Electroacupuncture"[Title/Abstract] OR "electro acupuncture"[Title/Abstract] OR "acupuncture points"[Title/Abstract] OR "acupuncture point"[Title/Abstract] OR "Acupoint"[Title/Abstract] OR "Acupoints"[Title/Abstract] OR "transcutaneous electric nerve stimulation"[Title/Abstract] OR "transcutaneous electric stimulation"[Title/Abstract] OR "percutaneous electric nerve stimulation"[Title/Abstract] OR "TENS"[Title/Abstract] OR "transcutaneous electrical stimulation"[Title/Abstract] OR "percutaneous electrical nerve stimulation"[Title/Abstract] OR "transcutaneous electrical nerve stimulation"[Title/Abstract] OR "transcutaneous nerve stimulation"[Title/Abstract] OR "percutaneous neuromodulation therapy"[Title/Abstract] OR "percutaneous neuromodulation therapies"[Title/Abstract] OR "percutaneous electrical neuromodulation"[Title/Abstract] OR "transdermal electrostimulation"[Title/Abstract] OR "transcutaneous electric nerve stimulation"[Title/Abstract] OR "analgesic cutaneous electrostimulation"[Title/Abstract] OR "scalp acupuncture"[Title/Abstract] OR "acupuncture ear"[Title/Abstract] OR "auricular acupuncture"[Title/Abstract] OR "ear acupuncture"[Title/Abstract] OR "auricular acupunctures"[Title/Abstract] OR "auricular points"[Title/Abstract] OR "auricular point"[Title/Abstract] OR "ear points"[Title/Abstract] OR "ear point"[Title/Abstract] OR "Meridian"[Title/Abstract] OR "dermal needle"[Title/Abstract] OR "plum blossom needle"[Title/Abstract] OR "seven star needle"[Title/Abstract] OR "catgut embedding"[Title/Abstract] OR "fire needle"[Title/Abstract] OR "Meridians"[Title/Abstract] | **60,257** |
| **#5** | #1 OR #2 | **15,184** |
| **#6** | #3 OR #4 | **60,282** |
| **#7** | (clinical[tiab] AND trial[tiab]) OR "clinical trials as topic"[mesh] OR "clinical trial"[pt] OR random*[tiab] OR "random allocation"[mesh] OR "therapeutic use"[sh] | **636,772,2** |
| **#8** | #5 AND #6 #7 | **205** |

| **Embse** | | |
| --- | --- | --- |
| **#1** | 'irritable colon'/exp | **32,494** |
| **#2** | 'irritable bowel syndrome':ab,ti OR 'irritable bowel syndromes':ab,ti OR 'syndrome, irritable bowel':ab,ti OR 'syndromes, irritable bowel':ab,ti OR 'colon, irritable':ab,ti OR 'irritable colon':ab,ti OR 'colitis, mucous':ab,ti OR 'colitides, mucous':ab,ti OR 'mucous colitides':ab,ti OR 'mucous colitis':ab,ti OR 'ibs':ab,ti | **30,575** |
| **#3** | 'acupuncture'/exp | **57,828** |
| **#4** | 'acupuncture':ab,ti OR 'auriculotherapy':ab,ti OR 'acupressure':ab,ti OR 'electroacupuncture':ab,ti OR 'electro acupuncture':ab,ti OR 'acupuncture points':ab,ti OR 'acupuncture point':ab,ti OR 'acupoint':ab,ti OR 'acupoints':ab,ti OR 'transcutaneous electric stimulation':ab,ti OR 'percutaneous electric nerve stimulation':ab,ti OR 'tens':ab,ti OR 'transcutaneous electrical stimulation':ab,ti OR 'percutaneous electrical nerve stimulation':ab,ti OR 'transcutaneous electrical nerve stimulation':ab,ti OR 'transcutaneous nerve stimulation':ab,ti OR 'percutaneous neuromodulation therapy':ab,ti OR 'percutaneous neuromodulation therapies':ab,ti OR 'percutaneous electrical neuromodulation':ab,ti OR 'transdermal electrostimulation':ab,ti OR 'transcutaneous electric nerve stimulation':ab,ti OR 'analgesic cutaneous electrostimulation':ab,ti OR 'scalp acupuncture':ab,ti OR 'acupuncture, ear':ab,ti OR 'auricular acupuncture':ab,ti OR 'ear acupuncture':ab,ti OR 'auricular acupunctures':ab,ti OR 'auricular points':ab,ti OR 'auricular point':ab,ti OR 'ear points':ab,ti OR 'ear point':ab,ti OR 'meridian':ab,ti OR 'dermal needle':ab,ti OR 'plum-blossom needle':ab,ti OR 'seven-star needle':ab,ti OR 'catgut embedding':ab,ti OR 'fire needle':ab,ti OR 'meridians':ab,ti | **75,333** |
| **#5** | #1 OR #2 | **39,374** |
| **#6** | #3 OR #4 | **89,761** |
| **#7** | ('clinical':ti,ab AND 'trial':ti,ab) OR 'clinical trial'/exp OR random* OR 'drug therapy':lnk | **7,060,546** |
| **#8** | #5 AND #6 AND #7 | **501** |

| **Cochrane library** | | |
| --- | --- | --- |
| **#1** | MeSH descriptOR: [Irritable Bowel Syndrome] explode all trees | **1,638** |
| **#2** | (Irritable Bowel Syndromes):ti,ab,kw OR (Syndrome, Irritable Bowel ):ti,ab,kw OR (Syndromes, Irritable Bowel):ti,ab,kw OR (Colon, Irritable):ti,ab,kw OR (Irritable Colon):ti,ab,kw OR (Colitis, Mucous):ti,ab,kw OR (Colitides, Mucous):ti,ab,kw OR (Mucous Colitides):ti,ab,kw OR (Mucous Colitis):ti,ab,kw OR (IBS):ti,ab,kw | **5,297** |
| **#3** | MeSH descriptOR: [Acupuncture] explode all trees | **713** |
| **#4** | (Acupuncture):ti,ab,kw OR (Auriculotherapy):ti,ab,kw OR (Acupressure ):ti,ab,kw OR (Transcutaneous Electric Nerve Stimulation ):ti,ab,kw OR (Electroacupuncture ):ti,ab,kw OR (Electro Acupuncture ):ti,ab,kw OR (Acupuncture Points ):ti,ab,kw OR (Acupuncture Point ):ti,ab,kw OR (Acupoint ):ti,ab,kw OR (Acupoints ):ti,ab,kw | **24,827** |
| **#5** | (Transcutaneous Electric Nerve Stimulation):ti,ab,kw OR (Transcutaneous Electric Stimulation):ti,ab,kw OR (Percutaneous Electric Nerve Stimulation):ti,ab,kw OR (TENS):ti,ab,kw OR (Transcutaneous Electrical Stimulation):ti,ab,kw OR (Percutaneous Electrical Nerve Stimulation):ti,ab,kw OR (Transcutaneous Electrical Nerve Stimulation):ti,ab,kw OR (Transcutaneous Nerve Stimulation):ti,ab,kw OR (Percutaneous Neuromodulation Therapy):ti,ab,kw OR (Percutaneous Neuromodulation Therapies):ti,ab,kw | **5915** |
| **#6** | (Percutaneous Electrical Neuromodulation):ti,ab,kw OR (Transdermal Electrostimulation):ti,ab,kw OR (Transcutaneous Electric Nerve Stimulation):ti,ab,kw OR (Analgesic Cutaneous Electrostimulation):ti,ab,kw OR (SCALP ACUPUNCTURE):ti,ab,kw OR (Acupuncture, Ear):ti,ab,kw OR (Auricular Acupuncture):ti,ab,kw OR (Ear Acupuncture):ti,ab,kw OR (Auricular Acupunctures):ti,ab,kw | **3,683** |
| **#7** | (Auricular Points):ti,ab,kw OR (Auricular Point):ti,ab,kw OR (Ear Points):ti,ab,kw OR (Ear Point):ti,ab,kw OR (Meridian):ti,ab,kw OR (Dermal Needle):ti,ab,kw OR (Plum-blossom Needle):ti,ab,kw OR (Seven-star Needle):ti,ab,kw OR (Catgut Embedding):ti,ab,kw OR (Fire Needle):ti,ab,kw OR (Meridians):ti,ab,kw | **3,929** |
| **#8** | #1 OR #2 | **5297** |
| **#9** | #3 OR #4 OR #5 OR #6 OR #7 | **29,761** |
| **#10** | #8 AND #9 | **225** |

| **Web of science** | | |
| --- | --- | --- |
| **#1** | TS= (Irritable Bowel Syndrome OR Irritable Bowel Syndromes OR Syndrome, Irritable Bowel OR Syndromes, Irritable Bowel OR Colon, Irritable OR Irritable Colon OR Colitis, Mucous OR Colitides, Mucous OR Mucous Colitides OR Mucous Colitis OR IBS) | **27,508** |
| **#2** | TS=(Acupuncture OR Auriculotherapy OR Acupressure OR Transcutaneous Electric Nerve Stimulation OR Electroacupuncture OR Electro Acupuncture OR Acupuncture Points OR Acupuncture Point OR Acupoint OR Acupoints OR Transcutaneous Electric Nerve Stimulation OR Transcutaneous Electric Stimulation OR Percutaneous Electric Nerve Stimulation OR TENS OR Transcutaneous Electrical Stimulation OR Percutaneous Electrical Nerve Stimulation OR Transcutaneous Electrical Nerve Stimulation OR Transcutaneous Nerve Stimulation OR Percutaneous Neuromodulation Therapy OR Percutaneous Neuromodulation Therapies OR Percutaneous Electrical Neuromodulation OR Transdermal Electrostimulation OR Transcutaneous Electric Nerve Stimulation OR Analgesic Cutaneous Electrostimulation OR SCALP ACUPUNCTURE OR Acupuncture, Ear OR Auricular Acupuncture OR Ear Acupuncture OR Auricular Acupunctures OR Auricular Points OR Auricular Point OR Ear Points OR Ear Point OR Meridian OR Dermal Needle OR Plum-blossom Needle OR Seven-star Needle OR Catgut Embedding OR Fire Needle OR Meridians) | **88,309** |
| **#3** | TS=(RANDomized Controlled Trial OR RANDomized Controlled Trials OR RANDomised Controlled Trials OR RCT OR RCTs) | **471,504** |
| **#4** | #1 AND #2 AND #3 | **141** |
